# Avian Neo-Sex Chromosomes Reveal Dynamics of Recombination Suppression and W Degeneration

**DOI:** 10.1093/molbev/msab277

**Published:** 2021-09-20

**Authors:** Hanna Sigeman, Maria Strandh, Estelle Proux-Wéra, Verena E Kutschera, Suvi Ponnikas, Hongkai Zhang, Max Lundberg, Lucile Soler, Ignas Bunikis, Maja Tarka, Dennis Hasselquist, Björn Nystedt, Helena Westerdahl, Bengt Hansson

**Affiliations:** 1 Department of Biology, Lund University, Lund, Sweden; 2 Department of Biochemistry and Biophysics, National Bioinformatics Infrastructure Sweden, Science for Life Laboratory, Stockholm University, Solna, Sweden; 3 Department of Medical Biochemistry and Microbiology, National Bioinformatics Infrastructure Sweden, Science for Life Laboratory, Uppsala University, Uppsala, Sweden; 4 Department of Immunology, Genetics and Pathology, Science for Life Laboratory, Uppsala Genome Center, Uppsala University, Uppsala, Sweden; 5 Department of Cell and Molecular Biology, National Bioinformatics Infrastructure Sweden, Science for Life Laboratory, Uppsala University, Uppsala, Sweden

**Keywords:** sex chromosome, neo-sex chromosome, recombination, degeneration, vertebrate, bird

## Abstract

How the avian sex chromosomes first evolved from autosomes remains elusive as 100 million years (My) of divergence and degeneration obscure their evolutionary history. The Sylvioidea group of songbirds is interesting for understanding avian sex chromosome evolution because a chromosome fusion event ∼24 Ma formed “neo-sex chromosomes” consisting of an added (new) and an ancestral (old) part. Here, we report the complete female genome (ZW) of one Sylvioidea species, the great reed warbler (*Acrocephalus arundinaceus*). Our long-read assembly shows that the added region has been translocated to both Z and W, and whereas the added-Z has retained its gene order the added-W part has been heavily rearranged. Phylogenetic analyses show that recombination between the homologous added-Z and -W regions continued after the fusion event, and that recombination suppression across this region took several million years to be completed. Moreover, recombination suppression was initiated across multiple positions over the added-Z, which is not consistent with a simple linear progression starting from the fusion point. As expected following recombination suppression, the added-W show signs of degeneration including repeat accumulation and gene loss. Finally, we present evidence for nonrandom maintenance of slowly evolving and dosage-sensitive genes on both ancestral- and added-W, a process causing correlated evolution among orthologous genes across broad taxonomic groups, regardless of sex linkage.

## Introduction

Sex chromosomes have evolved from autosomes many times across the animal and plant kingdoms and have been studied intensely not only for their role in sex determination but also for their other distinguishing characteristics, such as loss of recombination and sex-specific evolutionary pressures (Bachtrog et al. 2011; Abbott et al. 2017). Traditionally, most research on sex chromosomes has been done on species with highly heteromorphic sex chromosomes. However, the degeneration of such old sex chromosomes obscures the genomic signatures of their early evolutionary history. To learn more about the transition of sex chromosomes from their autosomal origin, newly formed sex chromosomes (formed de novo or by turnovers) or partially newly formed sex chromosomes (neo-sex chromosomes) are more suitable systems (Wright et al. 2016; Ponnikas et al. 2018).

In birds, the sex chromosomes (Z and W) originated more than 100 Ma (Zhou et al. 2014) as recombination became suppressed around the sex-determining gene (*DMRT1*; Smith et al. 2009). Since then, the sex chromosome copies have ceased to recombine along most of their length in the majority of species, except in some paleognaths (e.g., common ostrich *Struthio camelus*), resulting in heavy differentiation between Z and W with weak signatures of their shared origin and few surviving genes on the degenerated sex-limited W chromosome (Zhou et al. 2014; Smeds et al. 2015; Bellott et al. 2017). Birds have highly stable karyotypes with few inter-chromosomal rearrangements compared with other vertebrates (Ellegren 2010), and the Z chromosome has been shown to share synteny across its entire length even between widely different clades of birds (Nanda et al. 2008). However, there are a few exceptions, so far found among passerines, cuckoos and parrots, where autosome–sex chromosome fusions have enlarged the original sex chromosomes and formed neo-sex chromosomes (Brooke et al. 2010; Pala et al. 2012; Gan et al. 2019; Sigeman et al. 2019, 2020; Dierickx et al. 2020; Kretschmer et al. 2020; Huang et al. 2021). Such events often lead to an extension of recombination suppression to include also the translocated chromosomal region, which then becomes bound by the same evolutionary processes as the original sex chromosome (Bachtrog 2013). These neo-sex chromosomes provide excellent opportunities to study the drivers of recombination suppression between avian sex chromosomes and allow us to study rates of evolution between sex-linked genetic regions of different ages.

The songbird superfamily Sylvioidea (sensu lato; Moyle et al. 2016; Oliveros et al. 2019) split from other songbirds ∼24 Ma and has since undergone one of the fastest radiations within birds, with over 1,200 extant species (Alström et al. 2006). All Sylvioidea birds studied so far, that is, species representatives of 11 of the 22 families within Sylvioidea (Pala et al. 2012; Leroy et al. 2019; Sigeman et al. 2019, 2020), share a unique karyotype feature: a neo-sex chromosome pair formed by a chromosomal fusion between the ancestral sex chromosomes and a part of chromosome 4A (according to chromosome naming from the zebra finch, *Taeniopygia guttata*; Warren et al. 2010). This fusion has thus added new genomic material to the sex chromosomes of Sylvioidea birds, characterized by less Z-to-W differentiation and W degeneration compared with the ancestral part. Here, we present a detailed study of the evolutionary history of this neo-sex chromosome in a Sylvioidea species belonging to the family Acrocephalidae, the great reed warbler (*Acrocephalus arundinaceus*). By constructing a high-quality annotated reference genome from a female great reed warbler, containing both a Z and W chromosome, we can study both the chromosomal structure of this fusion event and how the previously autosomal region has evolved in this novel sex-linked environment in terms of recombination suppression, repeat accumulation, and gene differentiation.

## Results

### Sequencing, Assembly, Annotation, and Synteny

**Fig. 1.**
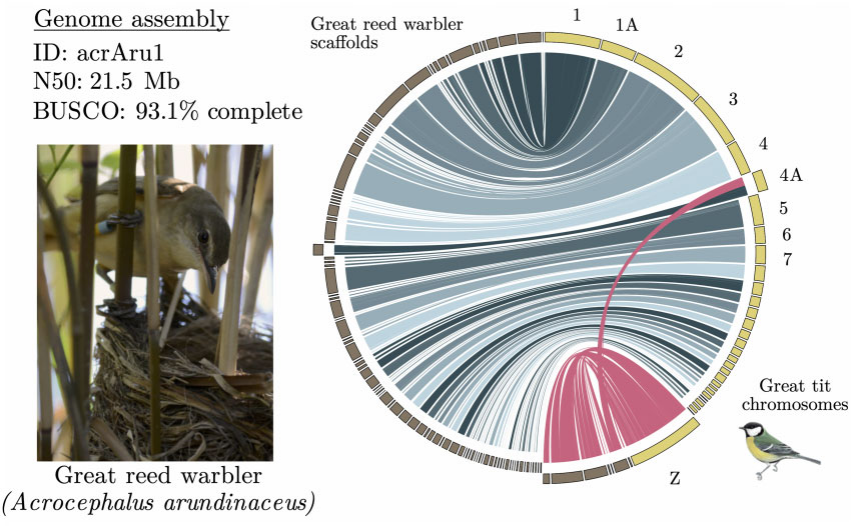
The great reed warbler and its genome assembly. Synteny analysis between the great reed warbler (scaffolds in brown) and the great tit genome (chromosomes in yellow) showing a single inter-chromosomal rearrangement: the autosome–sex chromosome fusion unique to Sylvioidea songbirds involving the Z chromosome and a 9.6-Mb part of chromosome 4A. Links involving sex-linked great reed warbler scaffolds are in pink, and links involving autosomal scaffolds are colored in different shades of blue. Great reed warbler scaffolds that share synteny (filtered to exclude short matches, see Materials and Methods) with great tit chromosomes Z and 4A, and these two chromosomes, are scaled to twice their actual size for illustrative purposes. Photograph of a female great reed warbler by August Thomasson.

We sequenced high-molecular weight DNA of a female great reed warbler from our long-term study population in southern central Sweden using a combination of long-read, linked-read, short-read sequencing, and optical data, to reconstruct its genome de novo (see details on raw data in supplementary table 1*a*, Supplementary Material online and genome statistics correlating to each stage in the genome assembly process in supplementary tables 2 and 3, Supplementary Material online). We also used information from a linkage map analysis (Ponnikas et al. 2020), based on a multigeneration pedigree of great reed warblers genotyped with Restriction site–Associated DNA (RAD) sequencing (Hansson et al. 2018), to identify and correct assembly errors (see Materials and Methods). The final assembly (acrAru1) consisted of 3,013 scaffolds and had an N50 of 21.4 Mb (fig. 1 and supplementary table 2, Supplementary Material online). The number of conserved avian single-copy orthologs (*n *=* *4,915) was assessed with BUSCO v.3.0.2 (aves_odb9 data set; Simão et al. 2015). The final assembly had 93.1% complete genes (supplementary table 3, Supplementary Material online), which is similar to other long-read sequenced genomes (e.g., 85–94% in different species of birds of paradise, family Paradisaeidae; Xu et al. 2019; Peona et al. 2021). The total repeat content of the final draft assembly was 16.3%, with long terminal repeats (LTRs) as the most common type of repeat (6.9%) followed by long interspersed nuclear elements (5.0%; supplementary table 4, Supplementary Material online). The genome assembly was annotated with 22,524 genes. Scaffolds belonging to the nonrecombining part of the Z and the W chromosome, respectively, were detected by evaluating sex-specific differences in read coverage and/or heterozygosity (supplementary table 5, Supplementary Material online) using whole-genome resequencing data from five male (ZZ) and five female (ZW) great reed warblers (supplementary table 1*b*, Supplementary Material online) which were aligned to the genome assembly.

A comparison between the genomes of the great reed warbler and the great tit (*Parus major*), which is the closest relative to the great reed warbler with a near-complete chromosome-level assembly (lacking only a few microchromosomes and the W chromosome), showed largely conserved synteny (fig. 1). We detected a single well-supported interchromosomal rearrangement: the autosome–sex chromosome fusion unique to Sylvioidea songbirds involving chromosome Z and approximately half (0–9.6 Mb) of chromosome 4A (20.7 Mb in total; fig. 1) through a scaffold bridging over between these two chromosome regions (see also below). This confirms the fusion between chromosome Z and chromosome 4A that has occurred basally in the Sylvioidea clade (Pala et al. 2012; Sigeman et al. 2020). The intrachromosomal collinearity between the two species was disrupted for several macro- as well as microchromosomes (supplementary fig. 1, Supplementary Material online). Throughout this article, we refer to the great reed warbler neo-sex chromosome region sharing synteny with other songbird Z chromosomes as the ancestral sex chromosome region (abbreviated as ancestral-Z and -W for the two sex chromosome copies, respectively), and the translocated region sharing synteny with chromosome 4A as the added sex chromosome region (abbreviated as added-Z and -W, respectively).

### Sex Chromosome Structure and Cross-Species Homology

**Fig. 2.**
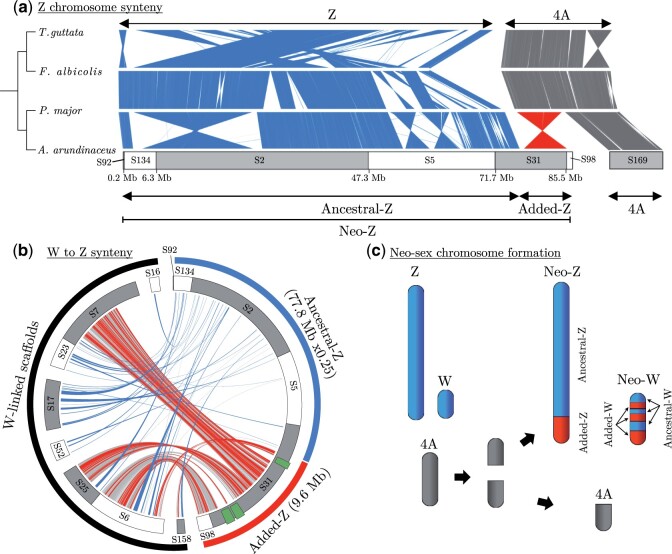
Structure of the great reed warbler Z and W neo-sex chromosomes. (*a*) Chromosome alignments of chromosome Z (blue) and 4A (gray) in four species of passerines, with the part of 4A representing the added-Z region in the great reed warbler indicated (red). Phylogenetic relationships between the species are depicted in a cladogram (left). Linkage map-oriented Z-linked great reed warbler scaffolds (*n* = 6) are indicated below the cross-species syntenies as gray and white segments with scaffold IDs. Scaffold169 (“S169”), which aligns with chromosome 4A and segregates as a separate autosomal chromosome, is also indicated. (*b*) Syntenic regions between the great reed warbler W-linked and Z-linked (gray and white alternating) scaffolds. The Z-linked scaffolds belonging to the ancestral sex chromosome region (blue) are scaled to 25% of their true size for illustrative purposes. Two pairs of W scaffolds shown to be physically linked (by linked-read data from a different female; see main text) are placed next to each other without gaps. The gray links show syntenic information on a genomic level, whereas chromosomal positions of gametologous (ZW) gene pairs are shown as blue links for ancestral sex chromosome genes and red links for added sex chromosome genes. Note that four W-linked scaffolds have genes with orthologs on both the ancestral and added sex chromosome regions, strongly suggesting that the ancestral-W and added-W are physically connected. Green symbols mark putative W-deletions (see main text). (*c*) A graphic representation of the fusion event forming the Sylvioidea neo-sex chromosome system.

We identified 22 Z-linked scaffolds (total length of 88.7 Mb; mean length 4 Mb; supplementary fig. 2 and supplementary table 5, Supplementary Material online) in the great reed warbler genome. Annotated genes were found on eight of these scaffolds (total length of 88.2 Mb; supplementary table 6*a*, Supplementary Material online), whereas the remaining scaffolds were short (0.002–0.11 Mb) and contained no genes. By searching for syntenies between these Z-linked scaffolds and the genomes of zebra finch and collared flycatcher (*Ficedula albicollis*), we determined that nine scaffolds (total length 73.3 Mb) shared synteny only with the ancestral Z chromosome and that one scaffold shared synteny with only chromosome 4A (supplementary table 6*b*, Supplementary Material online). One scaffold (Scaffold31; fig. 2*a* and supplementary table 6*b*, Supplementary Material online), however, shared synteny with the end of chromosome Z (position 67.6–72.9 Mb) as well as a large part of chromosome 4A (position 9.6–0.9 Mb). This locates the fusion point in the zebra finch genome to chromosome Z position 72.9 Mb and chromosome 4A position 9.6 Mb (fig. 2*c*; Pala et al. 2012; Sigeman et al. 2020).

Moreover, six of the 22 Z-linked scaffolds, which together cover 98.6% of the total length of the Z-linked scaffolds, were possible to order with our RAD-based linkage map analysis (fig. 2*a* and supplementary table 7, Supplementary Material online; also see Ponnikas et al. 2020). Linkage mapping further identified a scaffold (Scaffold217; 0.9 Mb in length) containing the pseudoautosomal region (PAR), that is, the region where the Z and W chromosomes recombine (see Ponnikas et al. 2020). Alignments to the genomes of zebra finch, collared flycatcher and great tit confirmed that the great reed warbler Z chromosome consists of an initial part corresponding to the PAR (0–0.9 Mb; which is not included in the Z chromosome sequence of the other species and thus not shown here), a large central part corresponding to the ancestral Z (0.9–77.8 Mb), and a final part, the added region, corresponding to the first half (9.6 Mb) of chromosome 4A (77.8–87.5 Mb; fig. 2*a* and supplementary tables 6 and 7, Supplementary Material online). The linkage map analysis further showed that the scaffold corresponding to the second half of chromosome 4A (Scaffold169; 9.6–20.7 Mb) segregates autosomally in the great reed warbler (fig. 2*a*), that is, confirming the fission of chromosome 4A in Sylvioidea (fig. 2*c*). In addition to the fusion between the ancestral Z and chromosome 4A in Sylvioidea, three large inversions broke the collinearity between the great reed warbler and the great tit and the collared flycatcher Z chromosomes, whereas the zebra finch differed by several Z chromosome rearrangements as described previously (Itoh et al. 2006; Kawakami et al. 2014) (fig. 2*a*).

The 50 W-linked scaffolds had a total length of 30.2 Mb (mean length 0.6 Mb). Of these, 15 scaffolds (with a total length of 28.0 Mb; mean length 1.9 Mb) were present in the gene annotation whereas the remaining ones were short (0.001–0.4 Mb) and contained no annotated genes (supplementary fig. 2 and supplementary table 5, Supplementary Material online). We identified and manually curated 153 gametologous (ZW) gene pairs in the great reed warbler gene annotation; 42 pairs from the ancestral and 111 from the added sex chromosome region (supplementary table 8, Supplementary Material online). Of the 42 ancestral W genes, 36 had previously been described in a detailed study of the W chromosome in the collared flycatcher (Smeds et al. 2015; supplementary table 9, Supplementary Material online). The collared flycatcher annotation included an additional eight W genes of which six were present in our great reed warbler annotation under the same gene name as in the flycatcher but did not have enough evidence to be classified as orthologs and were therefore not included here (see Materials and Methods). We aligned the 15 W-linked scaffolds to the great reed warbler Z-linked scaffolds, and cross-positioned the 153 ZW gametologs, and found that four great reed warbler W scaffolds showed substantial sequence similarity with, and contained many genes with gametologs on, both the ancestral-Z and added-Z chromosome regions, whereas the remaining W scaffolds only contained sequences with similarity to ancestral-Z (fig. 2*b*). The shared homology of four W-linked scaffolds to both ancestral-Z and added-Z strongly supports that the added-W region has fused with the ancestral-W region, and has subsequently been intrachromosomally rearranged (fig. 2*b and c*). A de novo assembly based on linked-read data from another great reed warbler female (supplementary tables 1*b*, 2, and 3, Supplementary Material online) showed evidence for two pairs of W scaffolds being physically linked (fig. 2*b*) and provided independent evidence for the correctness of three of the four scaffolds sharing synteny with both the ancestral-Z and added-Z chromosome regions. This confirms the presence of a single enlarged W chromosome in Sylvioidea birds (consisting of the ancestral W plus a part of chromosome 4A) as opposed to the alternative of two separate W chromosomes (i.e., a ZW_1_W_2_ system). We identified three putative W deletions on the added sex chromosome region (total size ∼2.4 Mb, indicated by green rectangles in fig. 2*b*), as parts of the Z-linked Scaffold31 share no synteny with corresponding W-linked scaffolds (fig. 2*b*). Despite these deletions, the total length of great reed warbler W scaffolds with synteny to added-Z (10.6 Mb) is larger than the corresponding Z scaffolds (9.6 Mb). To conclude, our data clearly support a fusion of the ancestral songbird sex chromosome and a part of chromosome 4A (inverted) in the great reed warbler, and that this fusion involves both Z and W, forming young (∼24 My; see below) and enlarged Z and W neo-sex chromosomes.

### Evolution of Recombination Suppression

Our genomic and linkage map data show that the neo-sex chromosome pair has ceased to recombine over most of the ancestral part (except the PAR) and over the whole added region in present-day great reed warblers. To estimate when the different parts of the great reed warbler sex chromosomes ceased to recombine, we constructed maximum-likelihood gene trees (see Materials and Methods) of gametologous gene pairs from six Sylvioidea species (including the great reed warbler) extracted from short-read sequences generated in this study (supplementary table 1*b*, Supplementary Material online). For the added part of the neo-sex chromosome, we evaluated recombination suppression within Sylvioidea using either the great tit or zebra finch as an outgroup. For the ancestral part of the neo-sex chromosome, we evaluated recombination suppression throughout the avian phylogeny by including Z chromosome sequences of six additional non-Sylvioidea species in the analysis and using the green anole (*Anolis carolinensis*) as an outgroup. The species were selected to widely represent divergence times to the great reed warbler. A dated phylogeny was constructed using a set of autosomal gene sequences and four calibration points (see Materials and Methods), which estimated the split between non-Sylvioidea and Sylvioidea (i.e., the node between *Parus major* and* Panurus biarmicus*) to ∼24 My (supplementary fig. 3, Supplementary Material online).

**Fig. 3.**
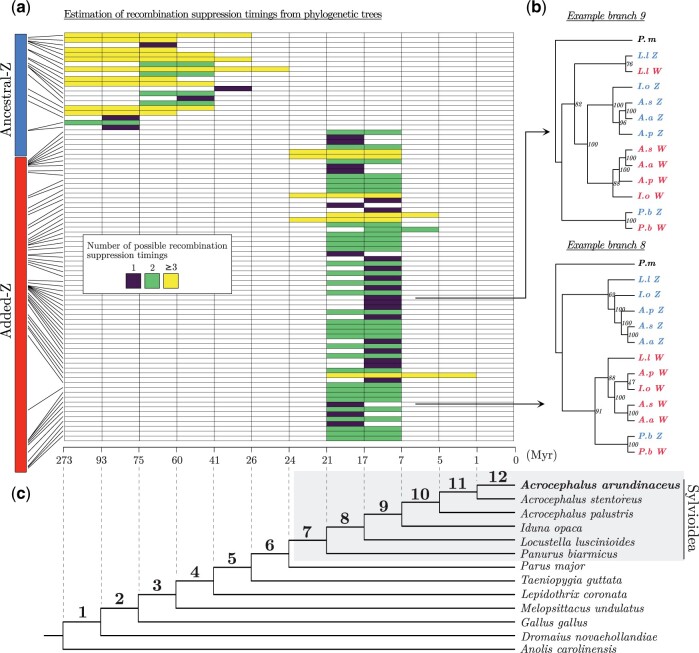
Timing of recombination suppression along the Sylvioidea ancestral-Z and added-Z chromosome regions. (*a*) Heatmap showing inferred timings of recombination suppression between great reed warbler gametologs based on the position and clustering of Sylvioidea Z and W sequences in maximum-likelihood gene trees (see main text for details). The chromosome ideogram (left) marks the chromosomal location of each gene (heatmap rows) on the great reed warbler neo-Z chromosome (the ancestral-Z region with 20 analyzed genes, and the added-Z region with 64 analyzed genes, are scaled differently for illustrative purposes). Each heatmap column corresponds to a branch in a dated avian phylogeny; see (*c*). The timing of recombination suppression could be traced to a single branch for some genes (dark purple). For others, the bootstrap values were too low (<70) to confidently discern between multiple possible timings (green or yellow). Supplementary figure 4, Supplementary Material online, shows these timing estimates plotted along the Z-linked and W-linked scaffolds. (*b*) Cladograms showing two genes representing different recombination suppression time points (branch 8 and 9, respectively) with bootstrap values (phylograms for all genes are given in supplementary trees, Supplementary Material online). (*c*) The phylogeny shows dated nodes for the great reed warbler and five additional Sylvioidea species, and six non-Sylvioidea birds and the green anole (Anolis carolinensis). We estimated the split between Sylvioidea and non-Sylvioidea to ∼24 My (supplementary fig. 3, Supplementary Material online).

After sequences from all species had been aligned and short alignments (<700 bp) removed, alignments for 20 and 64 genes in the ancestral and added region, respectively, remained. The timing of recombination suppression of these genes was estimated by evaluating the positioning and clustering of the gametologous gene pairs within and between Sylvioidea species, and in relation to outgroup species, considering only branches with bootstrap values ≥70 (see details in Materials and Methods). For ancestral-Z, all gene trees placed the Sylvioidea W sequences in a monophyletic clade (supporting the expectation that recombination was suppressed prior to their formation). Most trees also had several unsupported branches, which lowers the precision in estimating recombination suppression. Therefore, for many genes the estimated timing of suppression ranges from early in the avian phylogeny to prior to the suboscine passerine blue-crowned manakin (*Lepidothrix coronata*) ∼41 Ma (fig. 3 and supplementary table 10, Supplementary Material online). However, some gene trees support narrower time intervals with either early (>93–75 Ma) or relatively late (60–41 Ma) recombination suppression. These are scattered across the ancestral part of the sex chromosome (fig. 3*a*). This is in line with previous findings and is suggested to be explained by the formation of different layers of recombination suppression, “evolutionary strata,” which since then have become rearranged to different degrees in different lineages (Zhou et al. 2014).

In the much younger added sex chromosome region, the gametologs of *Panurus* cluster together with high bootstrap support in almost all gene trees (94%), which strongly suggests that recombination continued after the split of *Panurus* at ∼21 Ma (fig. 3). Similarly, the W sequences of the four Acrocephalidae spp. (three *Acrocephalus* spp. and *Iduna opaca*) cluster together in almost all gene trees (94%), which strongly suggests that recombination ceased prior to their formation ∼7 Ma (fig. 3). Moreover, the placement of the gametologs of *Locustella luscinioides*, a species with an intermediate phylogenetic position (∼17 Ma), reveals three common patterns: 1) the *Locustella* gametologs cluster with high bootstrap support (25% of genes; revealing that recombination continued after the split of *Locustella* at ∼17 Ma), 2) the *Locustella* W gametolog clusters with the W gametologs of the four Acrocephalidae spp. (14% of genes; supporting that recombination ceased before the Locustella split at ∼17 Ma), and 3) inconclusive placement (61% of genes; the timing of estimated recombination cessation set by the results of *Panurus* and Acrocephalidae to ∼21–7 Ma; fig. 3). The chromosomal position and estimated timing of recombination suppression of each gene are shown in figure 3. Based on these results, we conclude 1) that the added sex chromosome continued to recombine after the fusion event (∼24 Ma), 2) that suppression was initiated at multiple positions, scattered over the chromosome (including both ends; fig. 3), at different time points after the split of *Panurus*, ∼21 Ma, and 3) that recombination suppression was completed over the whole added region prior to the formation of Acrocephalidae, ∼7 Ma (fig. 3). Such a mosaic and gradual pattern of recombination suppression over the added sex chromosome does not support a hypothesis of a single recombination suppression event or a simple linear progression of recombination suppression starting from the fusion point. This spatial pattern of recombination suppression also cannot be explained by rearrangements on the W chromosome, as genes with different estimated timings of recombination suppression occur close together on W-linked scaffolds that align collinearly to Z-linked scaffolds (supplementary fig. 4, Supplementary Material online).

Because synonymous substitutions (d*S*) are expected to evolve at a relatively steady rate, Z-to-W d*S* values can be used as a proxy for timing since recombination suppression. We therefore cross-positioned d*S* values between great reed warbler Z and W gametolog sequences (see section Substitution Rates and Purifying Selection among Gametologs, and Materials and Methods) with their respective position on the neo-Z/neo-W chromosome. The d*S* values were generally higher toward the end of the ancestral Z, in line with evidence from other studies (e.g., Xu et al. 2019). For the added region, the d*S* values were relatively variable even between closely situated genes (supplementary figs. 5 and 6, Supplementary Material online). These results do, similarly to the phylogenetic recombination suppression analysis, not support a simple linear progression of recombination suppression over the added-Z.

### Repeat Accumulation and Loss of Diversity

**Fig. 4.**
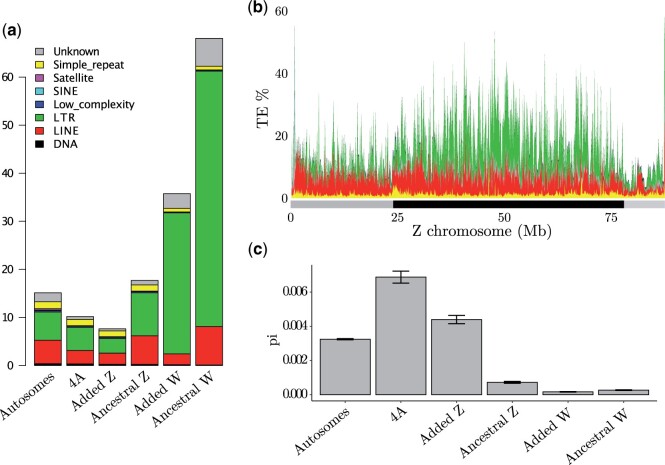
Repeat accumulation and loss of genetic variation on the great reed warbler sex chromosomes. (*a*) Percentages of repeat elements for the entire autosomal assembly (Autosomes), the autosomally segregating part of chromosome 4A (4A), and the added and ancestral parts of Z and W. (*b*) Repeats along the Z chromosome (100 kb windows), following the same color scheme as (a). The alternating gray and black bars below the *x*-axis mark the three strata discussed in the main text (younger ancestral strata, 0–25.0 Mb; older ancestral strata 25.0–77.8 Mb; added strata 77.8–86.6 Mb). (*c*) Nucleotide diversity estimates (mean values across 100 kb windows, with standard deviation) for the genomic regions, calculated from five female great reed warblers.

Recombination suppression between the sex chromosome copies is expected to have severe consequences. In particular, the sex-limited copy (Y and W), with its greatly reduced effective population size and lack of recombination in both sexes, is prone to repeat accumulation, loss of genetic variation, and gene functionality because of increasing influence of genetic drift and decreasing efficiency of selection (Bachtrog et al. 2011). Our analyses of the great reed warbler genome assembly confirm these patterns. The W scaffolds with synteny to the ancestral sex chromosome region consisted of 68.1% repeat elements and had the highest proportion of repeats of all chromosomal regions. The corresponding number for W scaffolds with synteny to the added sex chromosome region, with a more recent history of sex-linkage and recombination suppression, was 35.8% (fig. 4*a* and supplementary table 4, Supplementary Material online). Both of these W regions had a considerably higher repeat content than the autosomal average (15.1%), the ancestral-Z scaffolds (17.7%) and the added-Z scaffolds (7.6%) (supplementary table 4, Supplementary Material online). The repeat content of the added-Z scaffolds was slightly lower than that of the autosomal part of chromosome 4A (10.2%), suggesting that the repeat landscape of this region has so far been little affected by the translocation to the Z chromosome (fig. 4*a*). The central part of the Z chromosome (25.0–77.8 Mb), that is, the end part of ancestral-Z, which contains the older evolutionary strata previously identified in birds (Zhou et al. 2014; Xu et al. 2019), had a higher proportion of repeat elements (19.7%, mean value across 100 kb windows) than the beginning of the chromosome (0.9–25.0 Mb; not including the PAR; 12.2%), and also compared with the added-Z chromosome part (77.8–86.6 Mb; 8.5%) with its much more recent history of recombination suppression (fig. 4*b*). These results support that repeats accumulate continuously on sex chromosomes when recombination ceases and reach very high abundancies on old non-recombining parts of the avian W. Fusion events might be facilitated by repeats, and in line with this we saw a distinct local increase in repeat elements near the fusion point on the great reed warbler Z chromosome (∼77.8 Mb; supplementary fig. 7, Supplementary Material online).

To evaluate how the pattern of nucleotide diversity has been affected by sex linkage, we calculated nucleotide diversity estimates across 100 kb windows separately for five female and five male whole-genome resequenced great reed warblers (supplementary table 1*b*, Supplementary Material online). The analysis showed a nucleotide diversity for autosomes of 0.0032 and 0.0033 in females and males, respectively (fig. 4*c* shows data for females; supplementary table 11, Supplementary Material online gives data for both sexes), and revealed much lower levels for both the ancestral Z and W chromosome regions (Z: 0.0007 in males and females; W: 0.0003 in females; fig. 4*c* and supplementary table 11, Supplementary Material online); a pattern seen also in other bird species (reviewed in Wilson Sayres 2018). Both the added-W and -Z had lower nucleotide diversity levels (W: 0.0002 in females, Z: 0.0044 in females and 0.0040 in males; fig. 4*c* and supplementary table 11, Supplementary Material online) than the autosomally segregating part of chromosome 4A (0.0069 both in males and females; fig. 4*c* and supplementary table 11, Supplementary Material online). The diversity of the added-Z was, however, higher than the autosomal average. Despite its comparably recent history as sex linked, the added-W diversity level was as low as that of the ancestral-W. The nucleotide diversity values from females differed significantly between all chromosome types (fig. 4*c*; Dunn Kruskal Wallis multiple comparison with Benjamini–Hochberg correction; *P*_adjusted_ between < 0.00001 and 0.031), except between ancestral- and added-W (*P*_adjusted_ = 0.400; supplementary table 11, Supplementary Material online).

### Substitution Rates and Purifying Selection among Gametologs

Next, we analyzed the rate of synonymous (d*S*) and nonsynonymous substitutions (d*N*) of ZW gametologs to investigate whether purifying selection acts on sex-linked genes. We aligned the Z- and W-sequences of each of the great reed warbler ZW gametologs (see Evolution of Recombination Suppression section) together with orthologous gene copies from the zebra finch gene annotation (Z-linked genes for the ancestral region and chromosome 4A-linked genes for the added region) and calculated d*S*, d*N*, and d*N*/d*S* for each pairwise comparison and gene (see Materials and Methods). After filtering (minimum alignment length: 500 bp; maximum d*S*: 3), 35 gametologs from the ancestral sex chromosome region remained. For the added region, 79 gametologs remained after filtering (supplementary table 12, Supplementary Material online).

As expected from its much older history of sex linkage, the ancestral sex chromosome region showed higher d*S* and d*N* between the Z and W gametologs (median d*S* = 0.263; median d*N* = 0.026) compared with the added region (median d*S* = 0.078; median d*N* = 0.013) (Mann–Whitney *U* test; d*S*: *U *=* *177, *P *=* *1.33 × 10^−13^; d*N*: *U *=* *933, *P *=* *0.006; supplementary fig. 8*a and b*, Supplementary Material online). However, the d*N*/d*S* ratio was significantly higher for the added region (median d*N*/d*S*: 0.155; range: 0.001–0.890) than for the ancestral region (median d*N*/d*S*: 0.109; range: 0.001–0.289; *U *=* *1776, *P *=* *0.016; supplementary fig. 8*c*, Supplementary Material online). This result suggests that purifying selection is generally acting on sex-linked gametologs (d*N*/d*S* < 1 for all gametologs; supplementary table 12, Supplementary Material online), but particularly strongly so on genes being maintained both on the Z and the W chromosome over very long periods of time.

Then, we compared the substitution rates between each of the great reed warbler Z and W gametolog and the corresponding zebra finch ortholog (i.e., Z to zebra finch vs. W to zebra finch). For gametologs on the added region, where zebra finch chromosome 4A orthologs are analyzed, there was no difference between d*S* for W to zebra finch and d*S* for Z to zebra finch (Wilcoxon signed-rank test: *V = *1699, *P *=* *0.56), whereas the W gametologs showed higher d*N* and d*N*/d*S* to zebra finch than did Z gametologs (d*N*: *V *=* *2582, *P *=* *1.01 × 10^−9^; d*N*/d*S*: *V *=* *2588, *P *=* *5.82 × 10^−9^). Similarly, the genes on the ancestral sex chromosome had higher d*S*, d*N* (*V = *630, *P = *5.82 × 10^−11^ in both cases), and d*N*/d*S* values (*V *=* *524, *P *=* *1.11 × 10^−4^) between W to zebra finch than between Z to zebra finch. These results are in line with purifying selection being less efficient on the W than on the Z chromosome. Note, however, that the analysis of gametologs on the ancestral region is biased toward higher divergence values for W-linked genes as recombination suppression on the ancestral sex chromosome precedes the split between the zebra finch and great reed warbler, which makes the finch and warbler Z orthologs share more recent history.

### Conserved and Dose-Sensitive Genes Maintain W Gametologs

**Fig. 5.**
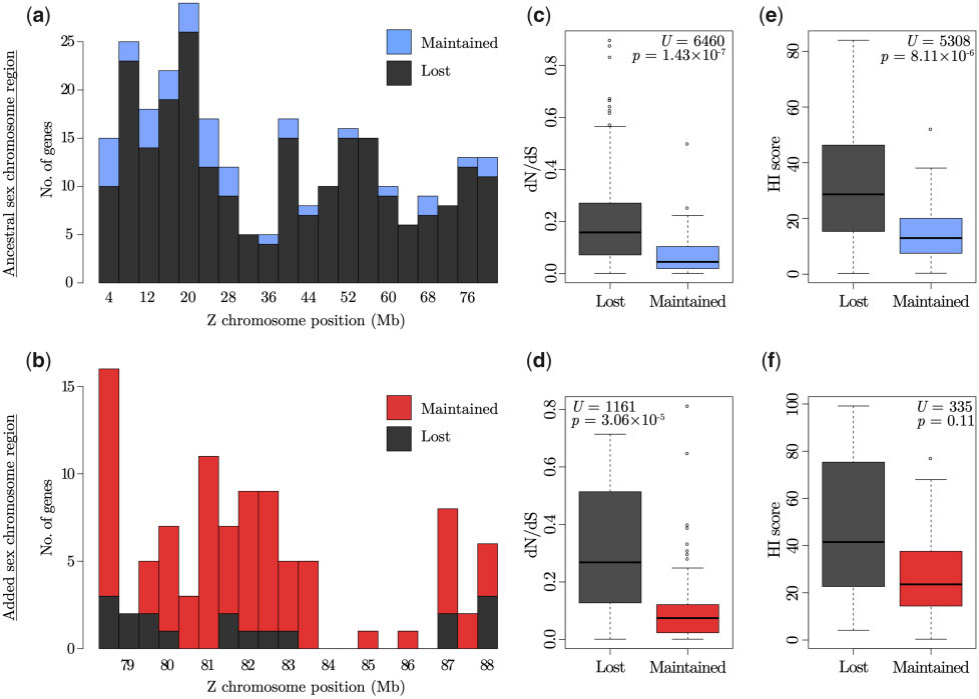
Nonrandom loss of W gametologs on the great reed warbler neo-sex chromosome. (*a*, *b*) Chromosome position of Z genes with lost (black) or maintained W gene copy (blue/red) on (*a*) the ancestral and (*b*) added part of the sex chromosome. (*c*–*f*) The ratio of nonsynonymous to synonymous substitution rates (d*N*/d*S*) between orthologous great reed warbler and zebra finch genes (*c*, *d*), and haploinsufficiency (HI) scores (*e*, *f*), for Z genes with and without a W copy on the ancestral (*c*, *e*; blue) and the added (*d*, *f*; red) sex chromosome region. The median value is marked by the black line in each box, and the upper and lower hinges signify the first and third quartiles. The whiskers extend to no more than 1.5× of the interquartile range from each hinge.

As the W chromosome degenerates, many W gametologs are lost, and the strong signature of purifying selection on sex-linked genes (supported by the d*N*/d*S* values above) suggests that the ones being maintained on the W are biased toward genes with conserved functions. To test this, we contrasted substitution rates between great reed warbler Z-linked genes and the corresponding zebra finch orthologs for 1) Z-linked genes where the W-linked gametolog has become lost, and 2) Z-linked genes where the W gametolog remains in the great reed warbler assembly. We did this for both the added and ancestral sex chromosome region, where in the latter analysis we also included 243 (nonmanually curated) genes from the ancestral Z chromosome of which there was no W copy in the gene annotation. After alignment of sequences and removing short (<500 bp) alignments, we analyzed 273 genes from the ancestral sex chromosome (35 with and 238 without a W gene copy), and 97 genes from the added sex chromosome (79 with and 18 without a W gene copy). Z genes with a lost W gene copy were distributed along the entire ancestral (fig. 5*a*) as well as added (fig. 5*b*) sex chromosome region.

On the ancestral part of the neo-sex chromosome, there was no difference in d*S* values between Z genes with (median = 0.115) and without (median = 0.119) a W gametolog (Mann–Whitney *U* test: *U *=* *4795, *P *=* *0.15), whereas both the d*N* (lost W, median = 0.020; maintained W, median = 0.005; *U *=* *6575, *P *=* *3.30 × 10^−8^) and d*N*/d*S* (lost W, median = 0.159; maintained W, median = 0.038; *U *=* *6460, *P *=* *1.43 × 10^−7^) values were significantly higher for Z genes without a W copy (fig. 5*c* and supplementary table 12, Supplementary Material online). The results were similar for the added part of the sex chromosome: the d*S* values did not differ between Z genes with (median = 0.128) or without (median = 0.130) a W gametolog (*U *=* *703, *P *=* *0.94), whereas the d*N* values was significantly higher for Z-linked genes without a W copy (lost W, median = 0.031; maintained W, median = 0.010; *U *=* *1129.5, *P *=* *1.05 × 10^−4^), and the same was true for the d*N*/d*S* values (lost W, median = 0.268; maintained W, median = 0.074; *U *=* *1161, *P *=* *3.06 × 10^−5^; fig. 5*d* and supplementary table 12, Supplementary Material online). The lower d*N* and d*N*/d*S* values for Z genes where the W gametolog has remained supports that these sex-linked genes are under strong purifying selection for being functionally conserved.

The W chromosome is further expected to be enriched for dose sensitive genes, as haploinsufficiency (HI) will pose problems for the heterogametic sex when one gene copy, in this case the W gametolog, is functionally lost. We downloaded predicted HI scores based on human studies (no equivalent data are available for birds), where a lower HI value describes that a diploid gene is less able to retain its full function when a mutation disrupts one of its gene copies, from the DECIPHER database (https://decipher.sanger.ac.uk/; accessed January 11, 2019) for orthologs of our Z-linked great reed warbler genes. In line with predictions (and despite the large phylogenetical distance between humans and warblers that could have compromised the relevance of using human-based HI scores in our analysis), we found for the ancestral sex chromosome region that genes with a remaining W copy had lower HI scores (*n *=* *33; median HI score = 12.95) than genes that had lost their W copy (*n *=* *217; median HI score = 28.66; Mann–Whitney *U* test: *U *=* *5308, *P *=* *8.11 × 10^−6^; fig. 5*e* and supplementary table 13, Supplementary Material online). For the added sex chromosome part, a similar but nonsignificant pattern was found (*n *=* *62 genes with a W copy: median HI score = 23.59; *n *=* *8 genes without a W copy: median HI score = 41.52; *U *=* *335, *P *=* *0.11; fig. 5*f* and supplementary table 13, Supplementary Material online).

### Evolution of Sex-Linked Genes Is Highly Predictable

**Fig. 6.**
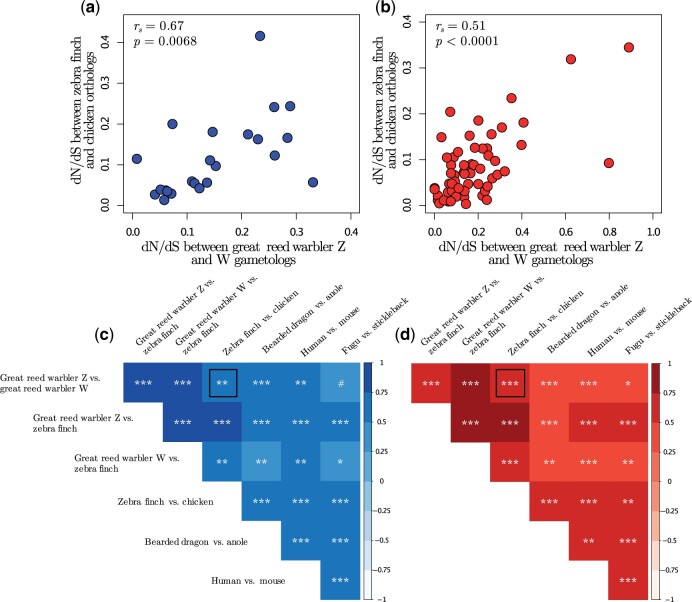
Correlated rate of evolution of orthologs in vertebrates. (*a*, *b*) Relationship between d*N*/d*S* values for great reed warbler Z and W gametologs, and d*N*/d*S* values for zebra finch and chicken Z orthologs, for genes located on (*a*) the ancestral and (*b*) the added sex chromosome region. Correlation coefficients and *P* values are shown. (*c*, *d*) Correlation coefficients (heat map) and significance levels (symbols) for pairwise correlations of seven sets of d*N*/d*S* values between orthologs (or gametologs) to genes located on (*c*) the ancestral and (*d*) the added sex chromosome region. Symbols signify *P* values of < 0.1 (#), < 0.05 (*), < 0.01 (**), and < 0.001 (***). The black squares mark the significance levels corresponding to the data shown in panels (*a*) and (*b*), which are highlighted as two examples of d*N*/d*S* correlations. The seven sets of d*N*/d*S* values came from comparisons of 1) great reed warbler gametologous gene pairs (Z vs. W), 2 and 3) each of the great reed warbler gametologs to zebra finch orthologs (great reed warbler Z vs. zebra finch, and great reed warbler W vs. zebra finch), and 4–7) orthologs for one species-pair each of birds (zebra finch and chicken), reptiles (anole and bearded dragon), mammals (human and mouse) and fish (stickleback and fugu), respectively.

Finally, we evaluated the consequences of sex-linkage per se for the evolutionary trajectory of the genes on the Sylvioidea neo-sex chromosome by making use of the rich source of available genomic data of orthologous genes in birds and other vertebrates with varying degree of sex- and autosomal linkage and phylogenetic distance to the great reed warbler. From Ensembl BioMart (Smedley et al. 2015), we downloaded d*N*/d*S* values from orthologs to sex-linked great reed warbler genes for one species-pair each of birds (zebra finch and chicken, *Gallus gallus*), reptiles (green anole and bearded dragon, *Pogona vitticeps*), mammals (human, *Homo sapiens*, and house mouse, *Mus musculus*), and fish (three-spined stickleback, *Gasterosteus aculeatus*, and fugu, *Takifugu rubripes*), respectively (supplementary table 14, Supplementary Material online). Next, we correlated these d*N*/d*S* values to each other as well as to the d*N*/d*S* values for great reed warbler gametologous gene pairs (great reed warbler Z vs. great reed warbler W) and for each great reed warbler gametolog to the zebra finch ortholog (great reed warbler Z vs. zebra finch; great reed warbler W vs. zebra finch). Interestingly, these pairwise analyses of d*N*/d*S* values showed strong positive correlations not only for comparisons within birds (two examples of pairwise correlations are shown in fig. 6*a and b*), but also for deeply diverged groups such as birds and fish (fig. 6*c and d*). In fact, for comparisons involving orthologs to genes on the ancestral sex chromosome region, all correlations were positive and all except one significantly so (Spearman correlation: *P* values < 0.05; exception: great reed warbler Z vs. great reed warbler W compared with stickleback vs. fugu; *P *=* *0.051; fig. 6*c*), and for comparisons involving orthologs on the added region all correlations were positive and significant (fig. 6*d*;*P* values < 0.05; scatter plots and *P* values for all correlations are provided as supplementary figs. 9 and 10, Supplementary Material online).

## Discussion

Fusions between autosomes and sex chromosomes are rare in birds and have so far been reported only among Sylvioidea species (Pala et al. 2012; Leroy et al. 2019; Sigeman et al. 2019, 2020; Dierickx et al. 2020) and three other lineages (Gan et al. 2019; Kretschmer et al. 2020; Huang et al. 2021). Our annotated genome assembly and detailed characterization of the Z and W chromosomes of one Sylvioidea species, the great reed warbler, provide strong evidence for translocation of a part of chromosome 4A to the ancestral sex chromosome through fusion events on both the Z and the W chromosomes. The Z fusion is covered by a single scaffold (Scaffold31), and we observe a local accumulation of repeats (mainly LTRs) at the fusion point which may have facilitated the translocation. Synteny between the translocated, added-Z chromosome region and the corresponding region on chromosome 4A in other passerines further revealed complete collinearity and maintained size (9.6 Mb), showing that no intra-chromosomal rearrangements involving this region have occurred since the fusion event. In contrast, the W chromosome, which to a large extent is covered by two sizable “superscaffolds,” is more dynamic with multiple large- and small-scaled rearrangements. Several W scaffolds contain sequences from both the ancestral and the added part, which provides strong evidence for a W fusion followed by intrachromosomal rearrangements. A fusion event on Sylvioidea W is further supported by karyotype data in larks, family Alaudidae (Bulatova 1981; see Sigeman et al. 2019), and by sequence data in white eyes, family Zosteropidae (Leroy et al. 2019).

The W chromosome is one of the most difficult regions in the genome to assemble due to its repetitive and haploid nature (Tomaszkiewicz et al. 2017). Karyotype data have shown that the size of the W chromosome in birds varies even over short timescales (Rutkowska et al. 2012), although much of the variation in size of W assemblies can also be attributed to differences in sequencing technology, with short-read sequencing failing to scaffold repetitive regions and therefore underestimating the actual size of W (Smeds et al. 2015; Xu et al. 2019). By using long-read sequencing and optical maps, we managed to assemble a total of 30.2 Mb of the W chromosome, of which 12.3 Mb could be traced back to the ancestral and 10.3 Mb to the added part. This suggests that our W assembly (considering both placed and unplaced W-linked scaffolds) approaches the size (∼21 Mb) of the latest version of the zebra finch W chromosome, which does not have an added-W (NCBI Annotation Release 105: bTaeGut2.pat.W.v2; Rhie et al. 2021). Despite these difficulties associated with assembling W chromosomes, it seems clear that the ancestral-W is much smaller than the ancestral-Z chromosome (∼80 Mb) in most birds, including the great reed warbler. In contrast, and despite a few Mb-large deletions of gene poor regions, our assembly of the added-W region (10.3 Mb) is longer than the added-Z region (9.6 Mb), which is likely the result of the observed repeat accumulation (mainly LTRs). A relative increase in size of the sex-limited chromosome compared with its chromosome copy has also been observed in the *Drosophila miranda* XY system (Bachtrog et al. 2019).

In present day great reed warblers, recombination is suppressed across the entire added sex chromosome region, meaning that recombination stopped between the fusion event and today. We show that the added region continued to recombine for several million years after the fusion event and thus acted as a second PAR in the ancestors of the great reed warbler. The relative importance of large- and small-scale rearrangement events (e.g., large inversions vs. smaller mutations) as well as gradual versus discontinuous changes during the evolution of recombination suppression has been debated (Charlesworth et al. 2005; Bergero and Charlesworth 2009; Ponnikas et al. 2018; Yazdi and Ellegren 2018). Up until recently, most evidence of large inversions causing recombination cessation had come from old and heavily differentiated sex chromosome systems such as from birds and mammals. Empirical data from more recently formed sex chromosome systems, however, has shown that both inversions and gradual events can lead to recombination suppression (reviewed in Wright et al. [2016] and Ponnikas et al. [2018]). The detected rearrangements in the added-W region in our data (there were no signs of rearrangements on the added-Z chromosome) may have contributed mechanistically to recombination suppression. However, rearrangements are also more likely to become fixed in regions of already low recombination, making the distinction between cause and consequence of suppression extremely difficult. Our phylogenetic analysis of gametologous gene pairs strongly suggest that recombination suppression in the great reed warbler lineage has progressed in a nonlinear, mosaic, and relatively small-scaled manner along the added part of the neo-sex chromosome. This does not follow an expected pattern of a single large-scale rearrangement or a linear progression of recombination suppression starting from the fusion point. Instead, it is in line with a local recombination suppression process, possibly mechanistically driven by a combination of relatively small inversions, repeat accumulation, and heterochromatinization (cf. Ponnikas et al. 2018). This mosaic pattern makes it difficult to define evolutionary strata on the added region of the neo-sex chromosome, which contrasts the situation on the ancestral avian sex chromosomes (Zhou et al. 2014; Xu et al. 2019). An evaluation of recombination suppression timings based on d*S* values between gametologous gene pairs is also in line with these conclusions.

As expected, we observe clear consequences of sex-linkage and recombination suppression on the Sylvioidea neo-sex chromosomes, in particular for the sex-limited copy (W) which does not recombine in either sex. Both the ancestral and added-W chromosome regions showed pronounced accumulation of repeat elements (68.1% and 35.8%, respectively; mainly LTRs) and low nucleotide diversity (0.0002 and 0.0001, respectively). The repeat content of the great reed warbler ancestral-W scaffolds was higher than in the short-read sequenced collared flycatcher W chromosome (FicAlb1.5: 49%; Smeds et al. 2015) and slightly lower than the W chromosome in the long-read sequenced chicken (galGal5: ∼68%; Kapusta & Suh 2017) and paradise crow (*Lycocorax pyrrhopterus*; ∼70%; Peona et al. 2021) genomes. The Z chromosome had similar repeat levels as autosomes, but we found support for reduced nucleotide diversity on the ancestral-Z (0.0007) compared with the genome-wide average (0.0032), and on the added-Z (0.0044) compared with the autosomal part of chromosome 4A (0.0069). In addition to supporting consequences of sex-linkage also on avian Z chromosomes, this result highlights the importance of comparing chromosomes with similar properties (e.g., size and gene density) in intra-specific analyses, and ideally homologous chromosomes in inter-specific analyses, when evaluating the genomic consequences of sex-linkage (Julien et al. 2012). Lower nucleotide diversity on sex chromosomes has been observed in many lineages (Wilson Sayres 2018), but the relative importance of effective population size effects and selection is poorly understood. In addition to this, the female-specific inheritance of the W chromosome is also expected to contribute to lower nucleotide diversity than on the Z chromosome and autosomes, as birds like, for example, mammals have male-biased mutation rates (Ellegren and Fridolfsson 1997). Regardless of the cause, the extremely low nucleotide diversity on the ancestral- as well as added-W chromosome regions likely diminishes the evolutionary potential of the neo-sex chromosome W copy.

Instead, W-linked genes are likely to either become lost by degeneration and drift, or be preserved through purifying selection. Our analyses of synonymous and nonsynonymous substitutions, and haploinsufficiency, strongly suggest that the W chromosome is enriched for dose-sensitive and conserved genes that are being maintained by purifying selection. The d*N*/d*S* ratio between gametologs was low for the added region and even more so for the ancestral region, which can be explained by particularly strong purifying selection on the few surviving genes on the ancestral W chromosome region. Together, these results suggest that W gametologs with conserved functions are being maintained functionally by purifying selection over long evolutionary timescales, and that the new set of sex-linked genes on the added part of the Sylvioidea neo-sex chromosomes mimics the ancestral avian sex chromosome at an earlier stage of its evolution. Our set of 41 gametologous gene pairs on the ancestral W region was highly overlapping with the gene set found in the flycatcher W chromosome (i.e., entirely ancestral; Smeds et al. 2015), which shows that the ancestral W chromosome in the great reed warbler has not undergone more pronounced degeneration compared with other songbirds. We also observe that Z-linked genes with a remaining copy on the W chromosome are more conserved and dose sensitive than genes whose copy has been lost from the W chromosome, a pattern that is concordant with studies of ZW-gene pairs in chicken (Bellott et al. 2017). In general, although degeneration seems a likely long-term evolutionary trajectory for most W-linked genes, apparently some W genes are maintained for long periods of time by purifying selection. We did not find any genes on the added-W without gametologs on the added-Z. This contrasts with, for example, the situation in some mammals, where a few genes have been added to the Y chromosome (Cortez et al. 2014) and in *Drosophila miranda*, where intense gene translocation to the Y chromosome has occurred (Bachtrog et al. 2019).

Strong purifying selection on sex-linked genes are thought to drive patterns of convergent sex chromosome evolution across different taxonomic groups with independently evolved sex chromosome systems, such as birds (ZW) and mammals (XY) (Bellott et al. 2017). Our results extend these conclusions by showing that gene rate evolution (d*N*/d*S*) is strongly positively correlated between widely diverged taxonomic groups, regardless of whether the genes are autosomal or sex-linked, Z- or W-linked, or located on newer or older parts of the sex chromosome. For example, we find highly significant correlations between d*N*/d*S* values for a set of great reed warbler Z and W gametologs, and d*N*/d*S* for orthologs to these genes in two lizard species. The majority (99.8%) of d*N*/d*S* values are <1, and all pairwise correlations are positive and significant (except for one: *P *=* *0.051). This strongly suggests that these broad taxonomic trends are driven by different strenghts of purifying selection acting on genes with more or less conserved essential functions on a deep phylogenetic level. However, we cannot exclude the action of correlated positive selection in shaping these trends although we believe it has a minor influence, especially for W-linked genes. We conclude that the highly predictable evolutionary trajectory of sex-linked genes in both birds and mammals (cf. Bellott et al. 2017) is driven partly by sex-linkage per se (e.g., due to small effective population size and inefficient selection), partly by different degrees of functional conservation of specific genes.

## Materials and Methods

### Extraction and Library Preparation

High-molecular weight DNA from blood, and total RNA from liver, heart, and muscle tissue, from a juvenile female great reed warbler was extracted and sequenced with the following technologies: PacBio RSII (DNA), Illumina HiSeq X (DNA), chromium linked-read sequencing (10× Genomics; DNA), Bionano (DNA), Illumina HiSeq 2500 (RNA), and PacBio Iso-Seq (RNA). Information on extraction protocols is found in supplementary text 1.1, Supplementary Material online and details on sequencing technologies in supplementary table 1*a*, Supplementary Material online.

### Assembly Strategy

PacBio subreads longer than 500 bp and with quality (QV) > 80 were de novo assembled using FALCON v0.5.0 (Chin et al. 2016), using a pre-assembly cutoff of 8 kb. The draft assembly was error corrected twice using the PacBio reads and once using Illumina-sequenced paired-end data from the same individual. The draft assembly was split at putative misassembly sites and scaffolded using chromium linked-read data. We then identified and split scaffolds at putative misassembly sites based on synteny to the zebra finch genome, and performed a second round of scaffolding using the linked-read data. Bionano optical mapping data (using two enzymes: BSPQI and BSSSI) were de novo-assembled and used to anchor the scaffolds in the PacBio-based genome assembly. We filled in gaps in the draft assembly, using both short reads and PacBio long reads, followed by two more rounds of polishing. All scaffolds shorter than 1,000 bp were removed from the assembly. We identified and split seven scaffolds manually at misassembled sites. Lastly, we removed redundant scaffolds that represent haplotypes of another scaffold (“haplotigs”) by using the purge haplotigs pipeline (Roach et al., 2018). Details on all steps in the assembly strategy are found in supplementary text 1.2, Supplementary Material online and information on the data used in supplementary table 1*a*, Supplementary Material online.

To produce the circos plot (fig. 1), we ran SatsumaSynteny v2.0 (Grabherr et al. 2010) between the great reed warbler genome and the great tit genome (Parus_major1.1; GCA_001522545.2; Laine et al. 2016). We calculated the length (bp) of all matches between great reed warbler scaffolds and great tit chromosomes. Scaffolds that matched with more than 100 kb and more than 1% of its length to any great tit chromosome were kept for visualization. This left 123 scaffolds with a total length of 1.10 Gb. We used the tool bundlelinks (from circos-tools-0.23; Krzywinski et al. 2009) and settings -max_gap 100000 -strict -links to produce larger ranges of matches between the species. Lastly, we removed scaffolds that had been identified as W-linked (see above), leaving 100 scaffolds with a total length of 1.07 Gb. Alignments between great reed warbler scaffolds and great tit chromosomes were plotted in circos v.0.69-8 (Krzywinski et al. 2009).

### Repeat and Gene Annotation

We de novo predicted repeats in the assembly using RepeatModeler v.1.0.8 (Smit and Hubley 2008–2015) with the option -engine ncbi. We then ran RepeatMasker v4.0.7 (Smit et al. 2013–2015) with the output from RepeatModeler and a custom library with manually curated repeat elements from different bird genomes provided by Alexander Suh (Uppsala University; fAlb15_rm3.0_aves_hc.lib) with options -a -xsmall -gccalc. Gene models were predicted with MAKER v.3.00.0 (Holt and Yandell 2011; Campbell et al. 2014), using 1) only extrinsic evidence (proteins and transcripts), and 2) combining the gene builds from extrinsic evidence sequences with ab-initio predictions in Augustus v.3.2.3 (Stanke et al. 2006). Details on the gene builds are provided in supplementary text 1.3.2, Supplementary Material online.

We inferred the function of genes and transcripts using the translated CDS features of each coding transcript by running InterProScan v-5.7-48 (Jones et al. 2014). Gene names were inferred by blasting the same sequences against the manually curated Uniprot/Swissprot database (supplementary text 1.3.3, Supplementary Material online). We predicted tRNAs using tRNAscan v.1.3.1 (Lowe and Eddy 1997) (450 tRNAs) and other ncRNAs using the RNA family database Rfam v.11 (Nawrocki et al. 2015). A lift-over annotation to the great reed warbler genome was done using the 1) zebra finch (Taeniopygia_guttata.taeGut3.2.4.94; 17,487 genes) and 2) chicken (Gallus_gallus.Gallus_gallus-5.0.94; 18,345 genes) Ensemble gene annotations. OrthoMCL v2.0.9 (Li et al. 2003) with protein sequences from seven bird and mammal species was used to group orthologous protein sequences. In total, 7,623 ortholog groups (OG) were common to all species, and 374 OG specific to the great reed warbler. Details on all steps in the gene annotation strategy are found in supplementary text 1.3, Supplementary Material online.

### Sex Chromosome Analyses

#### Identifying Sex-Linked Scaffolds

To identify sex-linked scaffolds, we aligned whole-genome sequence data from five female and five male great reed warbler individuals to the reference genome (supplementary table 1*b* and supplementary text 2.1, Supplementary Material online). We followed the general method from Smeds et al. (2015) for identifying W-linked scaffolds by first parsing the alignment files for reads with any mismatching base pairs (bam file tag NM: i : 0) and then search for scaffolds where the median female coverage (across samples) was >25× whereas the median male coverage was zero. This resulted in 50 W-linked scaffolds of which 15 were represented in the gene annotation and were designated as “W-scaffolds.” The 35 scaffolds which were not present in the annotation file were grouped as “random W-scaffolds” (supplementary tables 6 and 7, Supplementary Material online).

To identify Z-linked scaffolds, we utilized the difference between the median coverage values for males and females (following the same method as above) but also the difference in heterozygosity. As females are haploid for Z-linked scaffolds whereas males are diploid, we expect them to differ in this measurement. We classified Z-linked scaffolds based on two criteria; 1) either the median coverage in females was less than 55% of the male coverage, or 2) the median female coverage was less than 65% and the heterozygosity values for males and females had an absolute difference of more than 0.1 (supplementary text 2.1, Supplementary Material online). Using this method, 22 scaffolds were classified as Z-linked, of which 8 were represented in the gene annotation file. Same as with the W-linked scaffolds, we designated these eight as “Z-linked scaffolds” and the other ones as “random Z-linked scaffolds” (supplementary tables 6 and 7, Supplementary Material online). A linkage map analysis using a pedigree of 511 great reed warblers assigned seven of these eight Z-linked scaffolds to the same linkage group, and identified an additional Z-linked scaffold: Scaffold92 (Ponnikas et al. 2020). Six of these sex-linked scaffolds (supplementary table 7, Supplementary Material online) could be anchored (i.e., ordered and oriented) successfully in the Z linkage group. Lastly, Scaffold217 was identified as the PAR according to the linkage map. This scaffold is 0.9 Mb in length, contains the PAR genes identified in other songbird species and had equal coverage values between the female and male great reed warblers (Ponnikas et al. 2020). Female and male genome coverage values, and heterozygosity values, are shown in supplementary figure 2, Supplementary Material online.

#### Chromosome Matches of Sex-Linked Scaffolds

For comparisons between the ancestral and the added sex chromosome region on a DNA level, we classified the sex-linked scaffolds that were represented in the gene annotation as either ancestral or added using whole-genome alignments to the zebra finch and the collared flycatcher. In these species, the added sex-linked region corresponds to chromosome 4A and the ancestral sex chromosome to chromosome Z. To get reliable genomic positions for the great reed warbler sex-linked scaffolds, we used the following method: We extracted all genomic ranges where a sex-linked scaffold (containing at least one gene in the annotation) had aligned to both the zebra finch and flycatcher genome. Among these ranges, we considered a genomic region as belonging to the ancestral sex chromosome (Z) if the same range aligned to chromosome Z (or Z_random) in both the zebra finch and flycatcher. In addition, genomic ranges that aligned in one species to chromosome Z (or Z_random) and an unplaced scaffold in the other species was accepted. The same was done for the added sex chromosome region (i.e., alignments to chromosome 4A or 4A_random). Genomic ranges shorter than 10 kb were ignored. This resulted in genomic ranges across eight Z-linked scaffolds with a combined length of 87.2 Mb, and 11 W-linked scaffolds with a combined length of 22.4 Mb (supplementary table 6*a and b*, Supplementary Material online).

To verify the correctness of some W-linked scaffolds, the genome of another great reed warbler female was assembled with chromium linked-read data sequenced from blood-extracted DNA (supplementary table 1*b*, Supplementary Material online) using supernova run 2.1.0 (Weisenfeld et al. 2017). Genome assembly statistics are provided in supplementary tables 2 and 3, Supplementary Material online.

#### Gametologs and Manual Curations of Sex-Linked Genes

The different gene builds generated in MAKER were imported into WebApollo (Lee et al. 2013) where we manually curated gametologous (ZW) gene pairs on both the ancestral and added region, and all additional genes on added Z. To identify gametologs, we used evidence from the OrthoMCL analysis, along with the lift-over annotations and gene order information from chicken and zebra finch (supplementary text 2.2, Supplementary Material online). We identified and curated 42 gametologous gene pairs from the ancestral sex chromosome. From the added sex chromosome region, we identified and curated 137 genes on Z-linked scaffolds of which 111 were found also on W-linked scaffolds. We also identified 277 genes from the ancestral Z without a W-copy by using single-copy orthologs across eight bird species and one lizard species. We then intersected these genes with genes that grouped with a single great reed warbler transcript in the ortholog analysis, and corresponded to zebra finch transcripts located on either the Z chromosome or Z_random.

We extracted and aligned the transcripts from these sex-linked genes (manually curated ZW gene pairs, *n *=* *153; curated Z genes without W gene copies, *n *=* *22; and uncurated Z genes without W gene copies, *n *=* *277) from the great reed warbler gene annotation together with the corresponding zebra finch transcript for each gene (supplementary text 2.2, Supplementary Material online). We calculated pairwise substitution rates between the three sequences (great reed warbler Z and W, and zebra finch) per gene using codeml from the PAML package v4.9 (Yang 2007). After filtering for a minimum length of 500 bp and d*S* < 3, 79 added sex chromosome gene pairs, and 18 added Z genes without a W-copy remained. On the ancestral sex chromosome, 35 gene pairs remained after filtering. Of the uncurated ancestral Z-linked genes without a W-copy, 238 remained after filtering.

#### Gametolog Extraction and Recombination Suppression Analysis

We in silico extracted Z- and W-sequences for each CDS of all gametologous gene pairs (*n *=* *153) from a male and a female of six Sylvioidea species (great reed warbler; clamorous reed warbler, *Acrocephalus stentoreus*; marsh warbler, *Acrocephalus palustris*; western olivaceous warbler, *Iduna opaca*; Savi’s warbler, *Locustella luscinioides*; and bearded reedling, *Panurus biarmicus*; supplementary table 1*b*, Supplementary Material online) based on aligned reads to a version of the great reed warbler reference genome from which all W-linked scaffolds (*n *=* *50) had been removed prior to mapping. The reason behind this approach was to avoid unequal mapping success to the Z- versus W-scaffolds due to recombination suppression being younger than the speciation dates. Based on SNP distributions between the sexes (conditions for phasing are in supplementary table 15, Supplementary Material online, and general methodology is described in Sigeman et al. 2018), we phased CDS sequences into a Z and W copy. SNPs with quality scores or genome coverage values lower than 20 were replaced with “N.” The sequences belonging to each CDS was aligned using PRANK v.170427 (Löytynoja 2014) and then concatenated in the right order using the program catfasta2phyml (https://github.com/nylander/catfasta2phyml/, last accessed September 24, 2021). We calculated per-exon genome coverage (using bamstat04; Lindenbaum 2015) and number of private alleles (using vcftools v.0.1.15; Danecek et al. 2011; option “–singletons”) for each sex and species. We removed genes where any of the sequences from Sylvioidea species showed signs of deletions of parts or the entire W-sequence, identified using the combined genome coverage and private allele data.

Then, we added one-to-one orthologs from outgroup species to these alignments using MAFFT v.7.407 (options –reorder –add –genafpair –adjustdirectionaccurately –maxiterate 1000 –nuc). For the ancestral sex chromosome region, we added orthologs from the following seven species: two non-Sylvioidea oscine passerines (great tit, *Parus major*, and zebra finch, *Taeniopygia guttata*), one suboscine passerine (blue-crowned manakin, *Lepidothrix coronata*), budgerigar (*Melopsittacus undulatus*), one Galloanserae (chicken, *Gallus gallus*), one Palaeognathae (emu, *Dromaius novaehollandiae*), and green anole (*Anolis carolinensis*). We only retained genes where all outgroups were present in the alignments. From the added sex chromosome region, we added orthologs from great tit and zebra finch, but retained only the outgroup species resulting in the longest final alignment after trimming (see below). The ortholog information was downloaded from BioMart along with the CDS sequences from these genes. All alignments were manually inspected and trimmed for poorly aligned regions in Geneious v.11.1.5 (https://www.geneious.com, last accessed September 24, 2021), and all sites with ambiguous nucleotides in the sequences (N) or gaps (-) were removed using Gblocks v.0.91b (Castresana 2000). We used the program GENECONV v. 1.81a (Sawyer 1999) to identify, and then remove, all sites that were flagged as potential gene conversion blocks between Z- and W-sequences within Sylvioidea species (options:/w123/lp).

After removing short alignments (<700 bp), 20 genes from the ancestral sex chromosome and 64 genes from the added sex chromosome region remained (note that these 64 genes are autosomal in all non-Sylvioidea species). We built phylogenetic trees using the maximum-likelihood algorithm in RAxML v.8.2.12 (raxmlHPC; Stamatakis 2014) using the following options: -m GTRGAMMAX -f a -N autoMRE. We evaluated the topology of these gene trees (supplementary trees, Supplementary Material online, only considering nodes with bootstrap support ≥70) to estimate the timing of recombination suppression. Specifically, on the added sex chromosome region we evaluated if the W-sequences from the different species clustered together with other W-sequences, or with their respective Z-linked gametolog. On the added sex chromosome region, we considered gametologs grouping together by species to have become recombination suppressed on their species-specific branch, whereas W-sequences from different species that significantly grouped together were considered to have become recombination suppressed on the branch leading up to these species. On the ancestral sex chromosome region, where all Sylvioidea W-sequences clustered together in all gene trees (and where the non-Sylvioidea species only have Z sequences), we estimated timing of recombination suppression based on the closest significantly supported branches flanking either side of the Sylvioidea W sequence cluster.

Finally, we constructed a dated phylogeny of all species used for this analysis (*n* = 13) based on 100 autosomal genes, as well as a dated phylogeny and calibration times from previous studies. For details, see supplementary text, Supplementary Material online.

#### Population Genomics

We used freebayes v.1.1.0 (Garrison and Marth 2012) to call genotypes in the resequenced five male and five female great reed warblers mentioned above (supplementary table 1*b*, Supplementary Material online). For the females, we called genotypes separately on sex-linked scaffolds by using the “–haploid” flag. The genotype data were filtered using a combination of vcftools and vcflib (https://github.com/vcflib/vcflib, last accessed September 24, 2021). First, the raw set of variants was filtered for overlap with annotated repeats. We further filtered variants that had mean site coverage of at least twice the median mean coverage across all sites. For females, this value was calculated separately for autosomal and sex-linked scaffolds. Next, we filtered for sites with a quality score larger than 20 (Q20), for alleles that were supported by at least one read on each strand (SAF > 0 and SAR > 0) and by at least one read centered to the left and right side of the variant (RPL > 0 and RPR > 0). We further removed male genotypes and female genotypes on autosomes that had a coverage less than 10×. For sex-linked scaffolds in females, we set the corresponding coverage threshold to 5×. Following the genotype filtering, we removed any sites that had less than 80% of called genotypes. Finally, we decomposed complex variants and short haplotypes into SNPs and indels and extracted bi-allelic SNPs for downstream analyses. SNPs were intersected with different annotation features using vcfintersect from the vcflib package.

We used vcftools to calculate nucleotide diversity. We downloaded a version of the software from https://github.com/jydu/vcftools (last accessed September 24, 2021), which in contrast to the official release support haploid data for calculations of diversity. For females, the sex-linked scaffolds were analyzed using the “-haploid” flag. Nucleotide diversity was calculated per SNP and summarized across scaffolds or particular scaffold intervals. To get a more unbiased estimate of the nucleotide diversity, we also estimated the number of callable sites in the genome. For this purpose, we used samtools mpileup (Li et al. 2009) to output coverage of each sample for each site. The software was run with default settings except for including reads with a minimum mapping quality of 1 and was run separately for males and females. From the raw output, we filtered sites based on the same coverage thresholds (depending on sex and autosomal or sex-linked scaffolds) and missingness of genotypes (80% called genotypes) as employed for the genotype filtering, and further removed positions that were overlapping with annotated repeats. The callable sites were intersected with different annotation features using BEDTools version 2.17.0 (Quinlan and Hall 2010).

## Supplementary Material

Supplementary data are available at *Molecular Biology and Evolution* online.

## Supplementary Material

msab277_Supplementary_Data
