# Supplementary material for: Avian Neo-Sex Chromosomes Reveal Dynamics of Recombination Suppression and W Degeneration
Source: Mol Biol Evol. 2021 Sep 20;38(12):5275–91. doi: 10.1093/molbev/msab277 (PMC8662655; doi:10.1093/molbev/msab277)
Supplement: msab277_Supplementary_Data [file msab277_supplementary_data.zip › Supplementary Figures.pdf]

Supplementary Figures

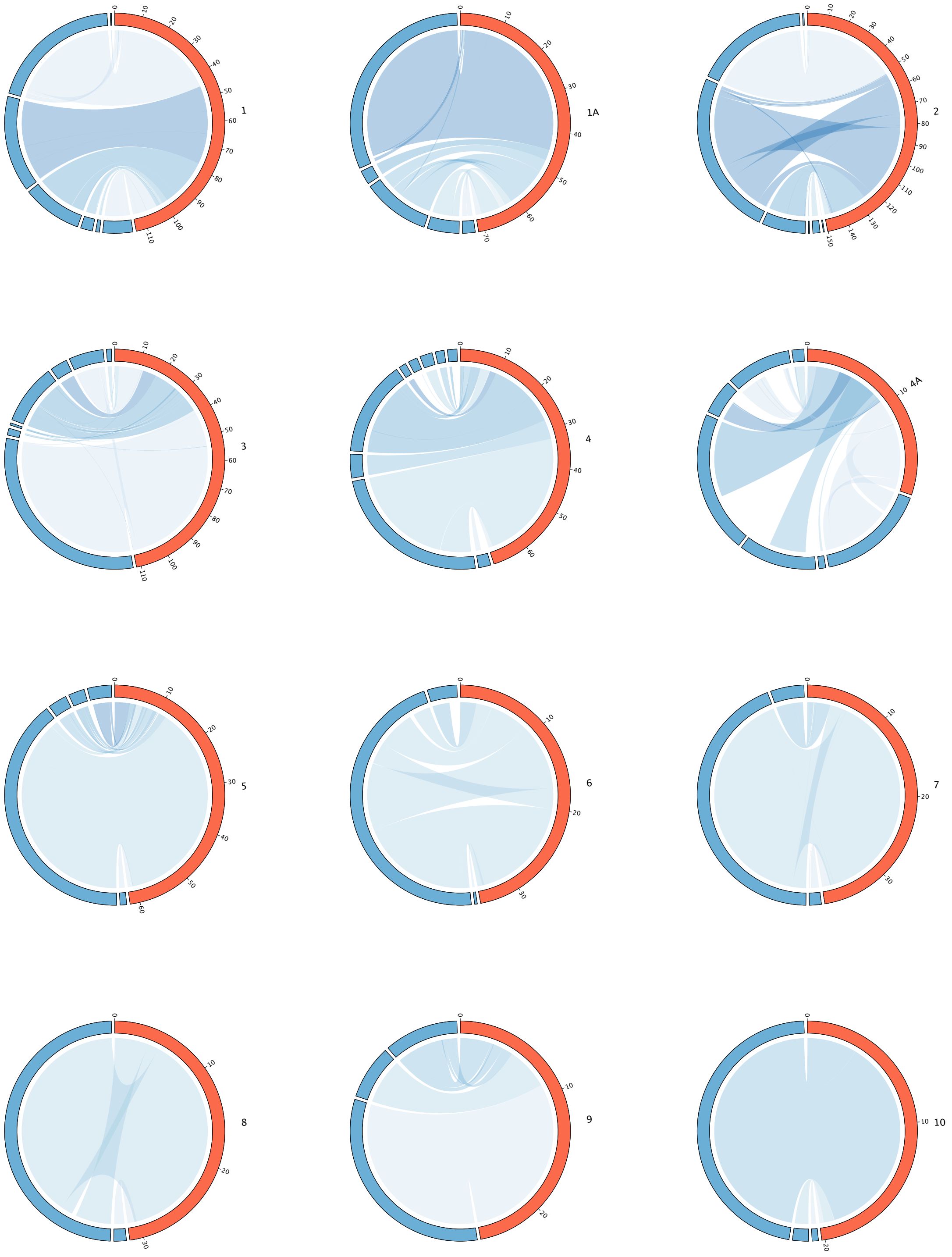

Supplementary Figure 1a. Synteny plots between great reed warbler scaffolds (blue) and great tit chromosomes (red; with chromosome names).

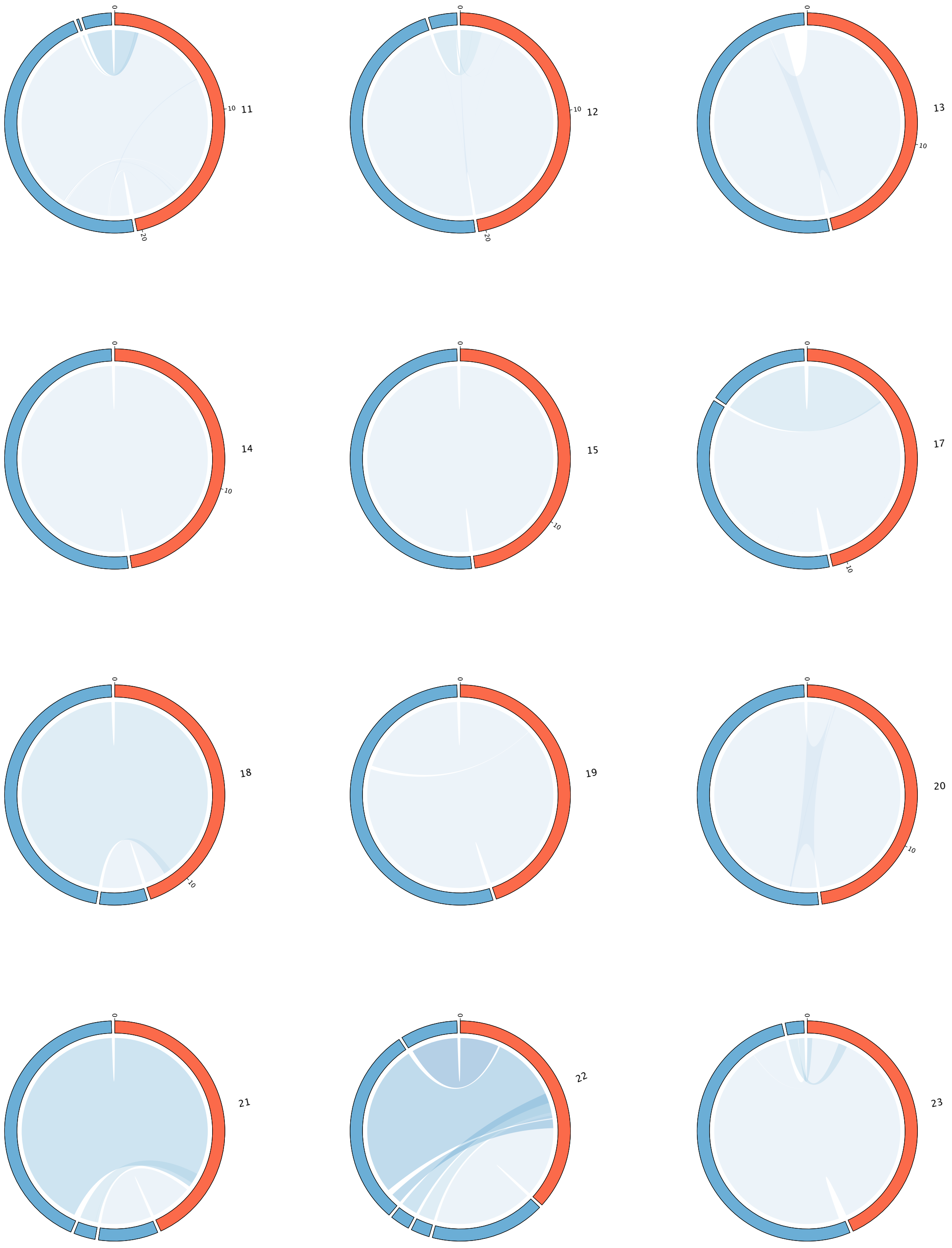

Supplementary Figure 1b. Synteny plots between great reed warbler scaffolds (blue) and great tit chromosomes (red; with chromosome names).

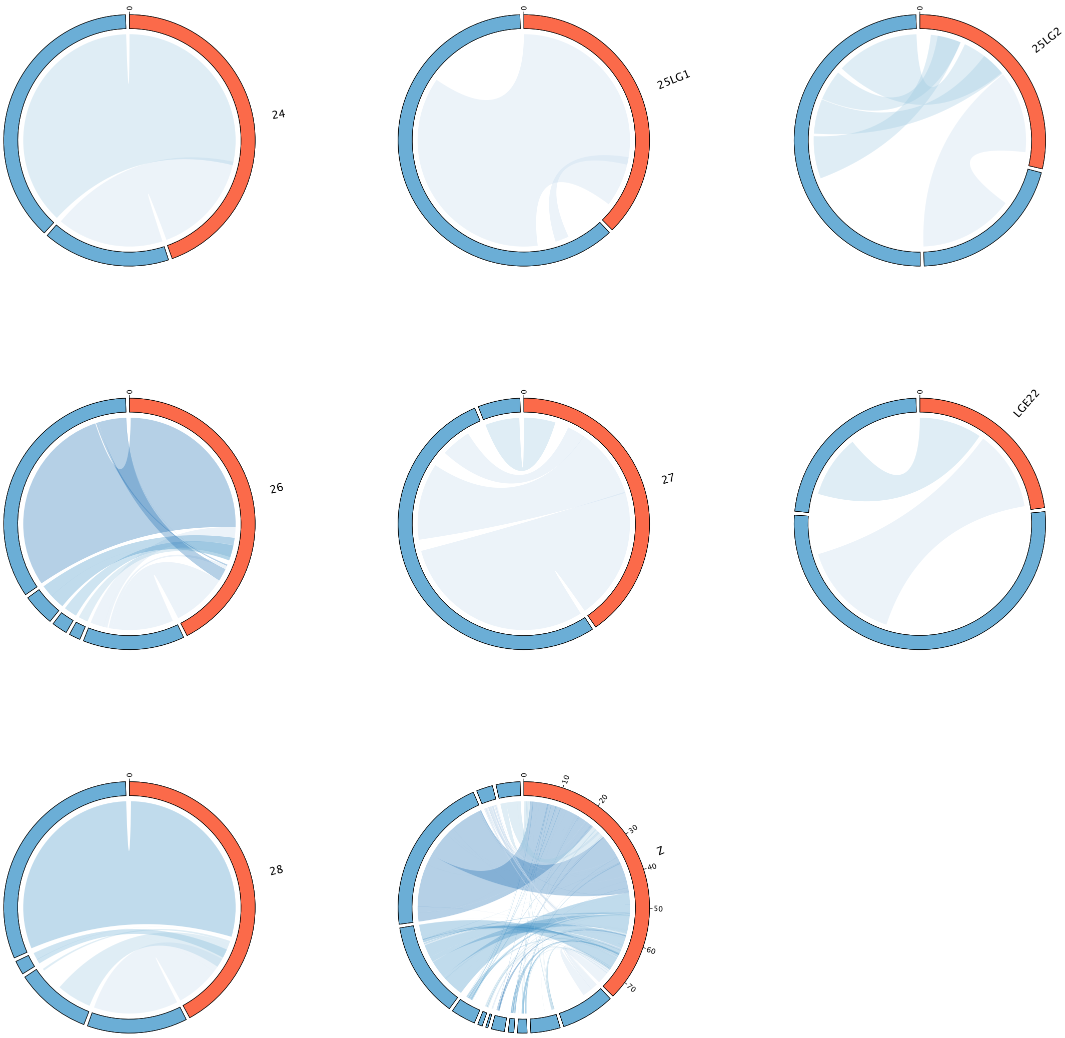

Supplementary Figure 1c. Synteny plots between great reed warbler scaffolds (blue) and great tit chromosomes (red; with chromosome names).

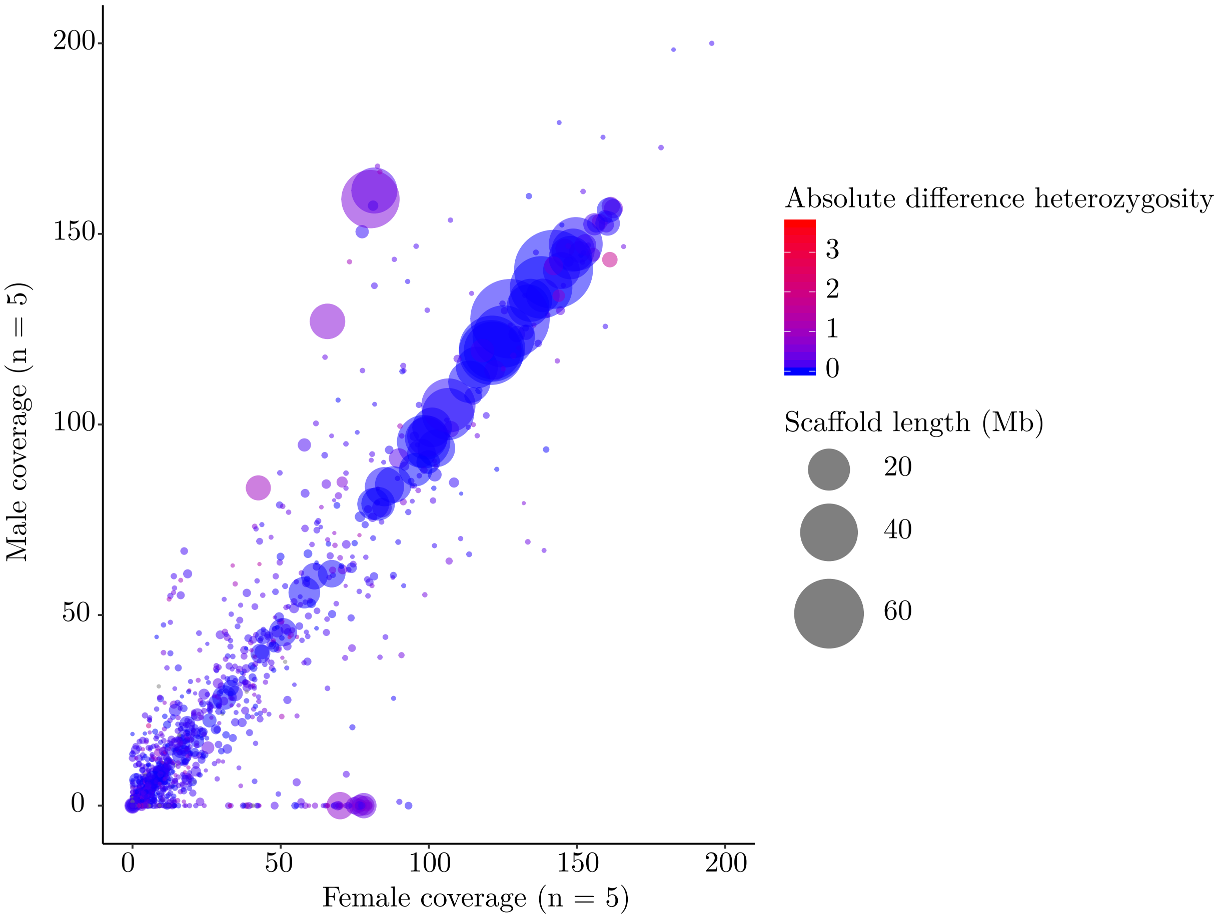

Supplementary Figure 2. Sex-specific genome coverage (x and y axis) and heterozygosity values (colour: absolute difference between sexes) for all scaffolds in the genome assembly, based on the great reed warbler individuals in Supplementary Table 1b (n = 5 females and 5 males). Point size reflect scaffold length (Mb). Z-linked scaffolds are expected to have higher coverage in males than females, and to differ in heterozygosity. W-linked scaffolds are expected to have no coverage in males.

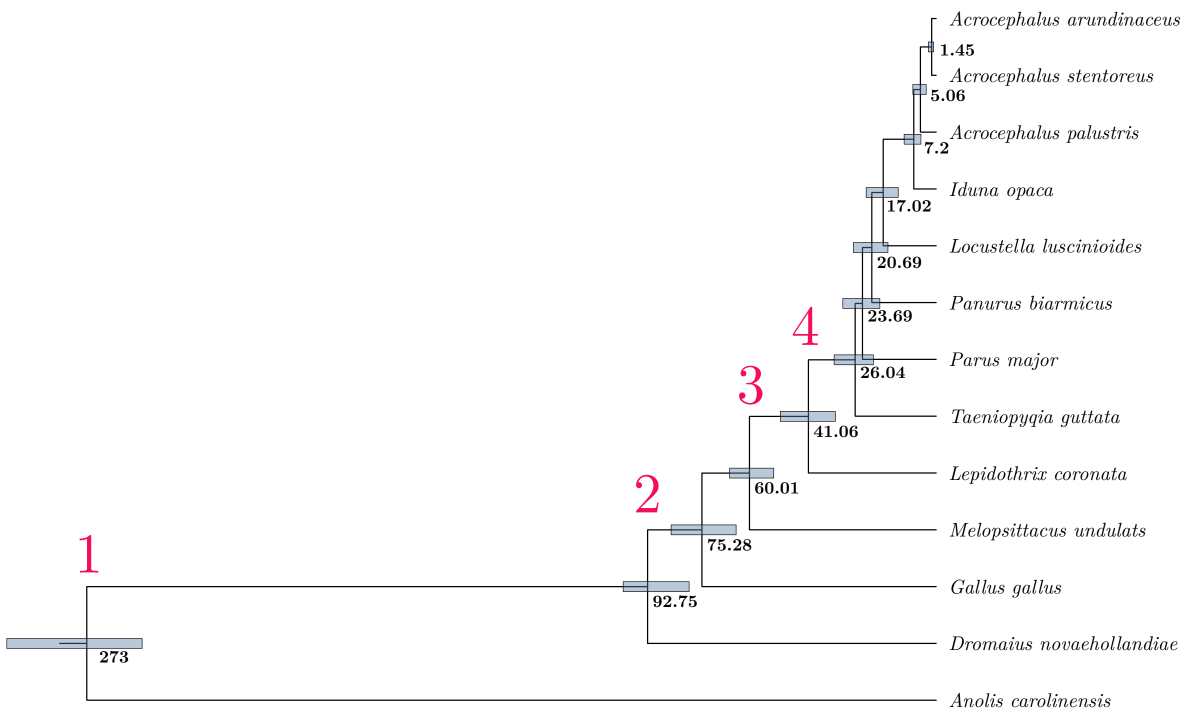

Supplementary Figure 3. Dated 13-species phylogeny reconstructed with MCMCTree. Calibrations were denoted with red numbers above nodes, including: (1) 255.9 - 299.8 Myr (Jarvis et al. 2014), (2) 66 - 99.6 Myr. (Jarvis et al. 2014), (3) 51.81 – 66.5 Myr. (Oliveros et al. 2019), (4) 27.25 – 56 Myr. (Oliveros et al. 2019).

References:

Jarvis ED, Mirarab S, Aberer AJ, Li B, Houde P, Li C, Ho SYW, Faircloth BC, Nabholz B, Howard JT, Suh A, et al. 2014. Whole-genome analyses resolve early branches in the tree of life of modern birds. *Science* 346(6215):1320-31.

Oliveros CH, Field DJ, Ksepka DT, Barker FK, Aleixo A, Andersen MJ, Alström P, Benz BW, Braun EL, Braun MJ, et al. 2019. Earth history and the passerine superradiation. *Proc. Natl. Acad. Sci. U.S.A.* 116(16):7916–7925.

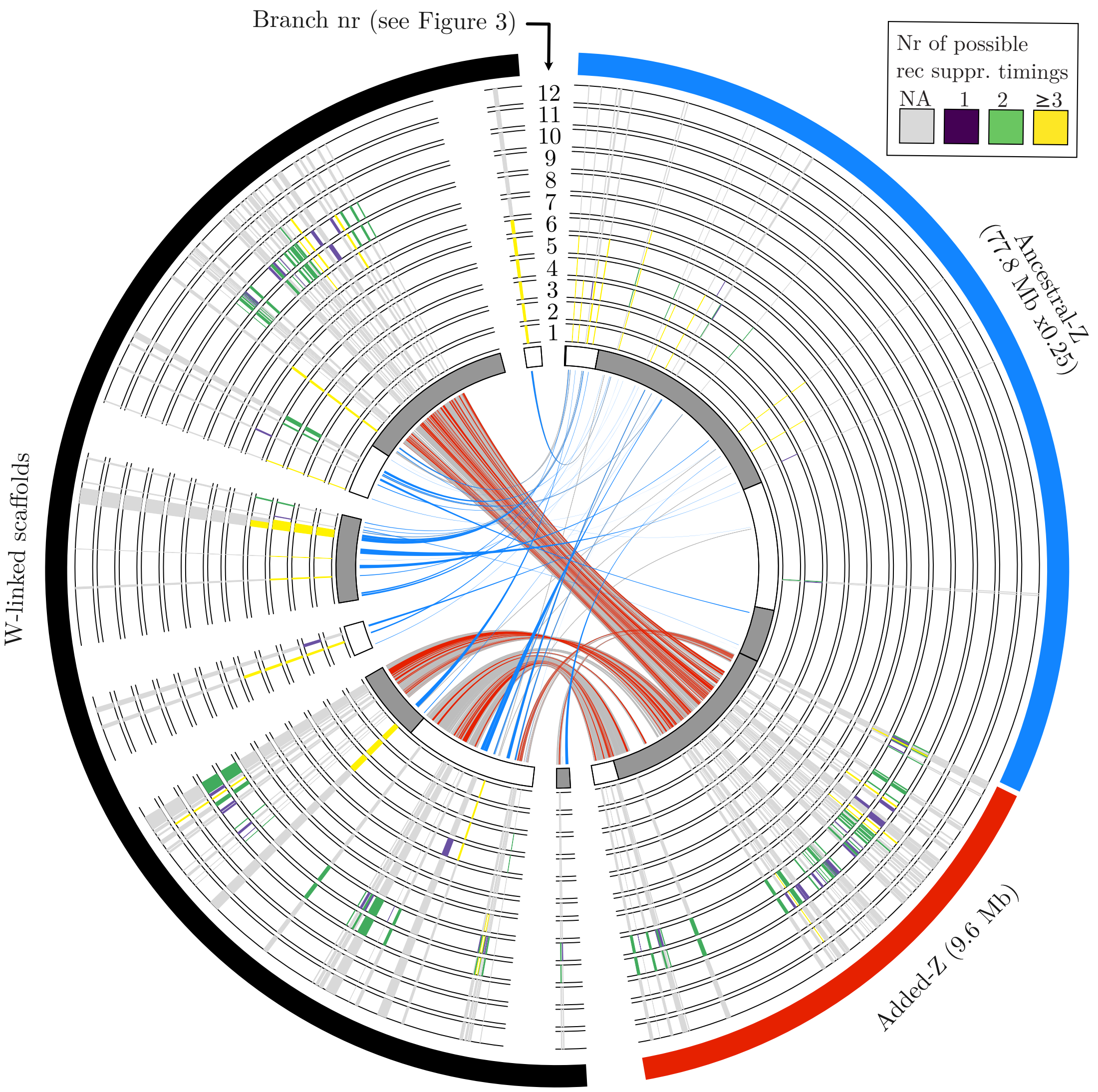

Supplementary Figure 4. Estimations of recombination suppression timings for gametologous gene pairs (corresponding to the data shown in Figure 3). The inner part of the plot (circos plot) is identical to Figure 2b, with Z-linked scaffolds on the right and W-linked scaffolds to the left. Gametologous gene pairs are plotted outside the circos plot (as heatmap values, where each heatmap row represents a branch number; see Figure 3) according to their genomic position on the W-linked and Z-linked scaffolds. The heatmap colour refers to the timing of recombination suppression given in Figure 3. Gene-specific values are provided in Supplementary Table 10. Phylogenetic trees underlying these estimates are given in Supplementary Trees.

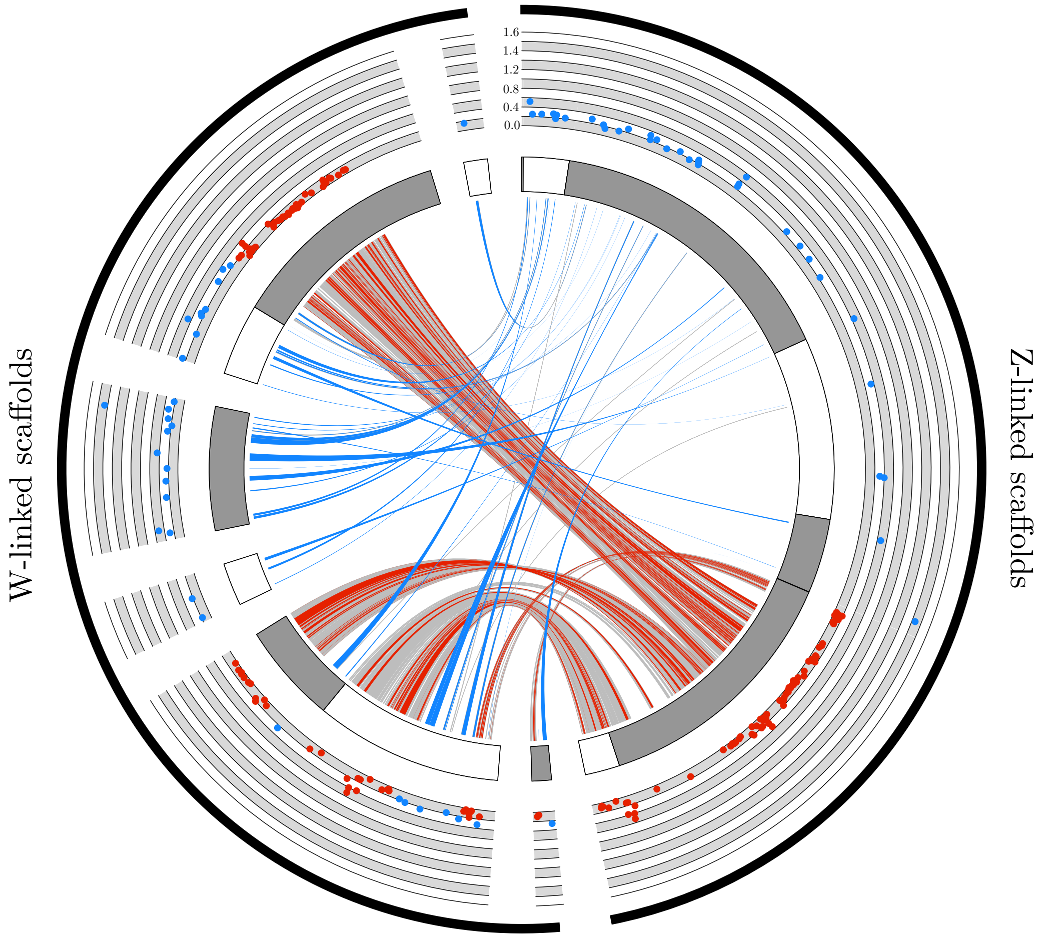

Supplementary Figure 5. dS values (between great reed warbler W and Z gametologs) plotted along the Z-linked and W-linked scaffolds (from Figure 2b). In this plot, all dS values are shown. In Supplementary Figure 8, only dS values ≤ 0.8 are shown.

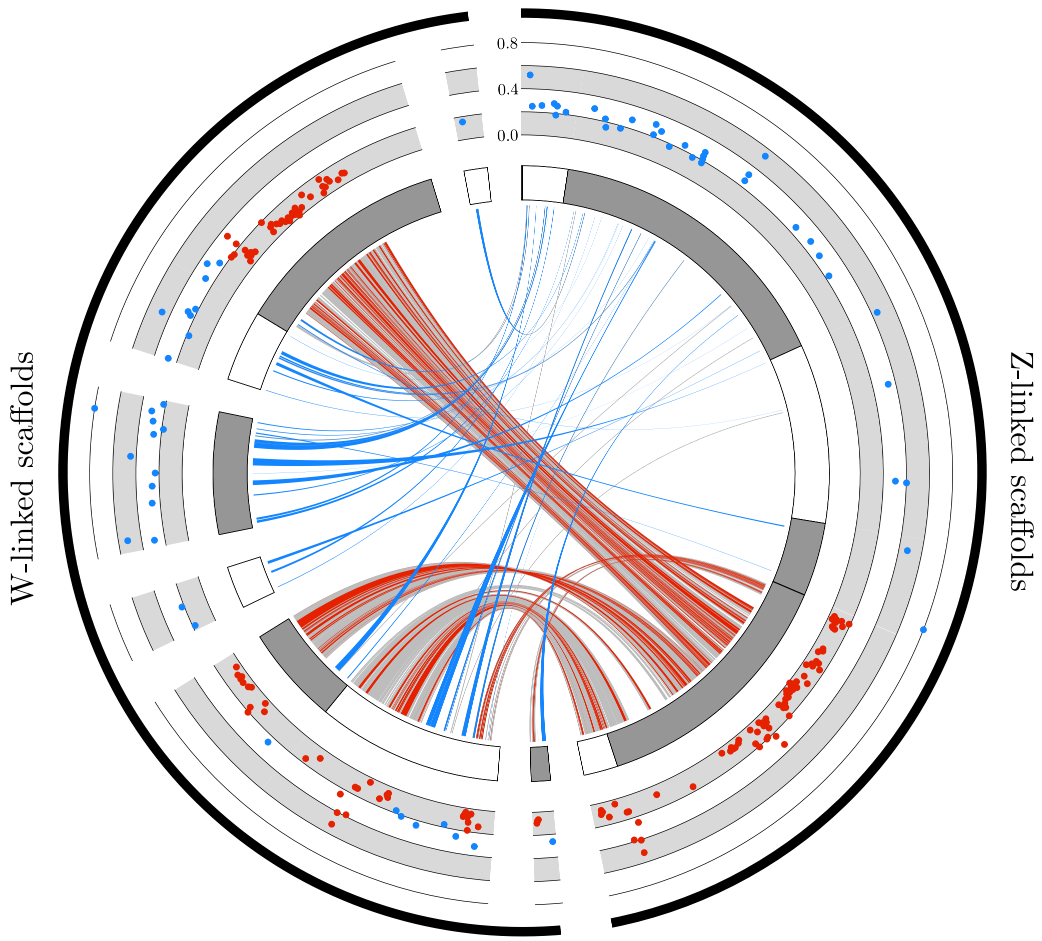

Supplementary Figure 6. dS values (between great reed warbler W and Z gametologs) plotted along the Z-linked and W-linked scaffolds (from Figure 2b). In this plot, only dS values ≤ 0.8 are shown.

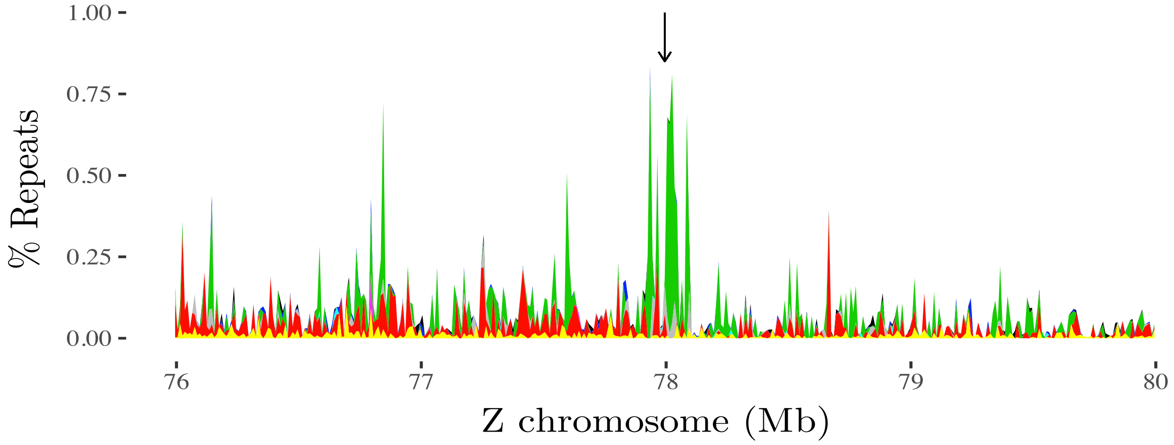

Supplementary Figure 7. Percentage of repeats around the fusion site between the ancestral and added sex chromosome (calculated using window sizes of 10kb), colored by type of repeat. The arrow marks the position of the Z to 4A fusion breakpoint.

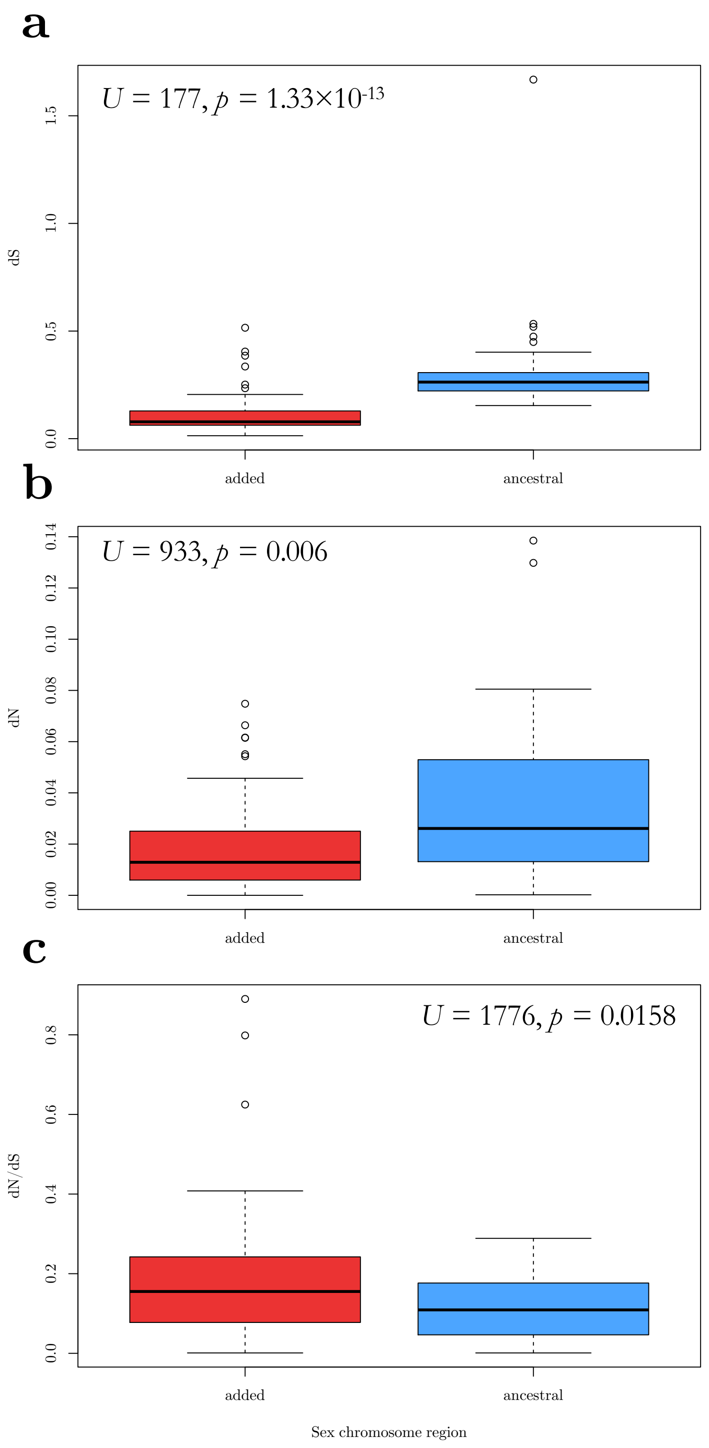

Supplementary Figure 8. Substitution rates between ZW gametologs positioned on the ancestral (blue) and added (red) sex chromosome region. (a) dS, (b) dN and (c) dN/dS. Statistics from Mann-Whitney U tests are reported for each panel.

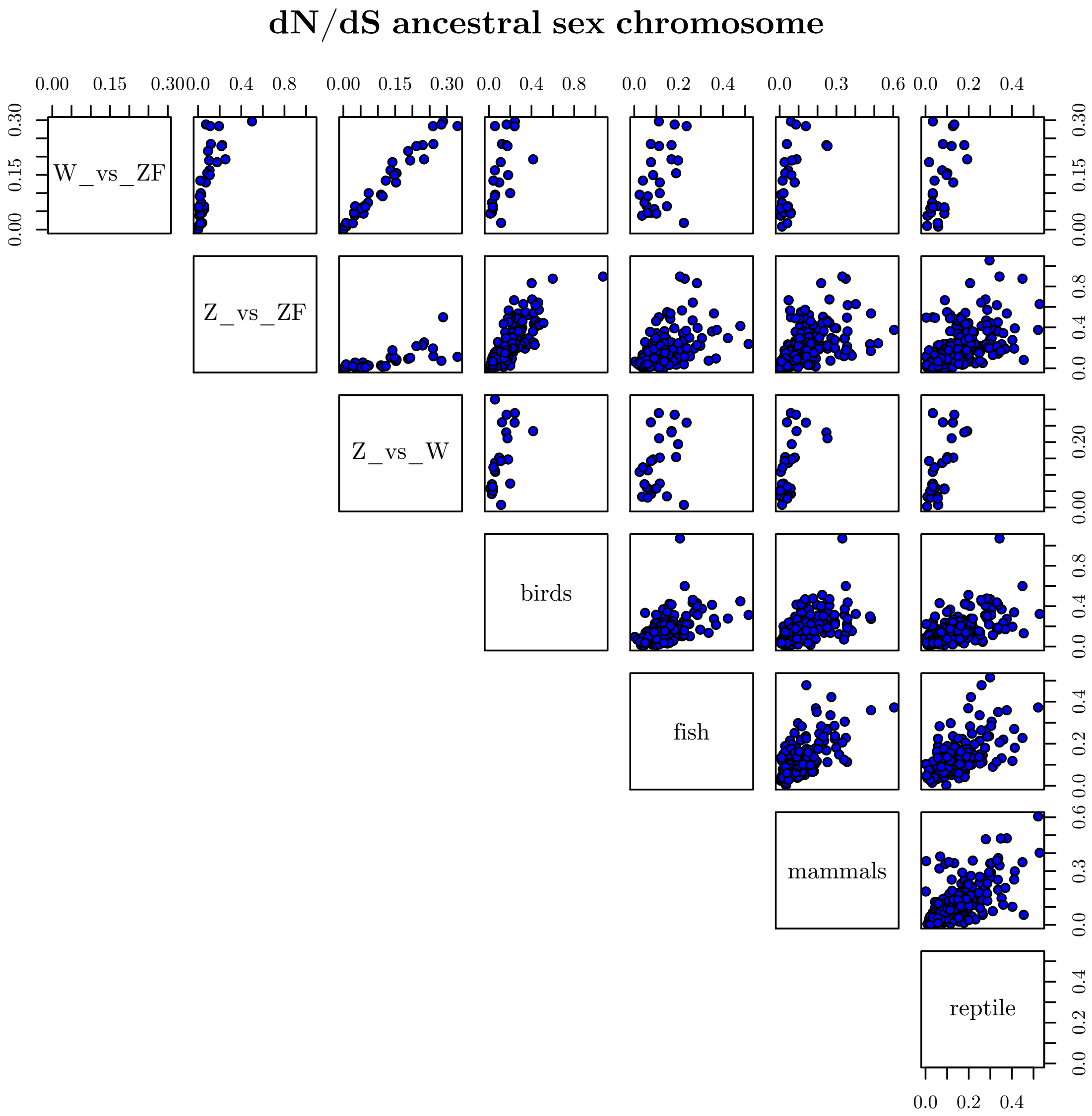

Supplementary Figure 9. Scatter plots of the dN/dS values used to produce Figure 5a. Comparisons: W_vs_ZF (great reed warbler W vs. zebra finch), Z_vs_ZF (great reed warbler Z vs. zebra finch), birds (chicken vs. zebra finch), fish (stickleback vs. fugu), mammals (human vs. mouse) and reptile (green anole vs. bearded dragon).

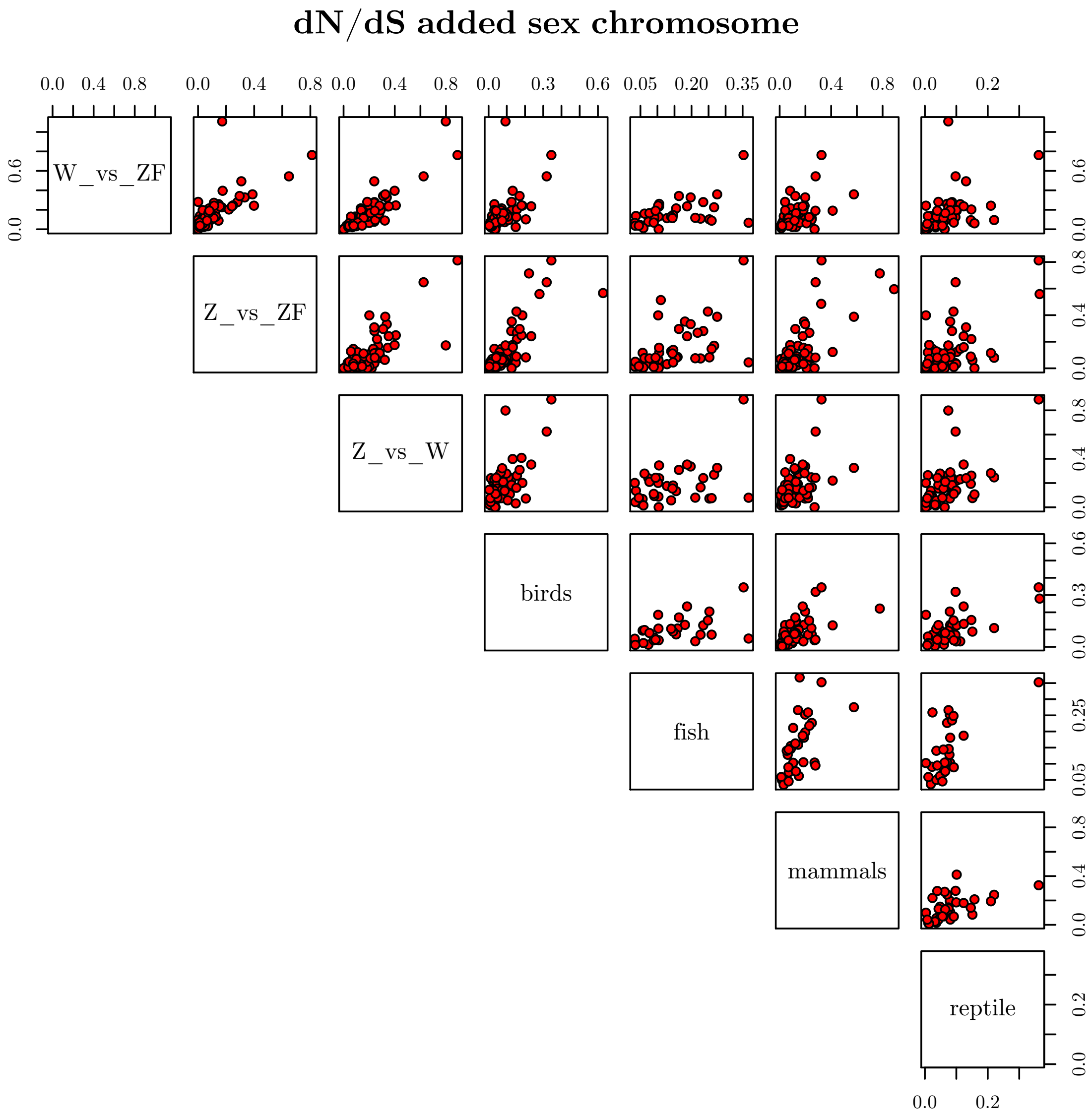

Supplementary Figure 10. Scatter plots of the dN/dS values used to produce Figure 5b. Comparisons: W_vs_ZF (great reed warbler W vs. zebra finch), Z_vs_ZF (great reed warbler Z vs. zebra finch), birds (chicken vs. zebra finch), fish (stickleback vs. fugu), mammals (human vs. mouse) and reptile (green anole vs. bearded dragon).
