# Supplementary material for: Avian Neo-Sex Chromosomes Reveal Dynamics of Recombination Suppression and W Degeneration
Source: Mol Biol Evol. 2021 Sep 20;38(12):5275–91. doi: 10.1093/molbev/msab277 (PMC8662655; doi:10.1093/molbev/msab277)
Supplement: msab277_Supplementary_Data [file msab277_supplementary_data.zip › Supplementary Text.pdf]

Supplementary Text

1 Great reed warbler genome sequencing, assembly and annotation

1.1 Extraction and library preparation

1.1.1 DNA extraction

High molecular weight DNA was extracted from blood of a juvenile female great reed warbler (used to produce the genome assembly; Supplementary Table 1a) and kept in -80°C in SET buffer (0.15 M NaCl, 0.05 M Tris, 0.001 M EDTA, pH 8.0) using standard phenol-chloroform extraction (Sambrook et al. 1989) with initial RNase treatment and Proteinase K digestion and final collection of purified DNA on a glass rod.

1.1.2 RNA extraction

RNA from the same individual was extracted from snap frozen liver, heart and muscle tissue that was kept in -80°C. The extraction was carried out using RNeasy mini kit (Qiagen, cat no. 74104), with 15 min on-column DNase treatment, according to the manufacturer's instructions. The RNA was used for generating short-read Illumina RNA-seq data (Supplementary Table 1a).

1.1.3 Iso-Seq libraries

To construct cDNA libraries for PacBio sequencing (Iso-Seq), mRNA was first purified from total RNA from the genome individual by two rounds of polyA-selection using Poly(A)Purist MAG kit (Ambion, cat nr AM1922) according to the manufacturer's instructions. The mRNA was used for cDNA synthesis according to "Procedure and Checklist – Isoform Sequencing (Iso-Seq) using the Clontech SMARTER PCR cDNA Synthesis kit and BluePippin Size-selection System" (Pacific Biosciences) and was prepared to an Iso-Seq library according to "Guidelines for Preparing cDNA Libraries for Isoform Sequencing (Iso-Seq) User Bulletin" (Pacific Biosciences).

1.2 Genome assembly

1.2.1 Long-read *de novo* assembly

The PacBio library was sequenced on 108 SMRT cells of the RSII instrument using the P6-C4 chemistry, which generated 13M subreads (117.4 Gb in total) with a mean length of 8.9 kb and a N50 read length of 11.7 kb (Supplementary Table 1a). All sequences shorter than 500 bp or with a quality (QV) < 80 were filtered out. The resulting set of subreads was then used for *de novo* assembly with FALCON v0.5.0 (Chin et al. 2016) using a pre-assembly length cutoff of 8 kb according to the following general configuration file:

```
input_fofn = input.fofn

length_cutoff = 8000
length_cutoff_pr = 12000

target = assembly

job_type = sge
job_queue = falconqueue1
sge_option_da = -pe fpe 4 -q %(job_queue)s
sge_option_la = -pe fpe 4 -q %(job_queue)s
sge_option_cns = -pe fpe 8 -q %(job_queue)s
sge_option_pda = -pe fpe 4 -q %(job_queue)s
sge_option_pla = -pe fpe 4 -q %(job_queue)s
sge_option_fc = -pe fpe 16 -q %(job_queue)s

default_concurrent_jobs = 96

pa_DBsplit_option = -x500 -s400
ovlp_DBsplit_option = -x500 -s400

falcon_sense_option = --output_multi --min_idt 0.70 --min_cov 4 --max_n_read 200 --n_core 8 --
min_cov_aln 4 --min_len_aln 40

overlap_filtering_setting = --max_diff 100 --max_cov 100 --min_cov 1 --n_core 8 --bestn 10

pa_HPCdaligner_option =   -v -B128 -t16 -e.70 -l1000 -s1000 -M28
ovlp_HPCdaligner_option = -v -B128 -t32 -e.96 -l500  -s1000 -M28 -h60

falcon_sense_skip_contained = false
skip_checks = True
dust = false
dazcon = false

use_tmpdir = /scratch
```

The draft assembly was error corrected twice with the same set of PacBio subreads that was used for *de novo* assembly using Quiver (Chin et al. 2013).

1.2.2 Error correction using Illumina data

The draft assembly was error corrected again using Illumina paired-end reads (2×150 bp) from the same individual that was used for PacBio sequencing (Supplementary Table 1a). The paired-end reads were first trimmed using Trimmomatic v.0.36 (Bolger et al. 2014) using the following settings: TruSeq3-PE.fa:2:30:10 LEADING:15 TRAILING:30 SLIDINGWINDOW:4:20 MINLEN:90. Of the original 479,067,329 read pairs, 356,222,474 (74.36%) survived trimming of both reads. The surviving read pairs were aligned to the PacBio draft assembly using bwa mem v.0.7.15 (Li & Durbin 2009), transformed to bam format and sorted with samtools v.0.1.19 (Li et al. 2009). Duplicate reads were then removed using picard MarkDuplicates v.2.0.1 (http://broadinstitute.github.io/picard). The number of aligned and deduplicated reads were 632,245,634. The aligned reads were then used to polish the genome assembly with Pilon version 1.17 (Walker et al. 2014) using the options --genome --frags -diploid --fixbases. The error corrected draft assembly consisted of 8,274 contigs in total, with a total genome length of 1.35 Gb. From this draft assembly, we extracted only the primary contigs (5,419 contigs with a total genome size of 1.22 Gb and N50 of 3.7 Mb).

1.2.3 Misassembly detection and scaffolding with linked-read data

We used Chromium linked-read data (10x Genomics) to identify and break apart scaffolds at suspected misassembled sites, and then to increase contiguity through scaffolding (Supplementary Table 1a). The linked-read data was demultiplexed and transformed to fastq format using the supernova mkfastq program from Supernova v.1.1.5 (Weisenfeld et al. 2017). The barcodes from the fastq data were processed and error corrected using the longranger basic (v.2.1.6) tool from 10x Genomics. After processing, 408 million read pairs remained with 95.6% whitelisted barcodes and a barcode diversity of 752,722. We used tigmint (Jackman et al. 2018) with settings as=100, depth_treshold=65, minsize=2000, number of mismatches=5 to break contigs at suspected misassemblies and low-quality regions. Next, arcs (Yeo et al. 2017) was used to scaffold the contigs using settings c = 5, e=30000 and r=0.05, followed by links v1.8.5 (also from the arcs pipeline) using settings -a 0.9 and -l 5. The resulting draft assembly consisted of 9,823 scaffolds (of which 6,727 are longer than 1,000 bp). The total length was still 1.2 Gb, scaffold N50 was 19 Mb and GC content was 43%.

In order to detect additional misassemblies in the polished and scaffolded draft assembly, we aligned it to the genome assembly of the zebra finch (taeGut3.2.4; Warren et al. 2010, downloaded from Ensembl; Yates et al. 2020) using SatsumaSynteny v2.0 (Grabherr et al. 2010). For each chromosome in the zebra finch assembly, we extracted genomic coordinates from great reed warbler draft assembly scaffolds that aligned to this chromosome using BEDTools merge (v2.27.1; Quinlan and Hall 2010) with option -d 100000 to combine genomic coordinates of warbler scaffolds that overlapped or that were within 100 kb of one another, oriented along the zebra finch chromosome coordinates. Only

alignments to the zebra finch genome that were larger than 100 kb were considered. Then, we used BEDTools complement to create genomic ranges also for the genomic regions that were not part of the zebra finch alignment dataset, if these ranges were longer than 100 bp. We then used BEDTools getfasta to make a new fasta file where scaffolds were cut according to these genomic ranges. This means that scaffolds that align to two separate zebra finch chromosomes (or only partly to a zebra finch chromosome) will be split into two in the breakpoint region. This new fasta file was processed with arcs and links for a second round of scaffolding using the same settings as above. The new draft assembly consists of 7,985 scaffolds (of which 6,531 are longer than 1,000 bp). While the length of the assembly remained almost unchanged, this scaffolding step increased the scaffold N50 to 21 Mb.

1.2.4 Scaffolding with optical mapping data

We then used Bionano optical mapping data to further increase the contiguity of our data (Supplementary Table 1a). DNA was extracted using the agarose plug method from blood (in SET buffer) of the same individual as above (§1.1.1). Two enzymes were used; BSPQI and BSSSI. The data from each enzyme was first assembled into separate *de novo* assemblies (using the script pipelineCL.py from Bionano Solve with settings -U -d -T 228 -j 228 -N 4 -i 5). The script runTGH.R from Bionano Solve (version Solve3.1_08232017) was used to anchor the scaffolds from the draft assembly to the optical mapping assemblies using standard settings, options: -e1 BSPQI -e2 BSSSI and using the provided configuration file 'hybridScaffold_two_enzymes.xml'. Scaffolds with a combined length of 1.1 Gb (N50: 19 Mb) were anchored in the new hybrid assembly, which had an N50 value of 20.5 Mb.

1.2.5 Gapfilling and additional error correction

To fill in gaps in the draft assembly, we used gapfiller (Nadalin et al. 2012) with short reads and PBjelly from PBSuite v15.8.24 (English et al. 2012) with PacBio long reads. To remove potential sequencing errors, the whole assembly was once more subjected to two rounds of Quiver polishing. All scaffolds shorter than 1,000 bp were removed from the assembly.

1.2.6 Splitting up of chimeric scaffolds

Seven scaffolds were manually broken apart at misassembled sites, identified through (i) the linkage map data (Ponnikas et al. 2020), (ii) aligned genomic Illumina reads from the same individual that was used to create the reference genome, and (iii) synteny information from the zebra finch genome. The scaffolds were split in the fasta file and the GFF file containing gene annotations using the script https://github.com/NBISweden/NBIS-UtilityCode/SplitFastaAndGFF.cc. None of the gene annotations was overlapping with any of the breakpoints.

1.2.7 Removal of redundant scaffolds

We removed redundant scaffolds that represent haplotypes of another scaffold (“haplotigs”) by using the purge haplotigs pipeline (Roach et al. 2018). For the pipeline we first estimated coverage for each scaffold by mapping PacBio subreads to the assembly using minimap2 v.2.13 (Li 2018). Based on the coverage distribution in the genome we set 60x as the threshold between haploid and diploid coverage. Any scaffold with a diploid coverage less than 80% was considered as a suspect haplotig and was searched against other scaffolds within the software. We removed scaffolds that had a best match coverage of at least 95% to its best hit. This resulted in the removal of 3,468 scaffolds with a mean length of 14,543 bp (range: 1,001 bp – 920,475 bp).

1.3 Annotation

1.3.1 RNA sequencing data generation and processing

We trimmed RNA-seq Illumina reads (Supplementary Table 1a) with trimmomatic v 0.36 (Bolger et al. 2014) using the parameters TruSeq3-PE-2.fa:2:30:10 SLIDINGWINDOW:4:5 LEADING:5 TRAILING:5 MINLEN:25, as suggested in the Trinity (Grabherr et al. 2011) documentation. Of 512,409,808 raw Illumina reads, 507,717,996 reads remained after trimming. For a reference-guided transcriptome assembly, the draft genome assembly was indexed using bowtie2 v.2.3.2 (Langmead & Salzberg 2012). We then aligned the trimmed RNA-seq reads separately for each tissue to the assembly using tophat v.2.1.1 (Kim et al. 2013) with the option --library-type=fr-firststrand. Accepted hits from the three tissues were merged into one file using samtools (v.1.3; Li et al. 2009) merge. The reads were then assembled using StringTie v.1.3.3 (Pertea et al. 2015) and cufflinks v.2.2.1 (Trapnell et al. 2010). The output was transformed from GTF to GFF format using gffread (Pertea & Pertea 2020) with option -E. We performed a *de novo* assembly using the trimmed RNA-seq Illumina reads combined for all three tissue samples in Trinity v.2.3.2 (Grabherr et al. 2011) with the options “--seqType fq --SS_lib_type RF”. Iso-Seq data was processed according to the “RS_IsoSeq” protocol version 2.3.0.

1.3.2 Gene builds

We then predicted gene models using MAKER v.3.00.0 (Holt & Yandell 2011; Campbell et al. 2014). The reference-guided and *de novo* RNA-seq assemblies from Illumina short read data (Supplementary Table 1a) were provided to MAKER along with an assembly of the Iso-Seq data as species-specific evidence. As additional evidence manually reviewed protein sequences (556,825 proteins) from the SwissProt section of the UniProt database were downloaded (2018-03) (Magrane & UniProt Consortium 2011), along with protein files from chicken (Gallus_gallus.Gallus_gallus-5.0.pep.all.fa; containing 30,252 proteins) and zebra finch (Taeniopygia_guttata.taeGut3.2.4.pep.all.fa; containing 18,204 proteins). We provided the repeat library fAlb15_rm3.0_aves_hc.lib and the output from

RepeatModeler (see above) as input for repeat masking with RepeatMasker and RepeatRunner (Yandell 2006) which are run internally by MAKER. We used the *ab-initio* gene finder Augustus v.3.2.3 (Stanke et al. 2006) with the pre-trained profile of chicken. Gene builds were constructed in MAKER, using 1) only the extrinsic evidence (proteins and transcripts), and 2) combining the gene builds from extrinsic evidence sequences with *ab-initio* predictions in Augustus. As the evidence run performed better than the *ab-initio* run (evaluated using BUSCO: 82.5% complete genes compared to 66.6%; and through visual inspection), we used the evidence run as a base and complemented it with the Augustus annotation track created during the *ab-initio* run using an in-house perl script (https://github.com/NBISweden/AGAT/blob/master/bin/agat_sp_complement_annotations.pl). Another in-house perl script (https://github.com/NBISweden/AGAT/blob/master/bin/agat_sp_fix_longest_ORF.pl) was used to improve the ORF start and end positions, to improve fragmented and missing genes.

1.3.3 Functional annotation

We inferred the function of genes and transcripts using the translated CDS features of each coding transcript. To retrieve a gene name and function of gene, we (i) blasted the predicted protein sequence of each transcript against the Uniprot/Swissprot reference dataset and (ii) ran the same sequences in InterProScan v-5.7-48 (Jones et al. 2014). Then, the Annie annotation tool (Tate et al. 2014) was used to extract relevant metadata into predictions for canonical protein names and functional predictions. This resulted in 20,807 gene models with 1,717 gene models without functional annotations. Gene names were inferred with a best blast hit approach using the Uniprot/Swissprot reference dataset. In total, 18,559 genes were named, of which 2,312 had duplicate gene names. We predicted tRNAs using tRNAscan v.1.3.1 (Lowe et al. 1997) (450 tRNAs) and other ncRNAs using the RNA family database Rfam v.11 (Nawrocki et al. 2014). A lift-over of annotations to the great reed warbler genome was done using the (i) zebra finch (Taeniopygia_guttata.taeGut3.2.4.94; 17,487 genes) and (ii) chicken (Gallus_gallus.Gallus_gallus-5.0.94; 18,345 genes) ensemble gene annotations. We first did pairwise alignments between the great reed warbler genome and the other genomes using SatsumaSynteny v.3.0. Then, Kraken (Zamani et al. 2014) was used to project the annotations from one genome to another using the pairwise alignments. Finally, an in-house script (https://github.com/NBISweden/AGAT/blob/master/bin/agat_sp_kraken_assess_liftover.pl) was used to handle the gene lift-overs. From the zebra finch, 14,686 genes were successfully lifted over (642 genes mapping to several locations), and 14,466 from chicken (571 genes to several locations).

2 Sex chromosome analyses

2.1 Sex-linked scaffolds

We aligned paired-end sequence data (Illumina HiseqX 150 PE) from five female (none being the reference genome individual) and five male great reed warbler individuals to the reference genome (sample information in Supplementary Table 1b). The reads were trimmed using Trimmomatic v.0.36 (Bolger et al. 2014) prior to alignment using settings TruSeq3-PE.fa:2:30:10 LEADING:15 TRAILING:30 SLIDINGWINDOW:4:20 MINLEN:90. Reads were aligned using bwa mem v.0.7.15 (Li & Durbin 2009) (option -M), alignments were sorted and converted to the bam format using samtools v.1.7 (Li et al. 2009) and reads were deduplicated with picard MarkDuplicates v.2.0.1 (http://broadinstitute.github.io/picard). We followed the general method from Smeds et al. (2015) for identifying W-linked scaffolds by first parsing the alignment files for reads with any mismatching base pairs (bam file tag NM:i:0). Then, per site genome coverage was calculated using samtools depth for reads with a minimum mapping quality of 20 and a maximum read depth of 80x (in order to avoid genome coverage values from repetitive regions). All genome coverage values were normalized between samples based on the total number of reads in the trimmed fastq files. The normalized coverage values were summed for each sex (5 females and 5 males), and the per-sex median coverage for each scaffold was calculated. We considered scaffolds where the male coverage was zero while the female coverage was > 25x to be W-linked. This cut-off resulted in 50 W-linked scaffolds with a median female coverage of 68.03x (mean coverage 61.83x), and a mean length of 605 kb (median 58 kb). Of these scaffolds, 15 were represented in the gene annotation and were designated as "W-scaffolds". The 35 scaffolds not present in the annotation file were grouped as "random W-scaffolds" (Supplementary Table 6,7).

To identify Z-linked scaffolds, we utilized the difference between the median coverage values for males and females (following the same method as above) but also the difference in heterozygosity. As females are haploid for Z-linked scaffolds while males are diploid, we expect them to differ in this measurement. We calculated inbreeding coefficients (F) for each scaffold using vcftools v0.1.15 (Danecek et al. 2011) with option --het and calculated the median for each sex. Next, we filtered the scaffolds for Z-linkage based on two criteria; a) either the median coverage in females were less than 55% of the male coverage, or b) the median female coverage was less than 65% and the heterozygosity value for males and females had an absolute difference of more than 0.1. Using this method, 22 scaffolds were considered to be Z-linked. The mean length of these was 4.03 Mb (median 31 kb). Of these 22 scaffolds, 8 were represented in the gene annotation file. Same as with the W-linked scaffolds, we designated these 8 as "Z-linked scaffolds" and the other ones as "random Z-linked scaffolds" (Supplementary Table 6,7). A linkage map analysis (Ponnikas et al. 2020) using a pedigree of 511 great reed warblers assigned seven of these eight Z-linked scaffolds to the same linkage group.

The one scaffold that was not assigned (Scaffold492) was relatively short (0.6 Mb) and had no informative RADseq SNPs in the mapping pedigree. An additional scaffold was identified through the linkage map analysis as belonging to the Z chromosome: Scaffold92. Six of these sex-linked scaffolds (Supplementary Table 7) could be anchored (i.e., ordered and oriented) successfully in the Z linkage group (Ponnikas et al. 2020). Lastly, Scaffold217 was identified as the pseudoautosomal region (PAR) according to the linkage map. This scaffold is 0.9 Mb in length, contains the PAR genes identified in other songbird species and had equal coverage values between the female and male great reed warblers (Ponnikas et al. 2020).

2.2 Gametologs and manual curations of sex-linked genes

The different gene builds generated in MAKER were imported into WebApollo (Lee et al. 2013) along with the protein and RNA-seq evidence, repeat annotations, and lift-overs from zebra finch and chicken, where we manually curated 147 gametologous (ZW) gene pairs and 25 Z-linked genes without a W-copy. For additional guidance in manual curations, we included four additional MAKER gene builds that were generated exclusively for sex-linked scaffolds, using 1) only the extrinsic evidence (proteins and transcripts including Iso-Seq data), and 2) combining the gene builds from extrinsic evidence sequences including Iso-Seq data with *ab-initio* predictions in Augustus, as well as 3) extrinsic evidence without Iso-Seq data, and 4) combining the gene builds from extrinsic evidence without Iso-Seq data with *ab-initio* predictions in Augustus. The identification of gametologs was done in the following way: we went through all W-linked scaffolds with gene annotations (i.e. the 15 scaffolds mentioned above). Each gene was blasted (blastx) against the non-redundant protein sequence database on NCBI and manually curated and the best supported isoform for each gene was selected. The same was done for the scaffolds marked as “Z-linked” that aligned either fully or partly to chromosome 4A in the zebra finch genome. To identify gametologous gene pairs on the added sex chromosome region (chromosome 4A) the W-linked and Z-linked genes needed to fulfil two of the following three criteria; i) the gene should be flanked by the same genes as in the zebra finch (or the chicken if the gene was not placed within the zebra finch genome, i.e on a random or Un chromosome), ii) there should be lift-over evidence to the same transcripts in either zebra finch or chicken, and iii) the genes should belong to the same orthology group based on an orthology analysis done using orthoMCL.

The gene order for ancestral W-linked genes is expected to be heavily scrambled between species. Therefore, we accepted W-linked genes where evidence from both the last two criteria (lift-over evidence and orthology evidence) were fulfilled, regardless of gene order. For all of those gene transcripts, we searched for transcripts present in the orthology analysis that were located on a Z-linked scaffold. These Z-linked scaffolds were accepted based on the same criteria as the 4A-linked genes (i.e. also having either gene order or lift-over support). Z-linked copies of four accepted W-

linked genes were missing from the orthology analysis, but were found in the gene annotation as they were located between the expected genes (i.e. conserved gene order) in the zebra finch or chicken, and had lift-over evidence matching the same gene as the W-linked gene copy. We identified 41 gametologous gene pairs from the ancestral sex chromosome with these criteria.

In total, we found 131 genes belonging to the added-Z region. Two of these were placed on zebra finch chromosome 4A_random, but in the correct place according to synteny in chicken, and the remaining 129 on chromosome 4A. Of these 131 genes, 106 genes were also found on the added-W region. From the remaining 25 genes, three genes had a W copy but insufficient ortholog evidence and for 22 genes we found only Z-linked transcripts. We also identified 277 Z-linked genes without a W-copy as follows: First, we downloaded information on orthologs from the following species: green anole (*Anolis carolinensis*; AnoCar2.0; GCA_000090745.1), emu (*Dromaius novaehollandiae*; droNov1; GCA_003342905.1), great spotted kiwi (*Apteryx haastii*; aptHaa1; GCA_003342985.1), chicken (*Gallus gallus*; GRCg6a; GCA_000002315.5), mallard (*Anas platyrhynchos platyrhynchos*; CAU_duck1.0; GCA_002743455.1), budgerigar (*Melopsittacus undulatus*; Melopsittacus_undulatus_6.3; GCA_000238935.1), blue-crowned manakin (*Lepidothrix coronata*; Lepidothrix_coronata-1.0; GCA_001604755.1), collared flycatcher (*Ficedula albicollis*; FicAlb_1.4; GCA_000247815.1), and great tit (*Parus major*; Parus_major1.1; GCA_001522545.2). We selected all genes that were present and classified as one-to-one orthologs in all species. We intersected these genes with genes that grouped with a single great reed warbler transcript in the ortholog analysis, and lastly, we selected only those transcripts corresponding to zebra finch transcripts located on either the Z chromosome or Z_random. From this list, we removed two of the transcripts which were also present in our gametolog analysis, meaning that they have a W copy (corresponding to zebra finch Ensembl transcript IDs ENSTGUT00000000103 and ENSTGUT00000001787).

We extracted these great reed warbler transcripts (147 manually curated ZW gene pairs, 22 manually curated Z-linked genes and 277 uncurated Z sequences) from the reference genome and added the zebra finch transcript for each gene. The sequences were aligned using the codon-aware aligner prank v.170427 (Löytynoja 2014) and removed gaps using Gblocks v.0.91b (Castresana 2000). After filtering for a minimum length of 500 bp and $dS < 3$, 79 added sex chromosome gene pairs remained, and 18 added-Z genes without a W-linked gene copy. On the ancestral sex chromosome, 35 gene pairs remained after filtering. Of the uncurated ancestral Z-linked genes without a W copy, 238 remained after filtering. We calculated pairwise substitution rates between the three sequences (great reed warbler Z and W, and zebra finch) per gene using codeml from the PAML package v4.9 (Yang et al. 2007).

2.3 Constructing a dated phylogeny

To get a set of autosomal genes, we downloaded one-to-one orthologs from the same set of outgroup species (*n* = 7; see above) which were not on chromosome 4A or Z in the zebra finch genome (i.e. on chromosomes 1, 1A, 1B, 2-15, or 17-28) based on gene information on BioMart (accessed on 30 May 2019). Using the zebra finch transcript ID, we then searched for these genes in the orthoMCl group.txt file and selected those genes where there was only a single great reed warbler transcript in the ortholog file. Using this method, which resulted in 3,570 genes, we wanted to ensure that we were using only single copy orthologs. We then randomly selected 100 genes (using the bash command shuf -n 100) and extracted autosomal sequences from five other Sylvioidea species (major alleles based on alignments of the samples indicated in Supplementary Table S1b) using the positions of these transcripts in the gene annotation. The topology of the phylogeny shown in Figure 3 was compiled from Jarvis et al. (2014) and Oliveros et al. (2019). This fixed topology was then dated with MCMCTree in PAML v.4.8a (Yang et al. 2007) using alignments of 69 concatenated autosomal sequences from all 13 species (see above) and the following calibration times (also from Jarvis et al. 2014 and Oliveros et al. 2019): divergence of lizard-bird (255.9 - 299.8 Myr), Palaeognathae-Neognathae (66 - 99.6 Myr), Psittaciformes-Passeriformes (51.81 - 66.5 Myr) and suboscine-oscine passerines (27.25 - 56 Myr; Supplementary Figure 3).

REFERENCES

Bolger AM, Lohse M, Usadel B. 2014. Trimmomatic: a flexible trimmer for Illumina sequence data. *Bioinformatics* 30(15):2114–2120.

Campbell MS, Holt C, Moore B, Yandell M. 2014. Genome Annotation and Curation Using MAKER and MAKER-P. Curr Protoc *Bioinformatics* 48, 4.11.1–39.

Castresana J. 2000. Selection of conserved blocks from multiple alignments for their use in phylogenetic analysis. *Mol. Biol. Evol.* 17(4):540–552.

Chin CS, Alexander DH, Marks P, Klammer AA, Drake J, Heiner C, Clum A, Copeland A, Huddleston J, Eichler EE, et al. 2013. Nonhybrid, finished microbial genome assemblies from long-read SMRT sequencing data. *Nat. Methods 10*(6):563–569.

Danecek P, Auton A, Abecasis G, Albers CA, Banks E, DePristo MA, Handsaker RE, Lunter G, Marth GT, Sherry ST, et al. 2011. The variant call format and VCFtools. *Bioinformatics* 27(15):2156–2158.

English AC, Richards S, Han Y, Wang M, Vee V, Qu J, Qin X, Muzny DM, Reid JG, Worley KC, et al. 2012. Mind the Gap: Upgrading Genomes with Pacific Biosciences RS Long-Read Sequencing Technology. *PLOS ONE 7*(11):e47768.

Grabherr MG, Haas BJ, Yassour M, et al. 2011. Full-length transcriptome assembly from RNA-Seq data without a reference genome. *Nat Biotechnol*. 29(7):644-652.

Holt C, Yandell M. 2011. MAKER2: an annotation pipeline and genome-database management tool for second-generation genome projects. BMC Bioinformatics 12, 491.

Jackman SD, Coombe L, Chu J, Warren RL, Vandervalk BP, Yeo S, Xue Z, Mohamadi H, Bohlmann J, Jones SJM, et al. 2018. Tigmint: correcting assembly errors using linked reads from large molecules. *BMC Bioinformatics* 19(1):1–10.

Jarvis ED, Mirarab S, Aberer AJ, Li B, Houde P, Li C, Ho SYW, Faircloth BC, Nabholz B, Howard JT, Suh A, et al. 2014. Whole-genome analyses resolve early branches in the tree of life of modern birds. *Science* 346(6215):1320-31.

Jones P, Binns D, Chang HY, Fraser M, Li W, McAnulla C, McWilliam H, Maslen J, Mitchell A, Nika G, et al. 2014. InterProScan 5: genome-scale protein function classification. *Bioinformatics* 30(9):1236–1240.

Kim D, Pertea G, Trapnell C, Pimentel H, Kelley R, Salzberg SL. 2013. TopHat2: accurate alignment of transcriptomes in the presence of insertions, deletions and gene fusions. *Genome Biol.* 14(4):R36.

Langmead B, Salzberg SL. 2012. Fast gapped-read alignment with Bowtie 2. *Nat. Methods* 9(4):357–359.

Lee E, Helt GA, Reese, JT, Munoz-Torres MC, Childers CP, Buels RM, Stein L, Holmes IH, Elsik CG, Lewis SE. 2013. Web Apollo: a web-based genomic annotation editing platform. *Genome Biol*. 14(8):1–13.

Li H, 2018. Minimap2: pairwise alignment for nucleotide sequences. *Bioinformatics* 34(18):3094–3100.

Li H, Durbin R. 2009. Fast and accurate short read alignment with Burrows–Wheeler transform. *Bioinformatics* 25(14):1754–1760.

Lowe TM, Eddy SR. 1997. tRNAscan-SE: A Program for Improved Detection of Transfer RNA Genes in Genomic Sequence. *Nucleic Acids Res*. 25(5):955–964.

Magrane M, UniProt Consortium. 2011. UniProt Knowledgebase: a hub of integrated protein data. *Database* bar009.

Nadalin F, Vezzi F, Policriti A. 2012. GapFiller: a de novo assembly approach to fill the gap within paired reads. *BMC Bioinformatics* 13 Suppl 14(Suppl 14):S8.

Roach MJ, Schmidt SA, Borneman AR. 2018. Purge Haplotigs: allelic contig reassignment for third-gen diploid genome assemblies. *BMC Bioinformatics* 19(1):1–10.

Sambrook J, Fritsch EF, Maniatis T. 1989. Molecular Cloning a Laboratory Manual, 2nd ed. New York: Cold Spring Harbor Laboratory Press.

Smeds L, Warmuth V, Bolivar P, Uebbing S, Burri R, Suh A, Nater A, Bureš S, Garamszegi LZ, Hogner S, et al. 2015. Evolutionary analysis of the female-specific avian W chromosome. *Nat. Commun*. 6:7330.

Stanke M, Keller O, Gunduz I, Hayes A, Waack S, Morgenstern B. 2006. AUGUSTUS: ab initio prediction of alternative transcripts. *Nucleic Acids Res.* 34(2):W435–W439.

Oliveros CH, Field DJ, Ksepka DT, Barker FK, Aleixo A, Andersen MJ, Alström P, Benz BW, Braun EL, Braun MJ, et al. 2019. Earth history and the passerine superradiation. *Proc. Natl. Acad. Sci. U.S.A*. 116(16):7916–7925.

Pertea G, Pertea M. 2020. GFF Utilities: GffRead and GffCompare. *F1000Research* 2020 9:304.

Pertea M, Pertea GM, Antonescu CM, Chang TC, Mendell JT, Salzberg SL. 2015. StringTie enables improved reconstruction of a transcriptome from RNA-seq reads. *Nat. Biotechnol.*, 33(3):290–295.

Ponnikas S, Sigeman H, Lundberg M, Hansson B. 2020. Extreme variation in recombination rate and genetic variation along the Sylvioidea neo-sex chromosome. biorXiv 2020.09.25.314054.

Quinlan AR, Hall IM. 2010. BEDTools: a flexible suite of utilities for comparing genomic features. Bioinformatics 26(6):841–842.

Tate R, Hall B, DeRego T, Geib S. 2014. Annie: the ANNotation information extractor. Available from: http://genomeannotation.github.io/annie.

Trapnell C, Williams BA, Pertea G, Mortazavi A, Kwan G, van Baren MJ, Salzberg SL, Wold BJ, Pachter L. 2010. Transcript assembly and quantification by RNA-Seq reveals unannotated transcripts and isoform switching during cell differentiation. *Nat. Biotechnol.* 28(5):511–515.

Walker BJ, Abeel T, Shea T, Priest M, Abouelliel A, Sakthikumar S, Cuomo CA, Zeng Q, Wortman J, Young SK, et al. 2014. Pilon: An Integrated Tool for Comprehensive Microbial Variant Detection and Genome Assembly Improvement. *PLOS ONE*, 9(11):e112963.

Weisenfeld NI, Kumar V, Shah P, Church DM, Jaffe DB. 2017. Direct determination of diploid genome sequences. *Genome Res.* 27(5):757–767.

Yandell, M. Comparative Genomics Library-RepeatRunner. 2006. http://www.yandell-lab.org/repeat_runner/index.html.

Yang Z. 2007. PAML 4: Phylogenetic Analysis by Maximum Likelihood. *Mol. Biol. Evol.* 24(8):1586– 1591.

Yates AD, Achuthan P, Akanni W, Allen J, Allen J, Alvarez-Jarreta J, et al. 2020. Ensembl 2020. Nucleic acids research, 48(D1), D682–D688.

Yeo S, Coombe L, Warren RL, Chu J, Birol I. 2017. ARCS: Assembly Roundup by Chromium Scaffolding. *Bioinformatics* 34(5):100750.

Zamani N, Sundström G, Meadows JR, Höppner MP, Dainat J, Lantz H, Haas BJ, Grabherr MG. 2014. A universal genomic coordinate translator for comparative genomics. *BMC Bioinformatics* 15(1):227.
