# Supplementary material for: Avian Neo-Sex Chromosomes Reveal Dynamics of Recombination Suppression and W Degeneration
Source: Mol Biol Evol. 2021 Sep 20;38(12):5275–91. doi: 10.1093/molbev/msab277 (PMC8662655; doi:10.1093/molbev/msab277)

ENSTGUT00000000024

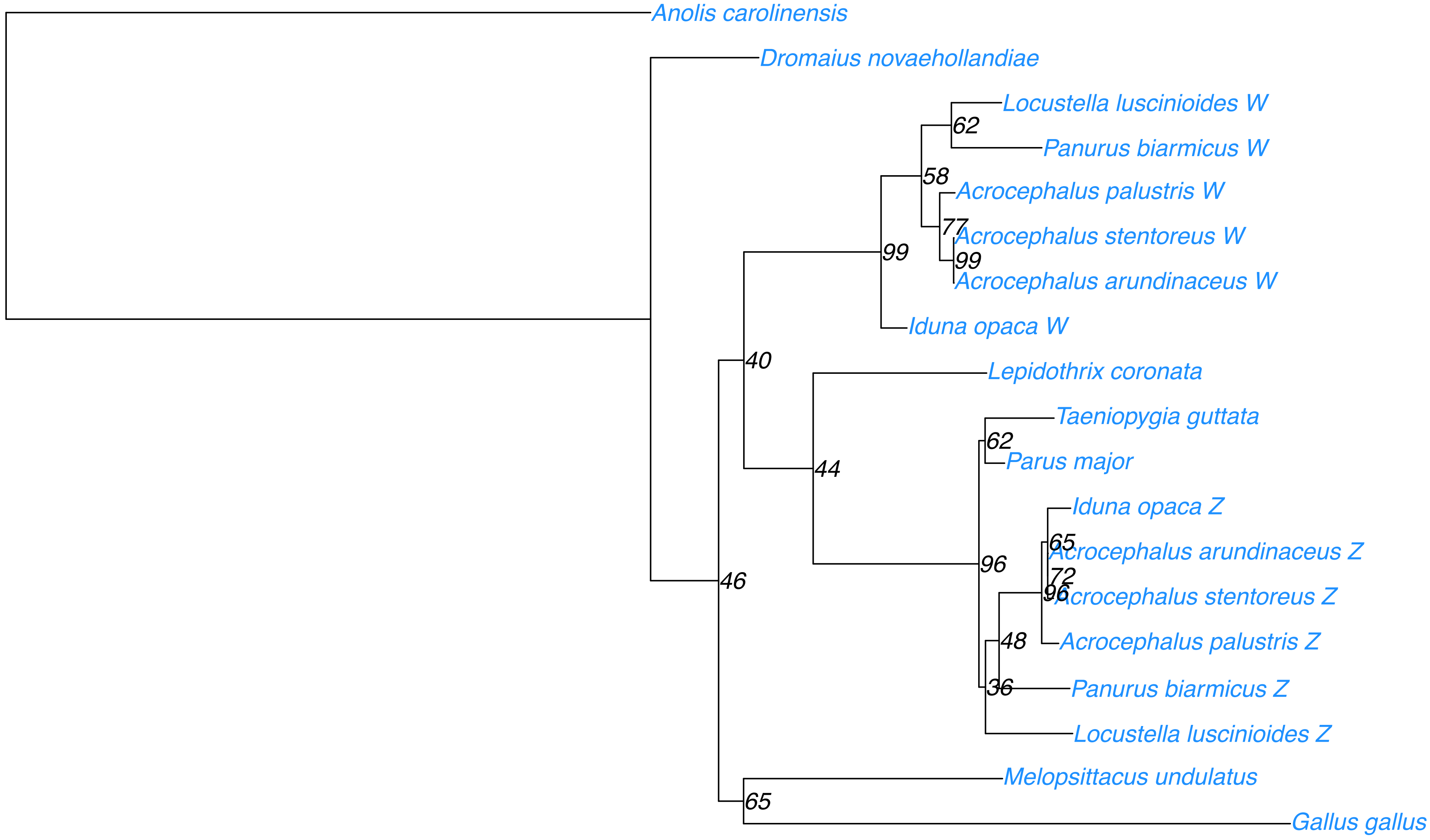

ENSTGUT00000005526

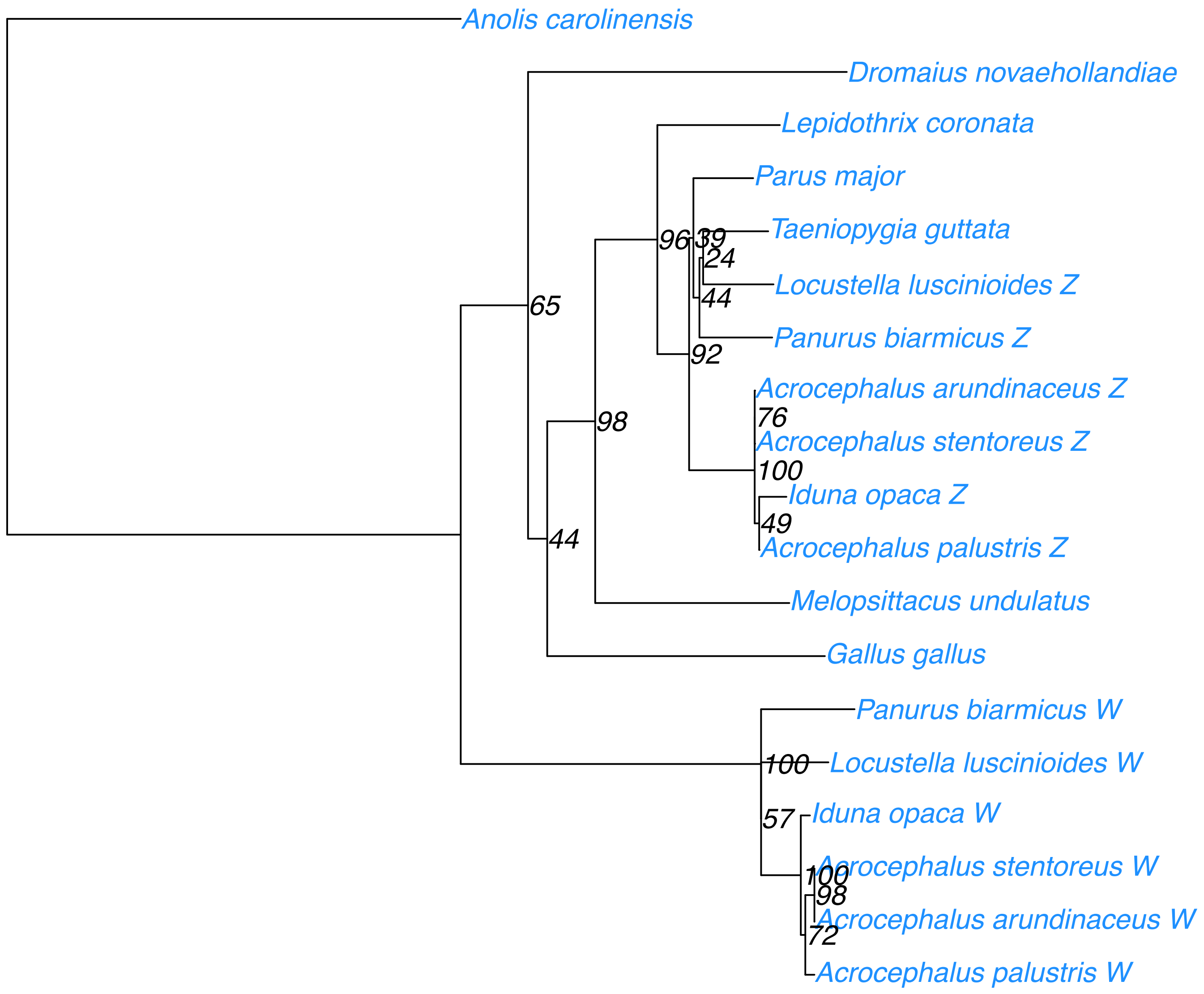

ENSTGUT00000005903

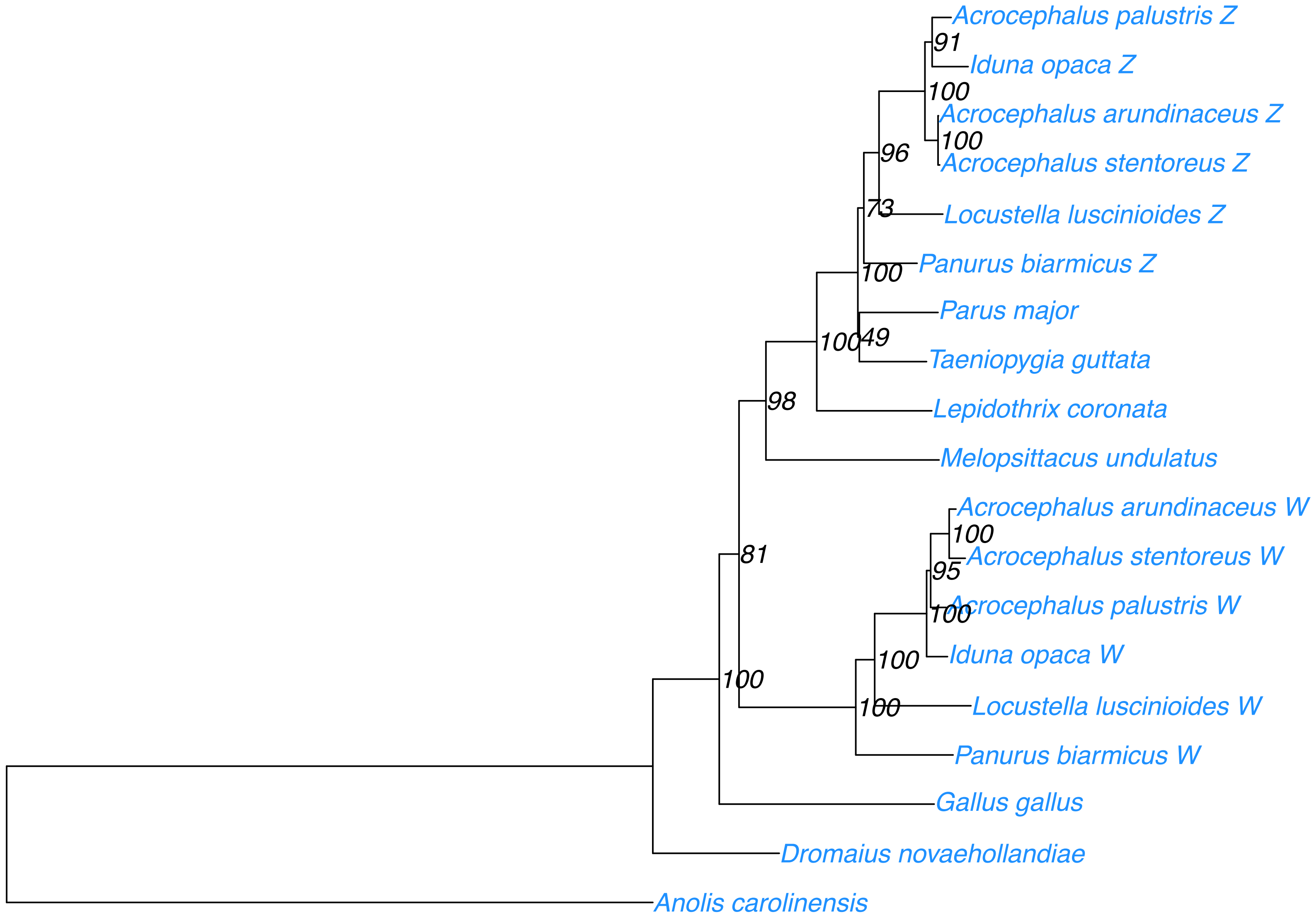

ENSTGUT00000005975

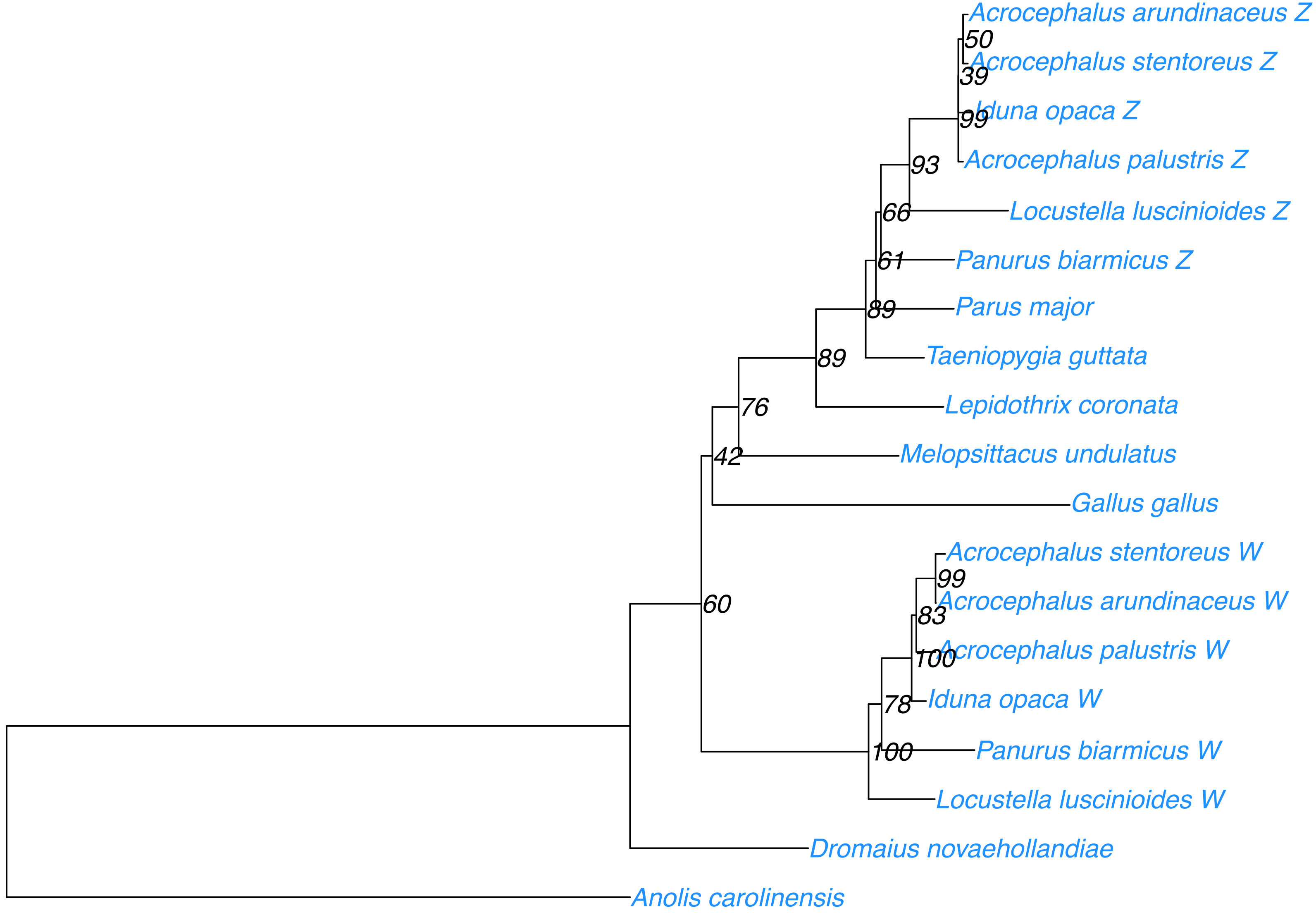

ENSTGUT00000006055

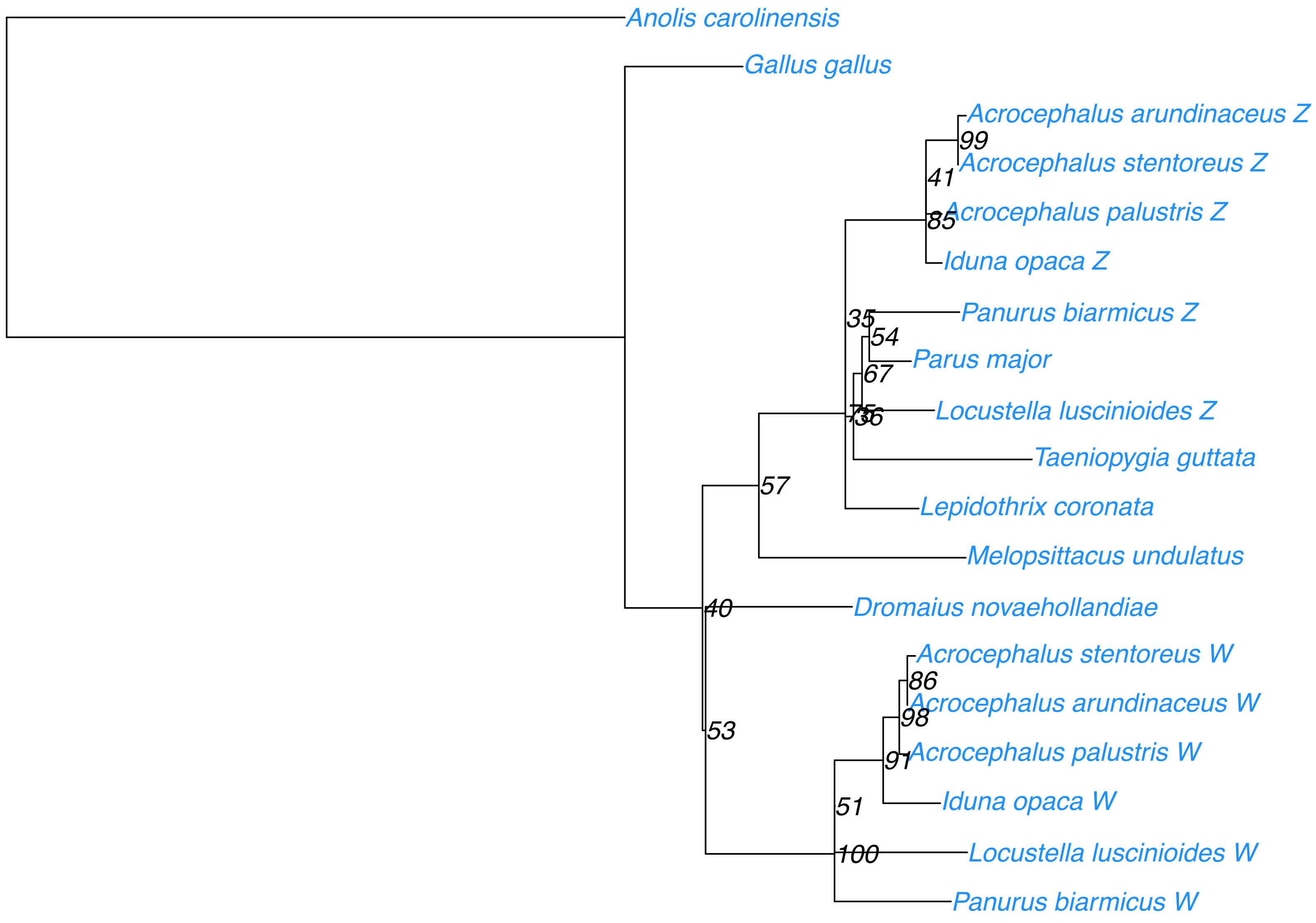

ENSTGUT00000006399

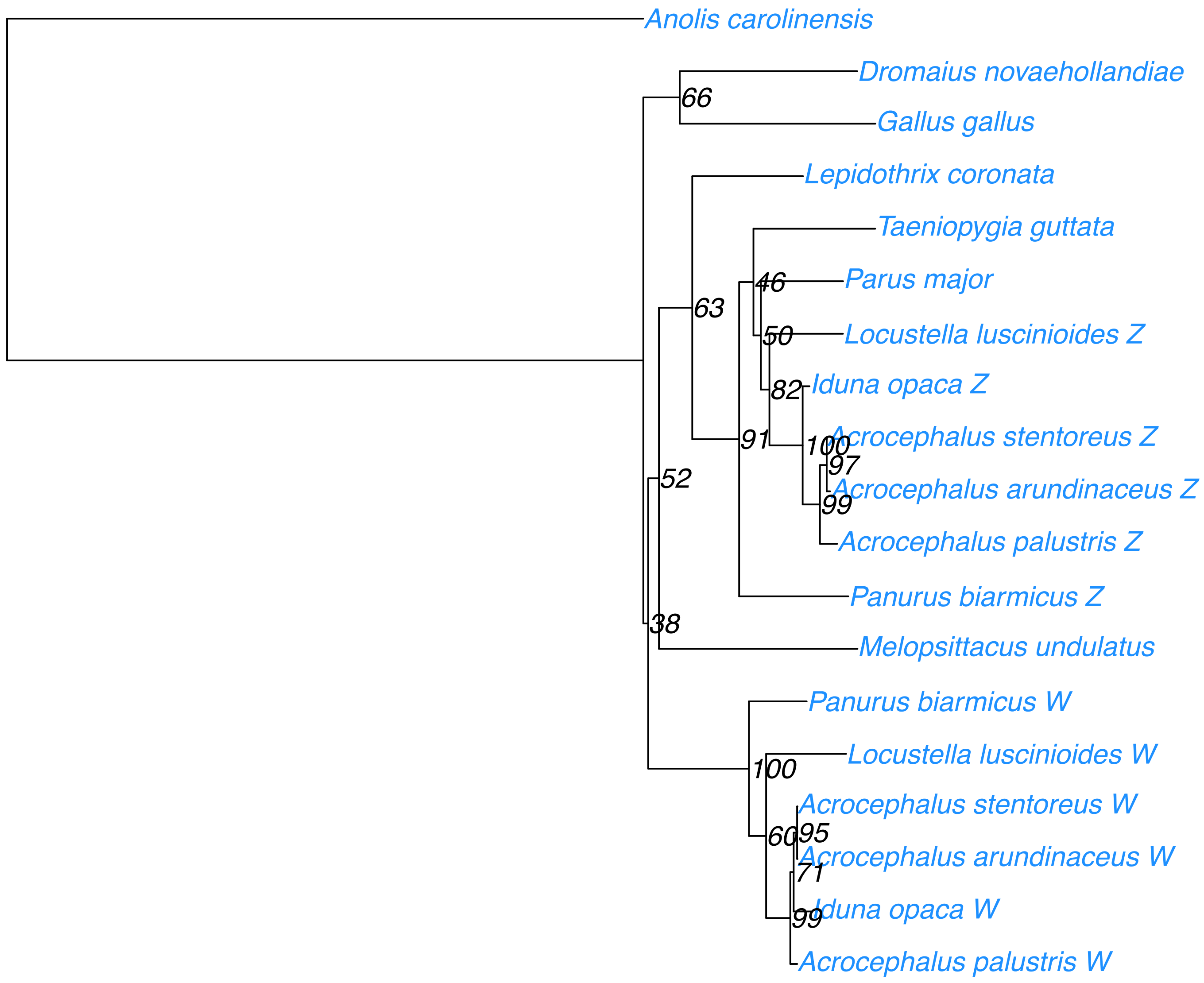

ENSTGUT00000002854

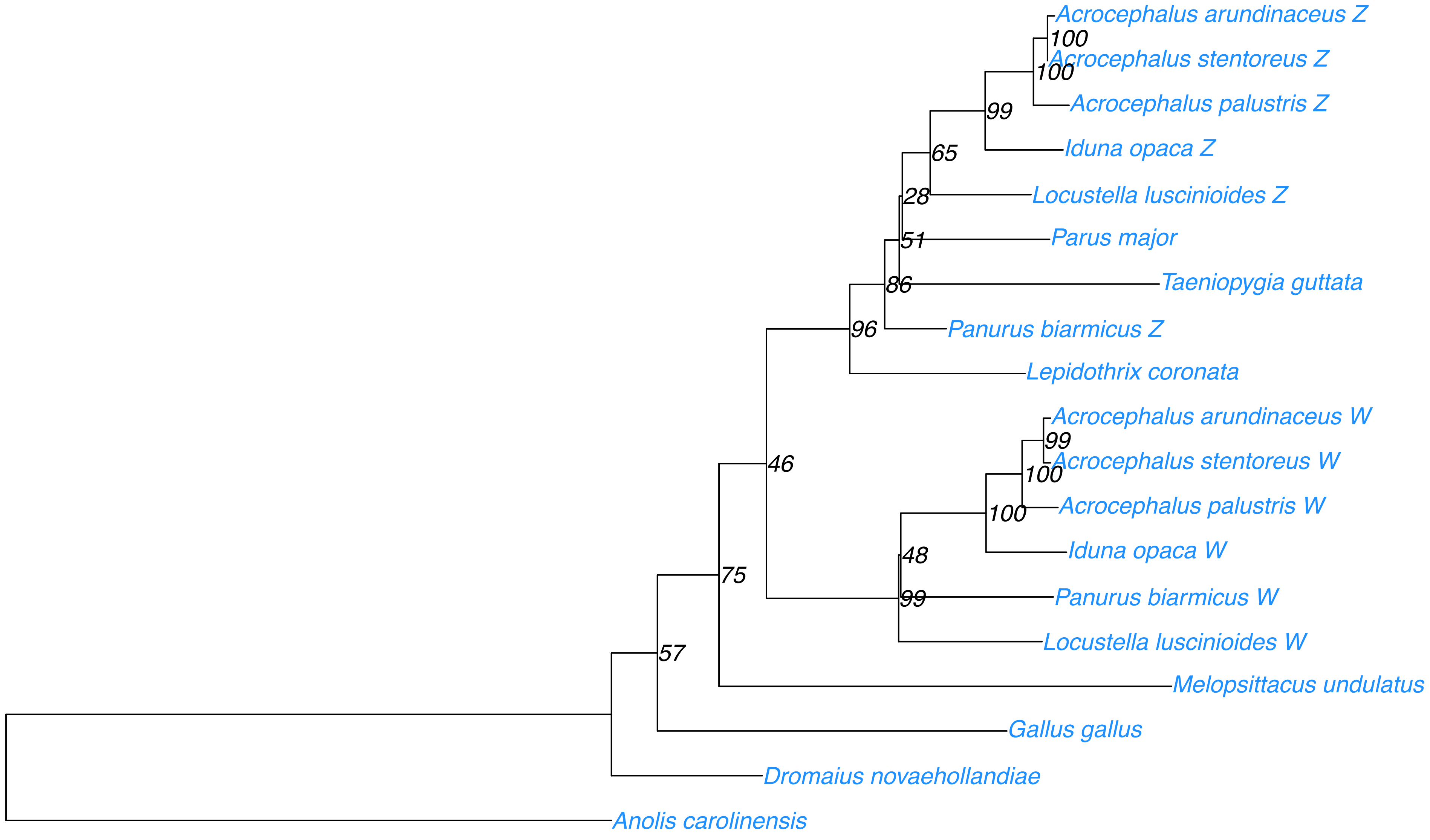

ENSTGUT00000002835

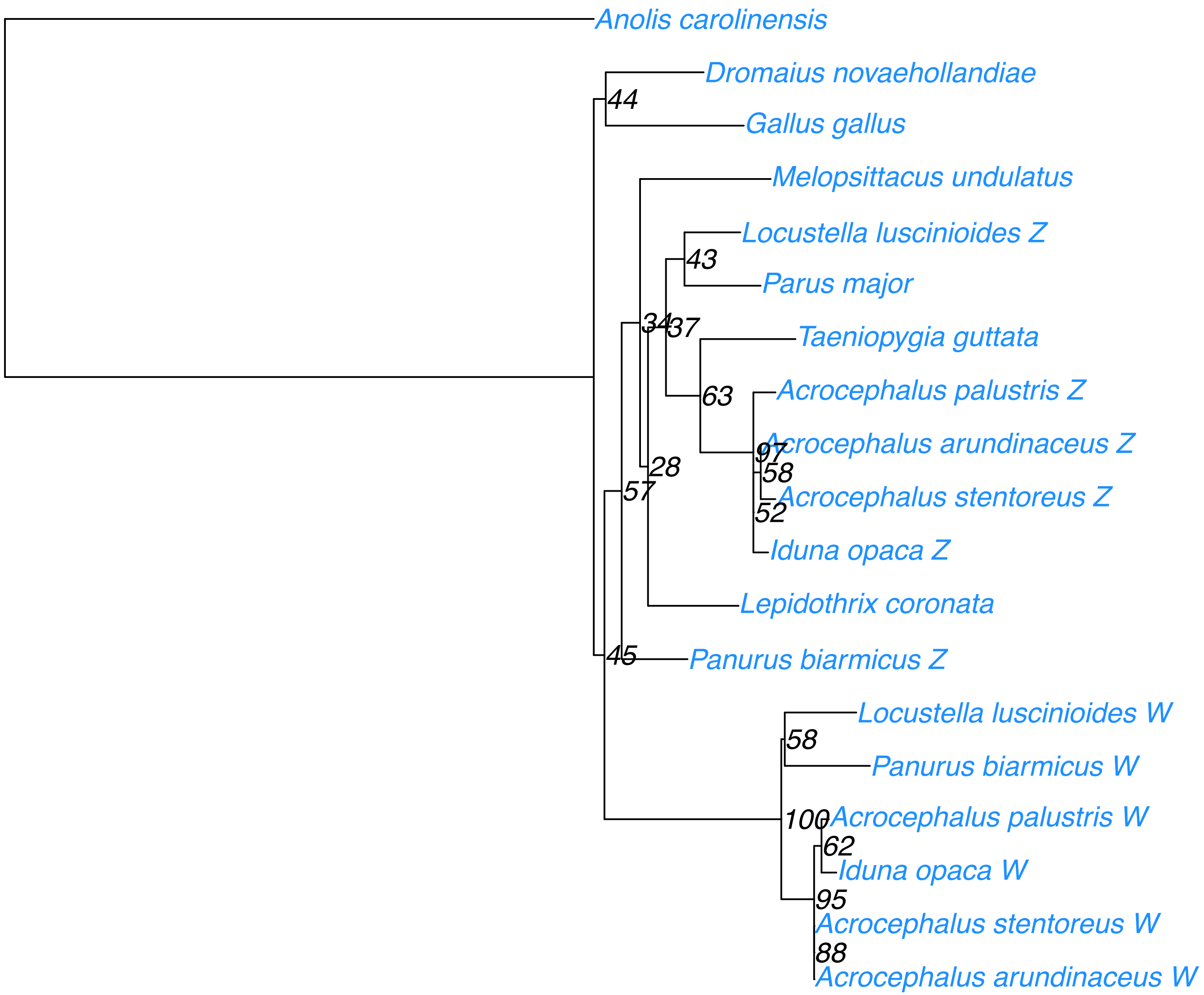

ENSTGUT00000002363

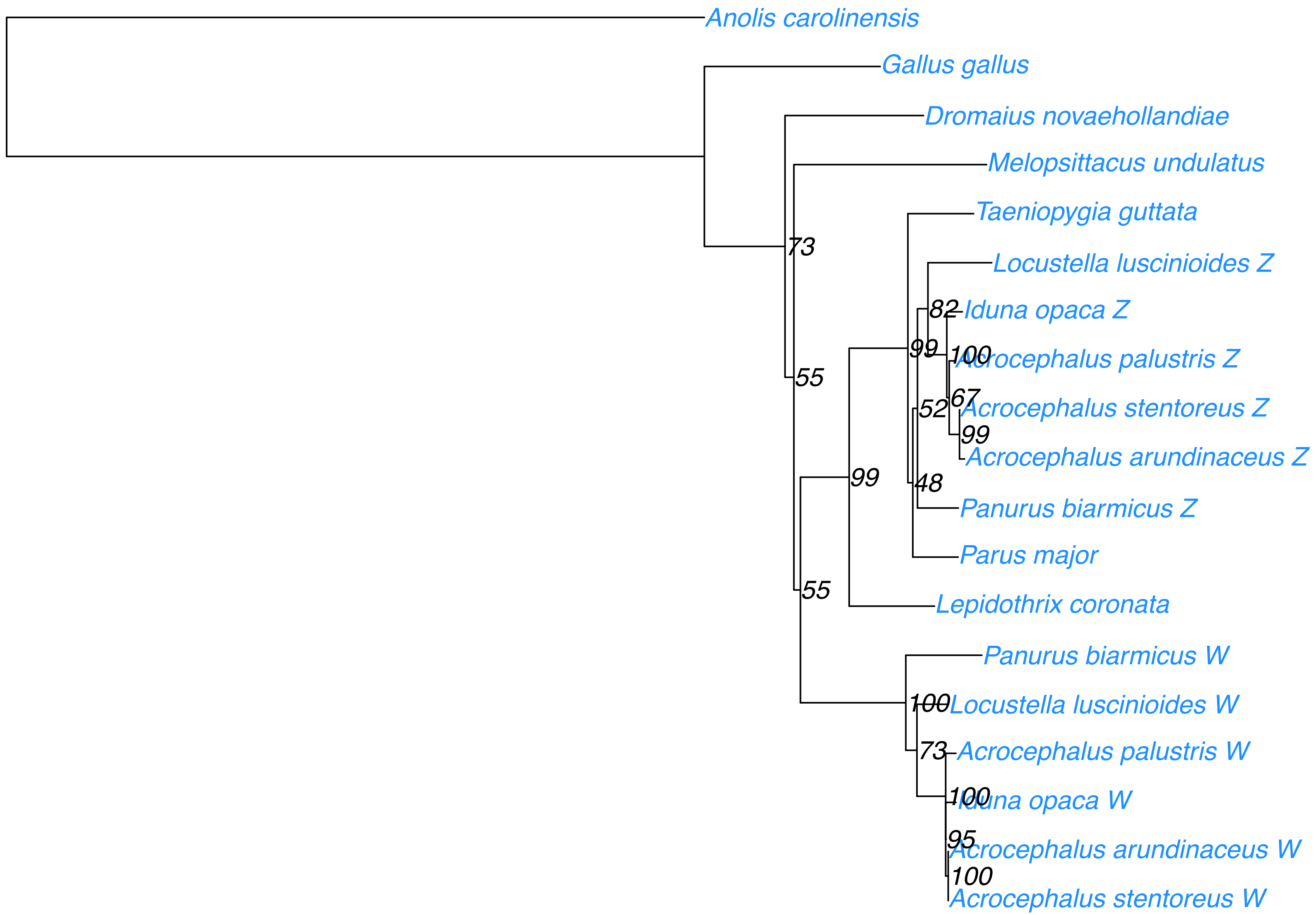

ENSTGUT00000002272

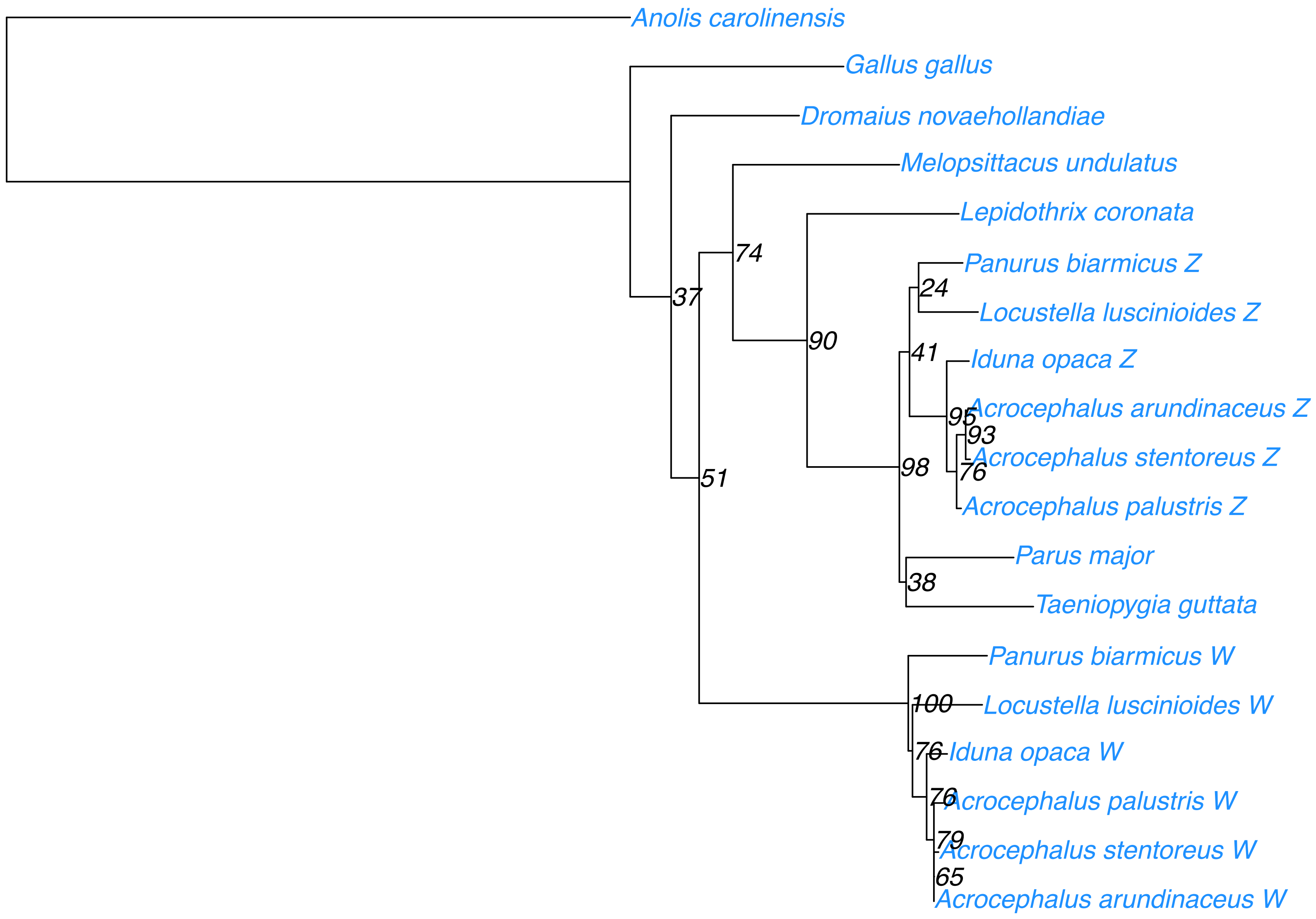

ENSTGUT00000001990

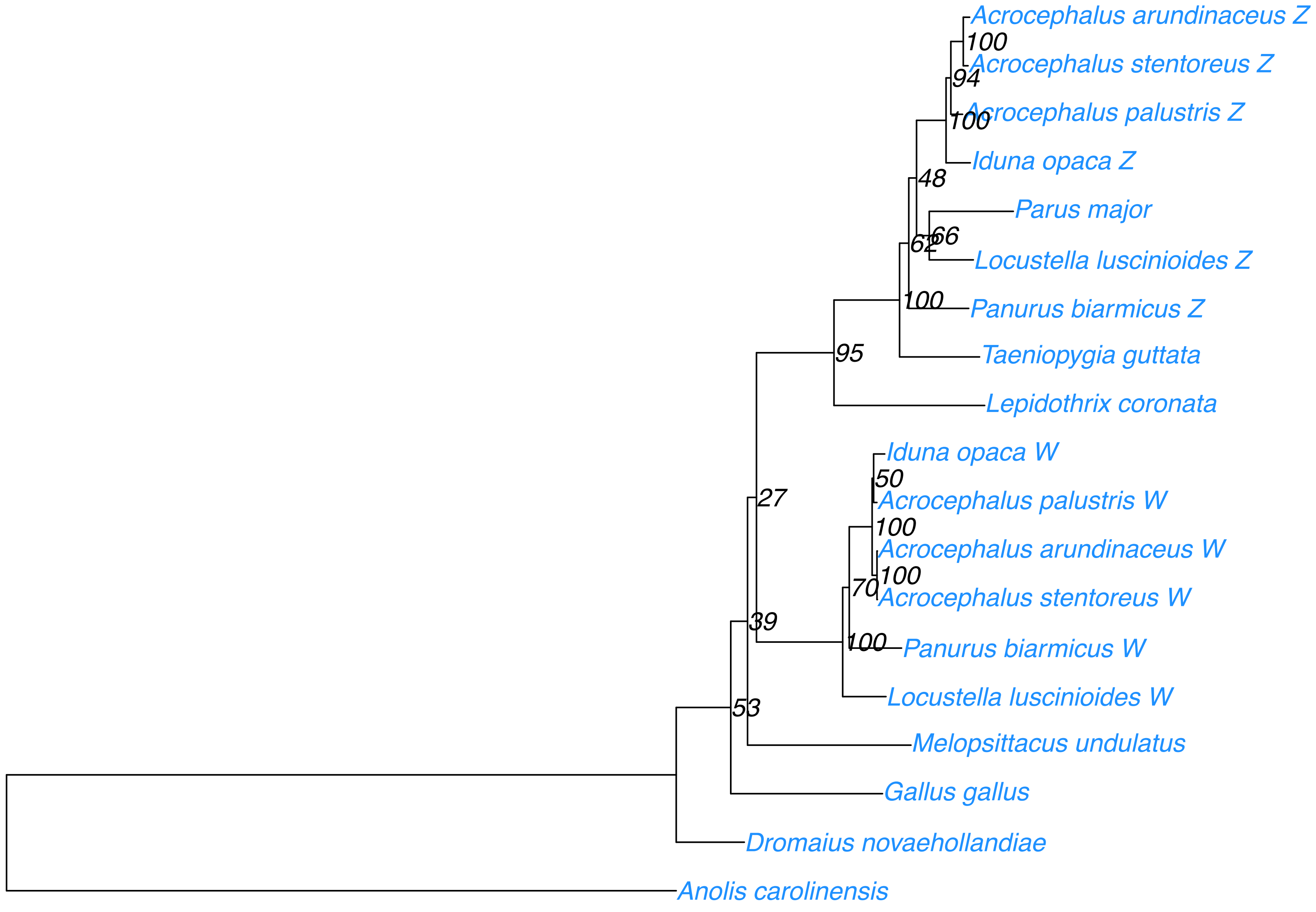

ENSTGUT00000001842

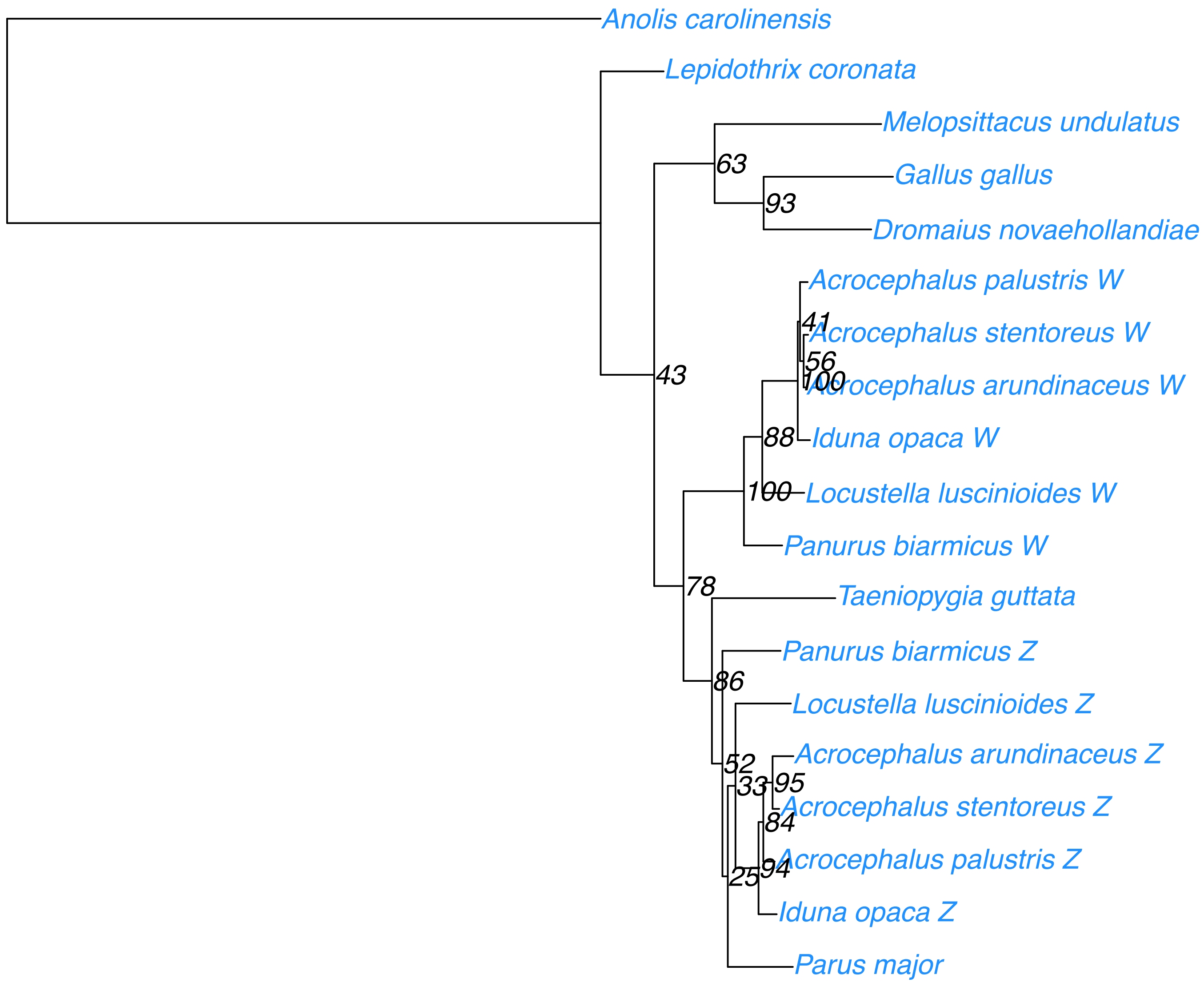

ENSTGUT00000001771

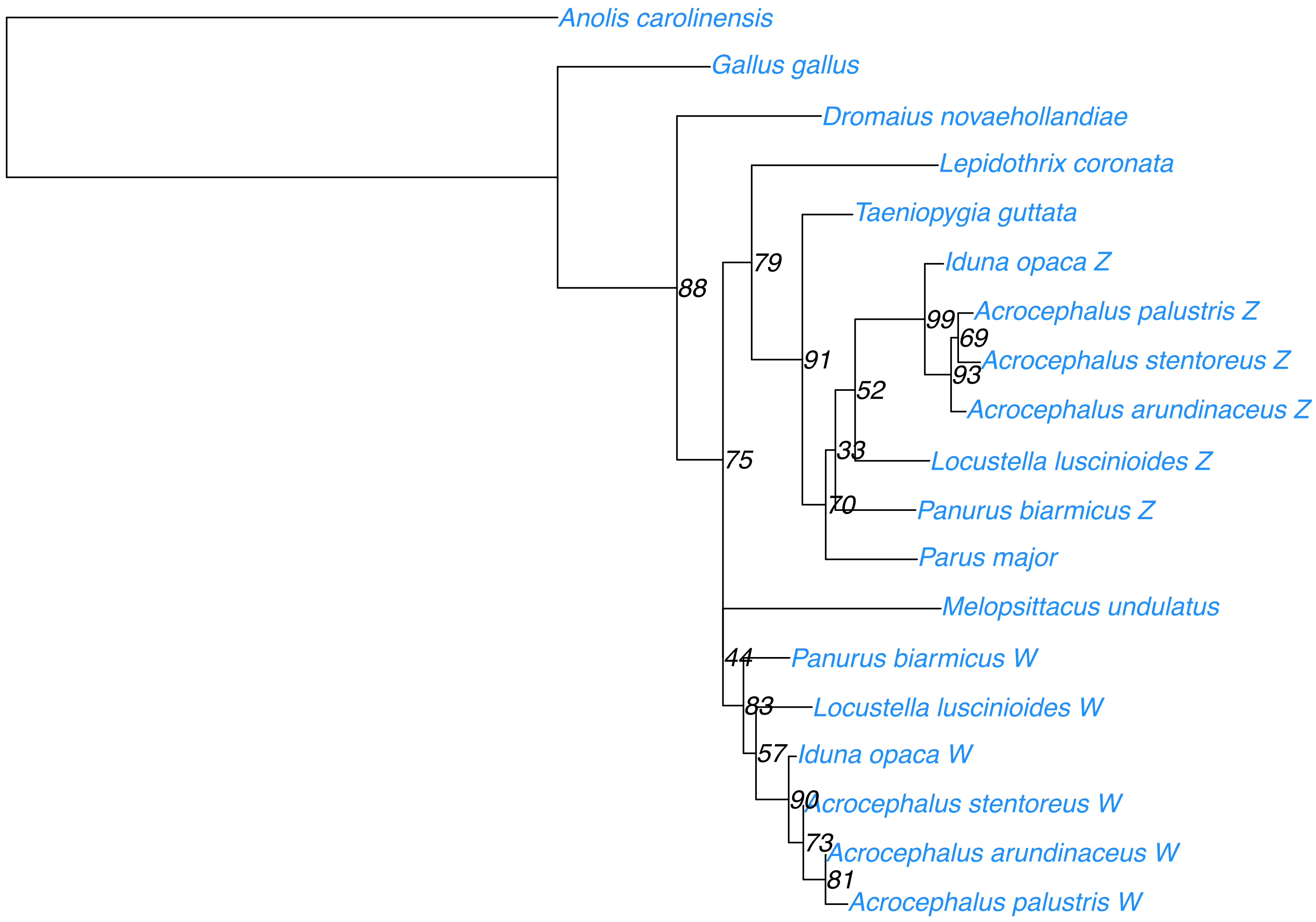

ENSTGUT00000001767

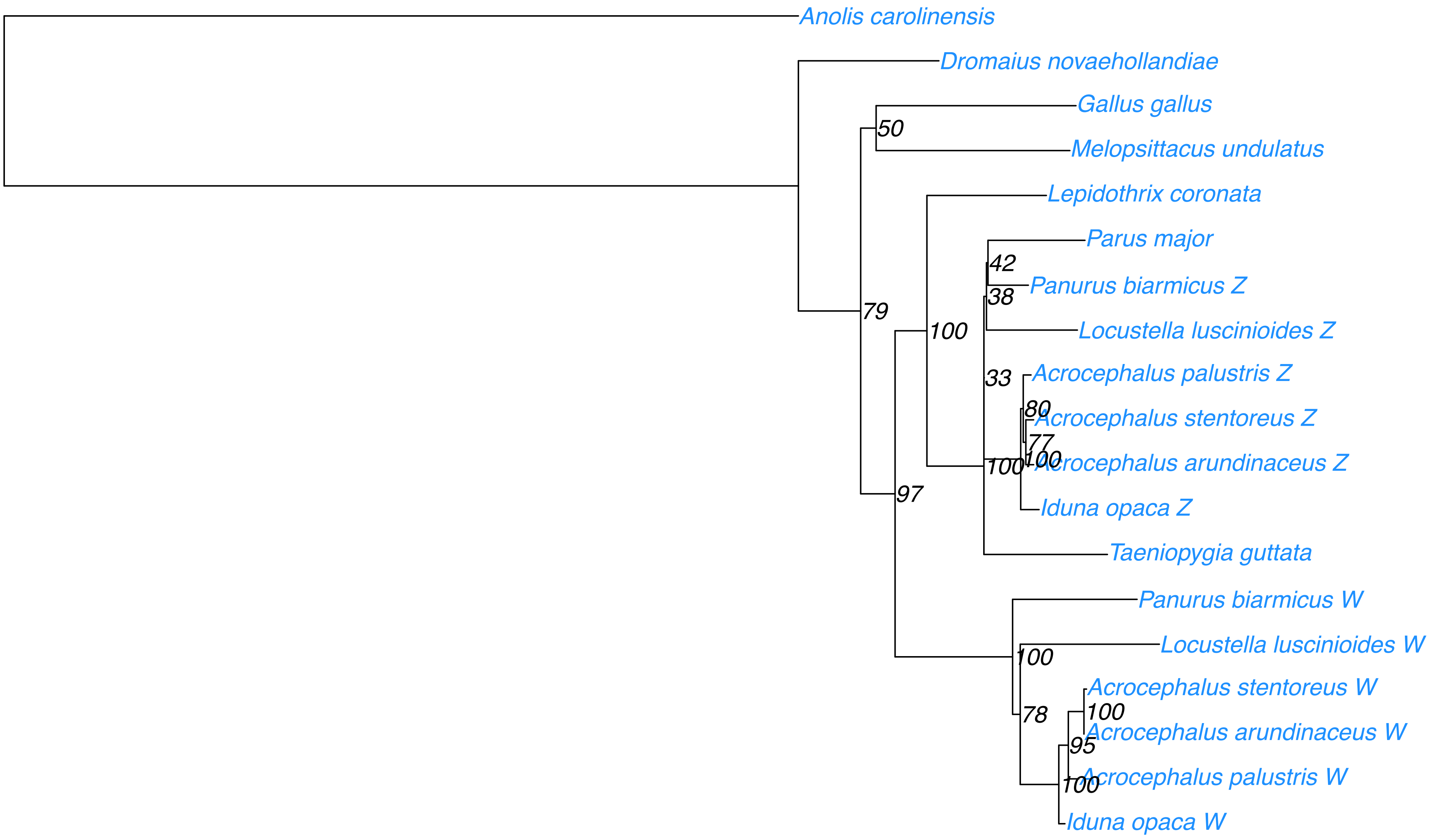

ENSTGUT00000001647

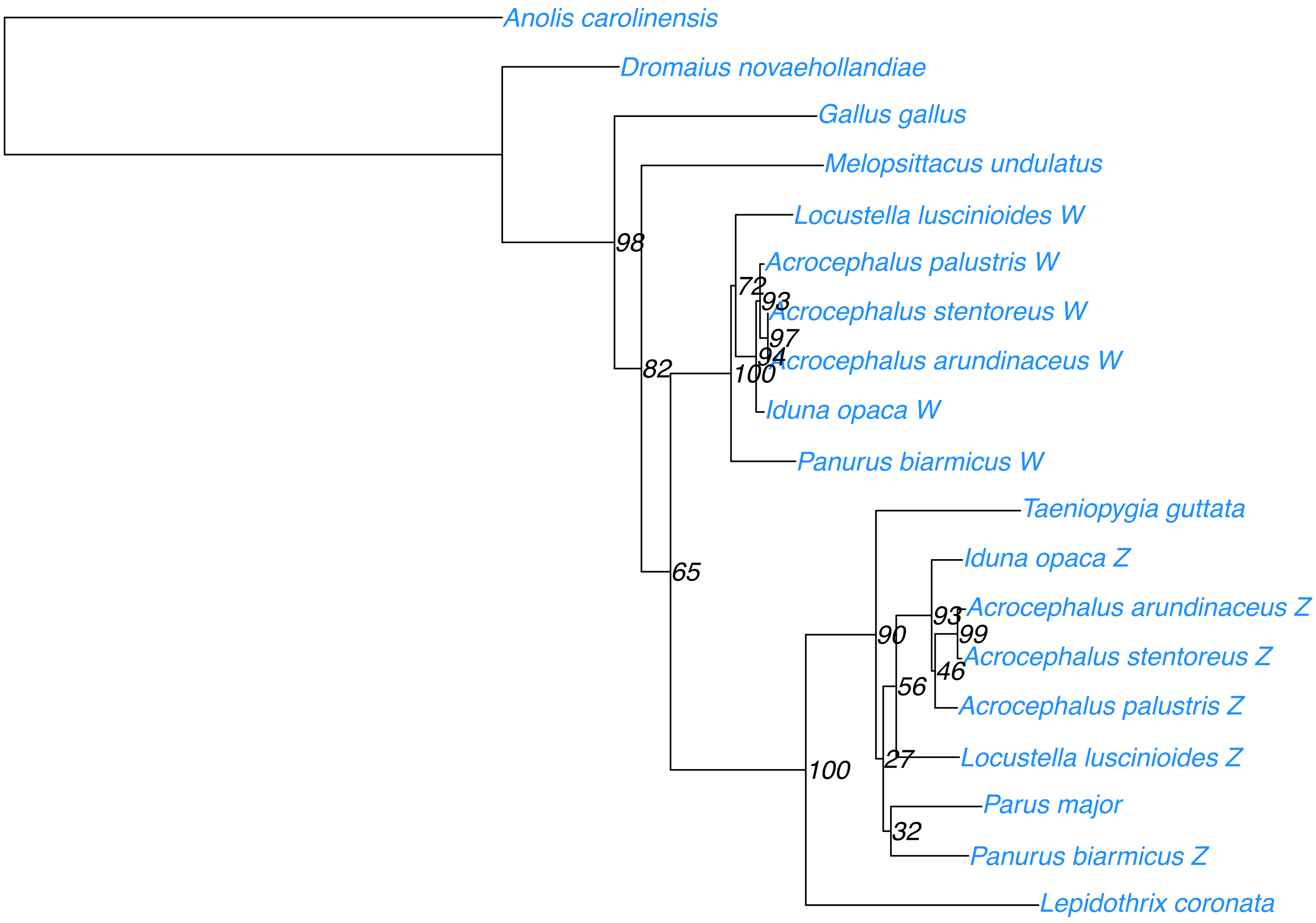

ENSTGUT00000003277

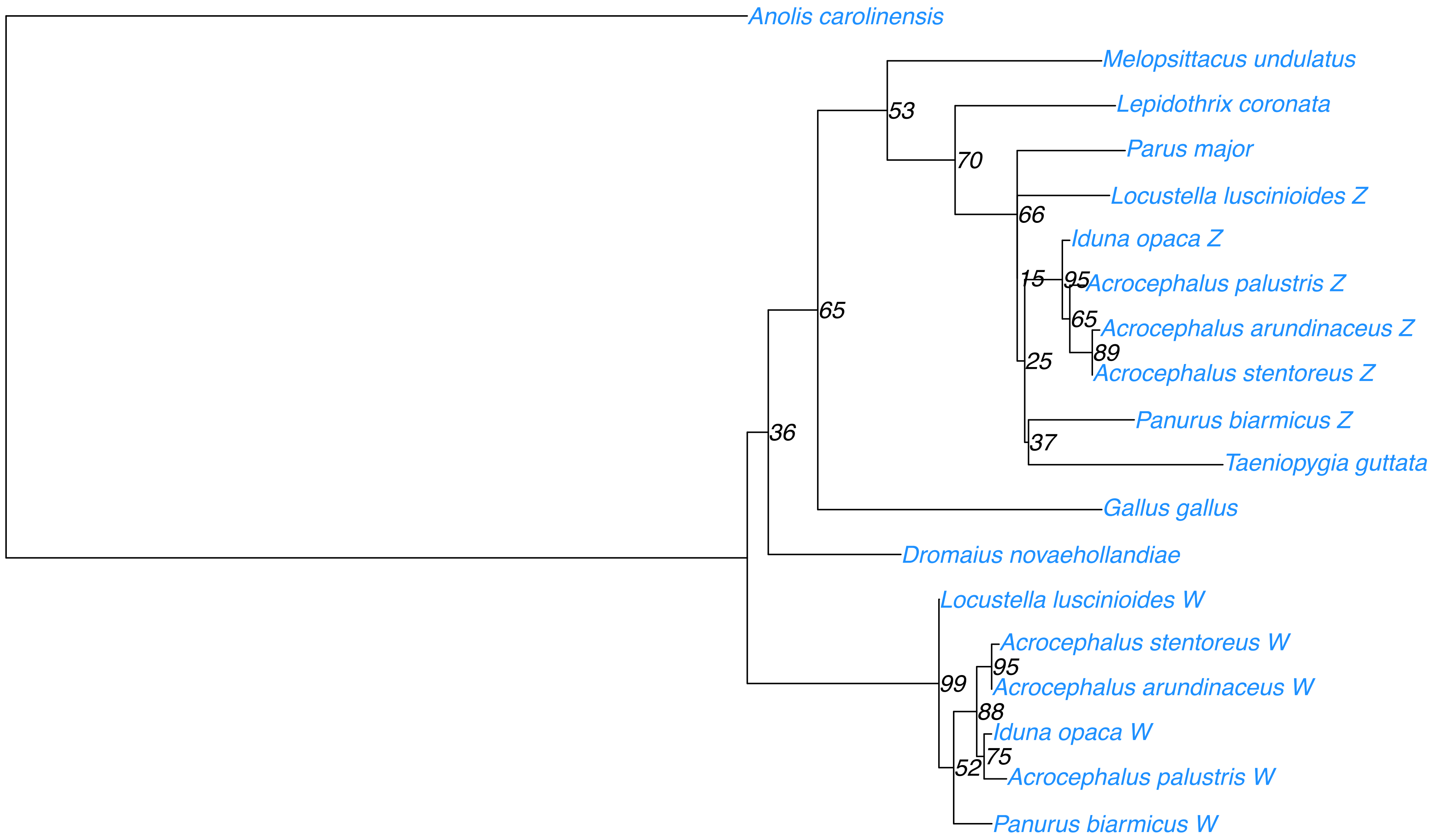

ENSTGUT00000000482

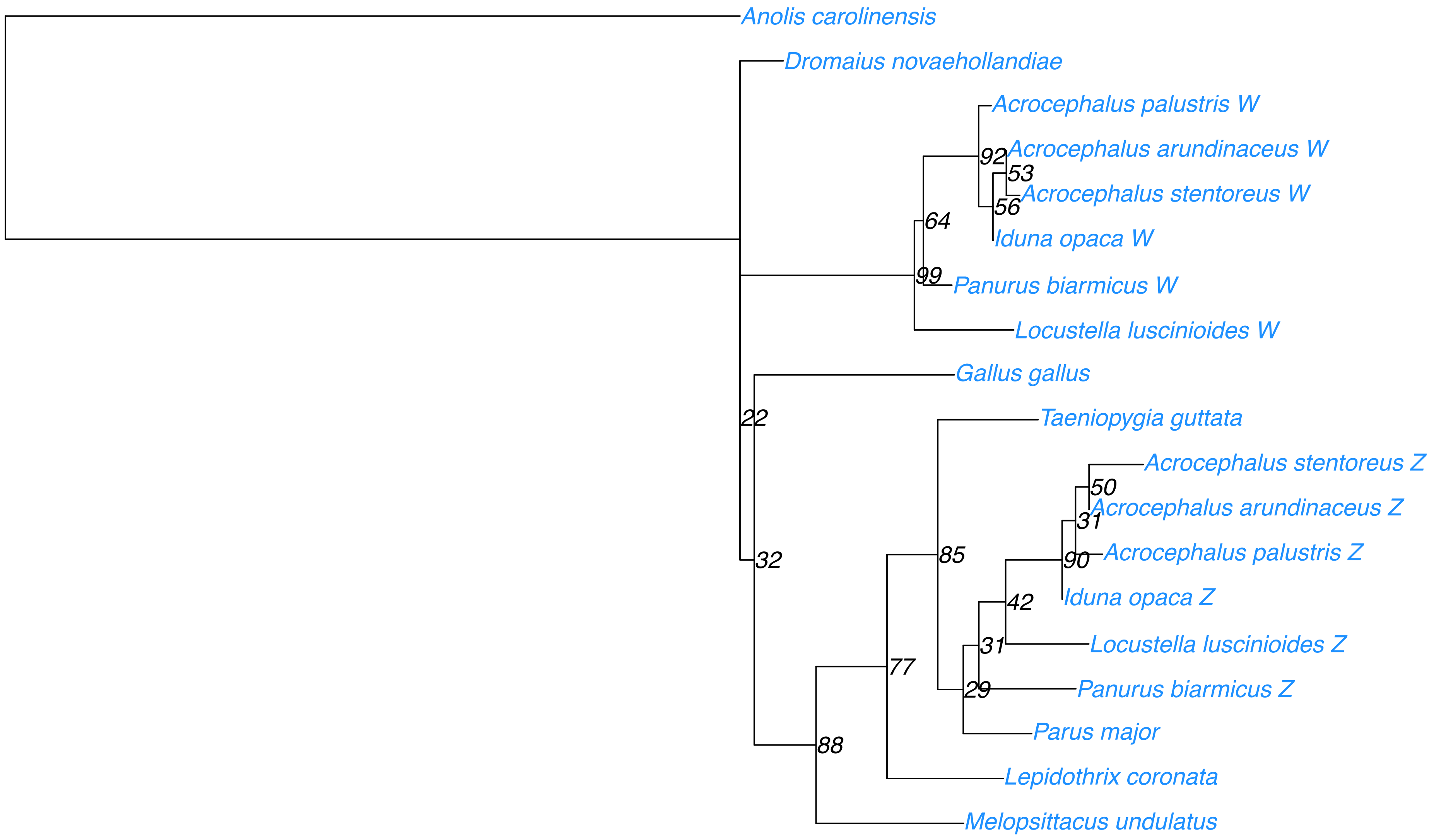

ENSTGUT00000000280

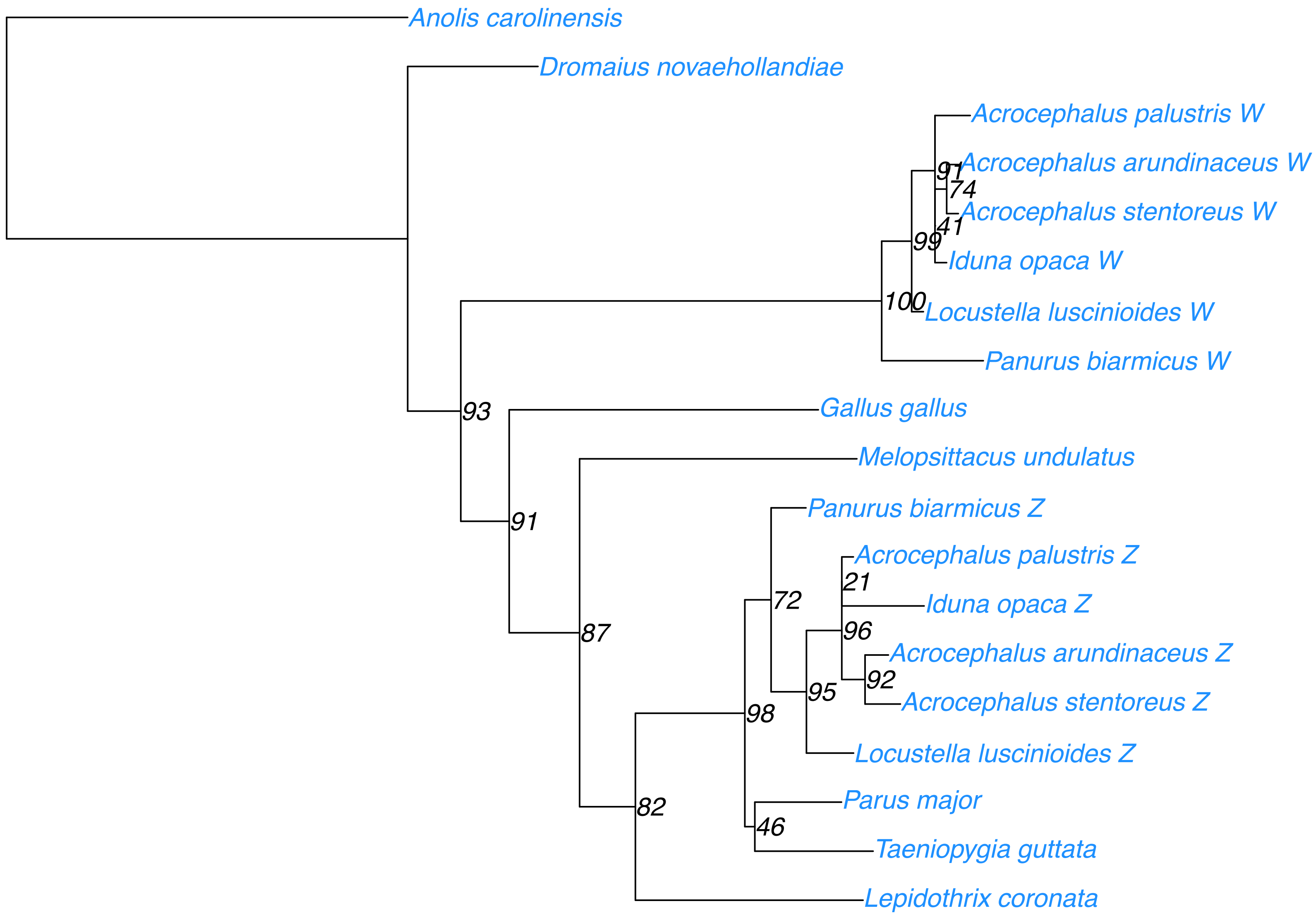

ENSTGUT00000001229

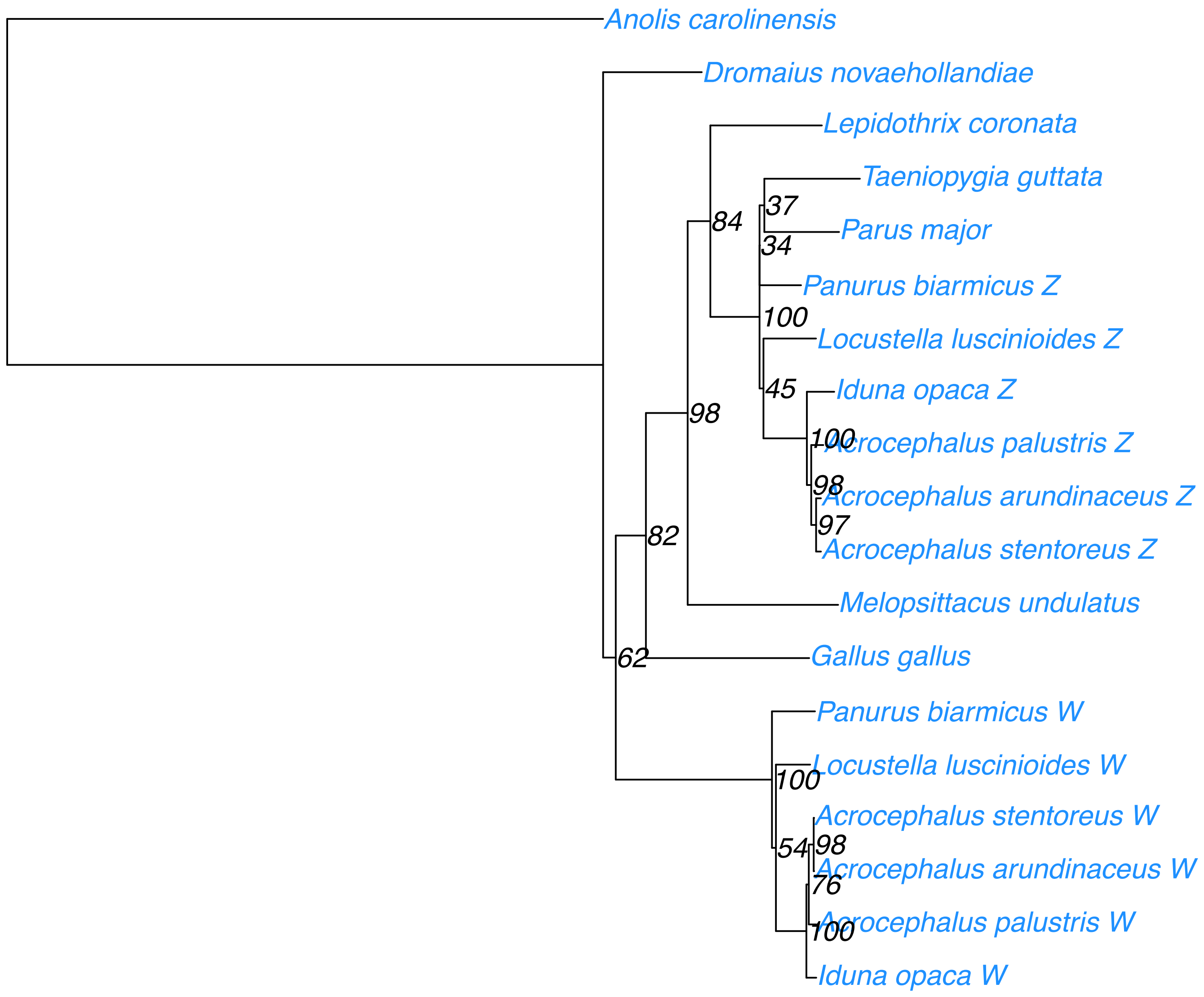

ENSTGUT00000001271

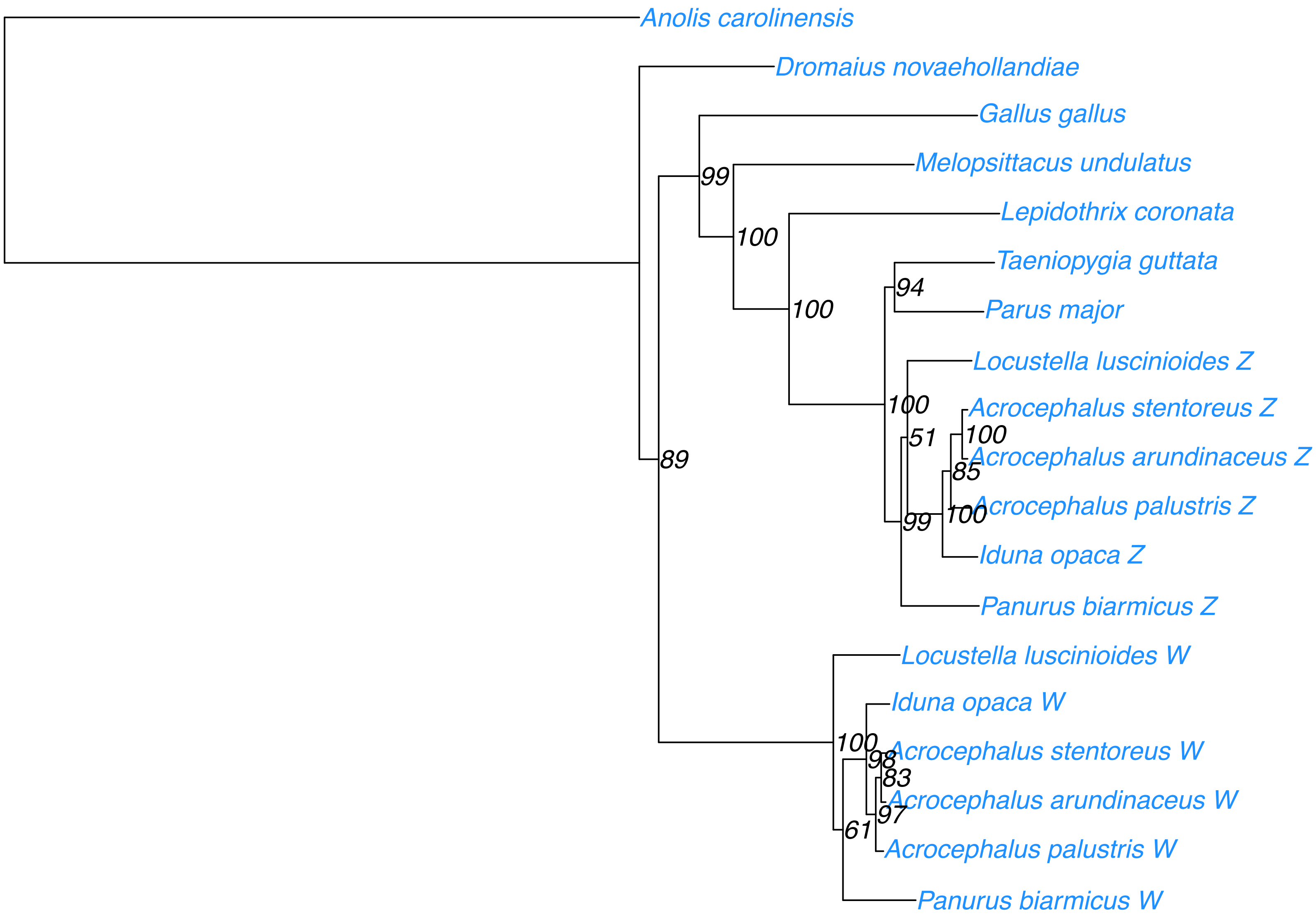

ENSTGUT00000003698

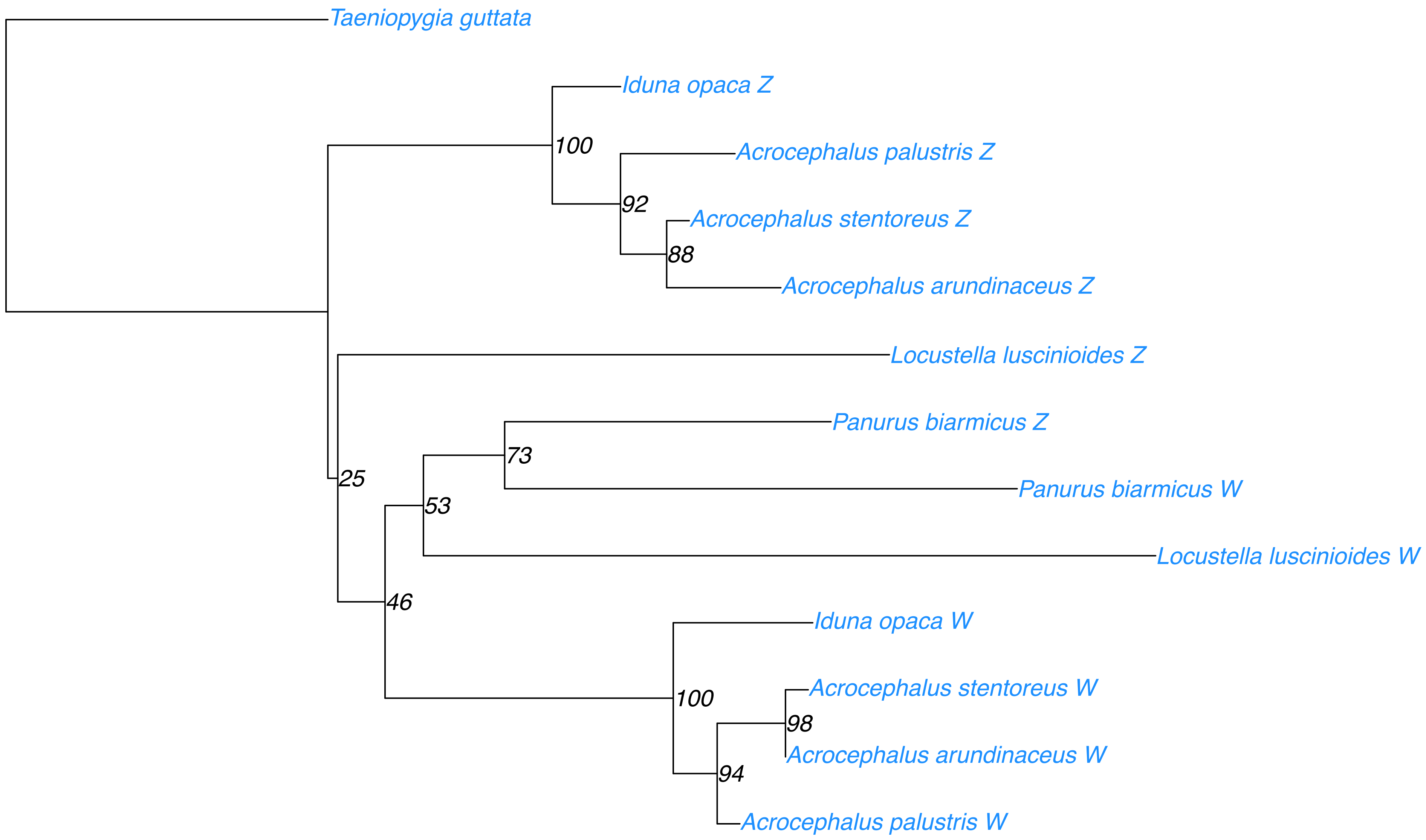

ENSTGUT00000003642

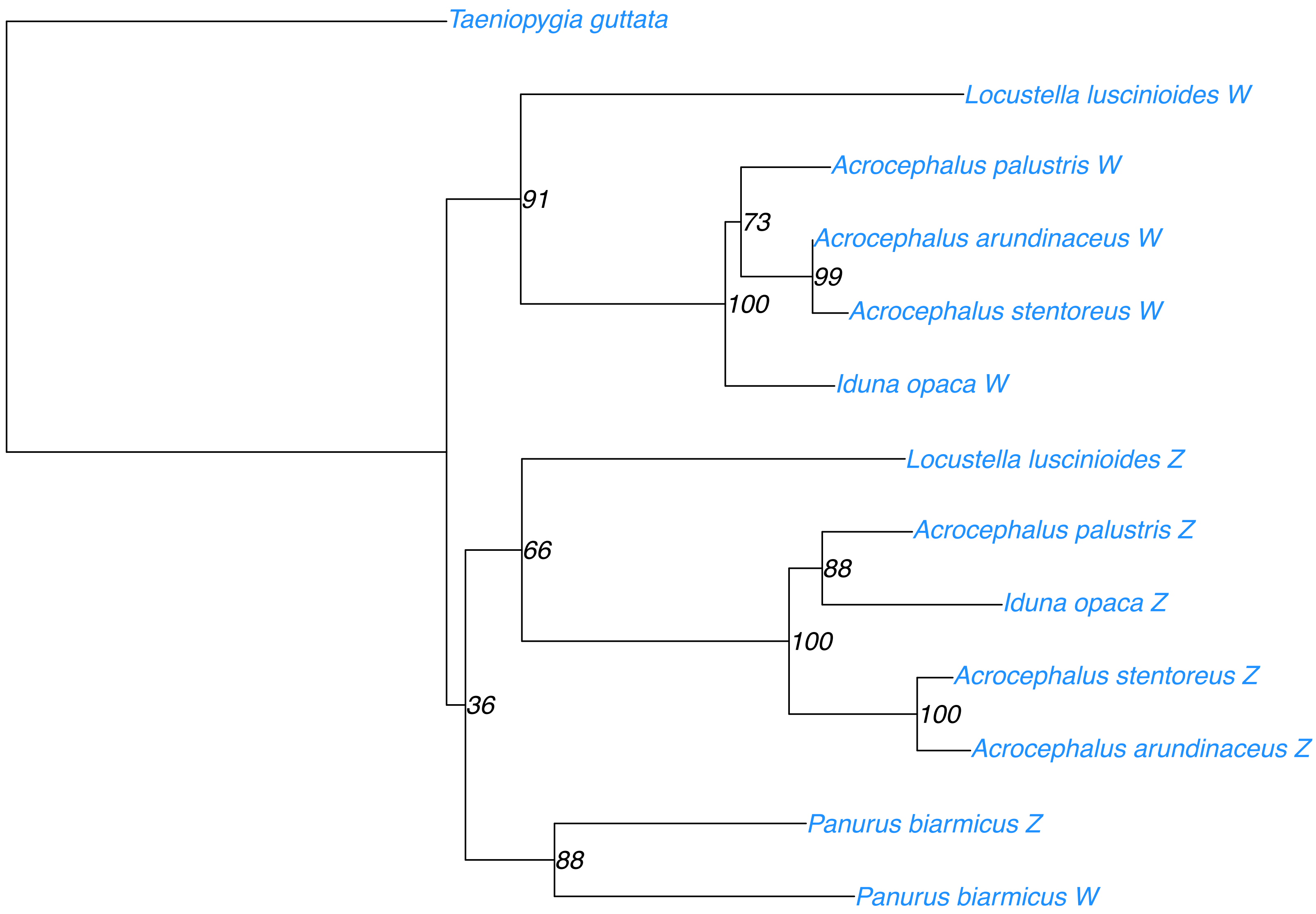

ENSTGUT00000003593

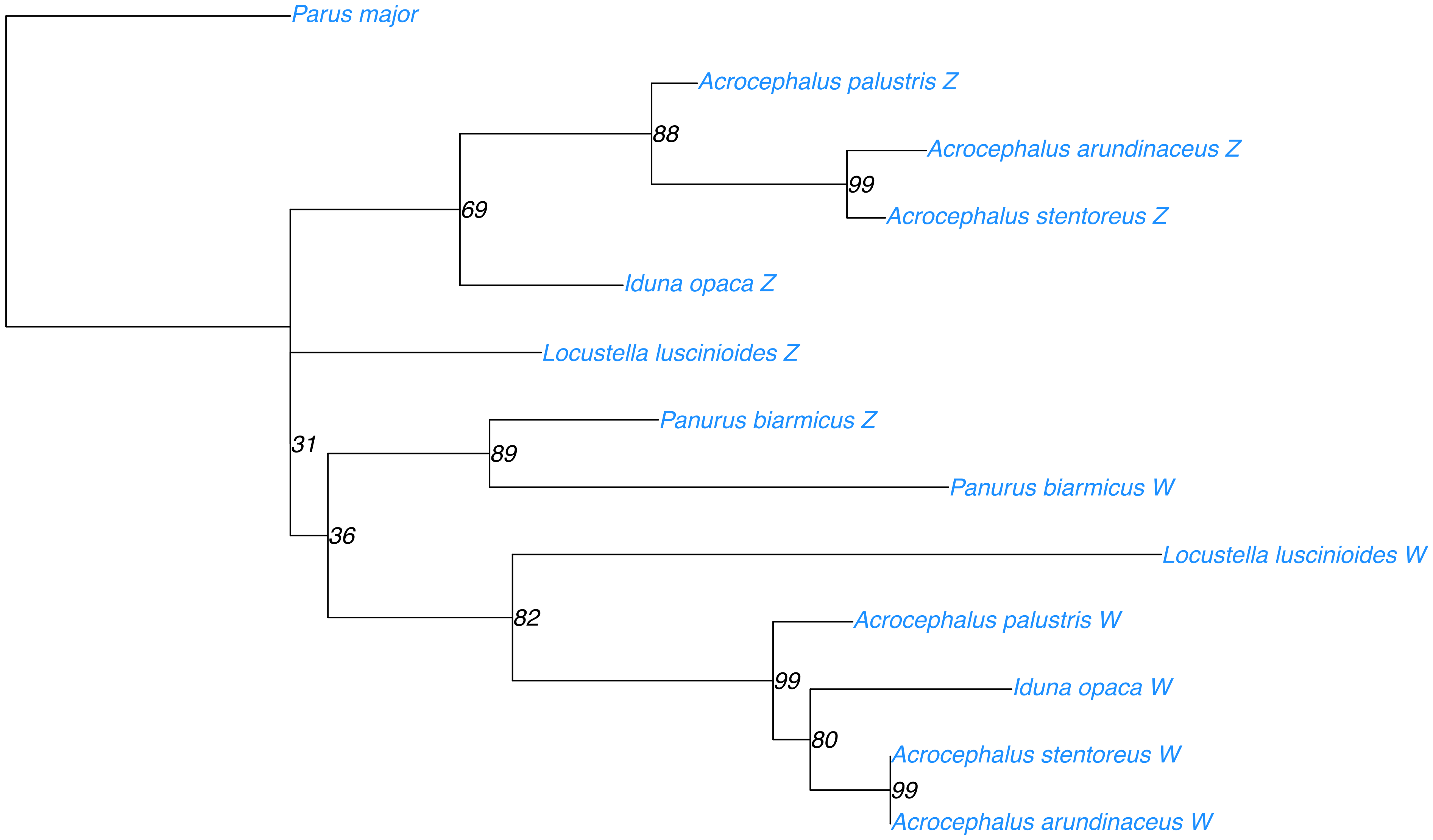

ENSTGUT00000018848

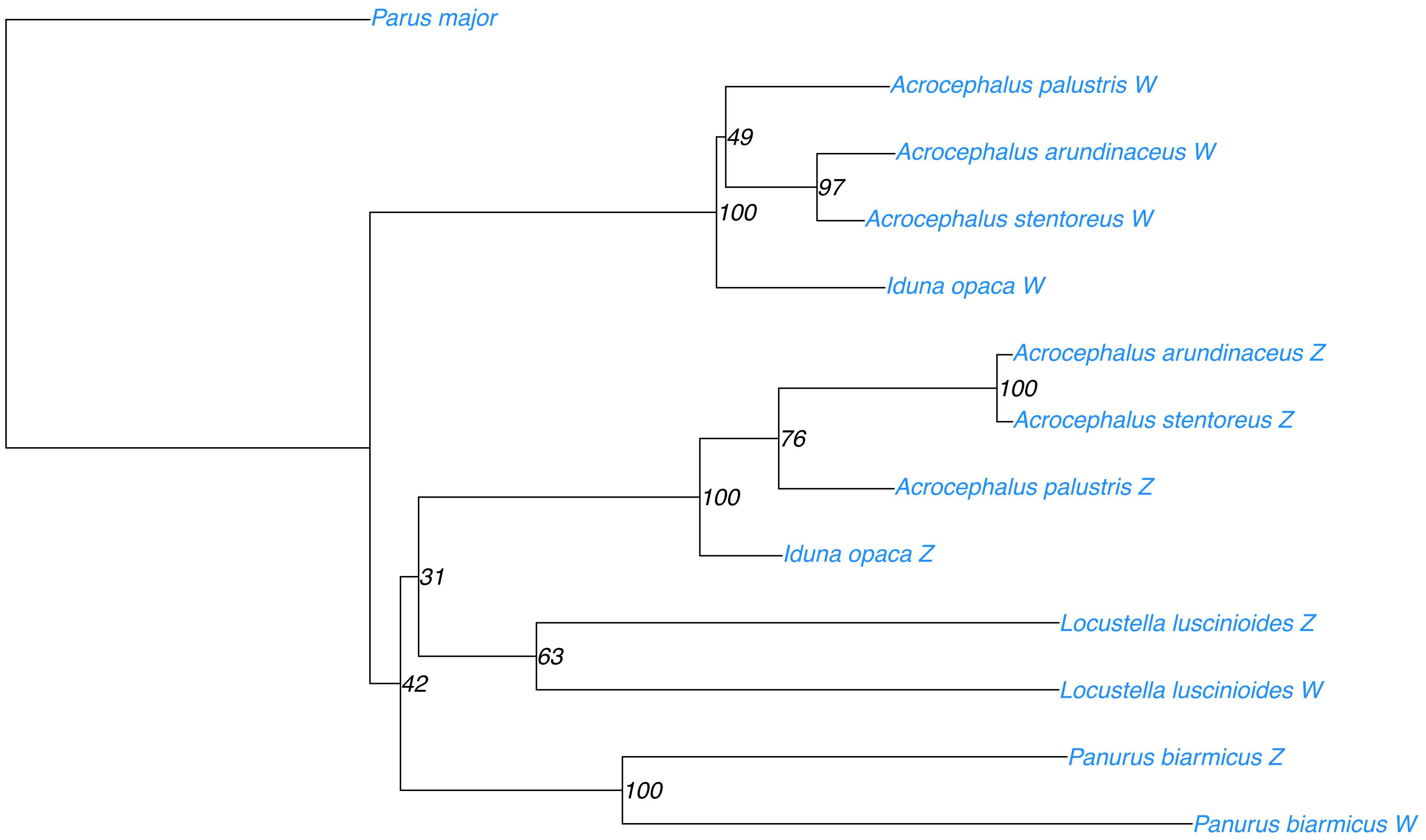

ENSTGUT00000013114

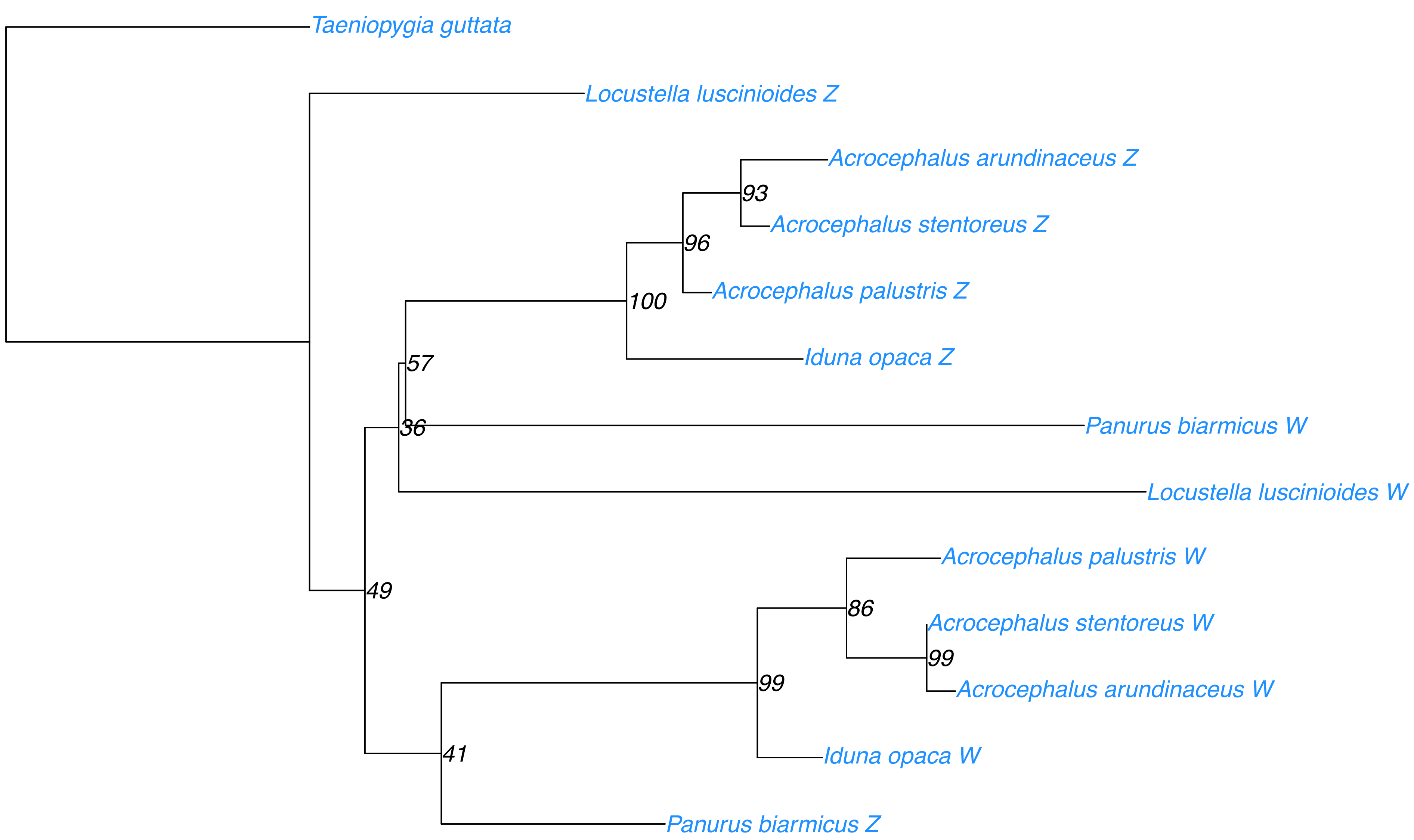

ENSTGUT00000003566

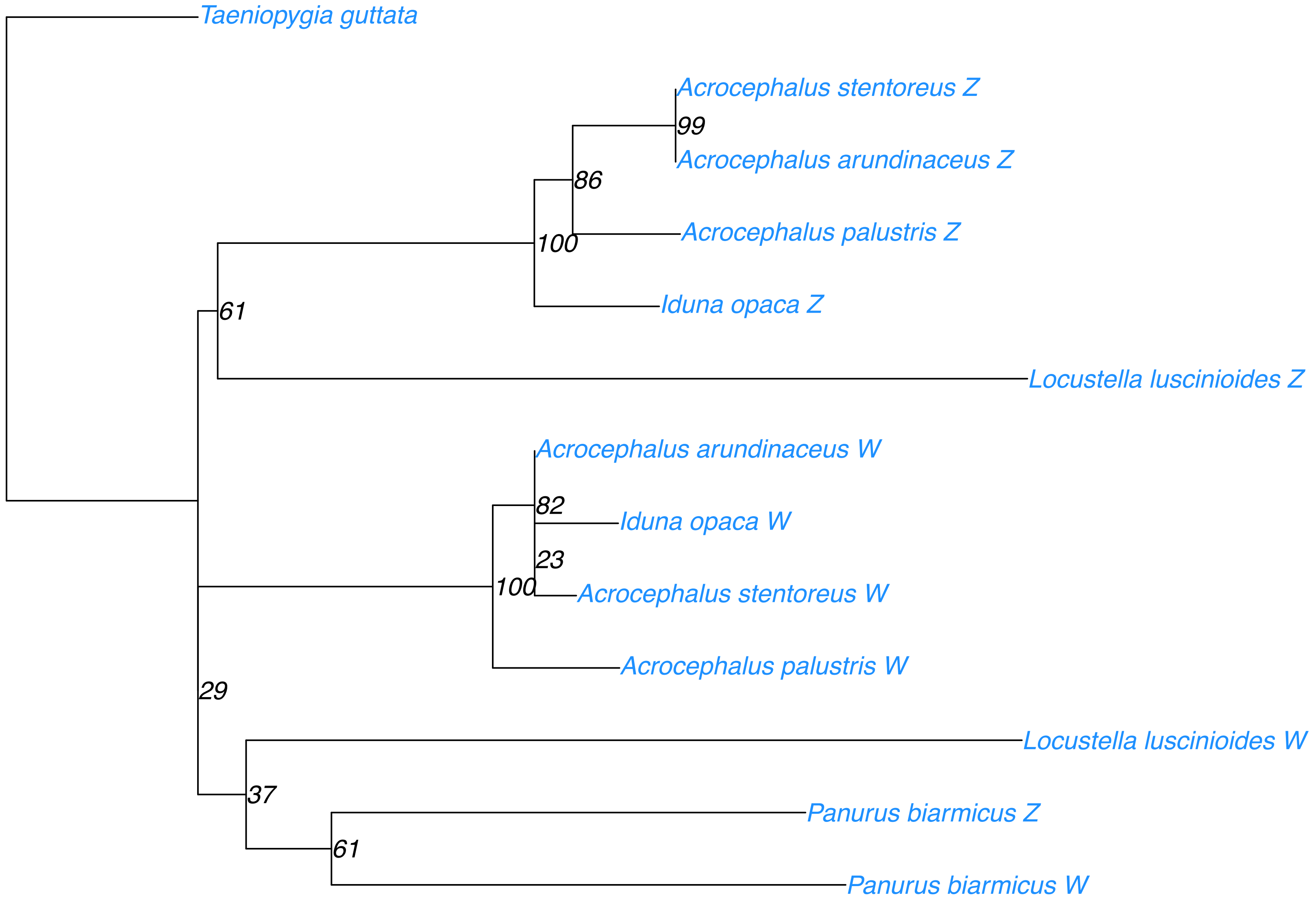

ENSTGUT00000003543

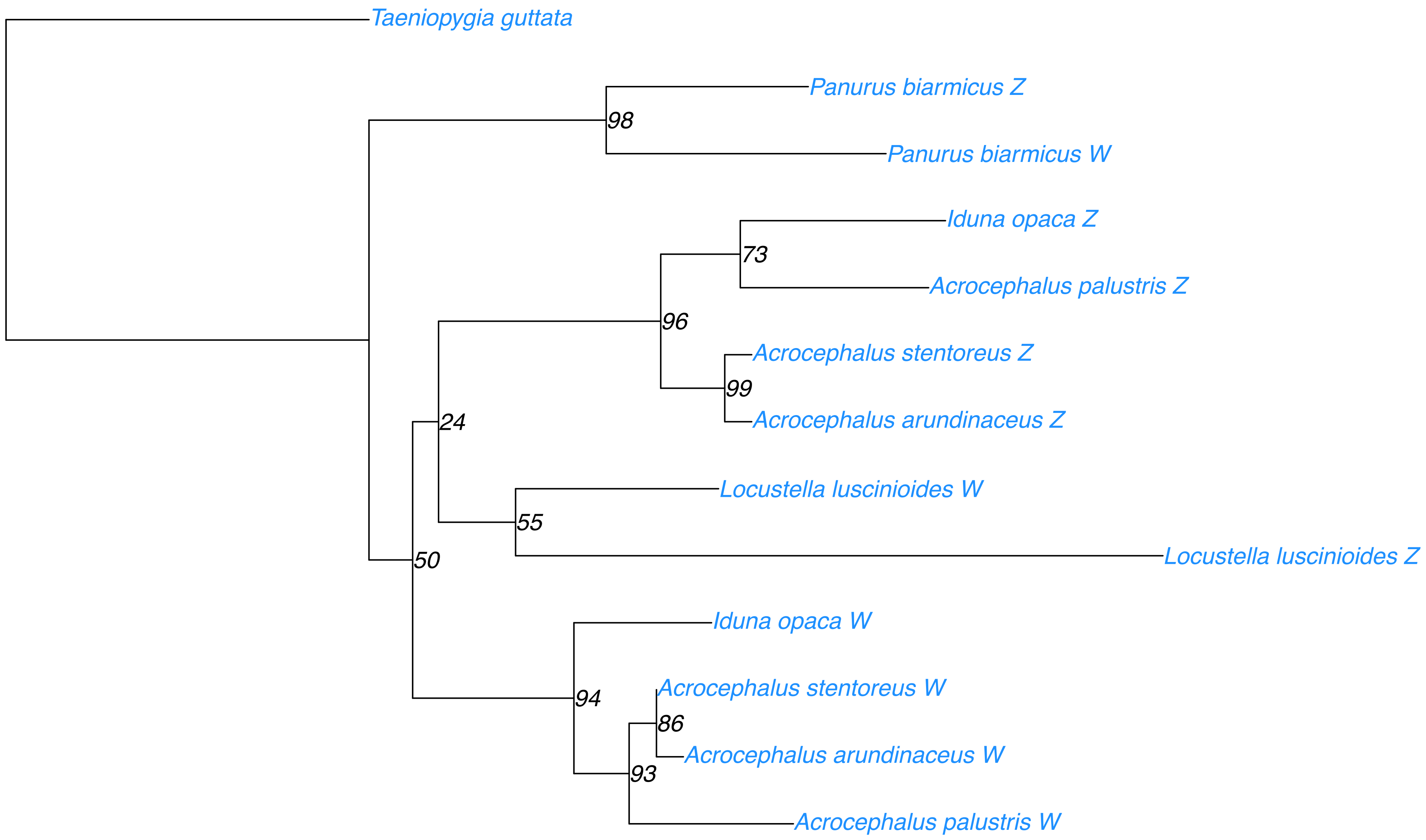

ENSTGUT00000003492

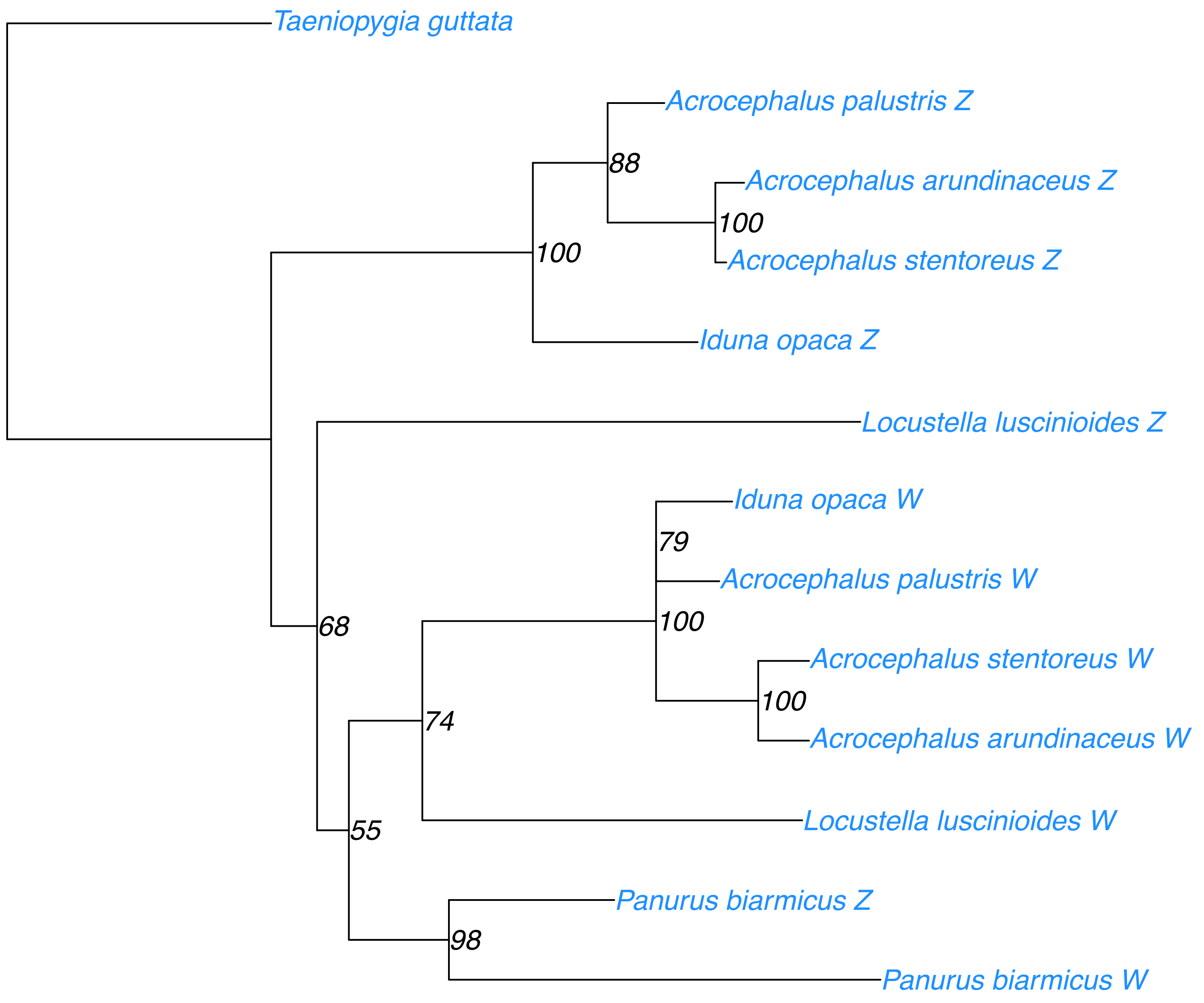

ENSTGUT00000003441

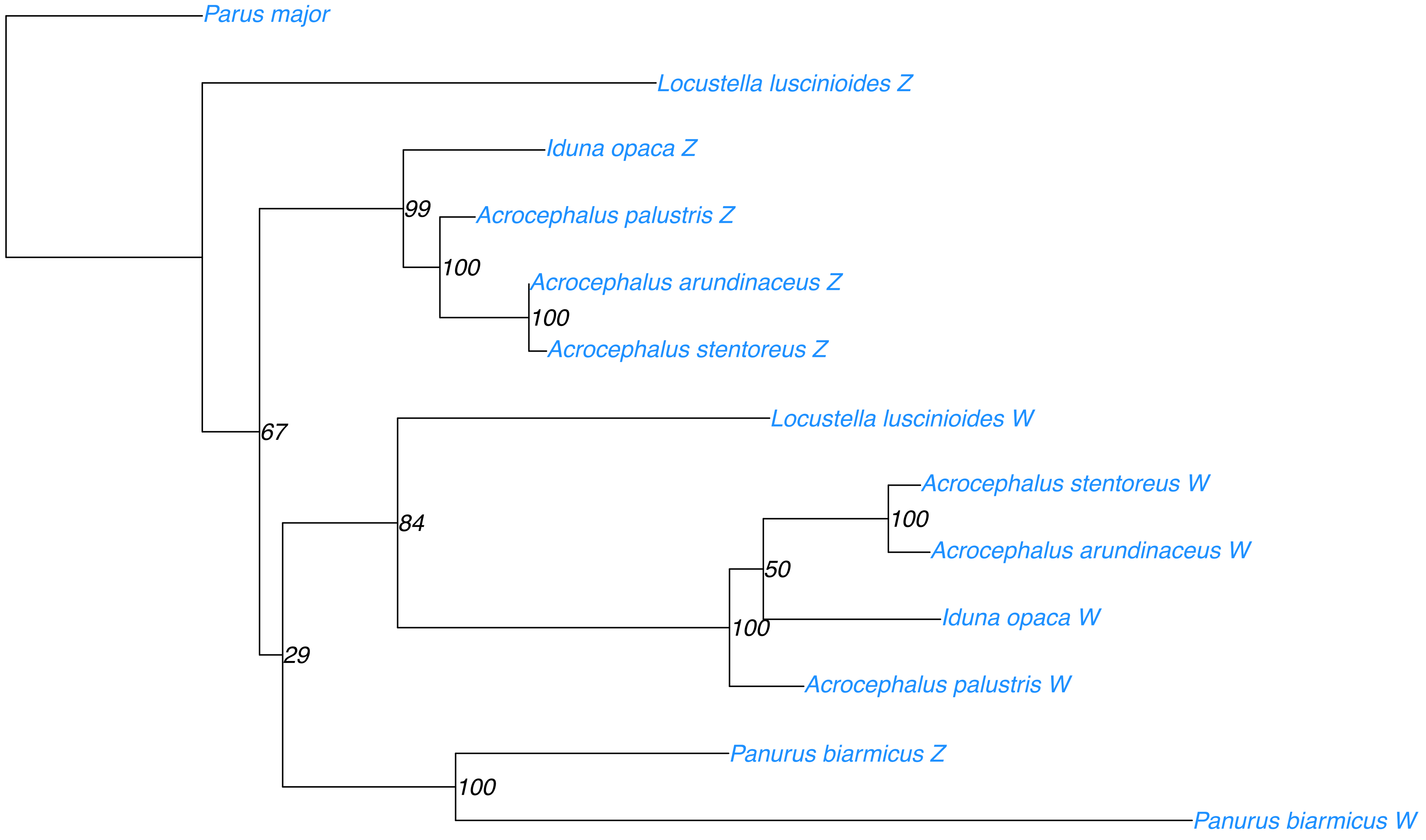

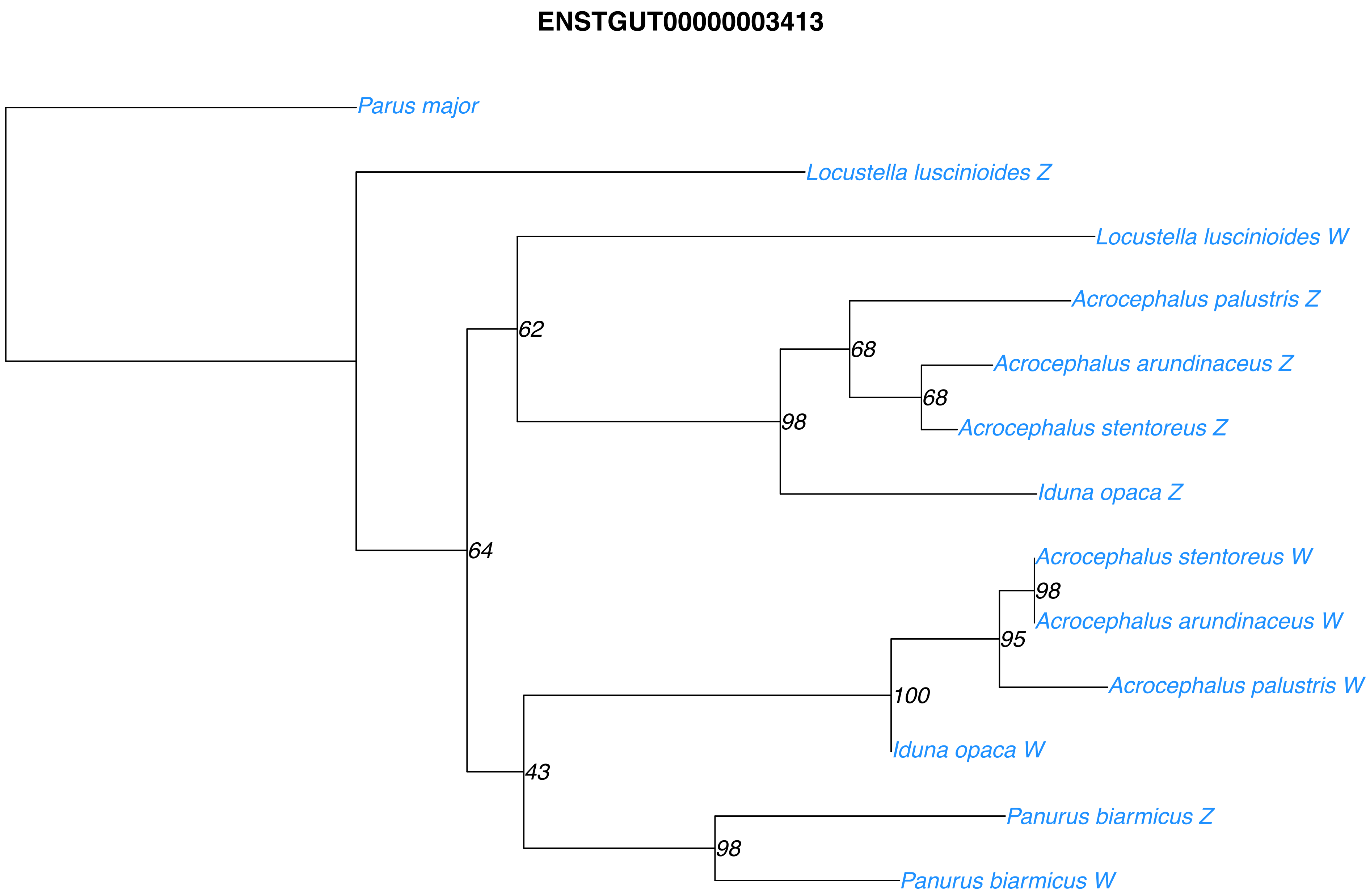
ENSTGUT00000003413
Parus major
Locustella luscinioides Z
Locustella luscinioides W
Acrocephalus palustris Z
Acrocephalus arundinaceus Z
Acrocephalus stentoreus Z
Iduna opaca Z
Acrocephalus stentoreus W
Acrocephalus arundinaceus W
Acrocephalus palustris W
Iduna opaca W
Panurus biarmicus Z
Panurus biarmicus W
62
68
68
98
64
98
95
100
43
98

ENSTGUT00000003284

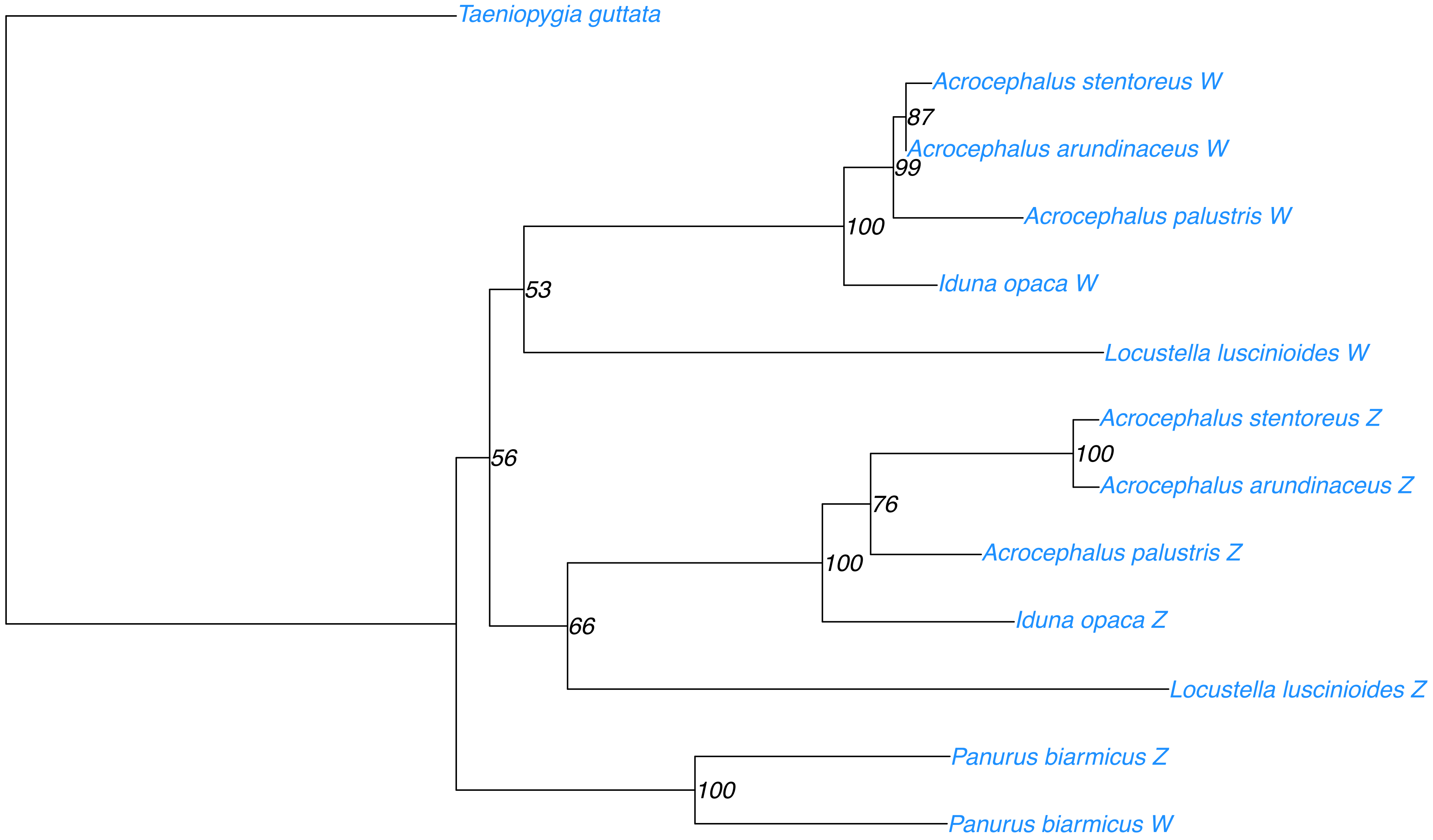

ENSTGUT00000003261

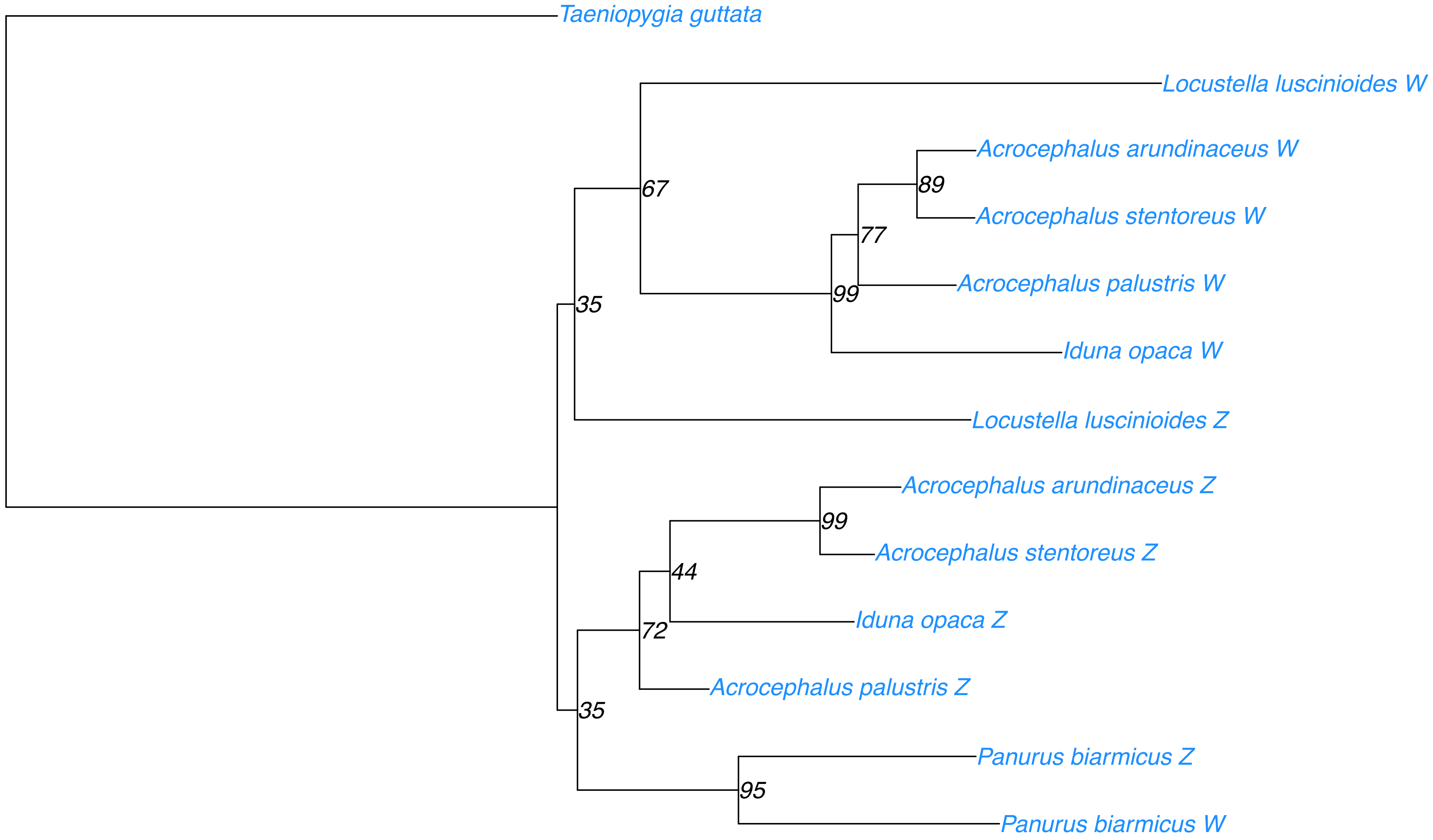

ENSTGUT00000003212

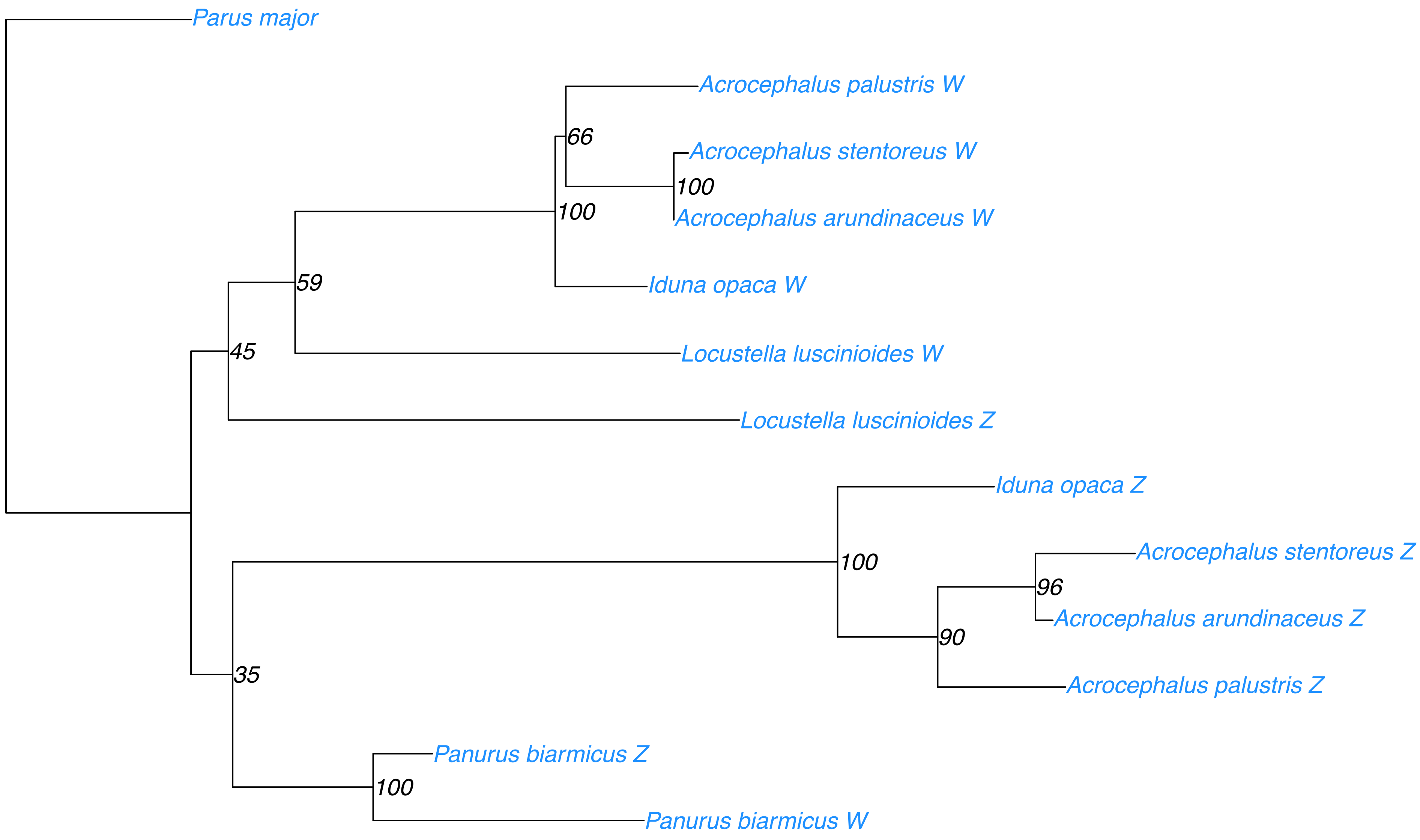

ENSTGUT00000003168

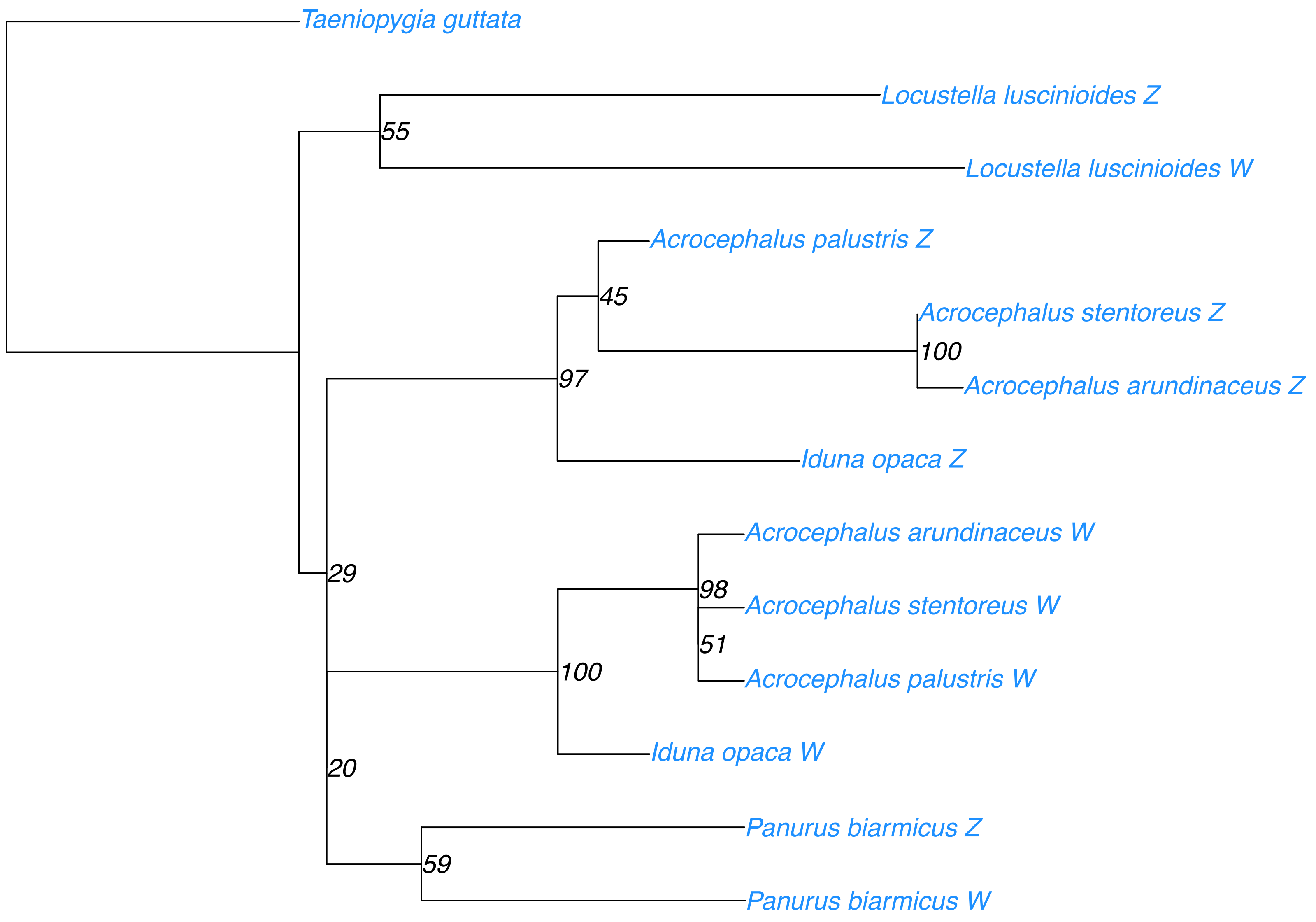

ENSTGUT00000003136

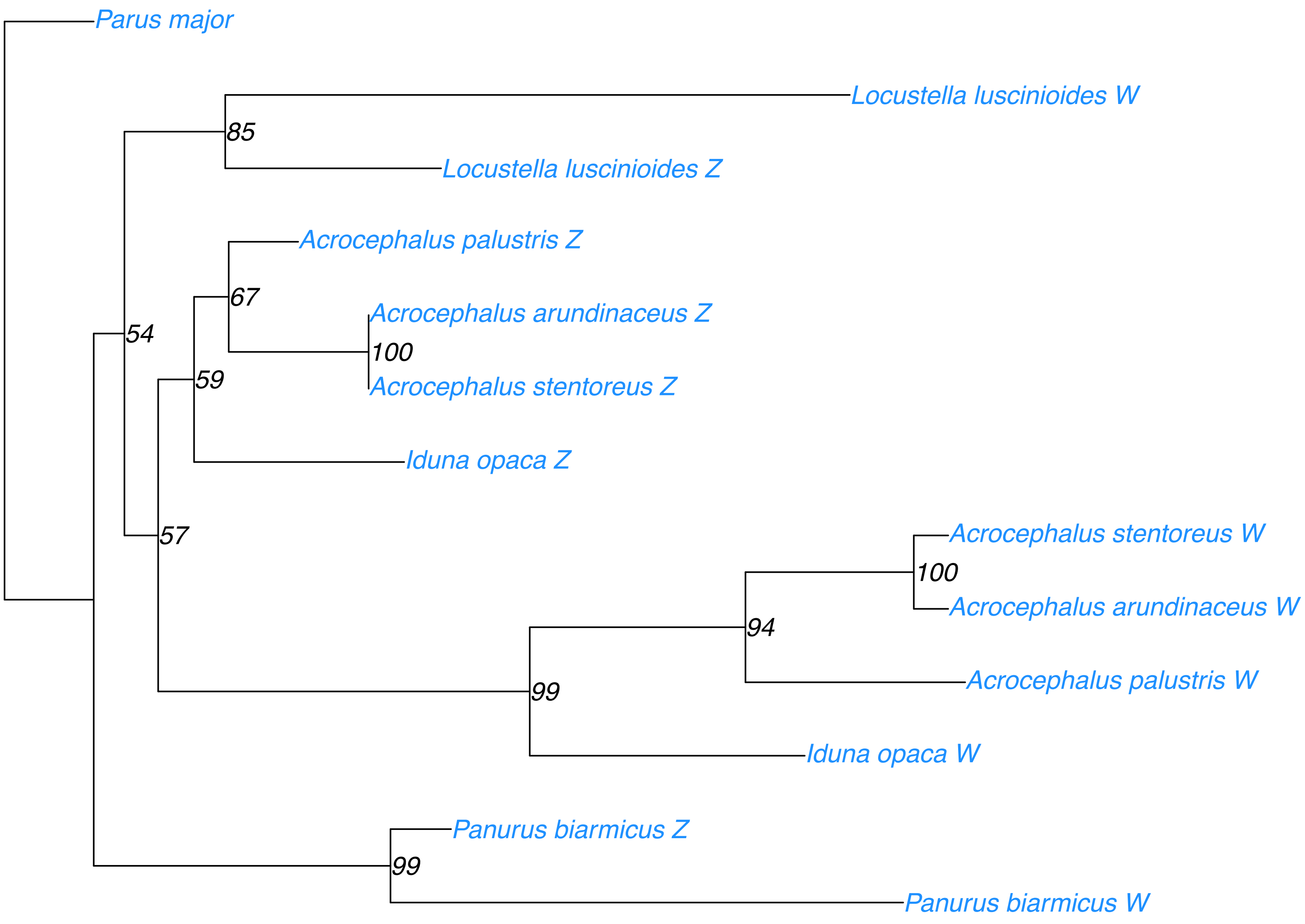

ENSTGUT00000003119

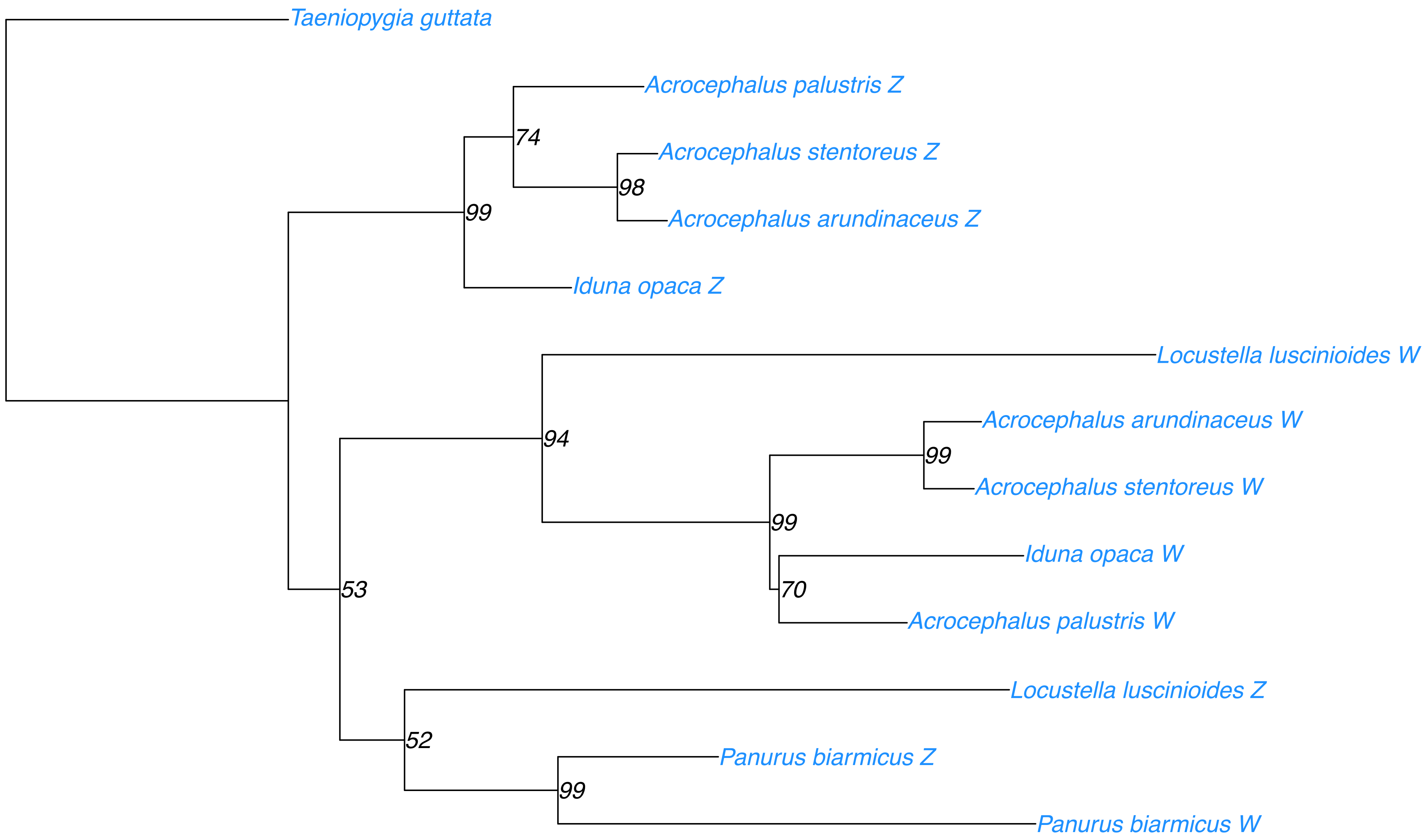

ENSTGUT00000003096

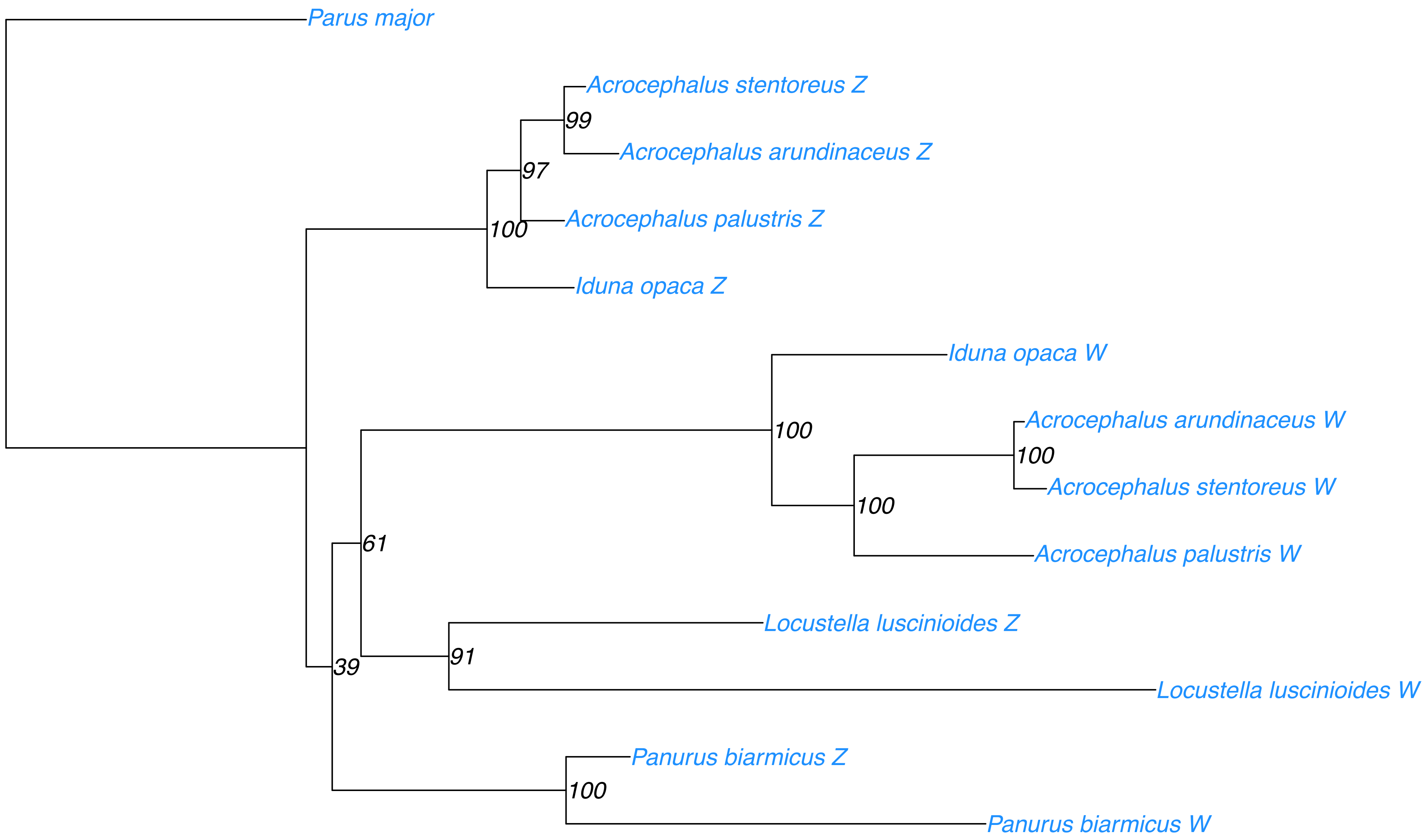

ENSTGUT00000003087

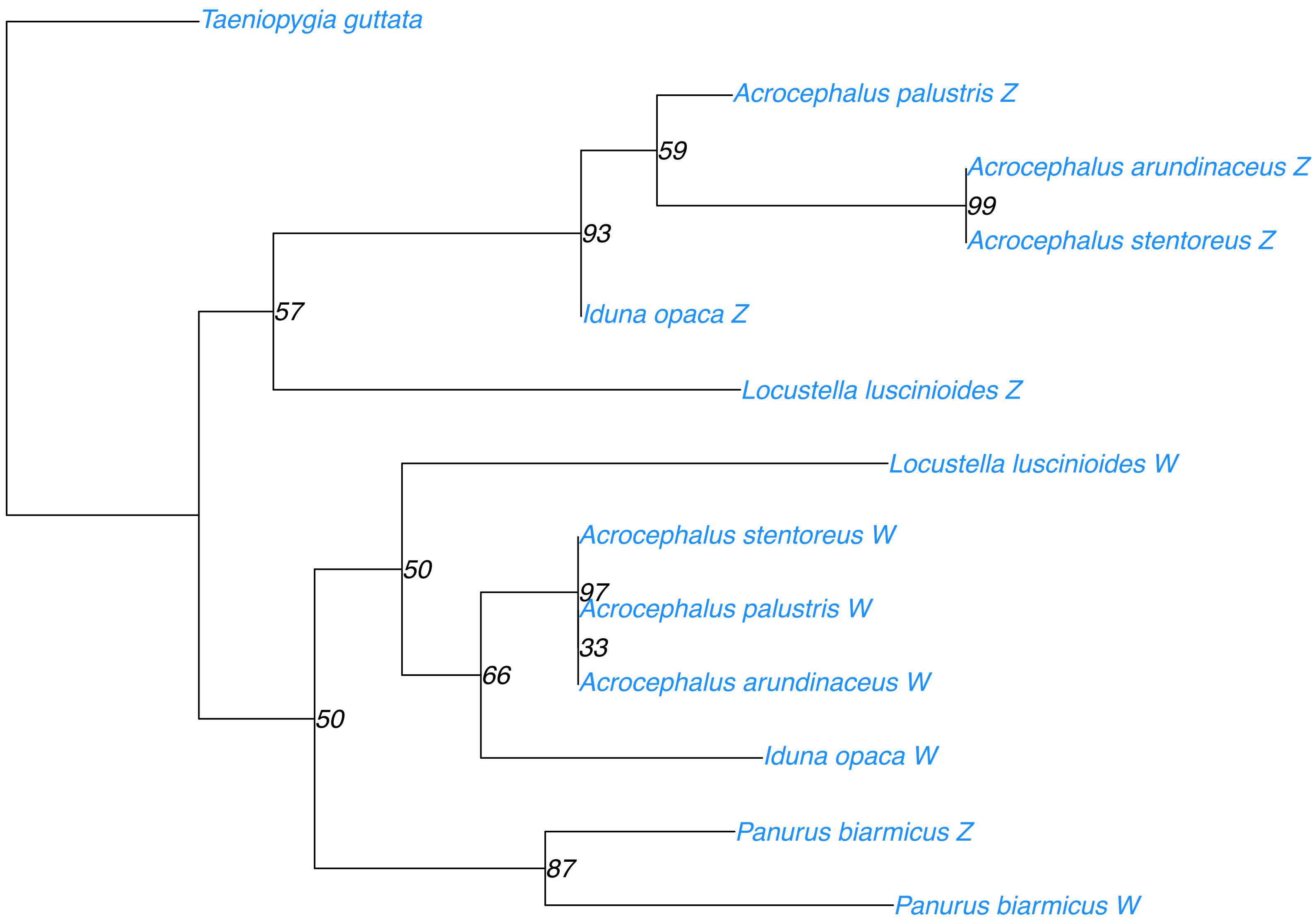

ENSTGUT00000003045

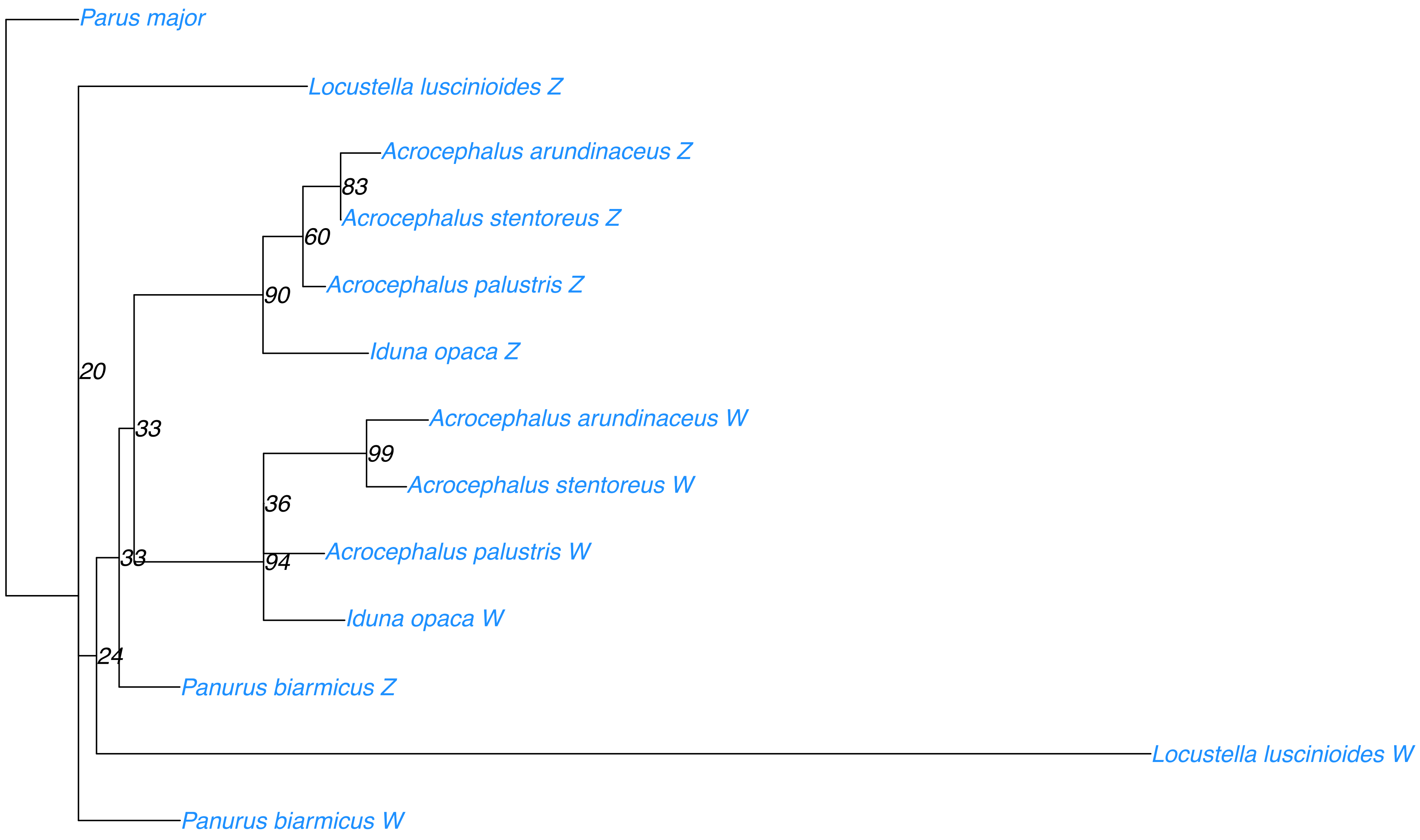

ENSTGUT00000003032

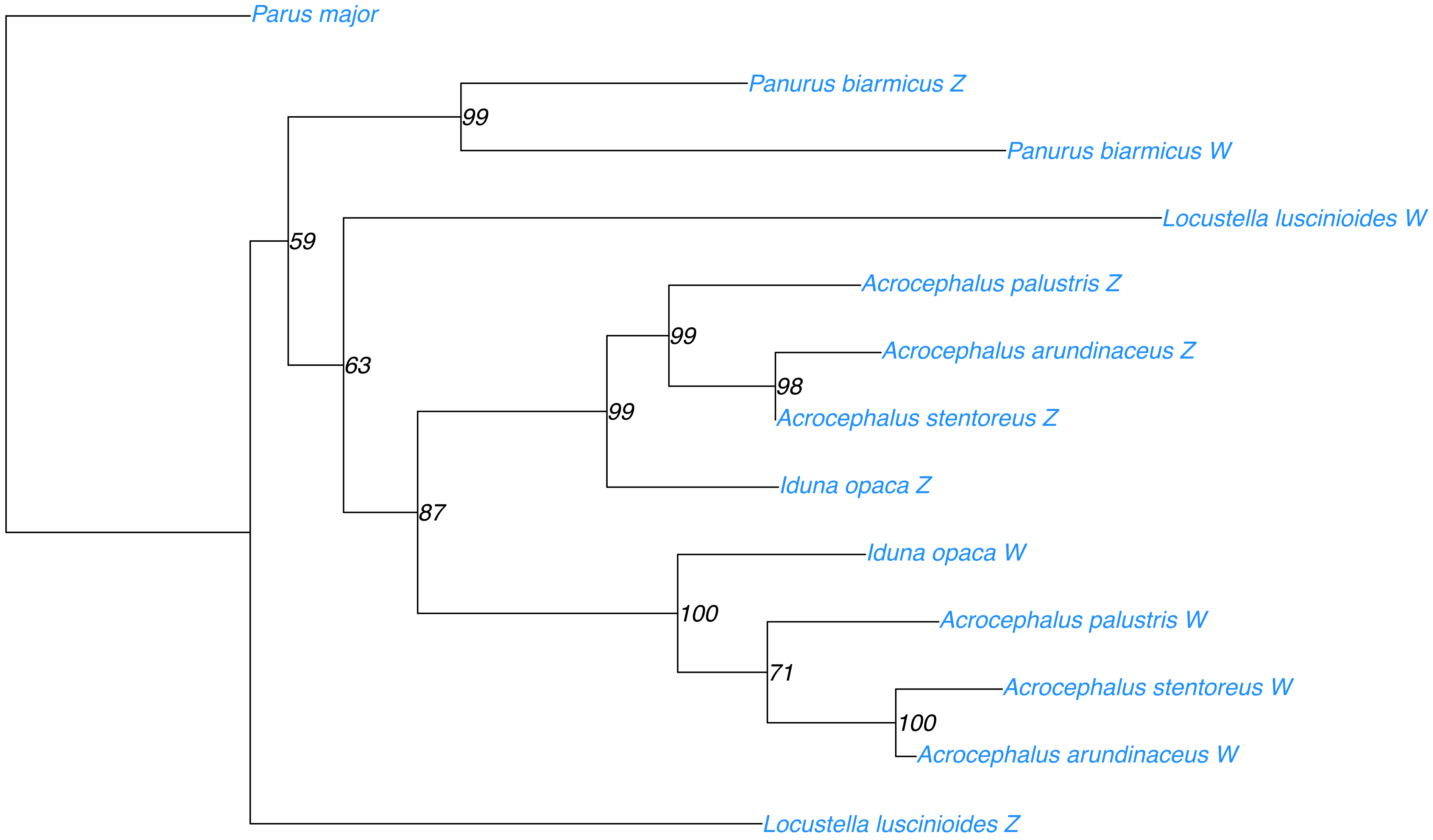

ENSTGUT00000003008

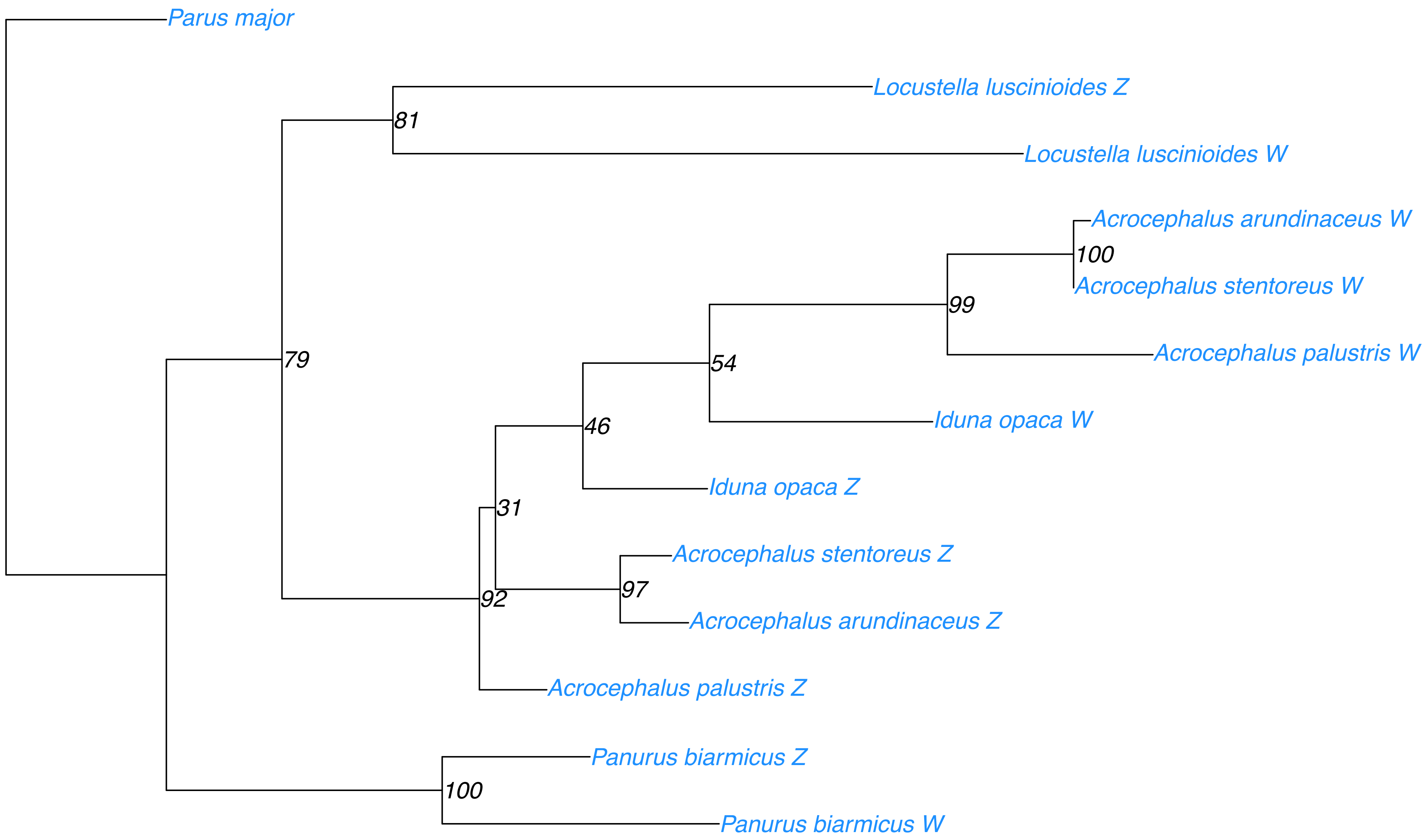

ENSTGUT00000002976

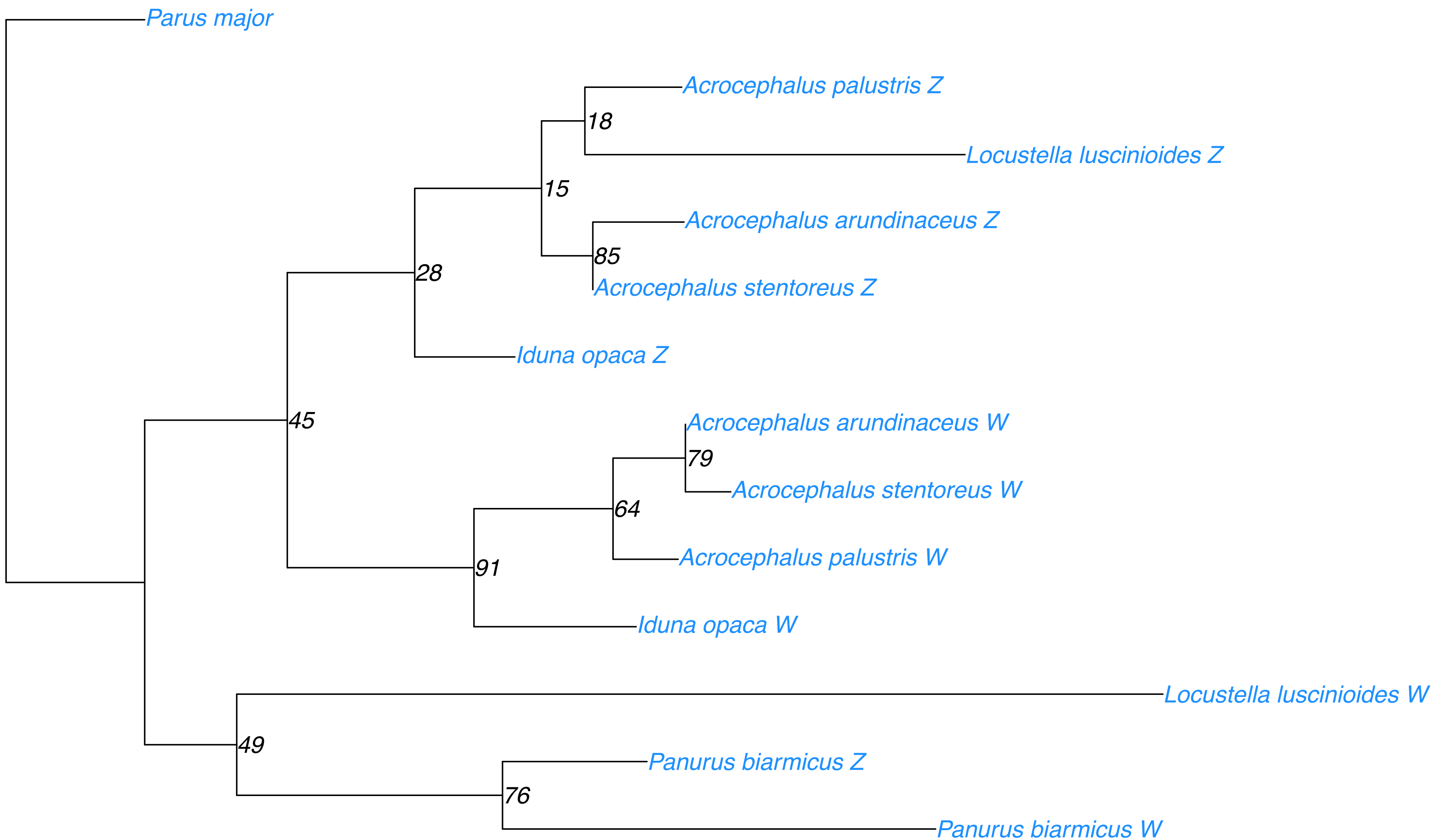

ENSTGUT00000002974

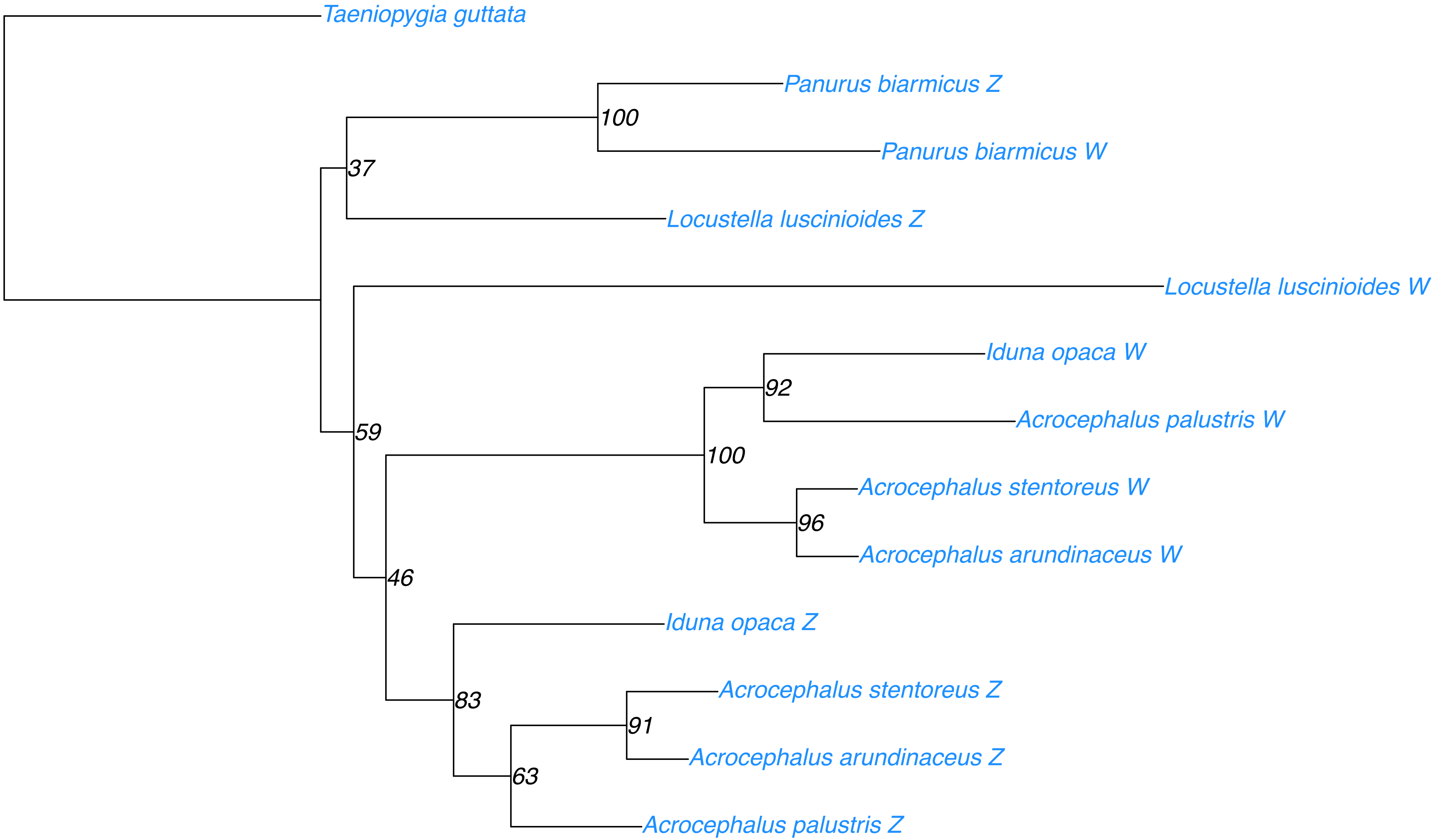

ENSTGUT00000002964

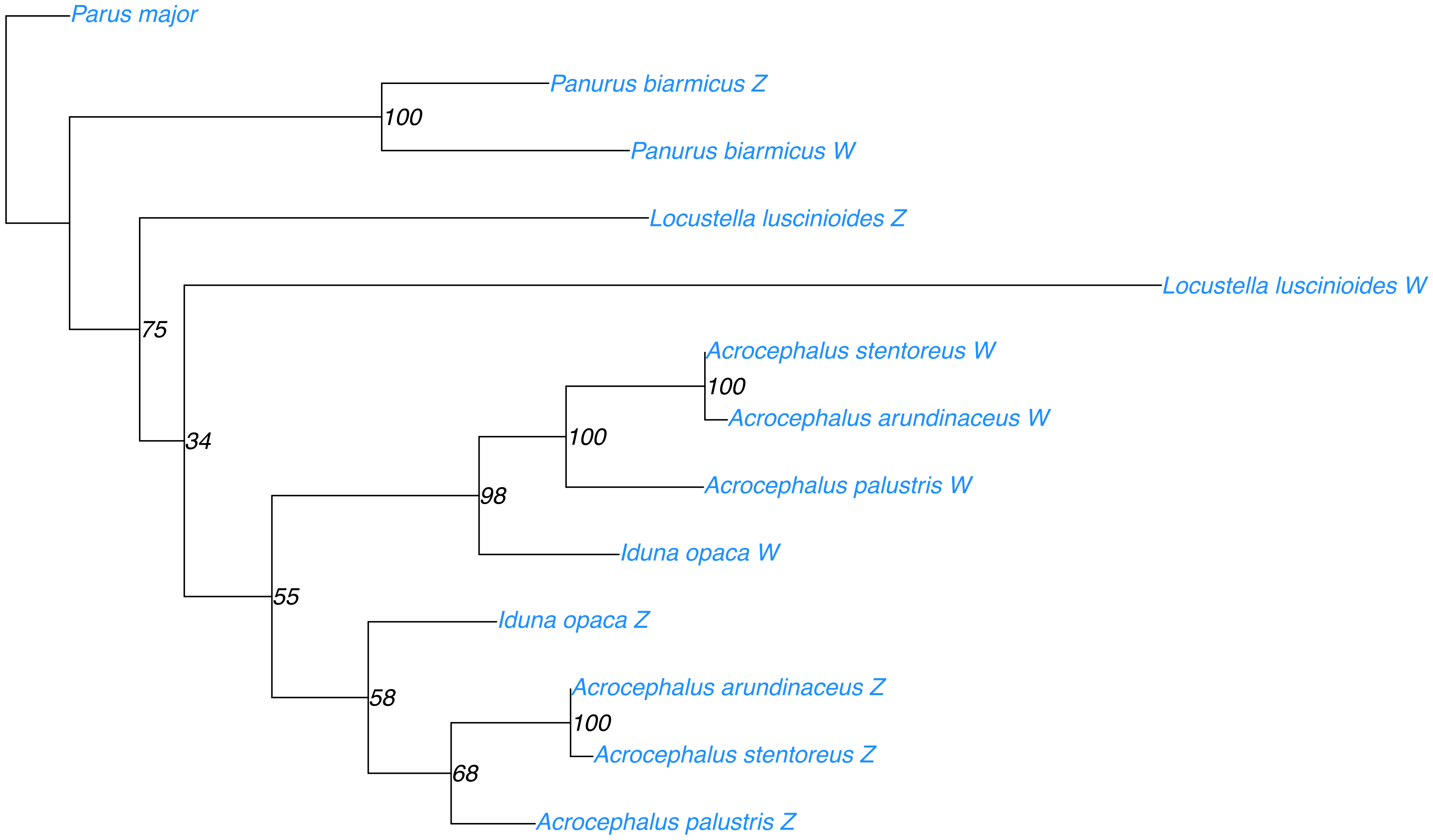

ENSTGUT00000002949

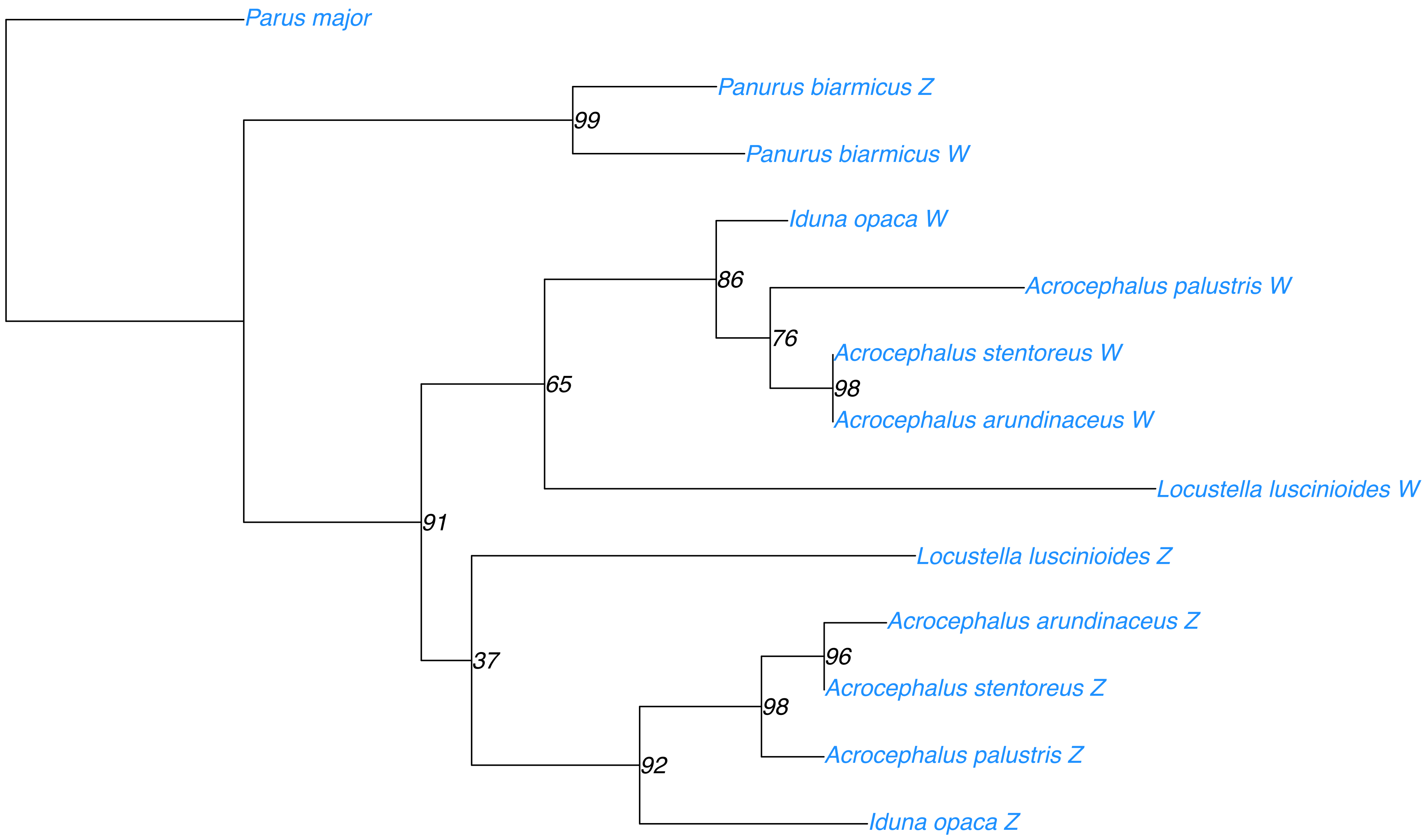

ENSTGUT00000002921

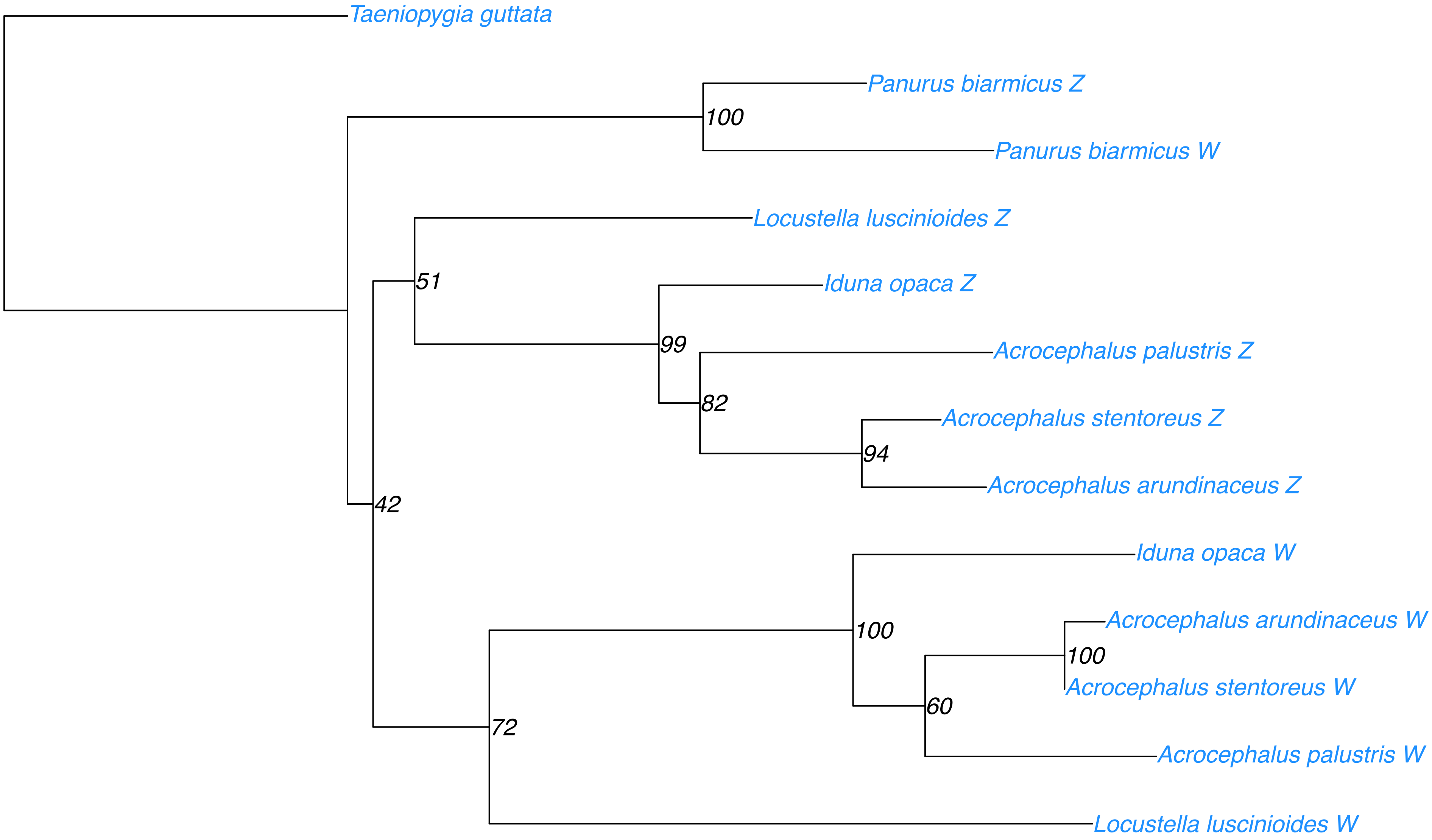

ENSTGUT00000002910

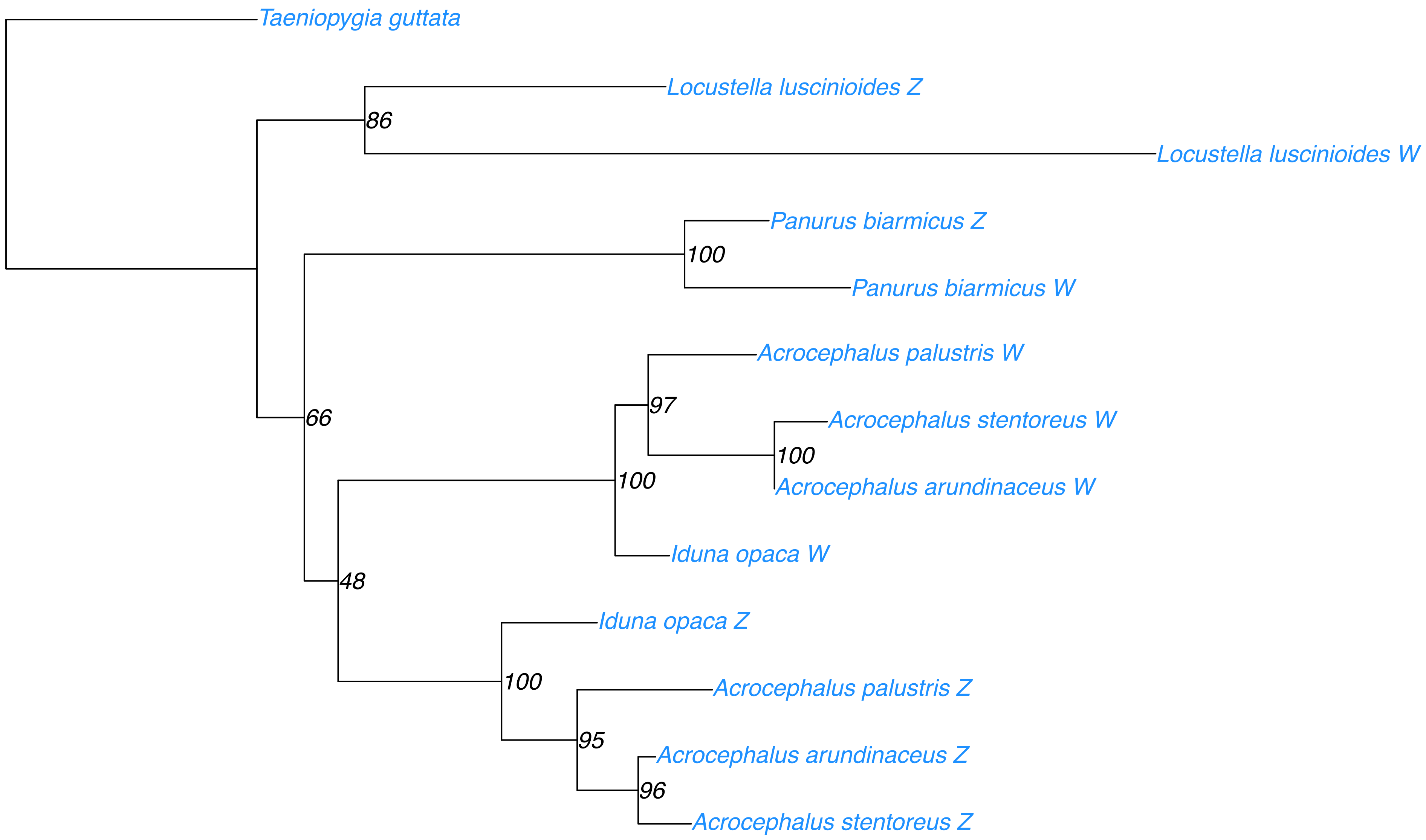

ENSTGUT00000002897

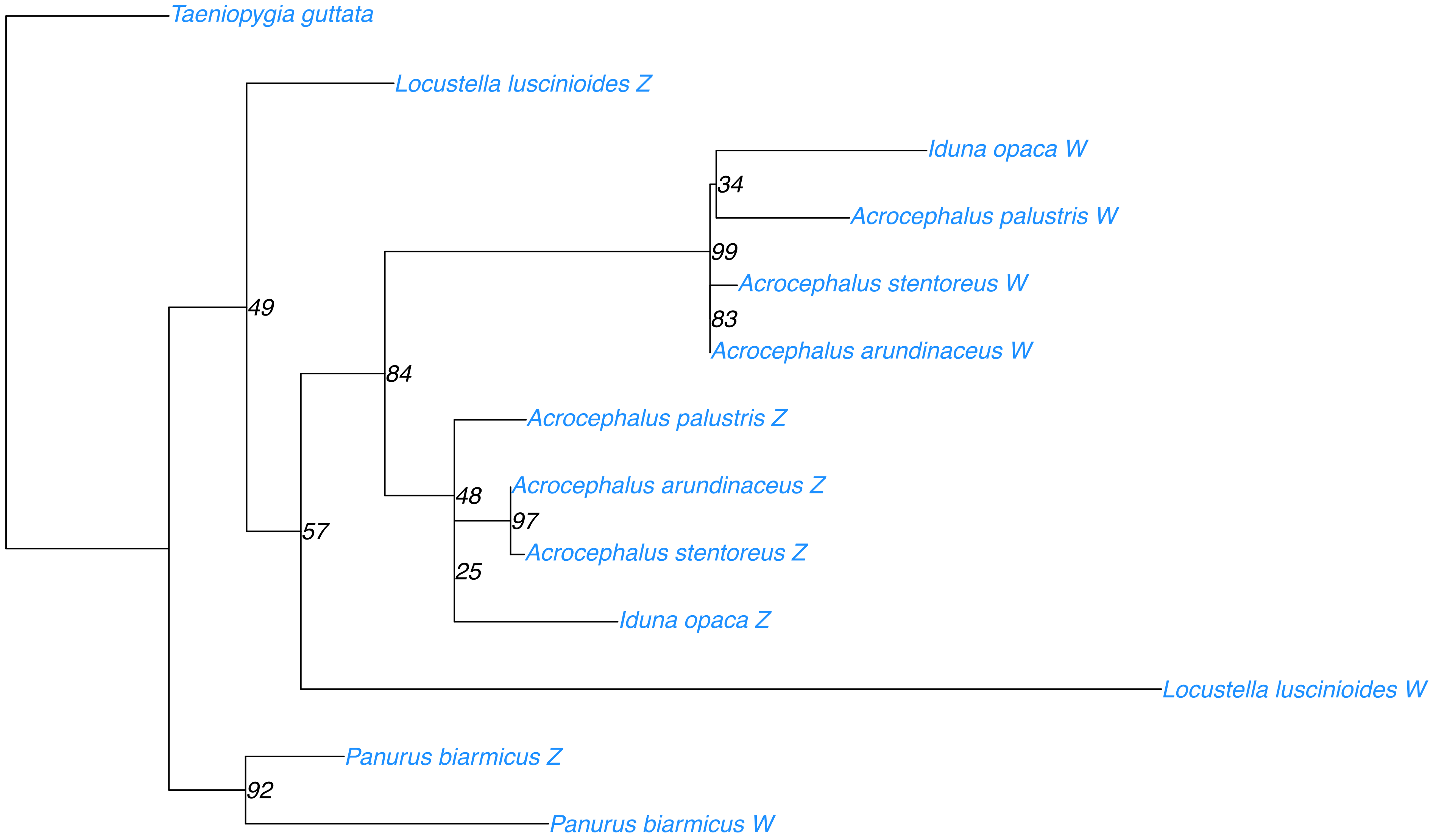

ENSTGUT00000002888

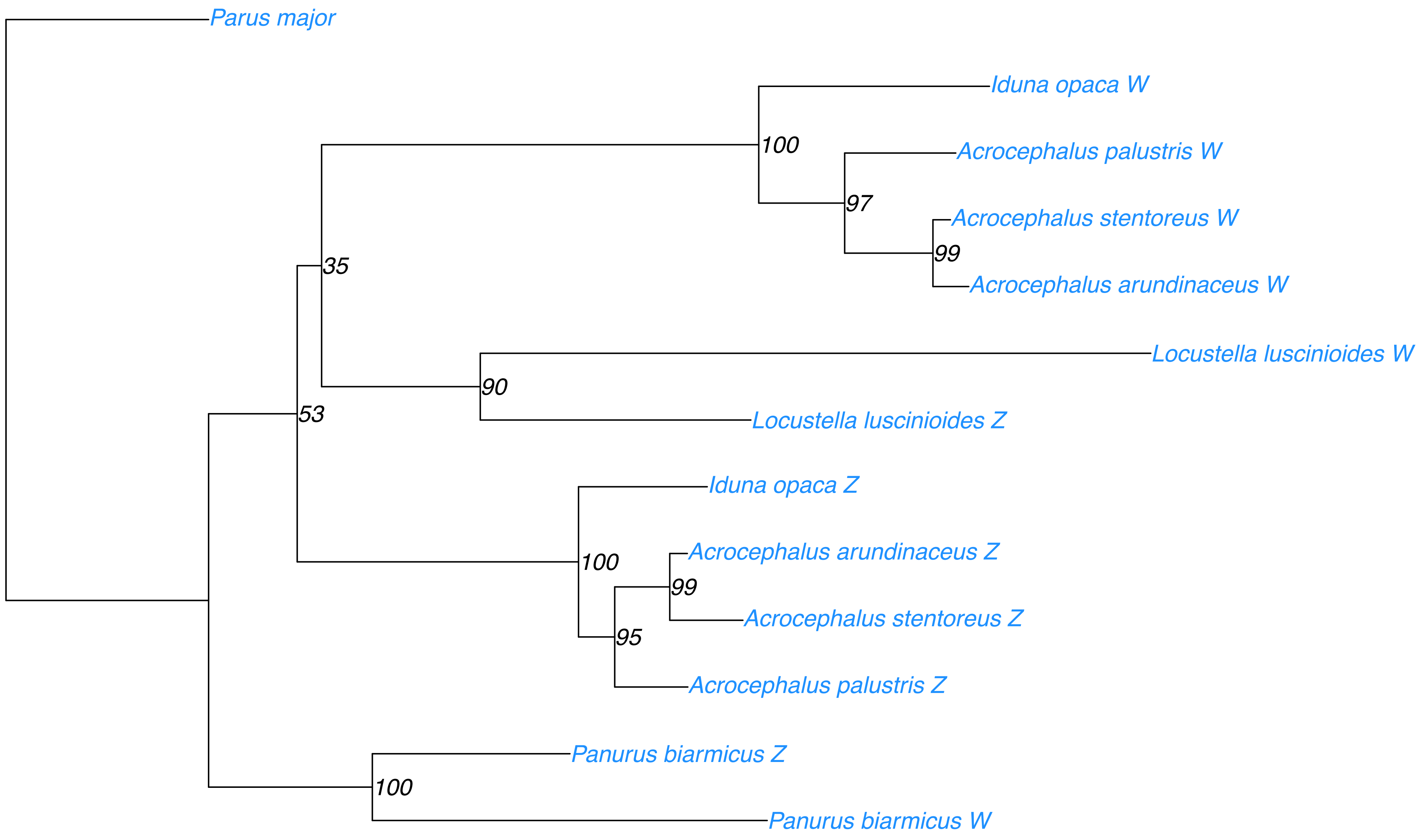

ENSTGUT00000002865

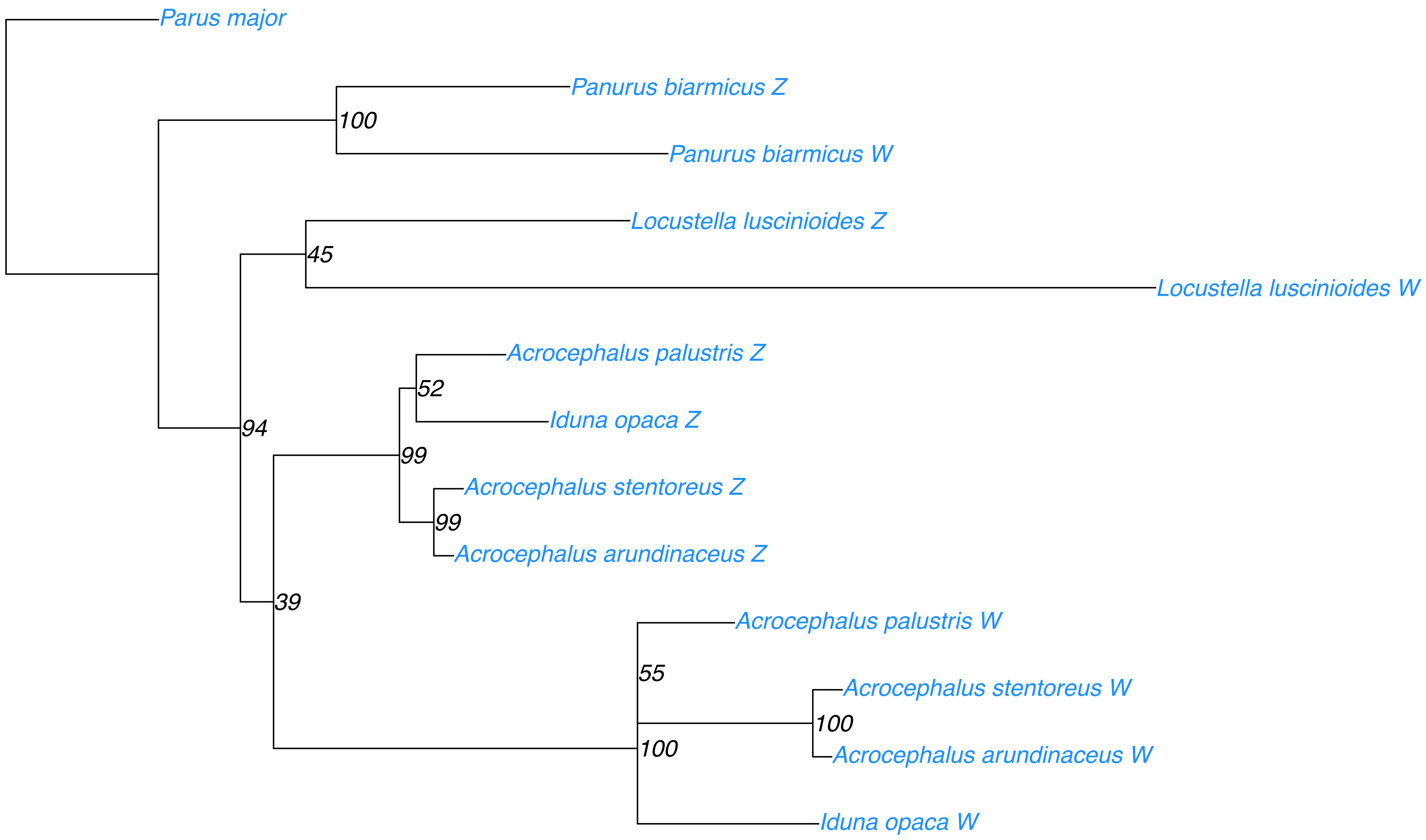

ENSTGUT00000002833

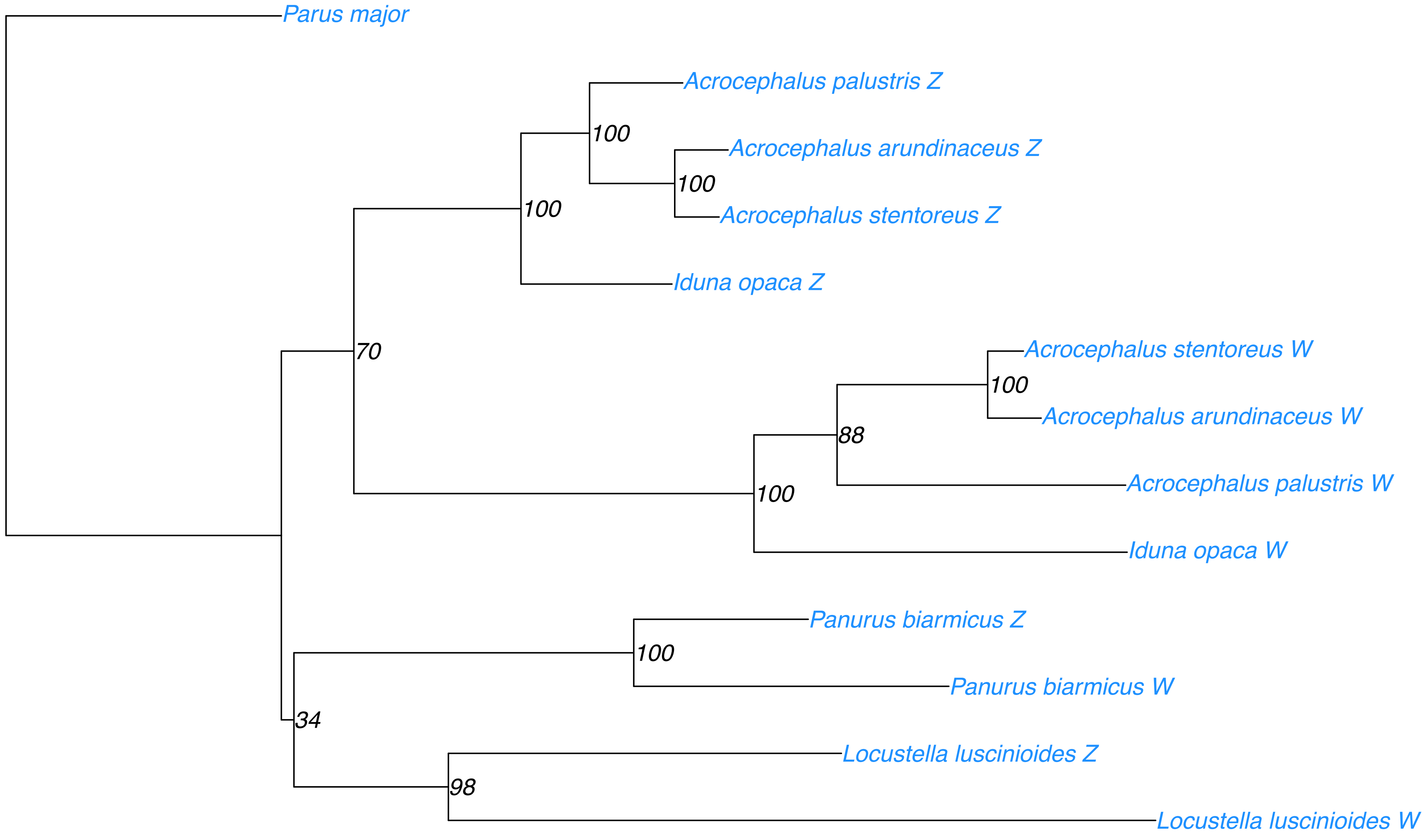

ENSTGUT00000002805

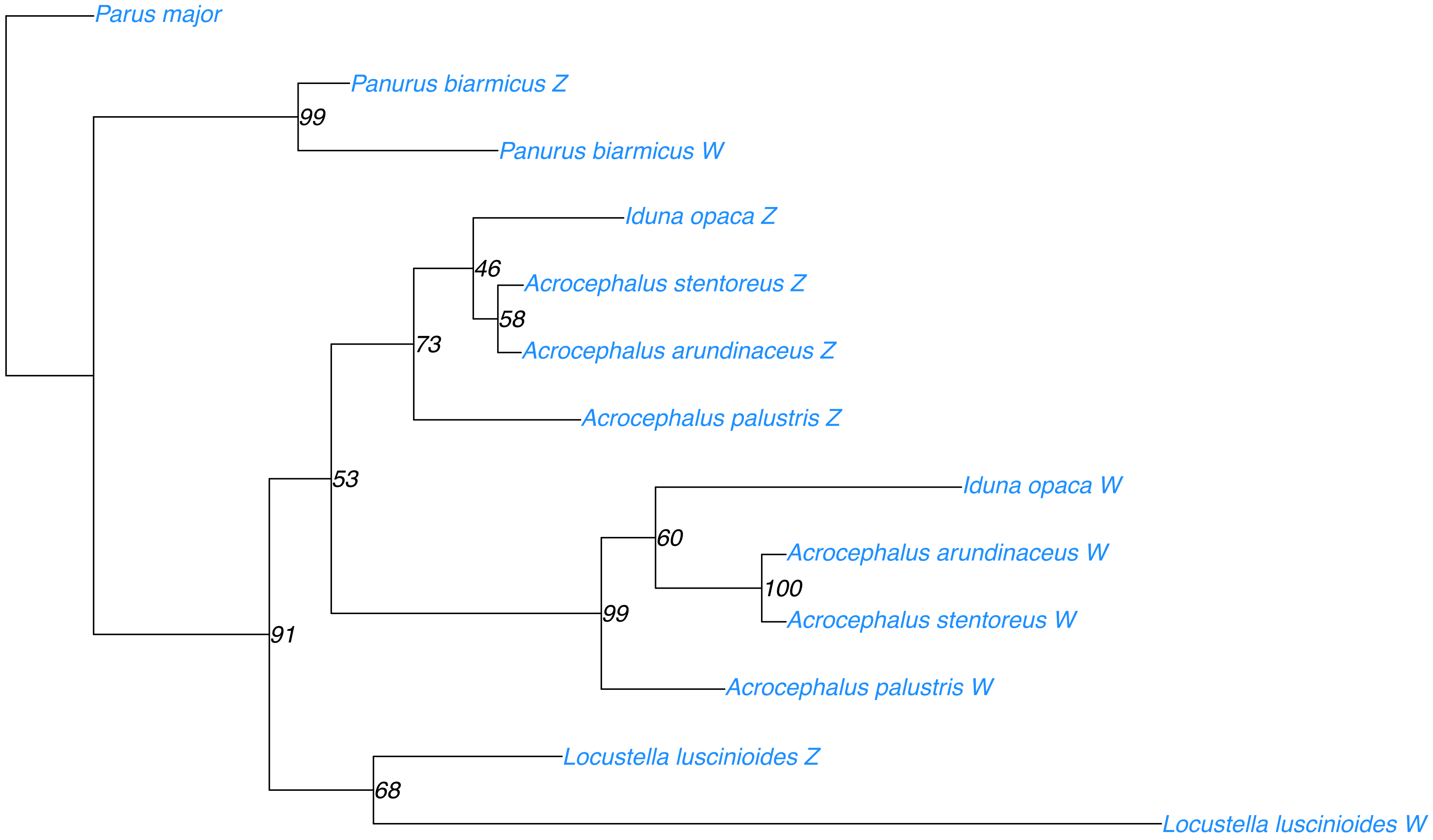

ENSTGUT00000002798

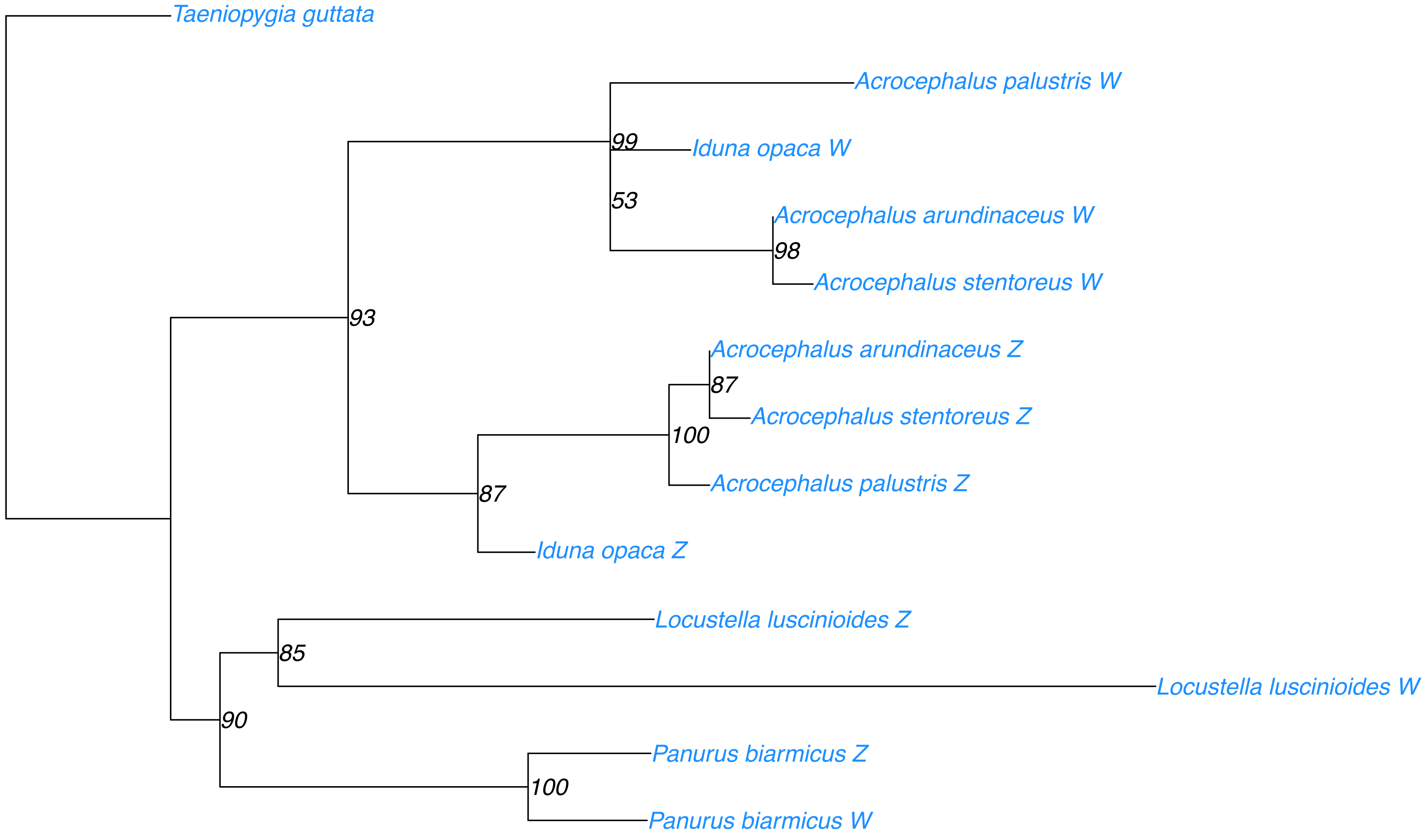

ENSTGUT00000002786

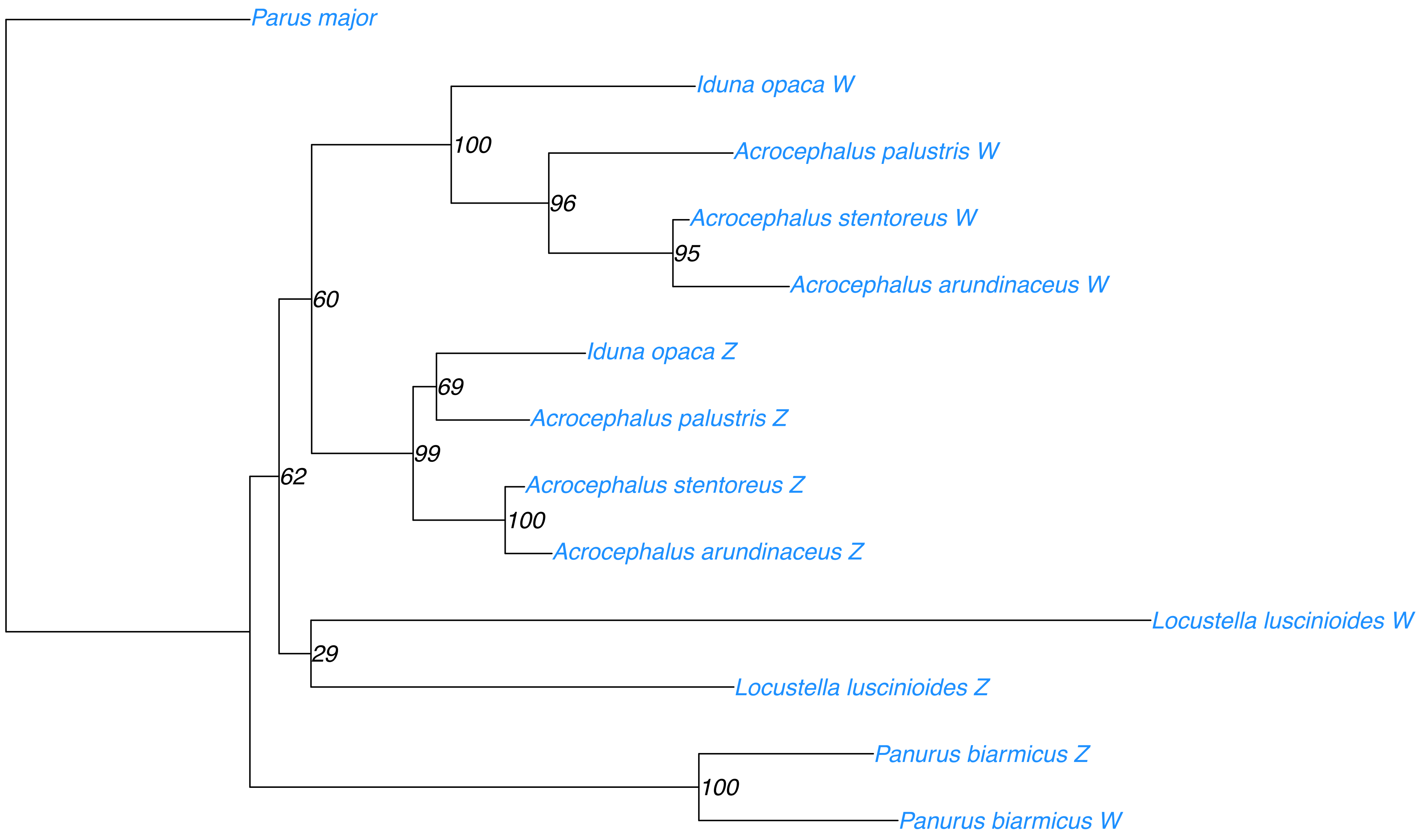

ENSTGUT00000002785

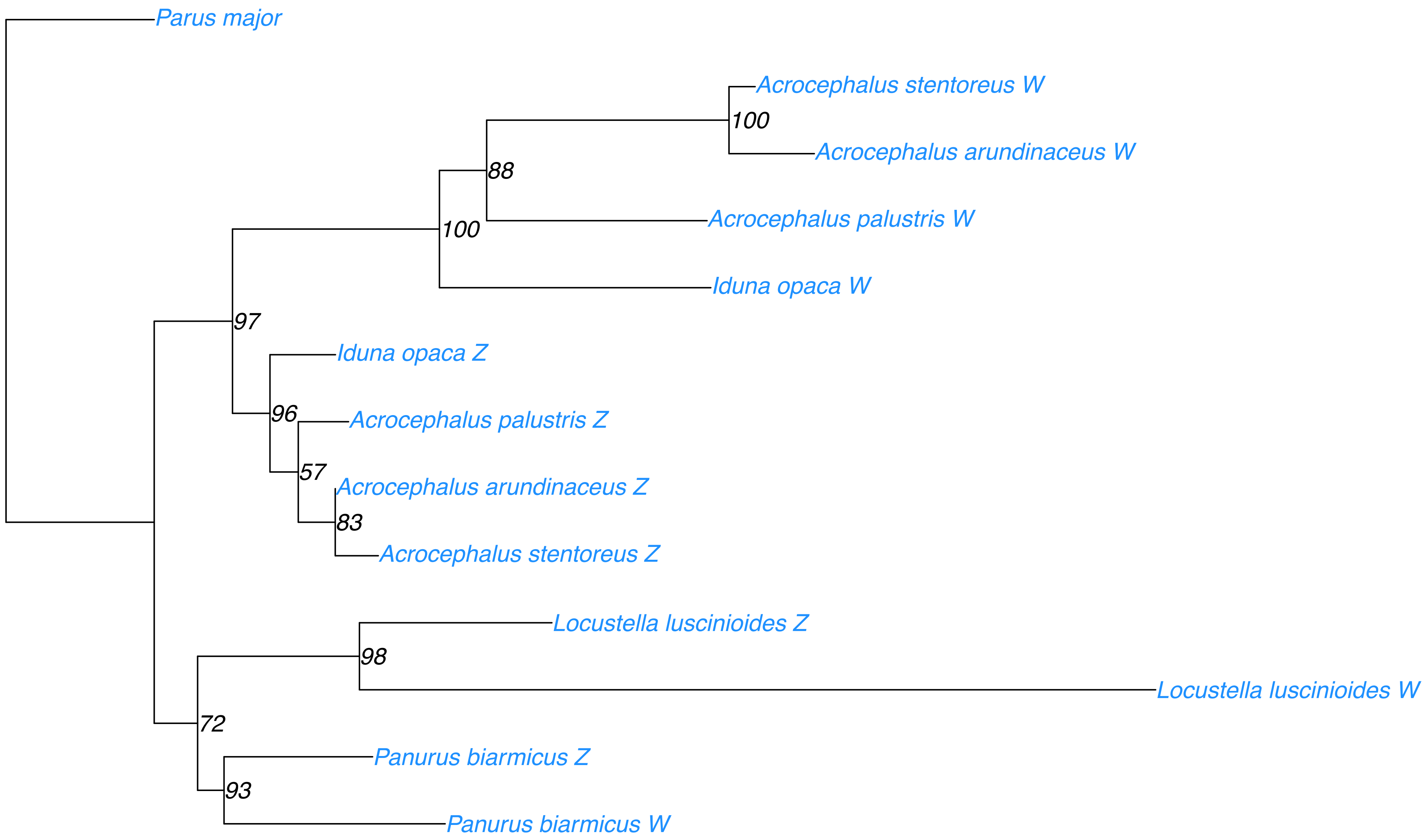

ENSTGUT00000002780

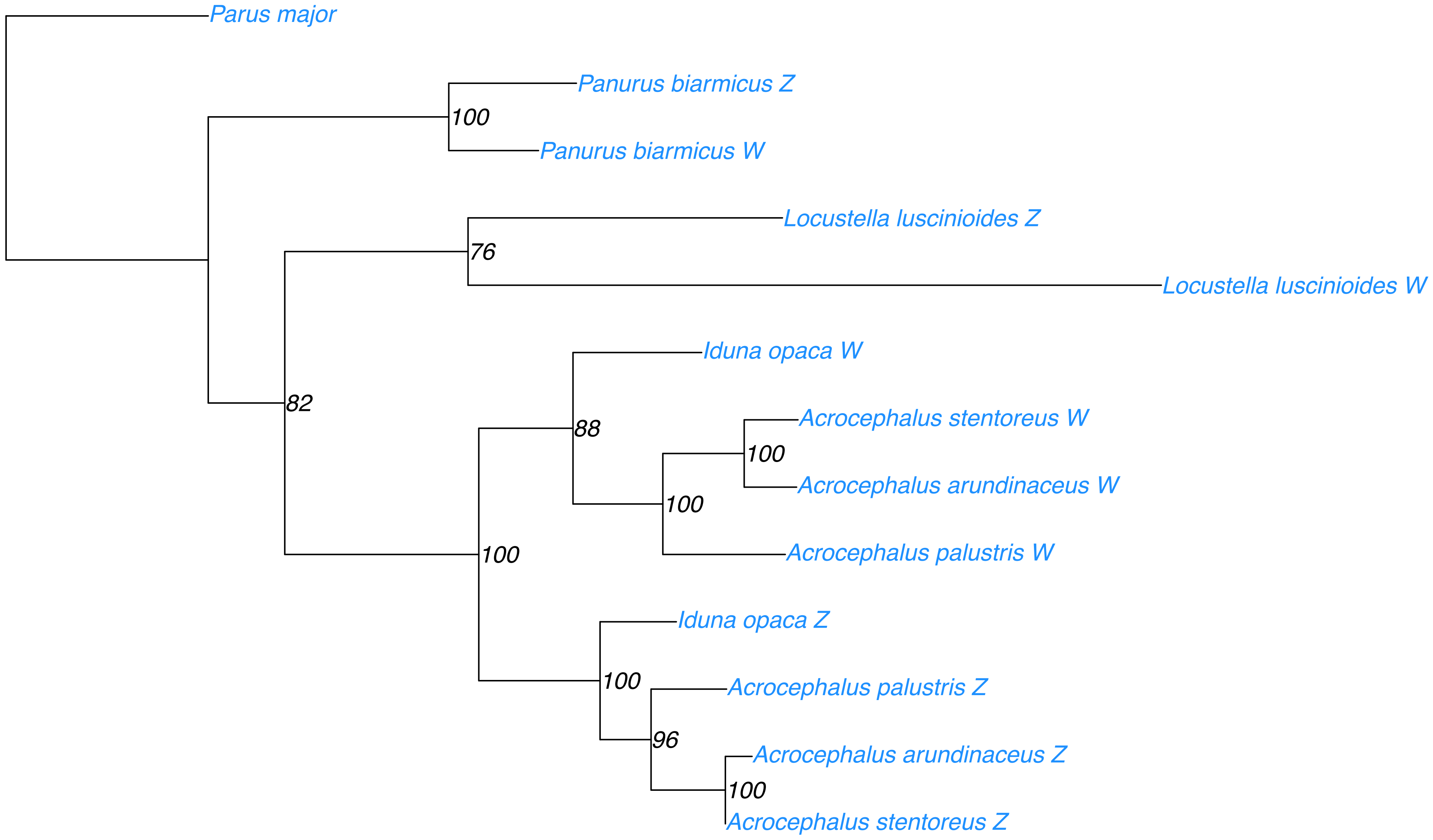

ENSTGUT00000002773

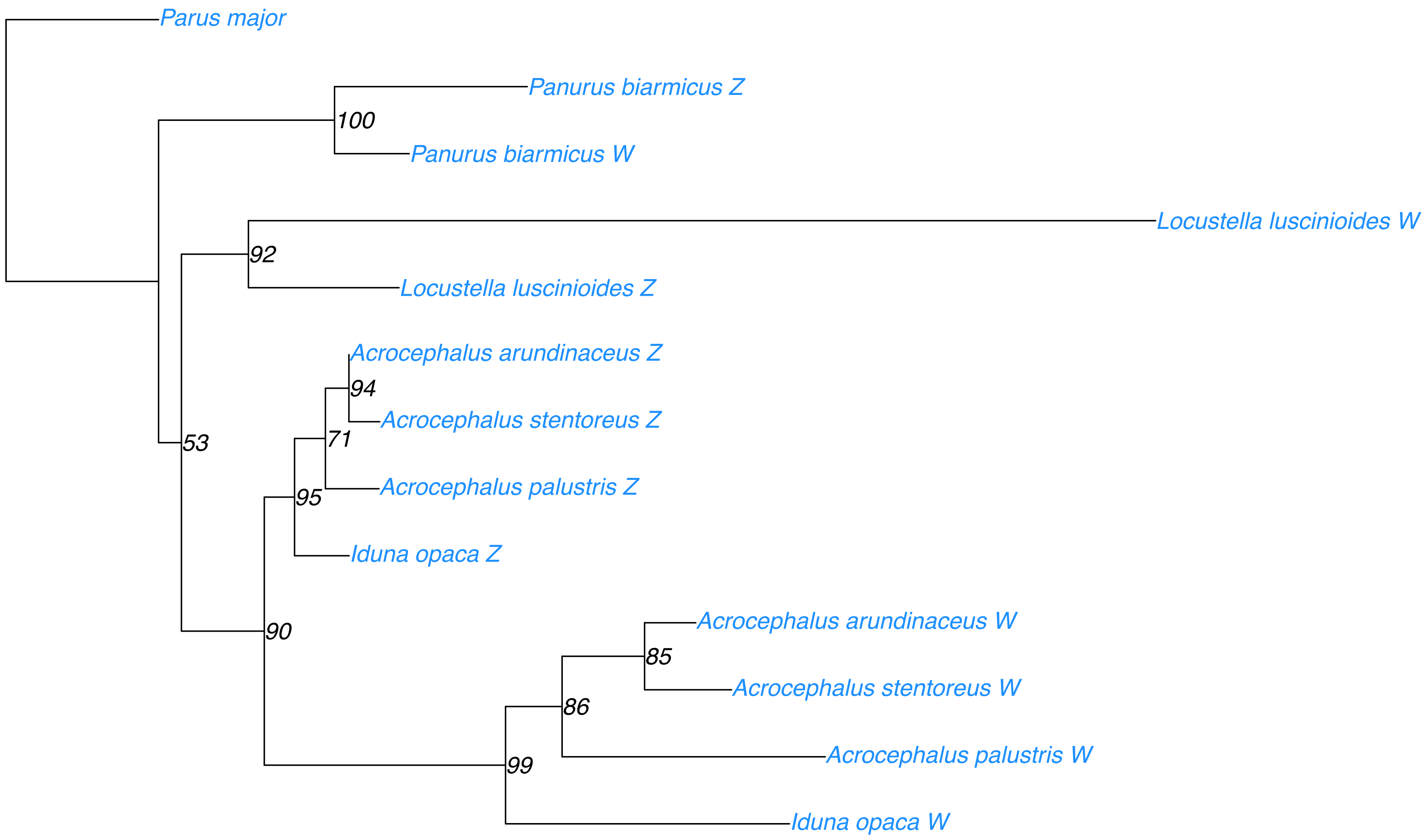

ENSTGUT00000002769

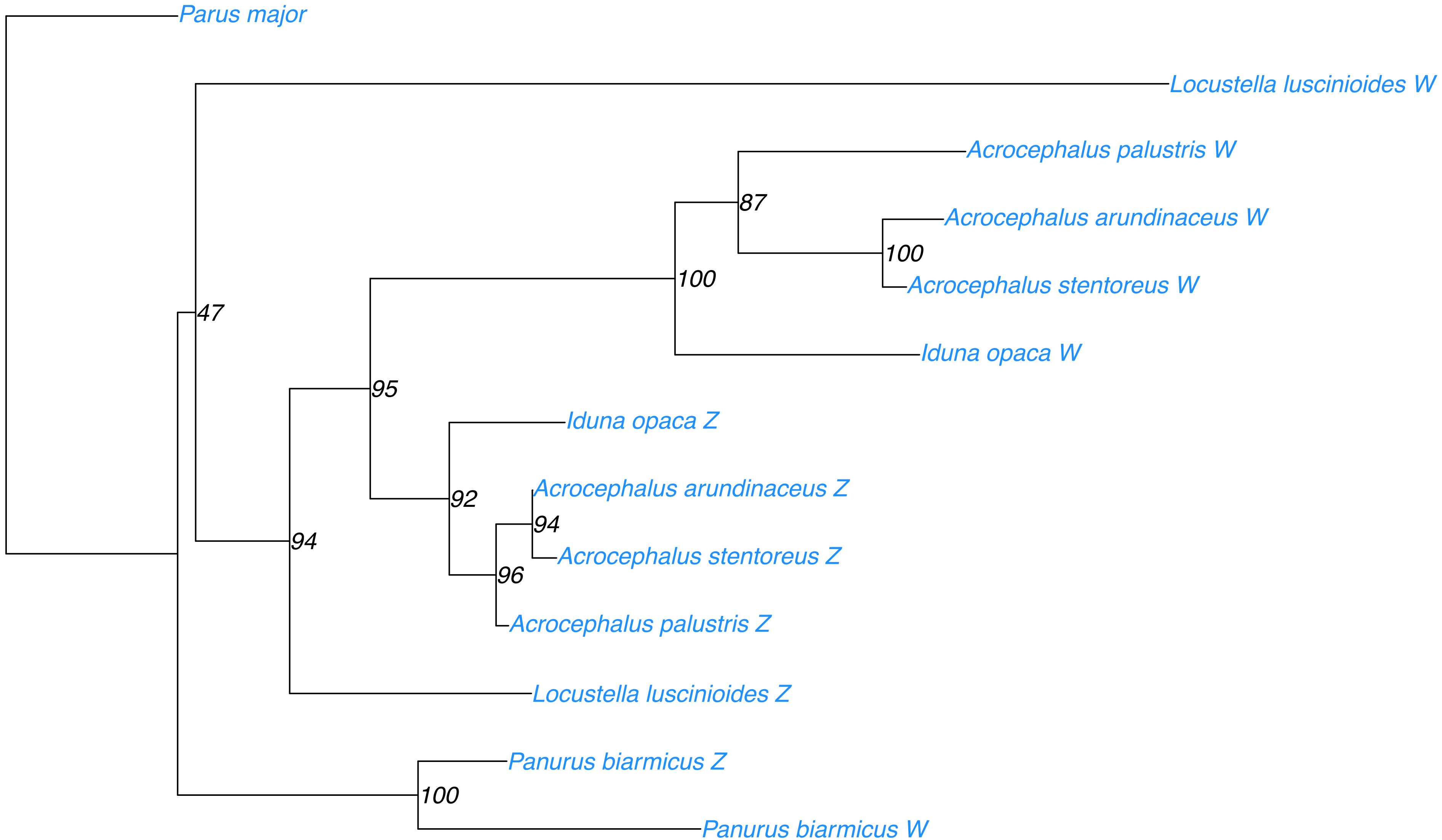

ENSTGUT00000002747

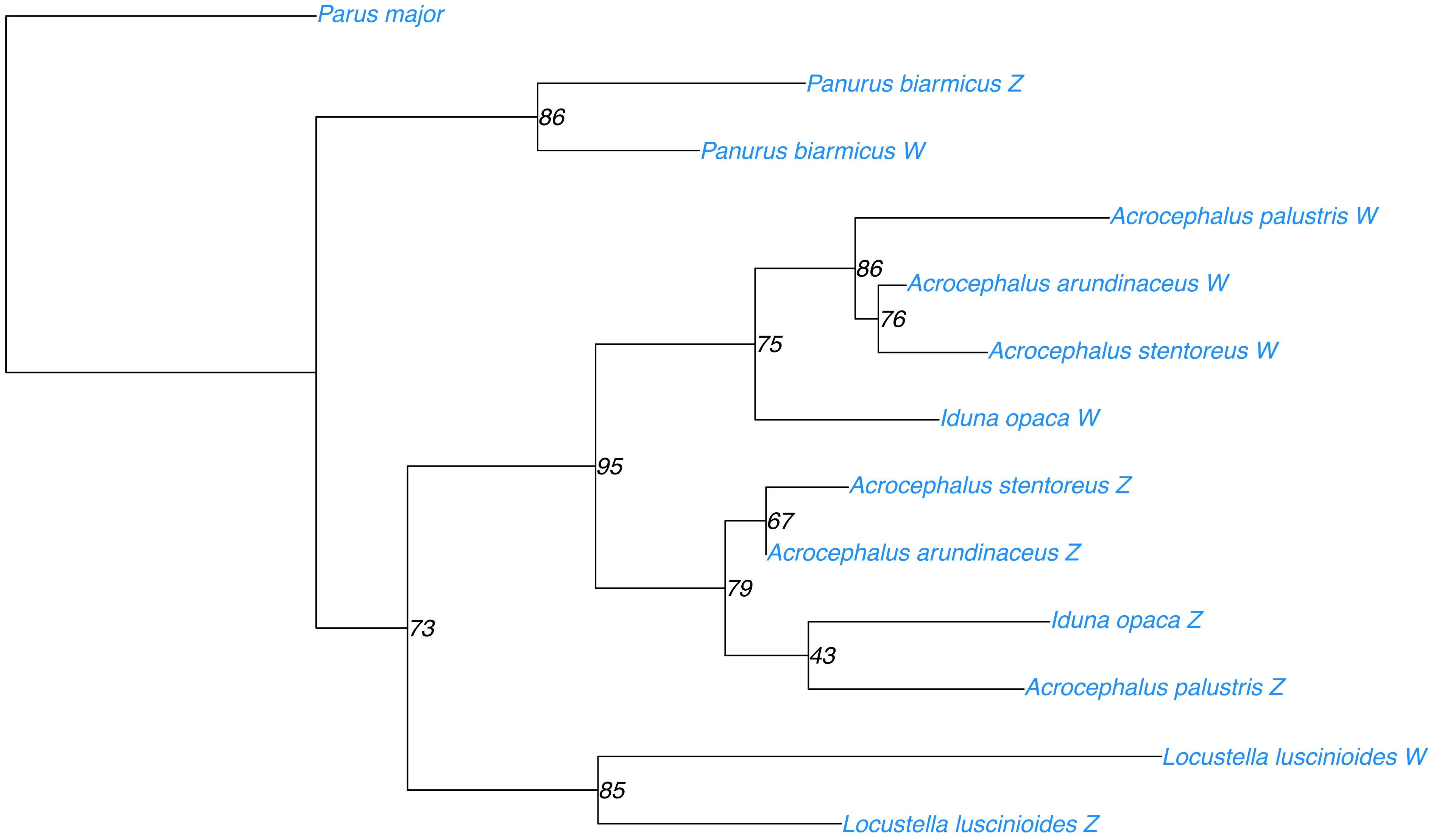

ENSTGUT00000002712

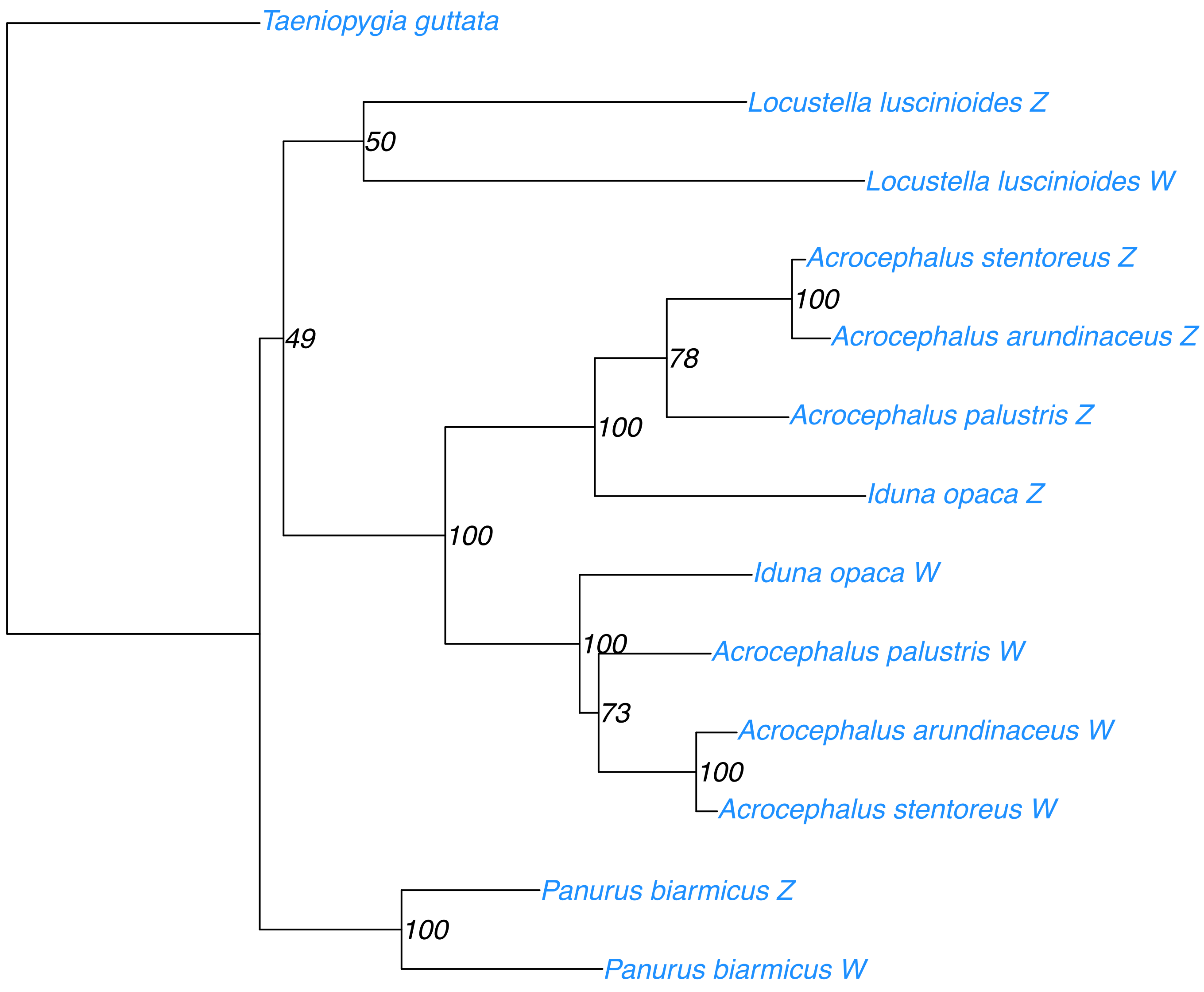

ENSTGUT00000002697

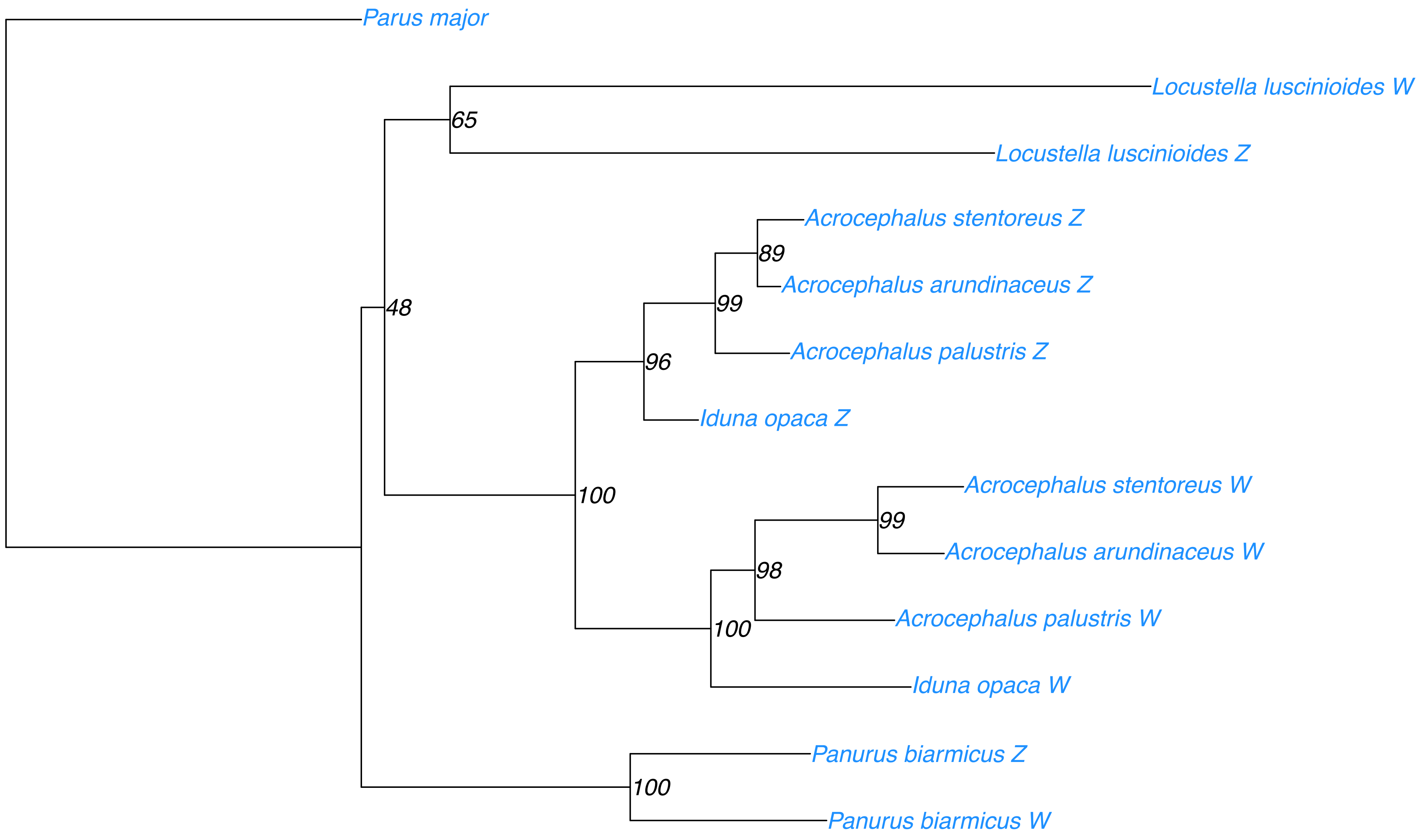

ENSTGUT00000002687

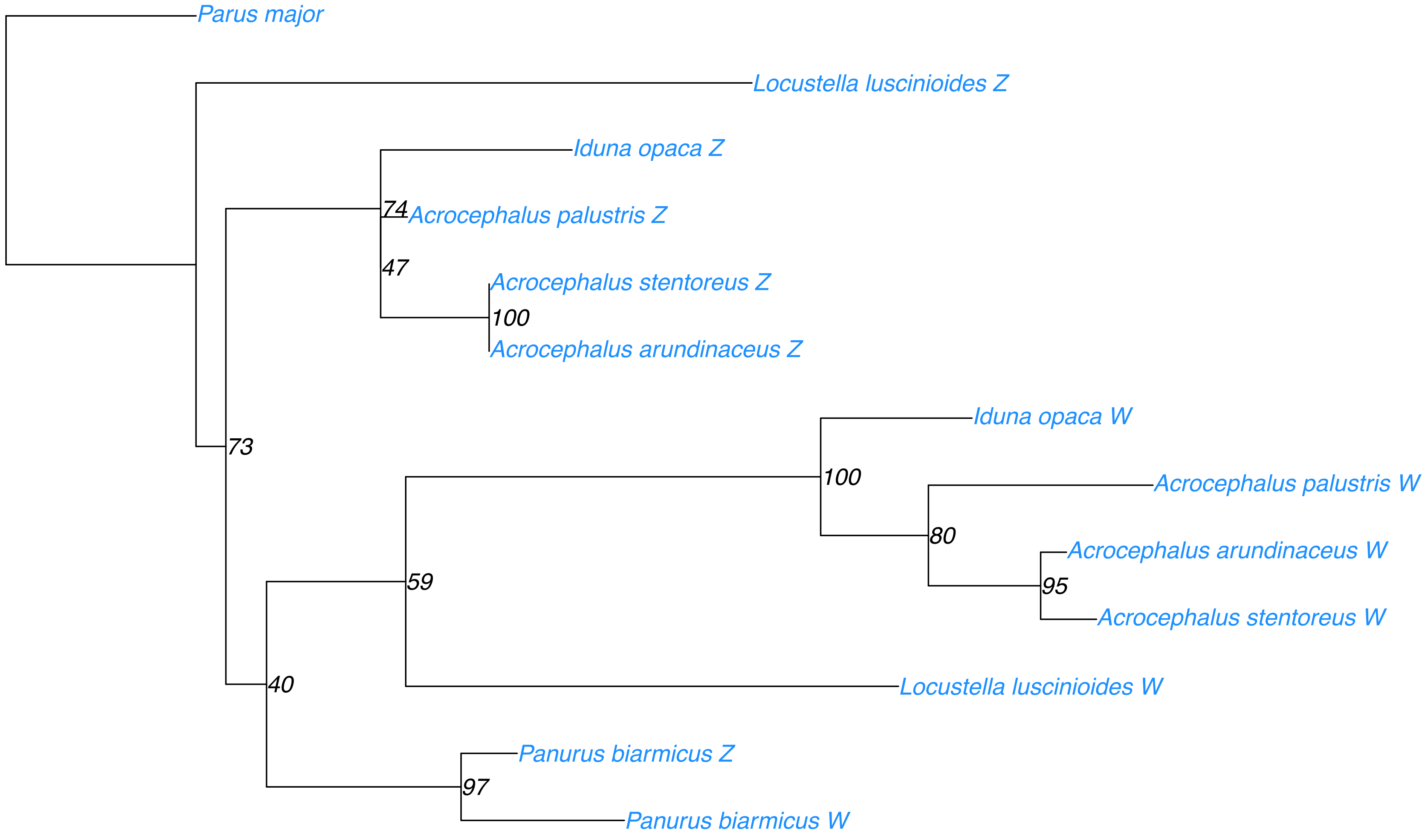

ENSTGUT00000002684

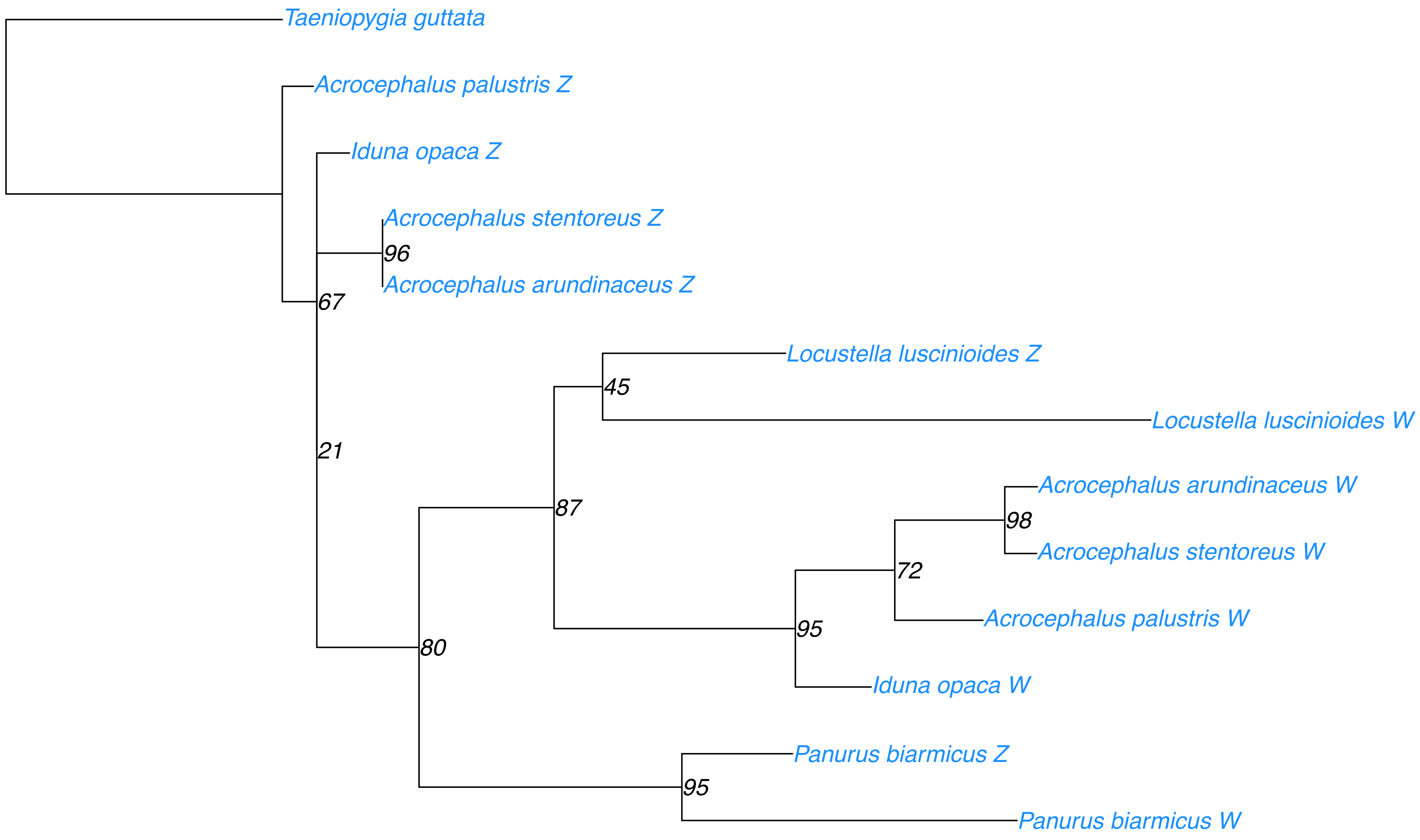

ENSTGUT00000002679

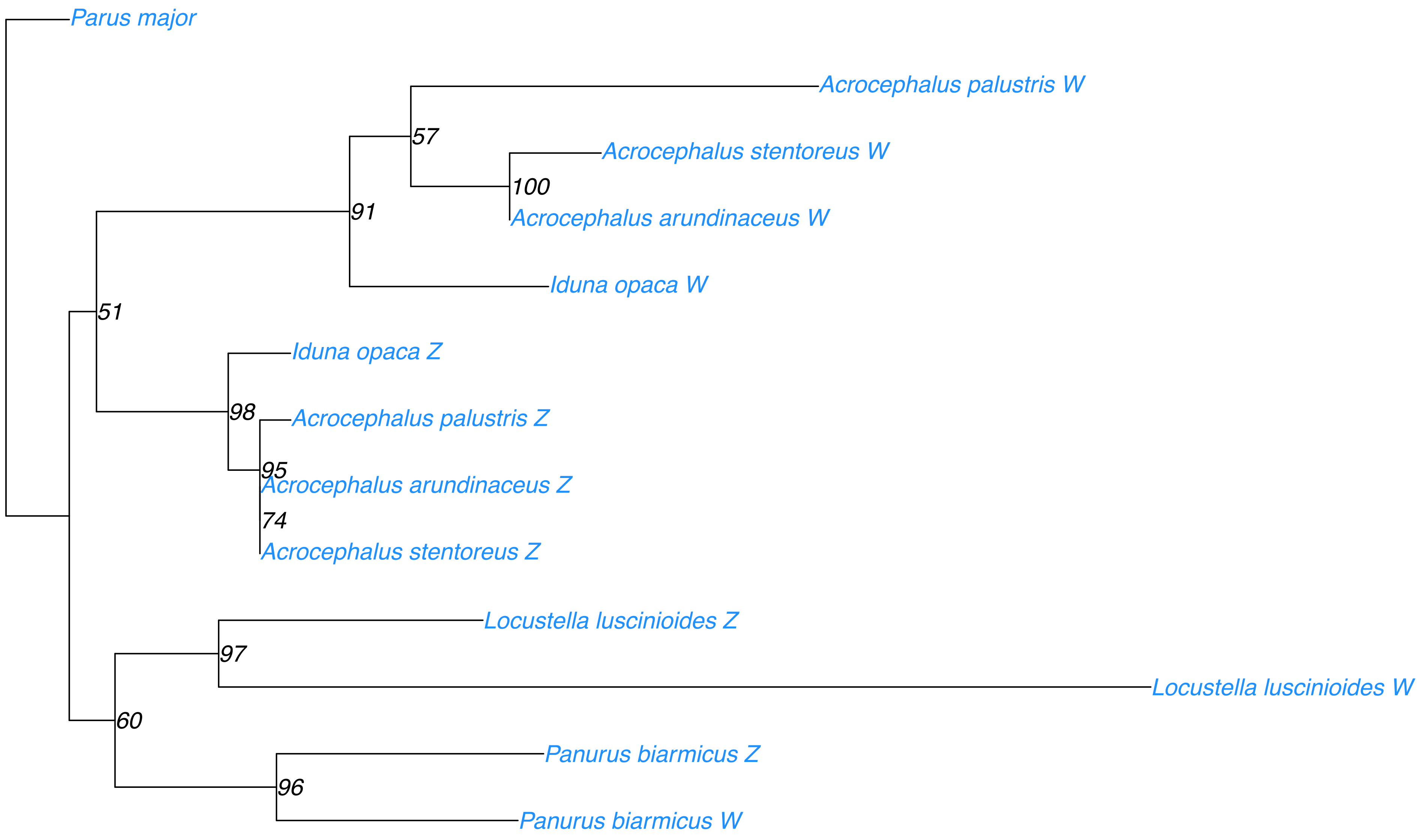

ENSTGUT00000015706

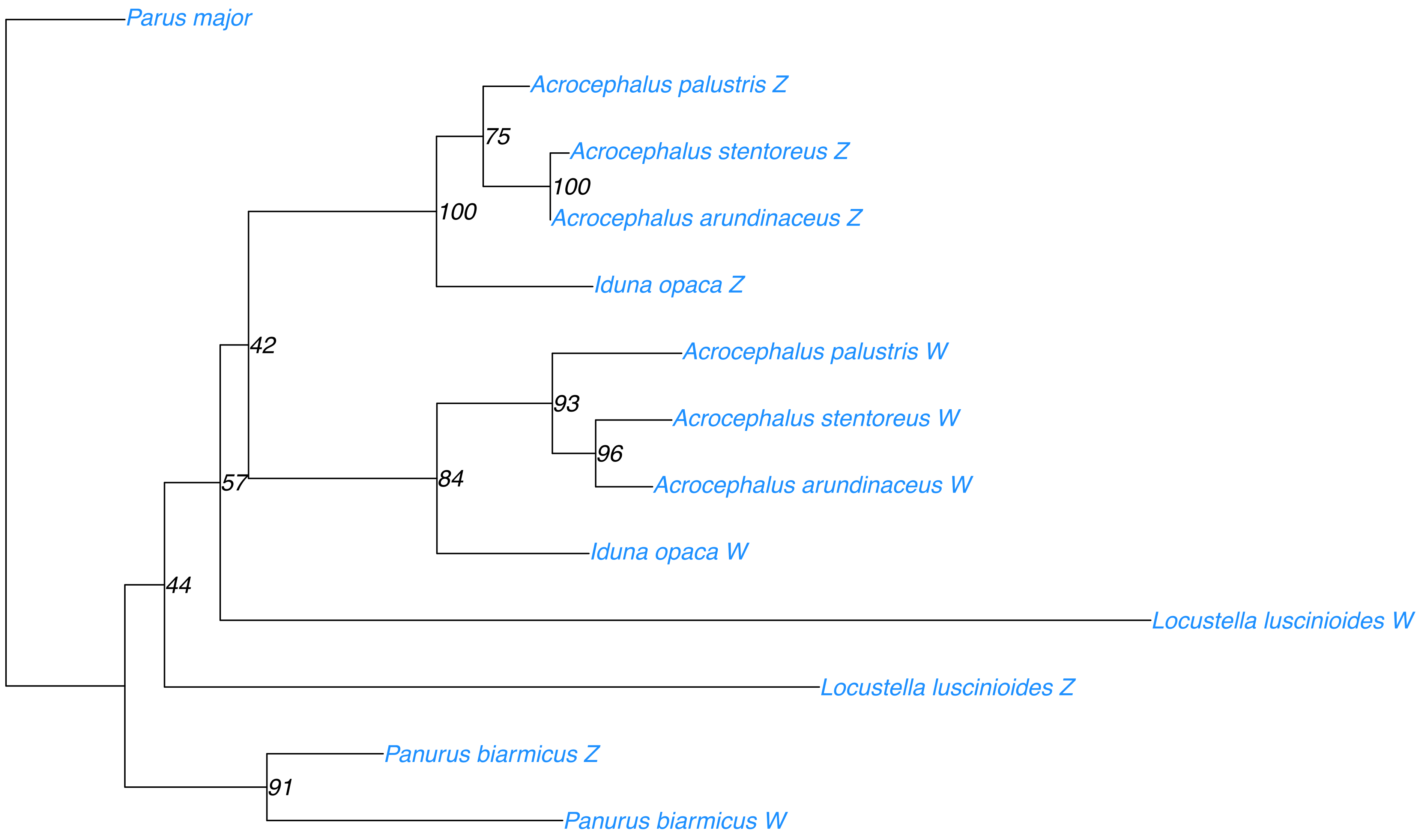

ENSTGUT00000002666

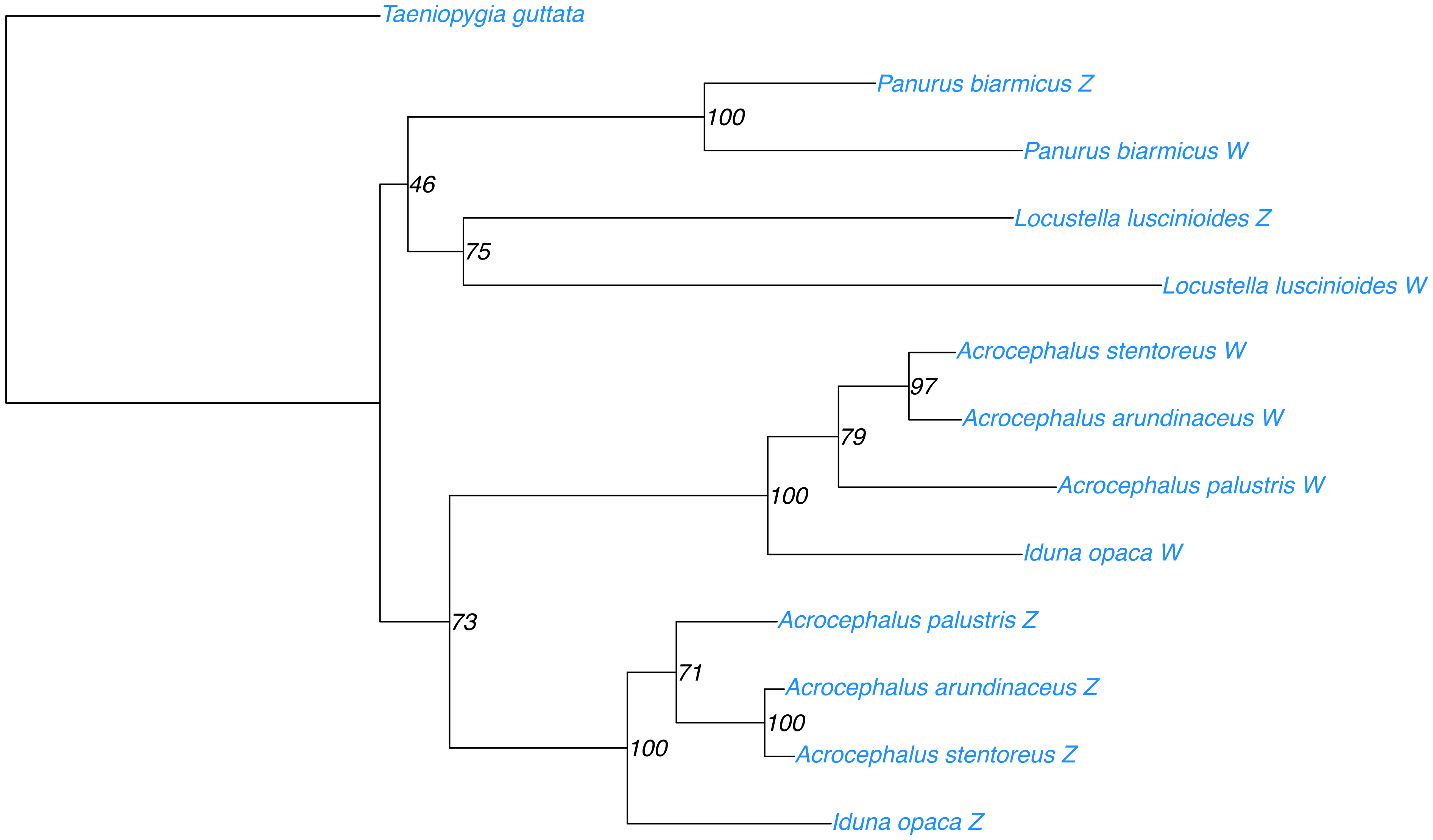

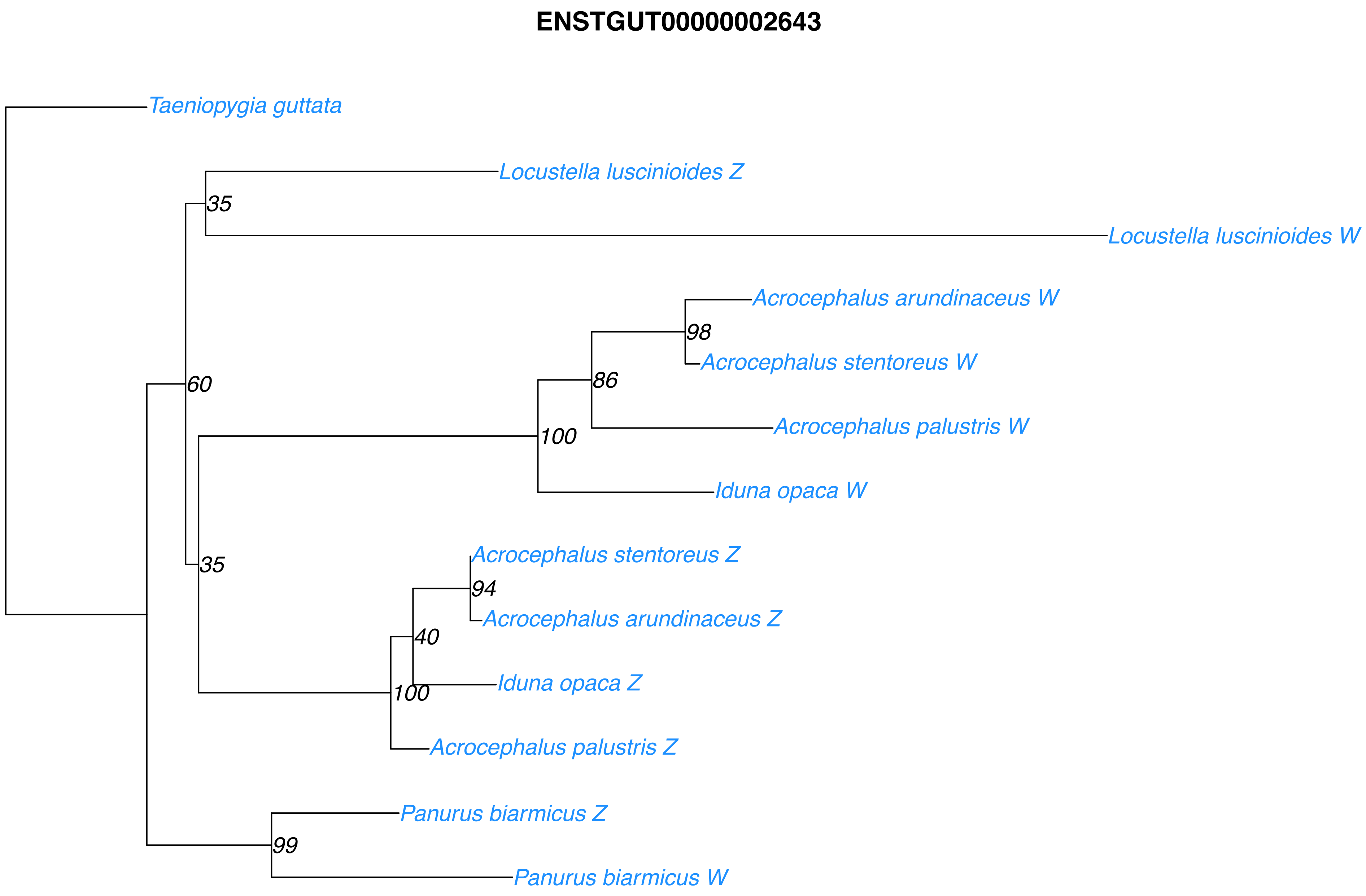
ENSTGUT00000002643
Taeniopygia guttata
Locustella luscinioides Z
35
Locustella luscinioides W
Acrocephalus arundinaceus W
98
Acrocephalus stentoreus W
86
60
Acrocephalus palustris W
100
Iduna opaca W
Acrocephalus stentoreus Z
35
94
Acrocephalus arundinaceus Z
40
Iduna opaca Z
100
Acrocephalus palustris Z
Panurus biarmicus Z
99
Panurus biarmicus W

ENSTGUT00000002623

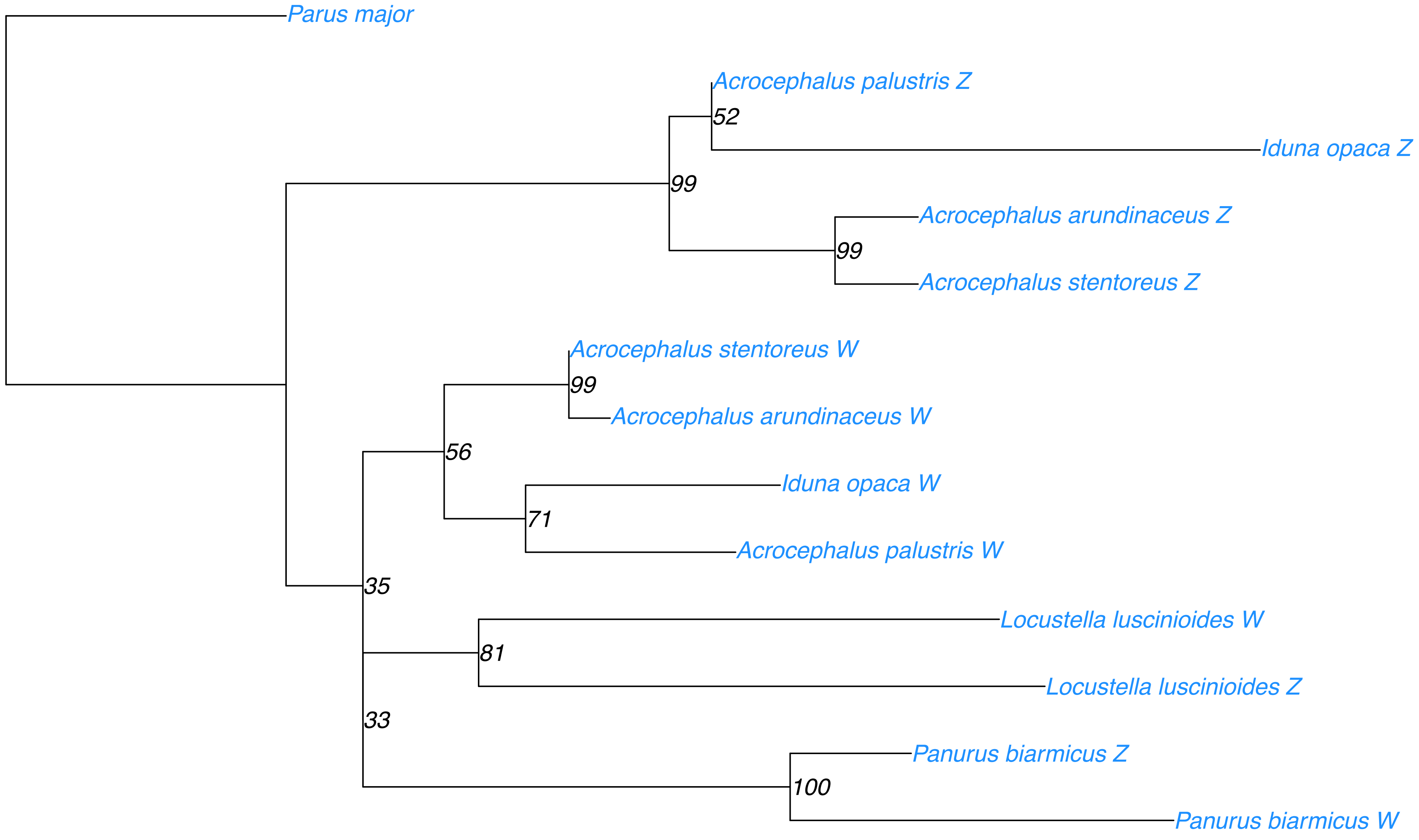

ENSTGUT00000002603

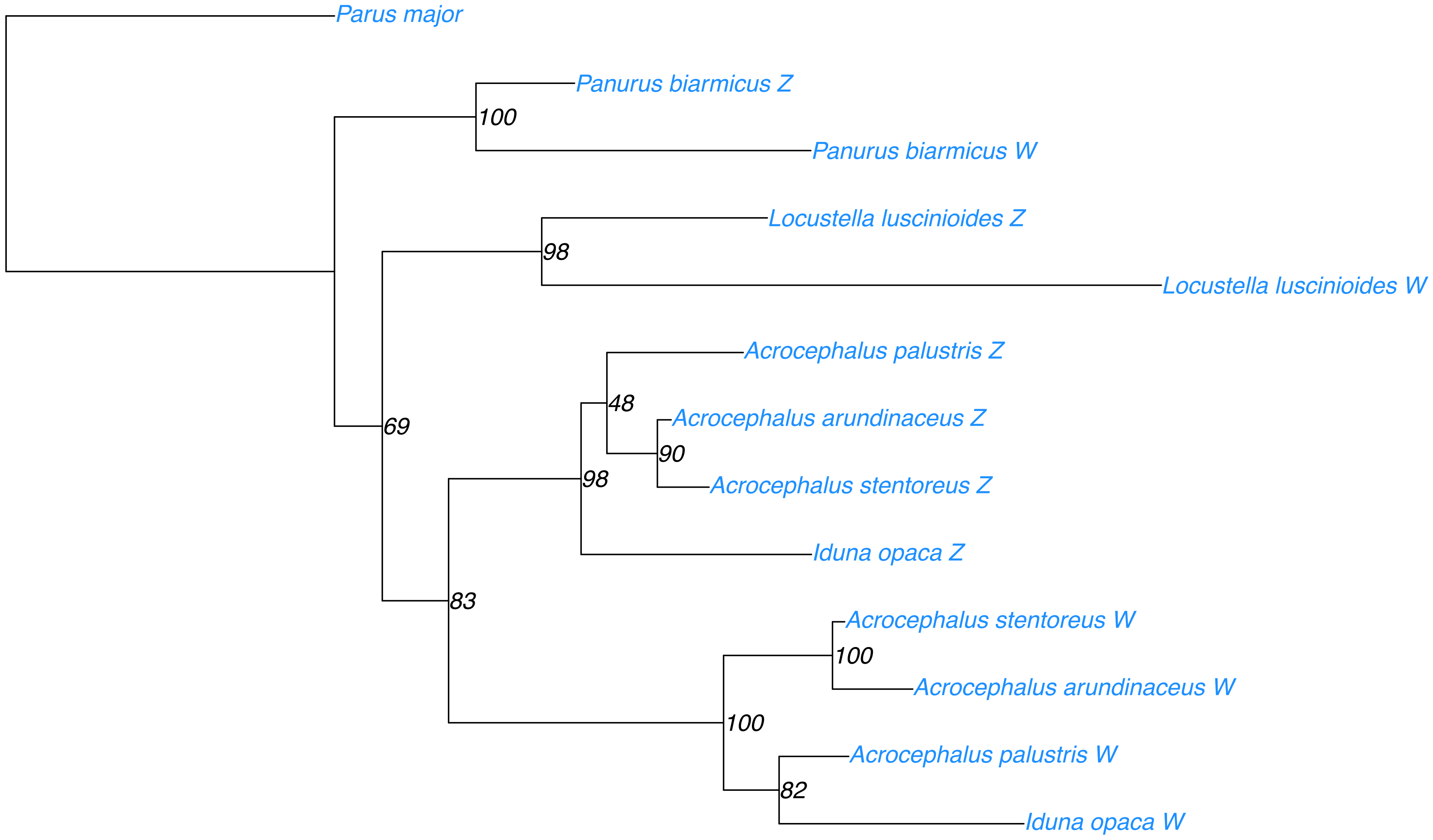

ENSTGUT00000002592

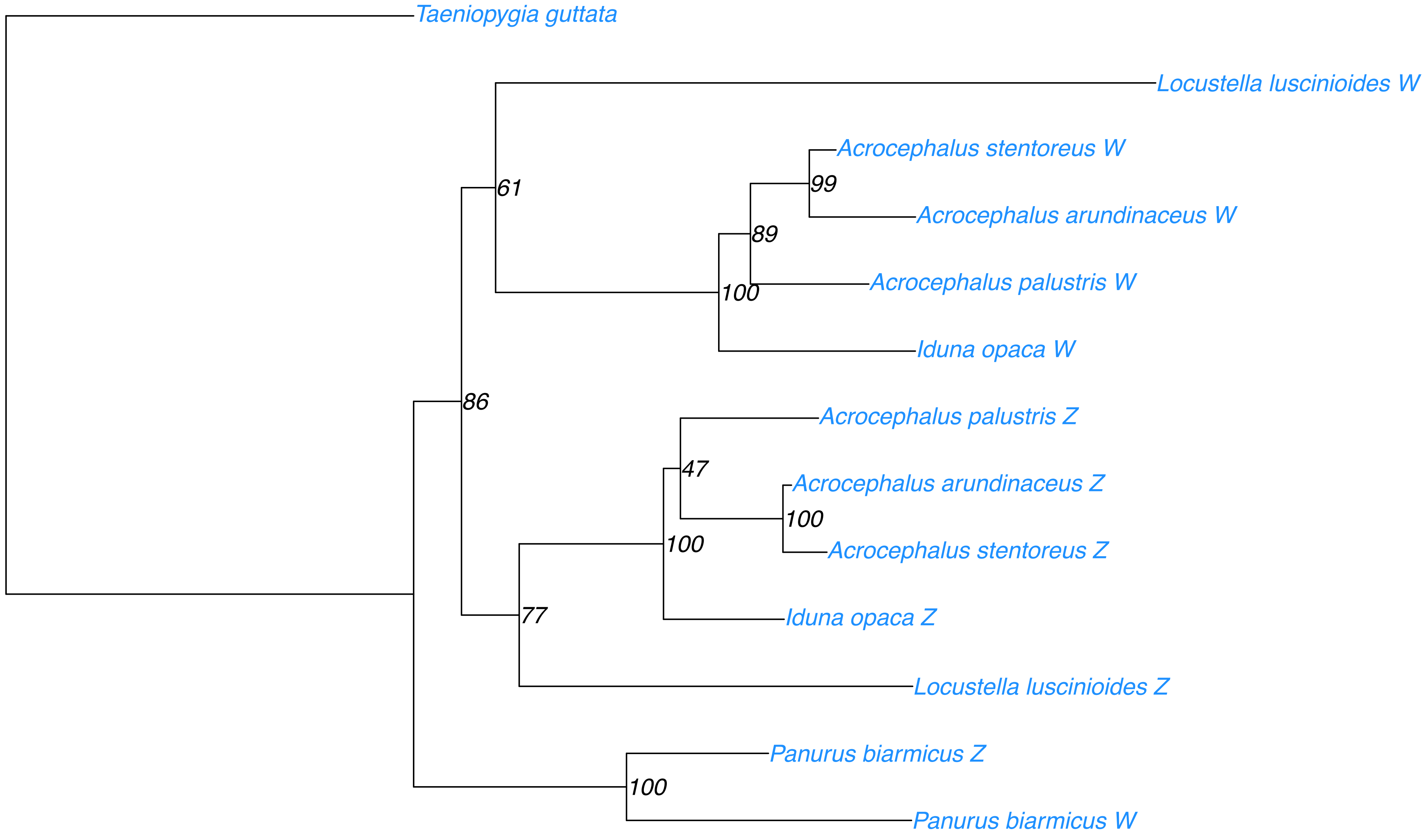

ENSTGUT00000002566

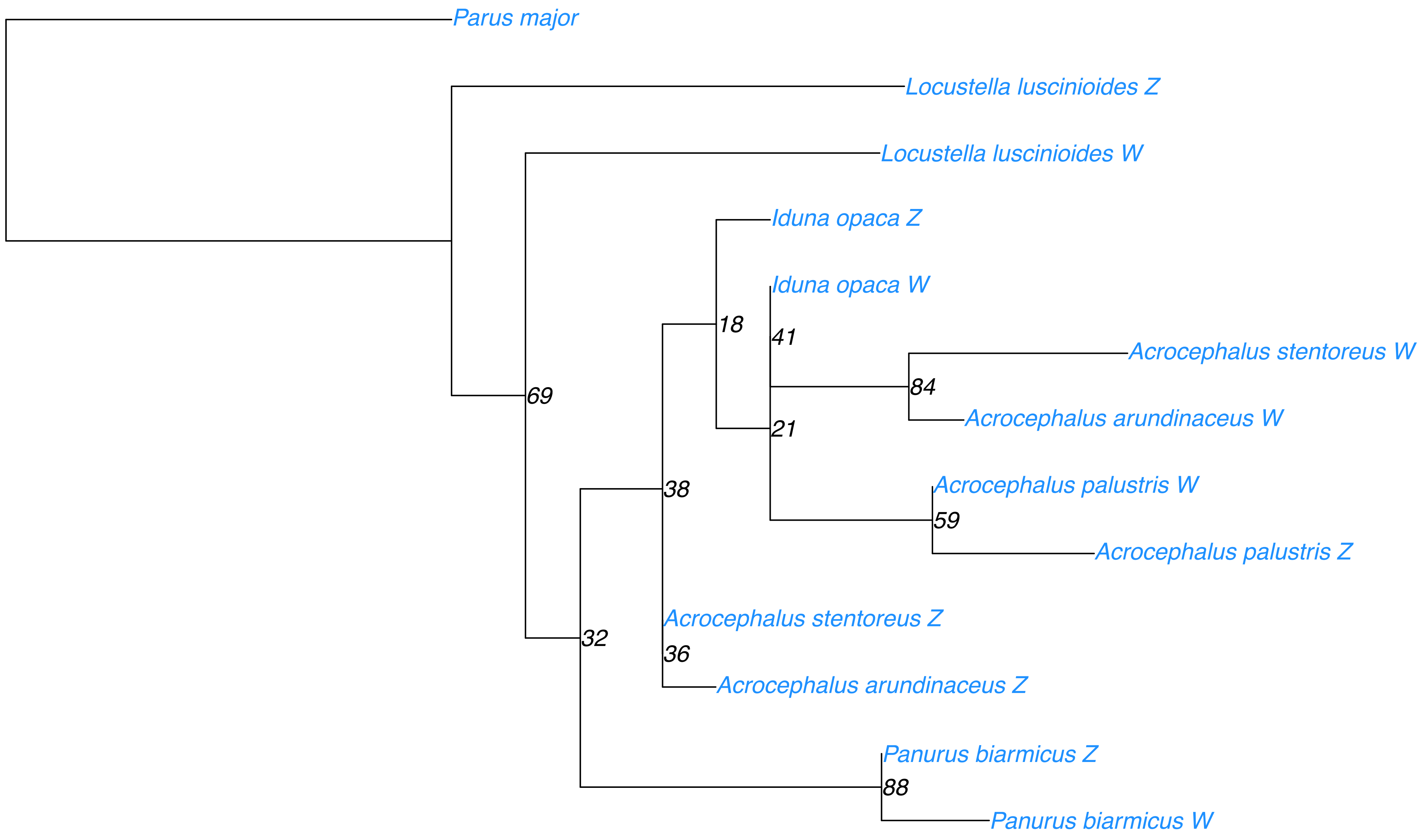

ENSTGUT00000002543

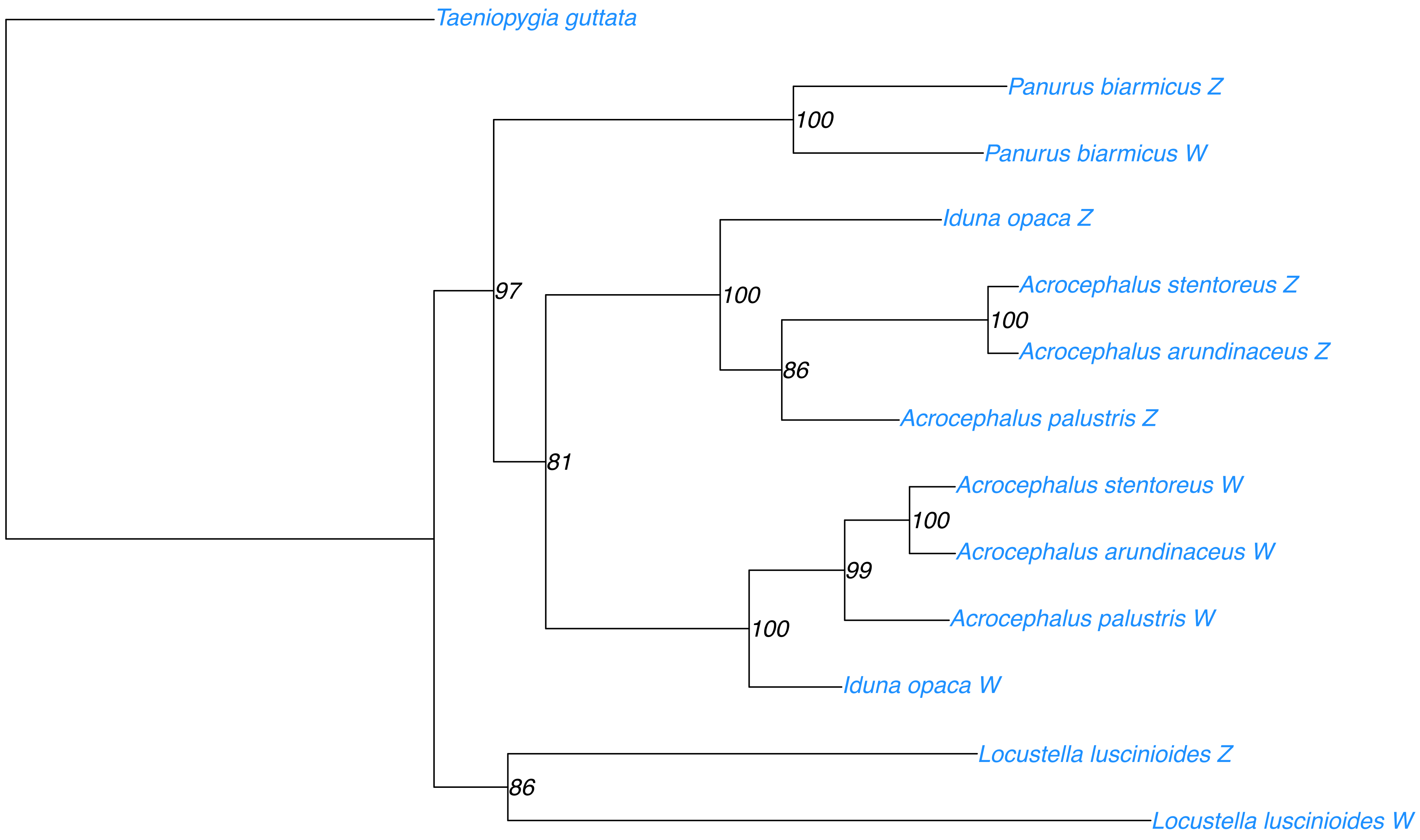

ENSTGUT00000002525

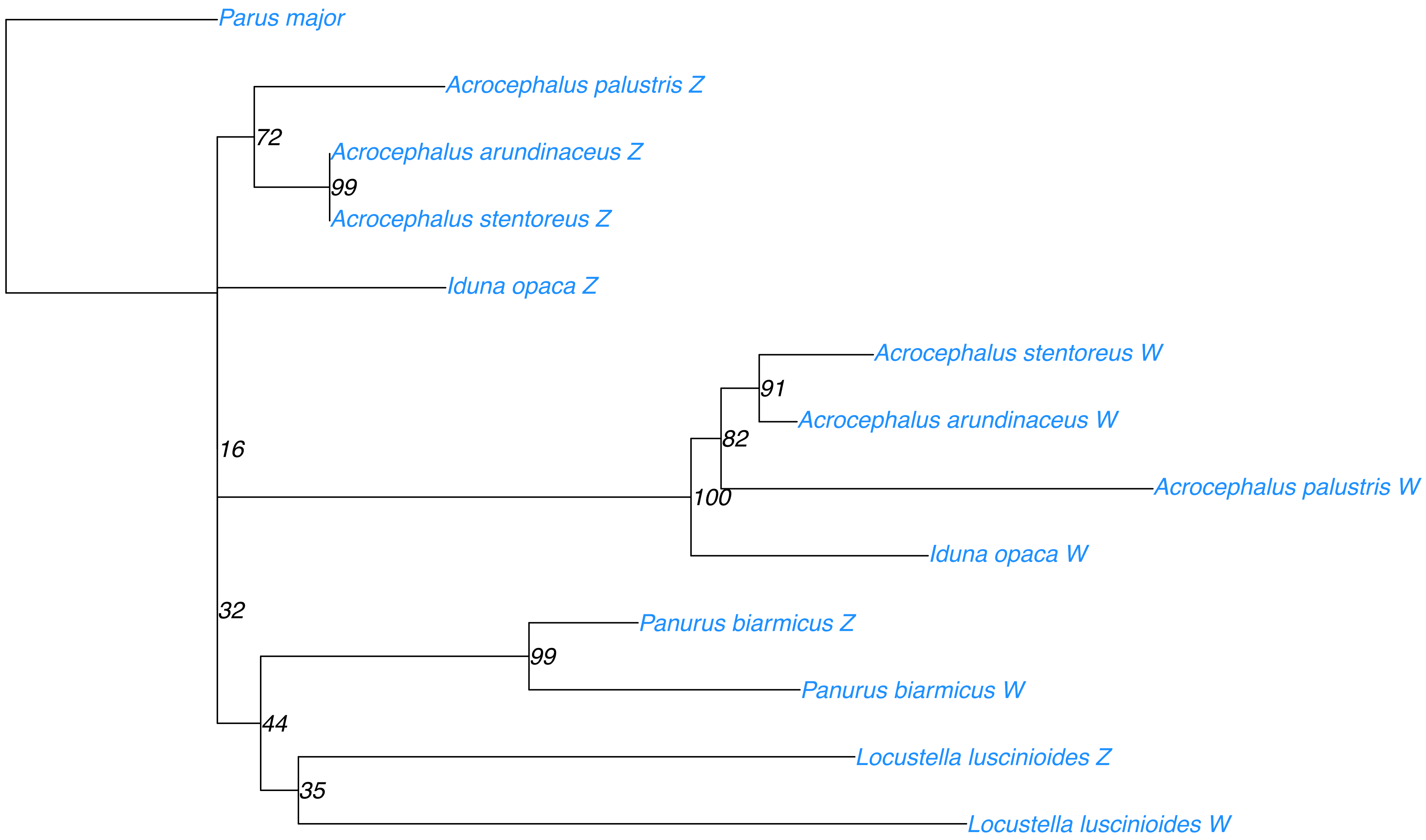

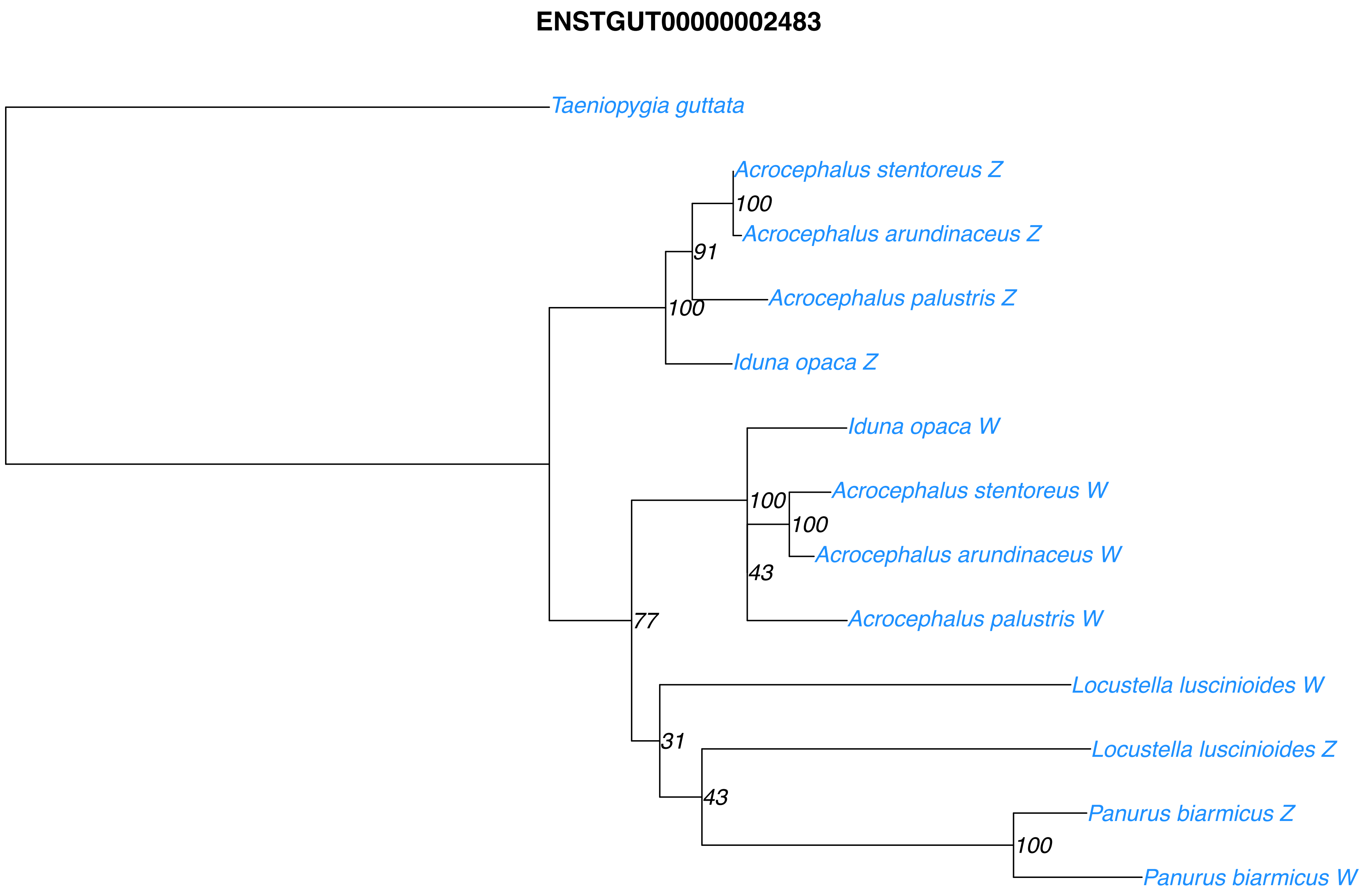

ENSTGUT00000002483
Taeniopygia guttata
Acrocephalus stentoreus Z
100
Acrocephalus arundinaceus Z
91
Acrocephalus palustris Z
100
Iduna opaca Z
Iduna opaca W
100
Acrocephalus stentoreus W
100
Acrocephalus arundinaceus W
43
Acrocephalus palustris W
77
Locustella luscinioides W
31
Locustella luscinioides Z
43
Panurus biarmicus Z
100
Panurus biarmicus W

ENSTGUT00000002340

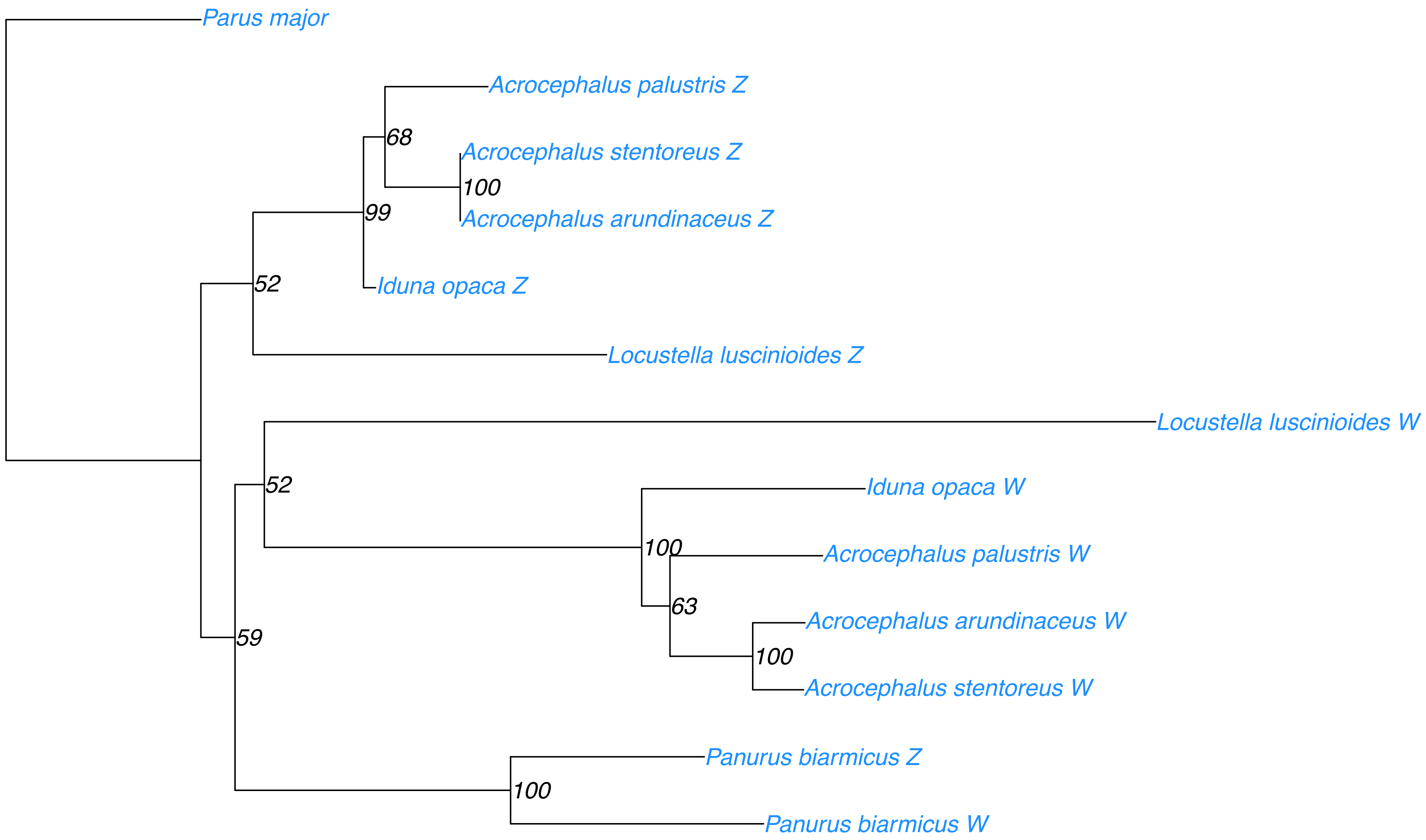

ENSTGUT00000002334

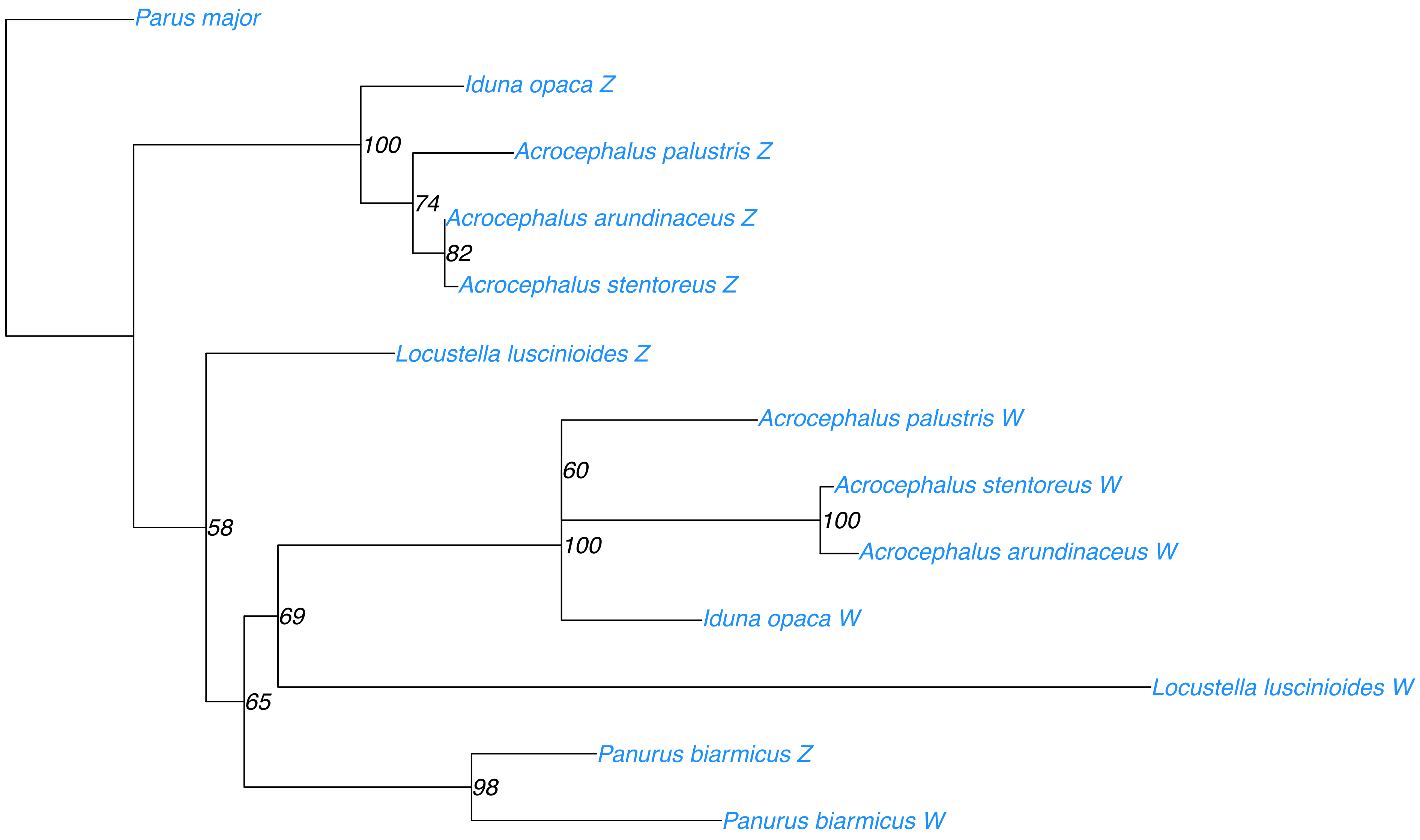

ENSTGUT00000002333

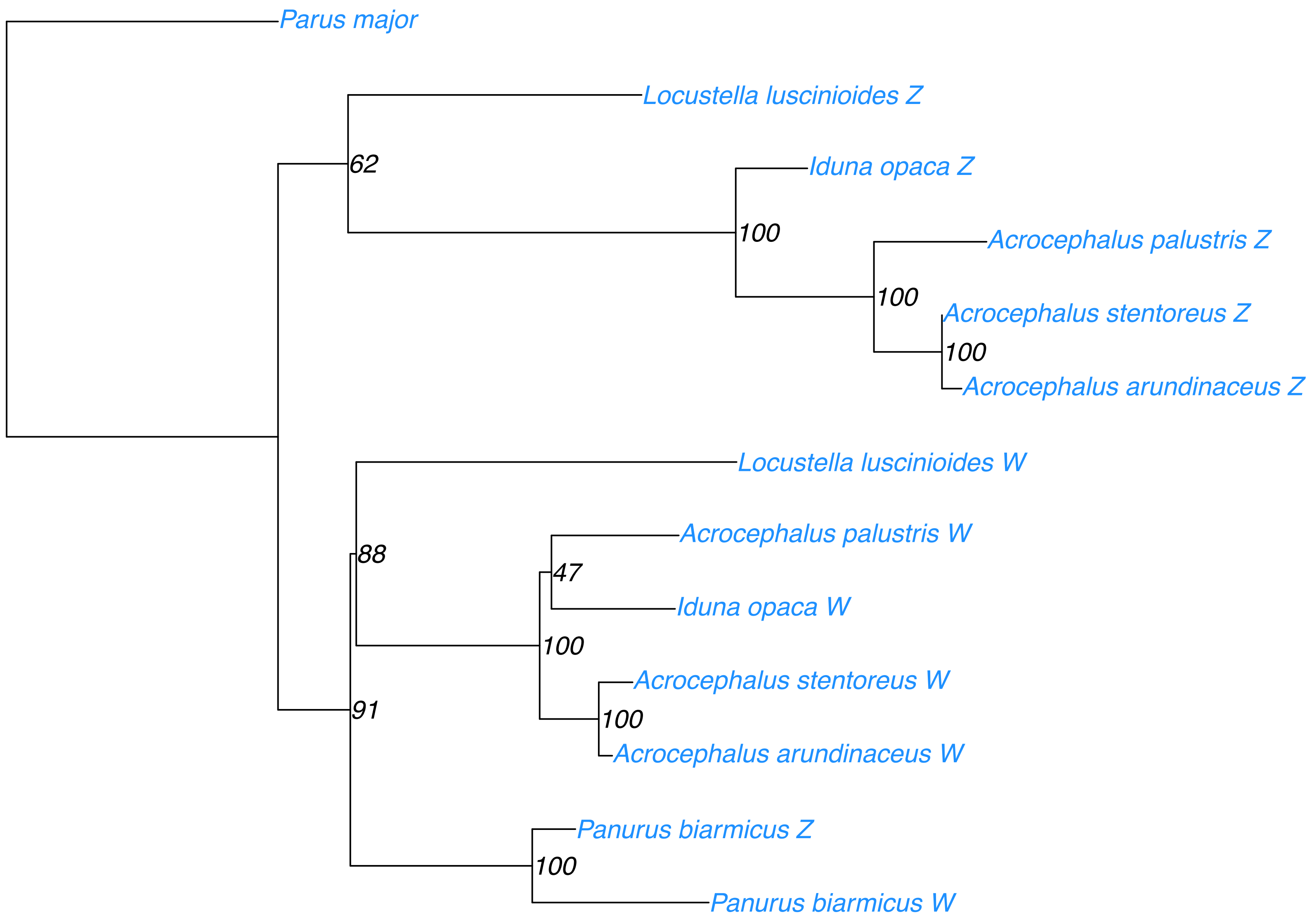

ENSTGUT00000002292

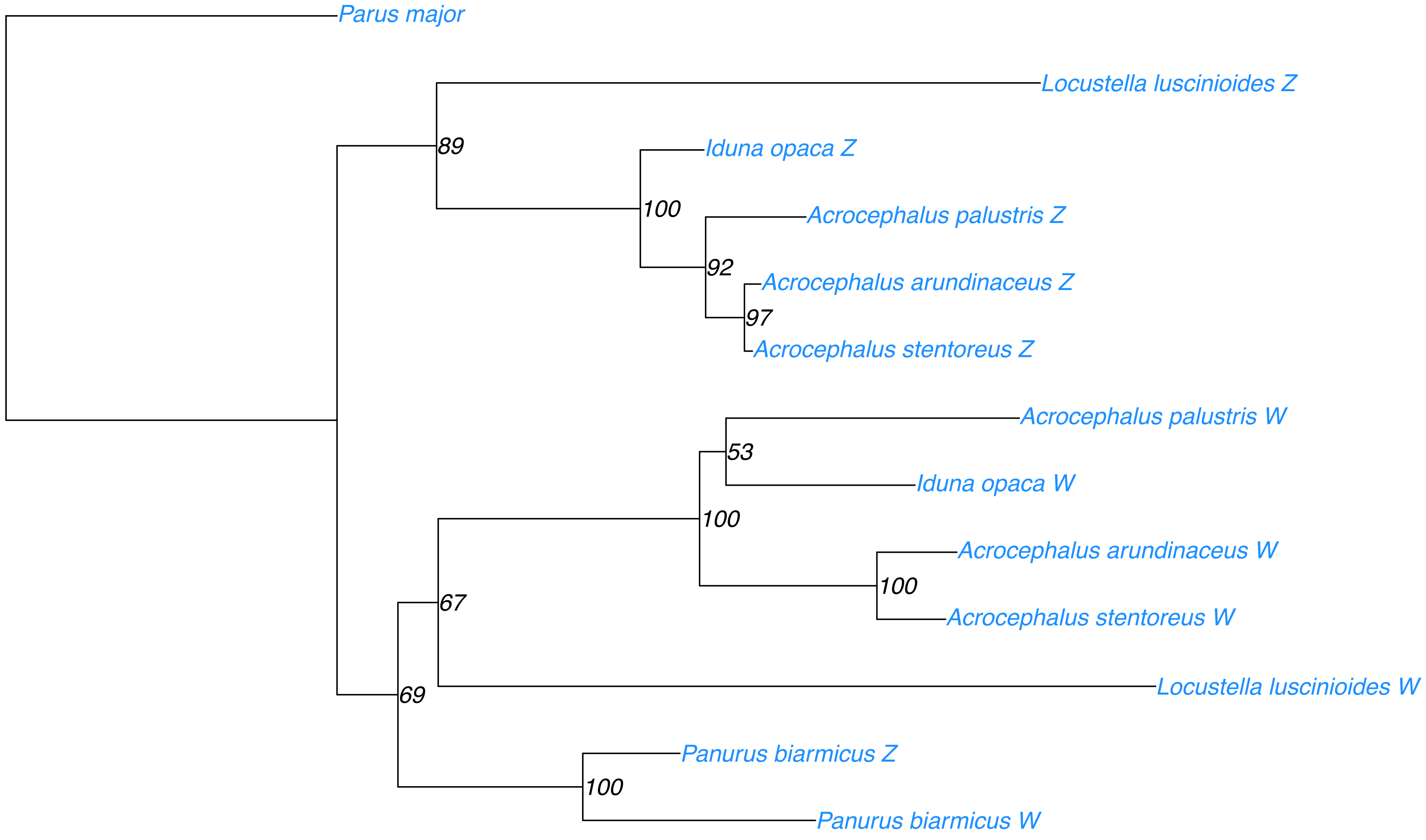

ENSTGUT00000002262

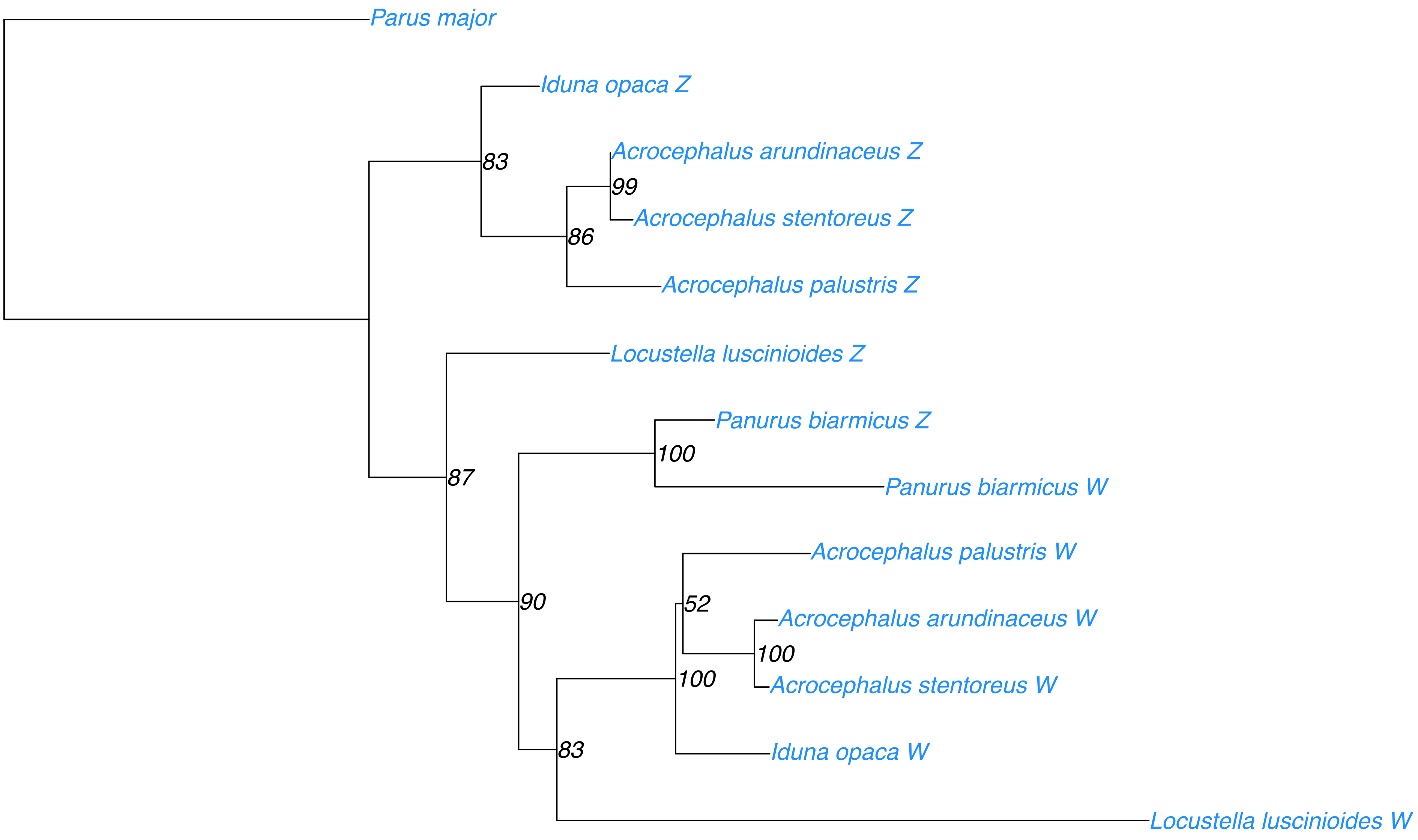

ENSTGUT00000002260

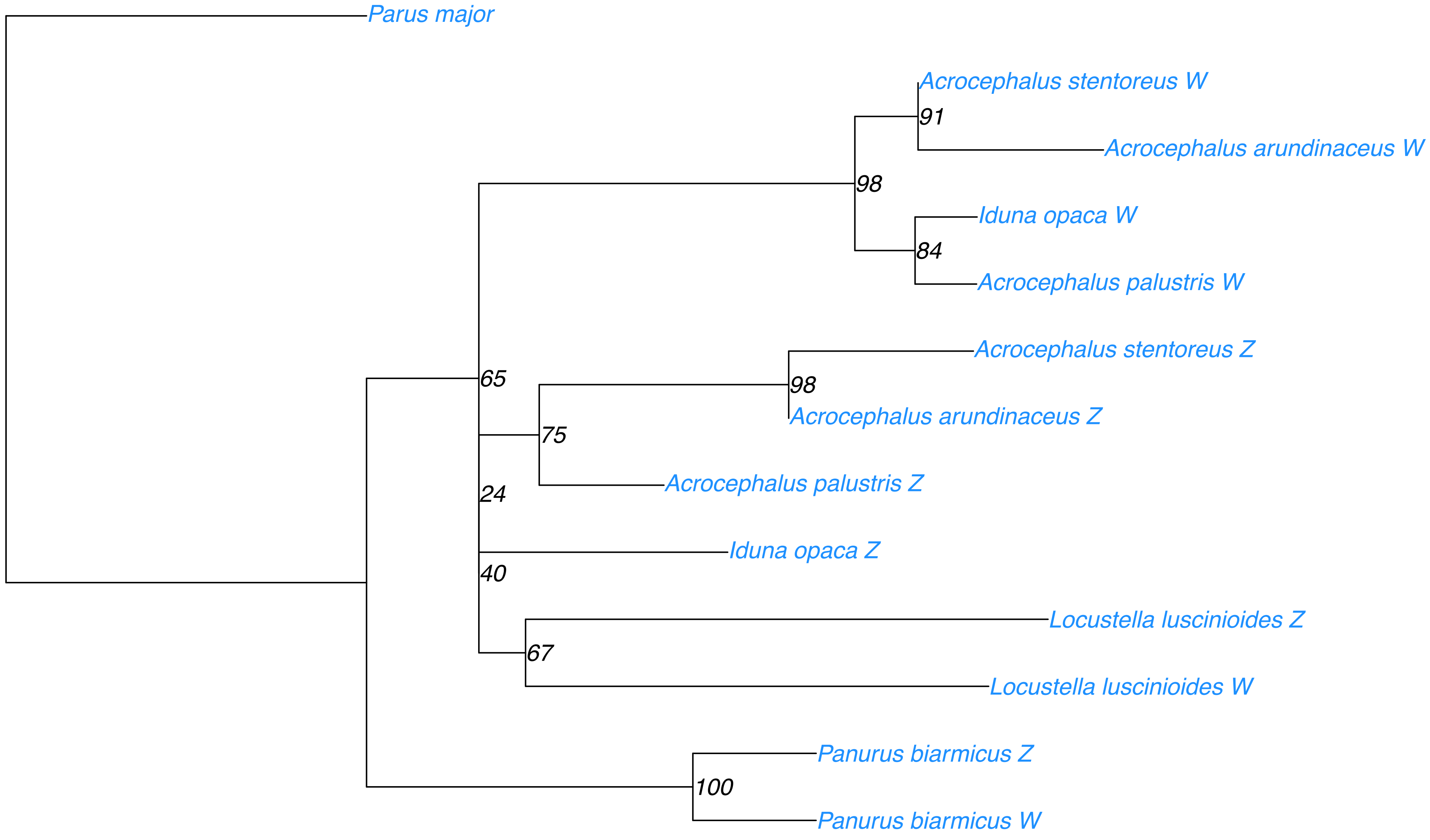

ENSTGUT00000002243

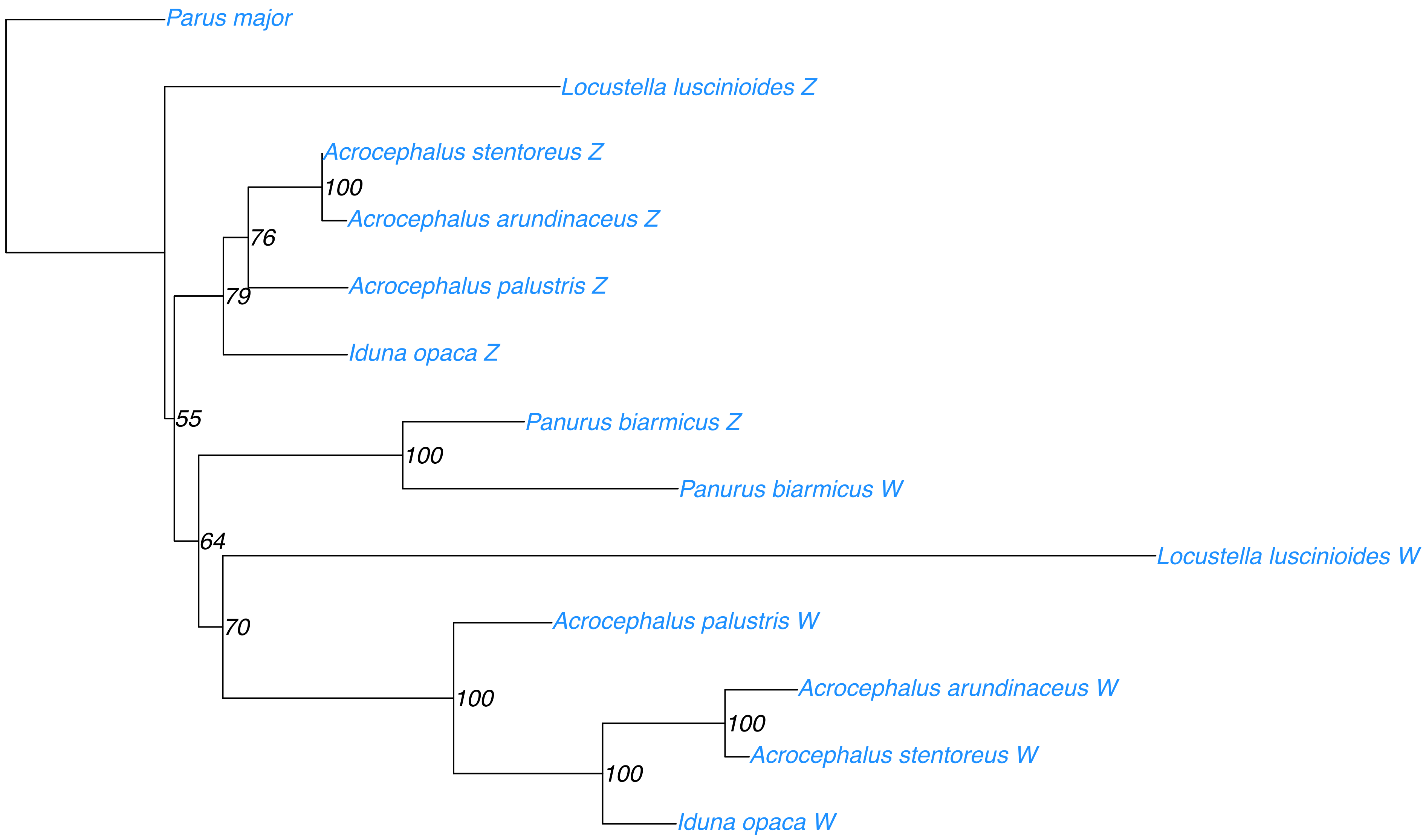

ENSTGUT00000002222

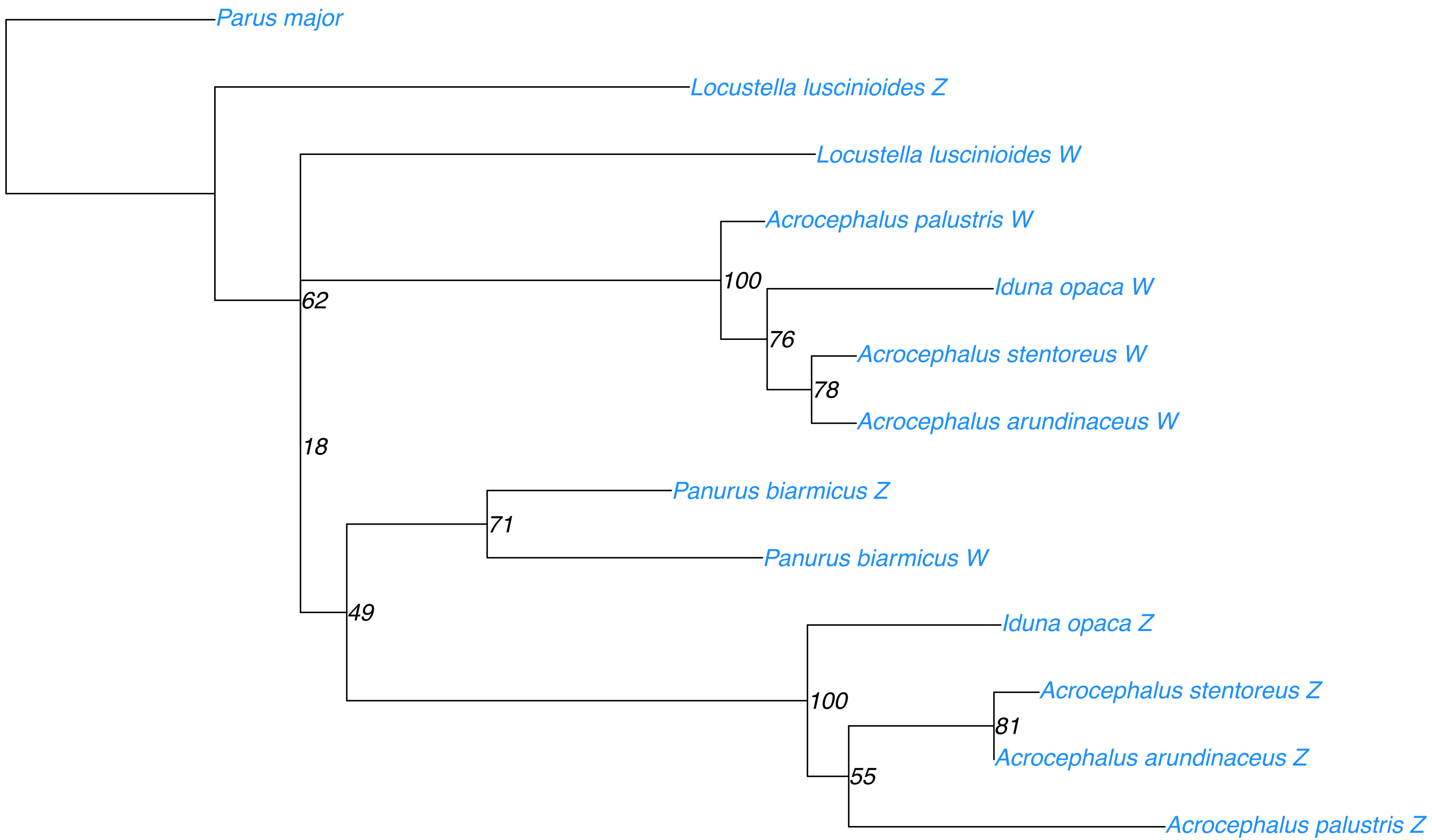

ENSTGUT00000002219

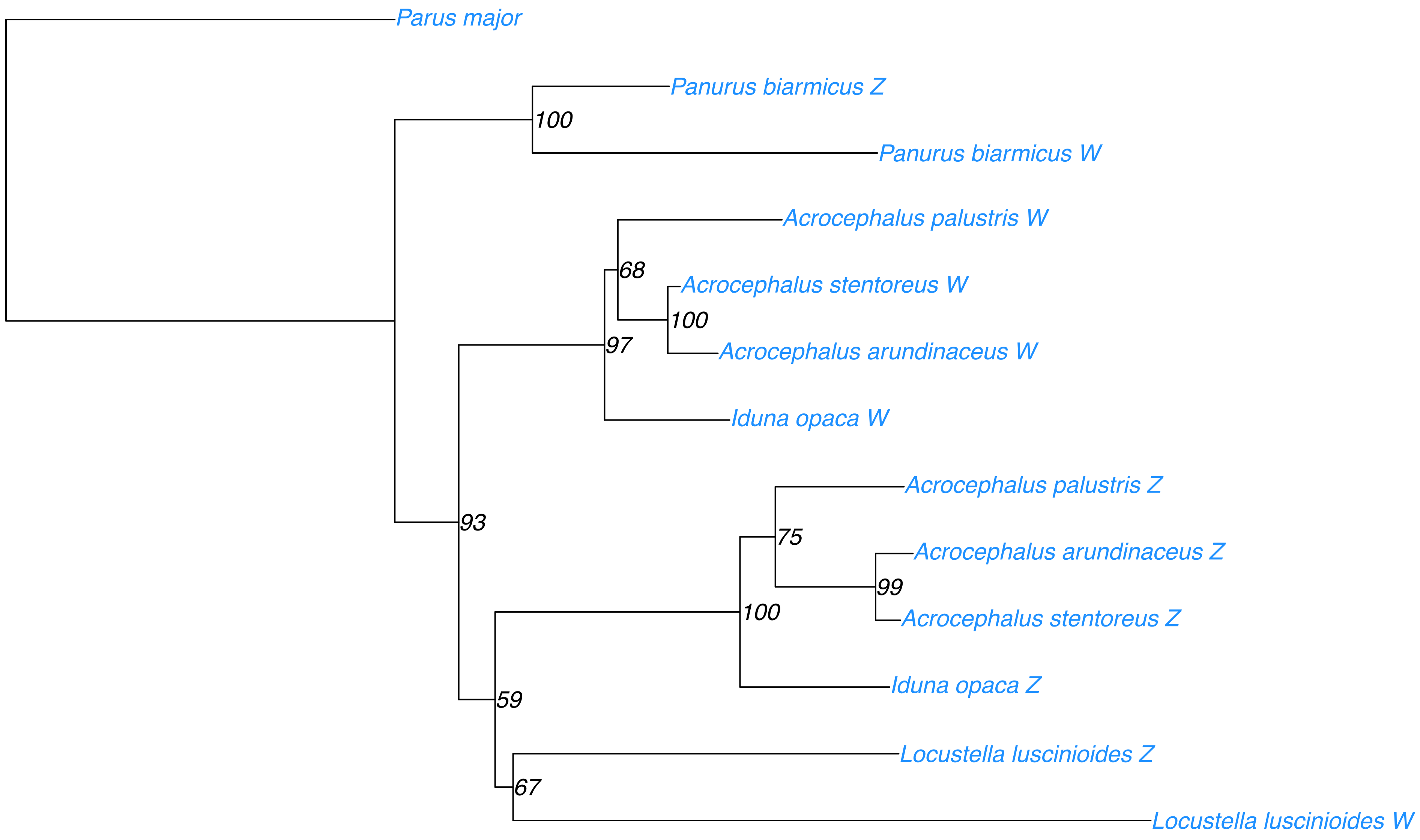

ENSTGUT00000002194

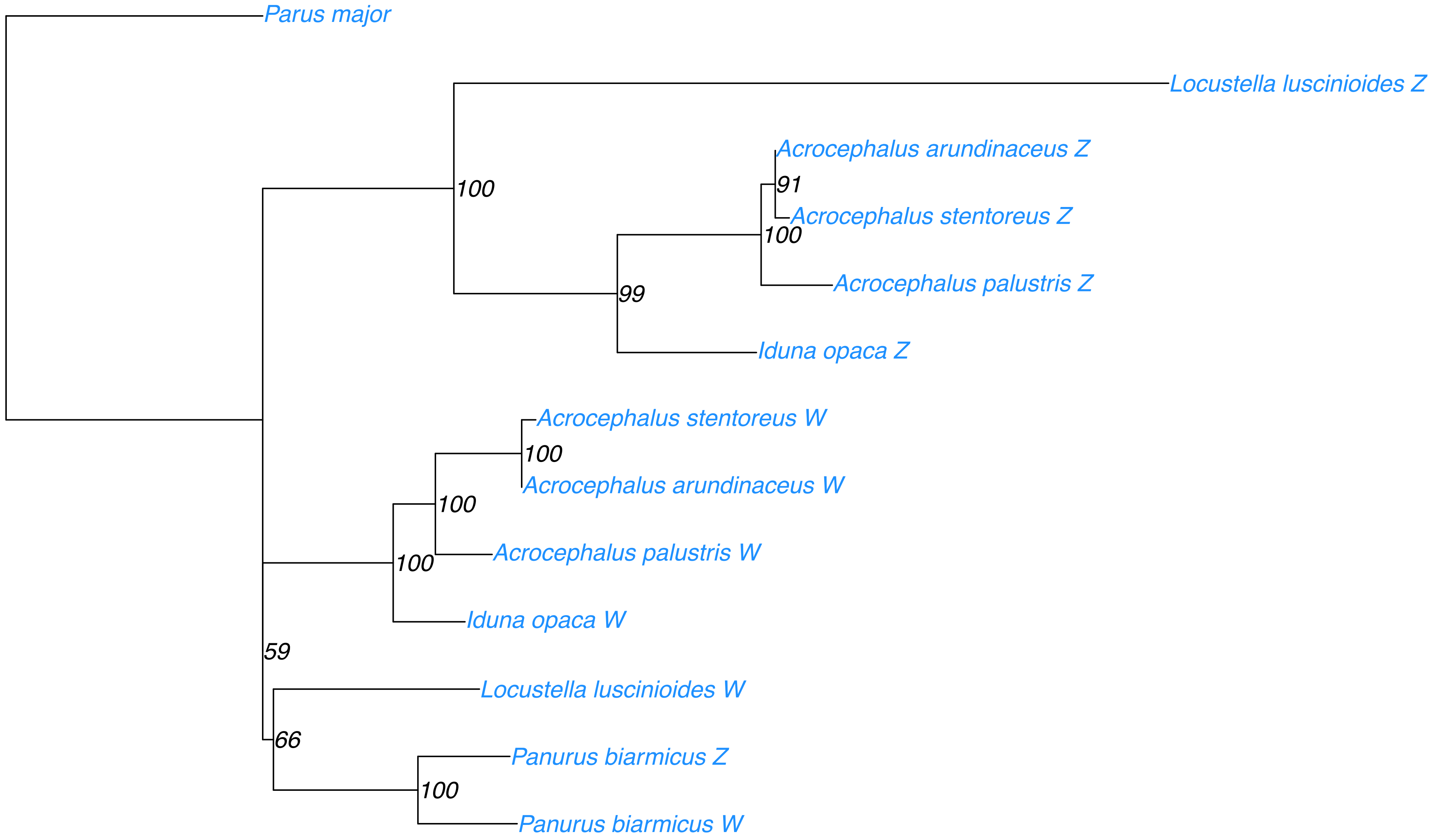

Supplement: msab277_Supplementary_Data [file msab277_supplementary_data.zip › Supplementary Trees.pdf]
